# Syntheses of Highly Functionalized Spirocyclohexenes by Formal [4+2] Annulation of Arylidene Azlactones with Allenoates

**DOI:** 10.1002/ajoc.201800275

**Published:** 2018-06-10

**Authors:** Andreas Eitzinger, Katharina Zielke, Michael Widhalm, Raphaël Robiette, Mario Waser

**Affiliations:** ^1^ Johannes Kepler University Linz Institute of Organic Chemistry Altenbergerstrasse 69 4040 Linz Austria; ^2^ University of Vienna Institute of Chemical Catalysis Währinger Strasse 38 1090 Vienna Austria; ^3^ Université catholique de Louvain Institute of Condensed Matter and Nanosciences Place Louis Pasteur 1 box L4.01.02 1348 Louvain-la-Neuve Belgium

**Keywords:** allenes, annulation, density functional calculations, diastereoselectivity, phosphines

## Abstract

A straightforward phosphine‐catalyzed formal [4+2] annulation between α‐branched allenoates and arylidene azlactones has been developed to access highly functionalized spirocyclohexenes. This cyclization favors the γ‐addition of the phosphine‐activated allenoates over a β′‐addition pathway. Detailed computational studies support the proposed mechanism and provide a reasonable explanation for the observed regioselectivity and the noted effect of the catalyst.

## Introduction

Cyclization reactions of allenoates that contain different acceptor groups have been thoroughly examined in recent years as a means to access (chiral) carbo‐ or heterocycles.^1^ Studies have shown these reactions to be highly dependent on the nature of the reagents, and thus, various catalysts and reaction conditions have been employed to access particular regioisomers and/or products of a certain ring size.^2^, ^3^ Classically, either tertiary amines or phosphines have been used as catalysts in these protocols, which can lead to complementary reaction pathways^2^ and provide access to a variety of (asymmetric) annulation strategies by starting from simple starting materials.^1^, ^2^, ^3^, ^4^, ^5^, ^6^, ^7^, ^8^, ^9^

**Scheme 1:**
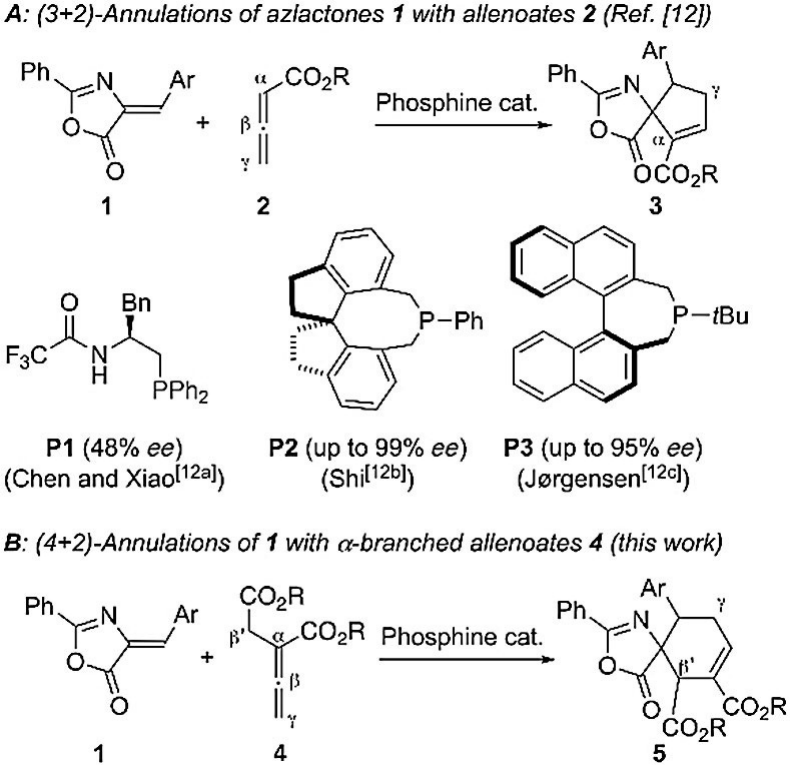
Recently reported [3+2] annulations of azlactones **1** and the herein described [4+2] approach that involves the reaction allenoates **4** with compounds **1** (*ee*=enantiomeric excess).

Azlactones are a prominent and commonly employed family of readily accessible amino acid precursors,^10^ and the corresponding arylidene azlactones **1** have become attractive acceptor molecules in asymmetric (ring‐forming) transformations.^11^, ^12^ The versatility of these compounds to act as two‐carbon synthons in formal [3+2] annulations with allenoates **2** under (chiral) phosphine catalysis was recently reported by the groups of Chen and Xiao,12a Shi,12b and Jørgensen.12c Their studies showed phosphine‐activated allenoates to undergo a highly selective γ‐attack of the acceptor molecule and, in the presence of chiral phosphines, yield chiral cyclopentene‐based masked α‐amino acids **3** in high yields with excellent enantioselectivity (Scheme 1 A).^12^

We recently showed α‐branched allenoates **4** to perform in a complementary manner to that of allenoates **2** in a reaction with *ortho*‐quinone methides.^13^ We then became interested in examining whether these allenoates could serve as four‐carbon synthons in the corresponding formal [4+2] annulation reaction^6^, ^7^ with arylidene azlactones **1** (Scheme 1 B). The success of this transformation would provide a method to access highly functionalized cyclohexene‐based α‐amino acid derivatives in a straightforward manner.

## Results and Discussion

### Reaction development

Our initial screening of the reaction conditions was carried out by treating parent acceptor **1 a** with diethyl allenoate ester **4 a** as our model reaction. An overview of the most interesting results from a detailed screening of different catalysts and conditions is shown in Table 1. All of these reactions were performed for 20 h in the presence of 3 equiv of allenoate **4 a** and 1 equiv of **1 a**. Other **4 a**/**1 a** ratios were examined, but lower yields were afforded, most likely the result of the allenoates participating in side reactions.^14^

**Table 1:** Optimization of the reaction conditions for the [4+2] annulation of arylidene azlactone **1 a** with allenoate **4 a**.^[a]^

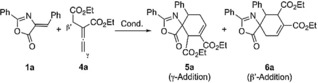							
Entry	Catalyst	Solvent	Base	*T* [°C]	**5 a**/**6 a** ^[b]^	d.r.^[c]^ **5 a**	Yield **5 a** ^[d]^
1	PBu_3_ (20 %)	CH_2_Cl_2_	Cs_2_CO_3_ (5 equiv)	25	4.0:1	5.2:1	29 %
2	PPh_3_ (20 %)	CH_2_Cl_2_	Cs_2_CO_3_ (5 equiv)	25	–	–	n.r.
3	DABCO (20 %)	CH_2_Cl_2_	Cs_2_CO_3_ (5 equiv)	25	–	–	n.r.
4	PBu_3_ (20 %)	CH_2_Cl_2_^[e]^	Cs_2_CO_3_ (5 equiv)	25	7.0:1	8.6:1	38 %
5	PBu_3_ (100 %)	CH_2_Cl_2_^[e]^	Cs_2_CO_3_ (5 equiv)	25	5.8:1	6.8:1	39 %
6	PBu_3_ (20 %)	THF^[e]^	Cs_2_CO_3_ (5 equiv)	25	5.0:1	5.0:1	58 %
7	PBu_3_ (20 %)	toluene^[e]^	Cs_2_CO_3_ (5 equiv)	25	5.8:1	5.0:1	48 %
8	PBu_3_ (20 %)	toluene^[e]^	–	25	3.8:1	5.0:1	50 %
9	PBu_3_ (20 %)	toluene^[e]^	–	60	3.8:1	4.2:1	65 %
10	PBu_3_ (20 %)	THF^[e]^	–	60	3.6:1	4.0:1	71 %
11	PEt_3_ (20 %)	THF^[e]^	–	60	3.3:1	4.1:1	73 %
12	PEt_3_ (20 %)	THF^[e]^	Cs_2_CO_3_ (5 equiv)	60	3.2:1	2.6:1	65 %
13	PCy_3_^[f]^ (20 %)	THF^[e]^	–	60	2.8:1	1.5:1	22 %
14	PPhMe_2_ (20 %)	THF^[e]^	–	60	4.5:1	6.1:1	60 %
15	PPh_2_Bu (20 %)	THF^[e]^	–	60	4.2:1	3.0:1	16 %
16	**P2** (20 %)	THF^[e]^	–	60	–	–	n.r.^[f]^
17	**P3** (20 %)	THF^[e]^	–	60	–	–	n.r.

**Figure 1:**
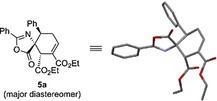
Single‐crystal X‐ray analysis of the major diastereomer of **5 a**.

[a] Reagents and conditions: **1 a** (0.1 mmol) and **4 a** (0.3 mmol) for 20 h. [b] Ratio determined by 1H NMR analysis of the crude product. [c] See Figure 1 for the relative configuration of the major diastereomer (d.r.=diastereomeric ratio). [d] Isolated yield of the mixture of diastereomers is reported. [e] The reaction mixture included the addition of MS (4 Å). [f] Cy=cyclohexyl, n.r.=no reaction.

Initial experiments that used either catalytic amounts of PBu_3_, PPh_3_, or 1,4‐diazabicyclo[2.2.2]octane (DABCO) in the presence of Cs_2_CO_3_ in CH_2_Cl_2_ revealed that only PBu_3_ led to the targeted [4+2] annulation reaction (Table 1, entries 1–3). The formation of adduct **5 a**, which originates from the γ‐addition of the allenoate to the acceptor, was clearly favored over the generation of β′‐addition product **6 a** (Table 1, entry 1). We also realized that the use of degassed solvents and the addition of molecular sieves had a beneficial effect on the product yield (Table 1, entry 1 vs. 4). Accordingly, all further reactions were performed in dry, degassed solvents and in the presence of 4 Å molecular sieves (MS).

**Scheme 2:**
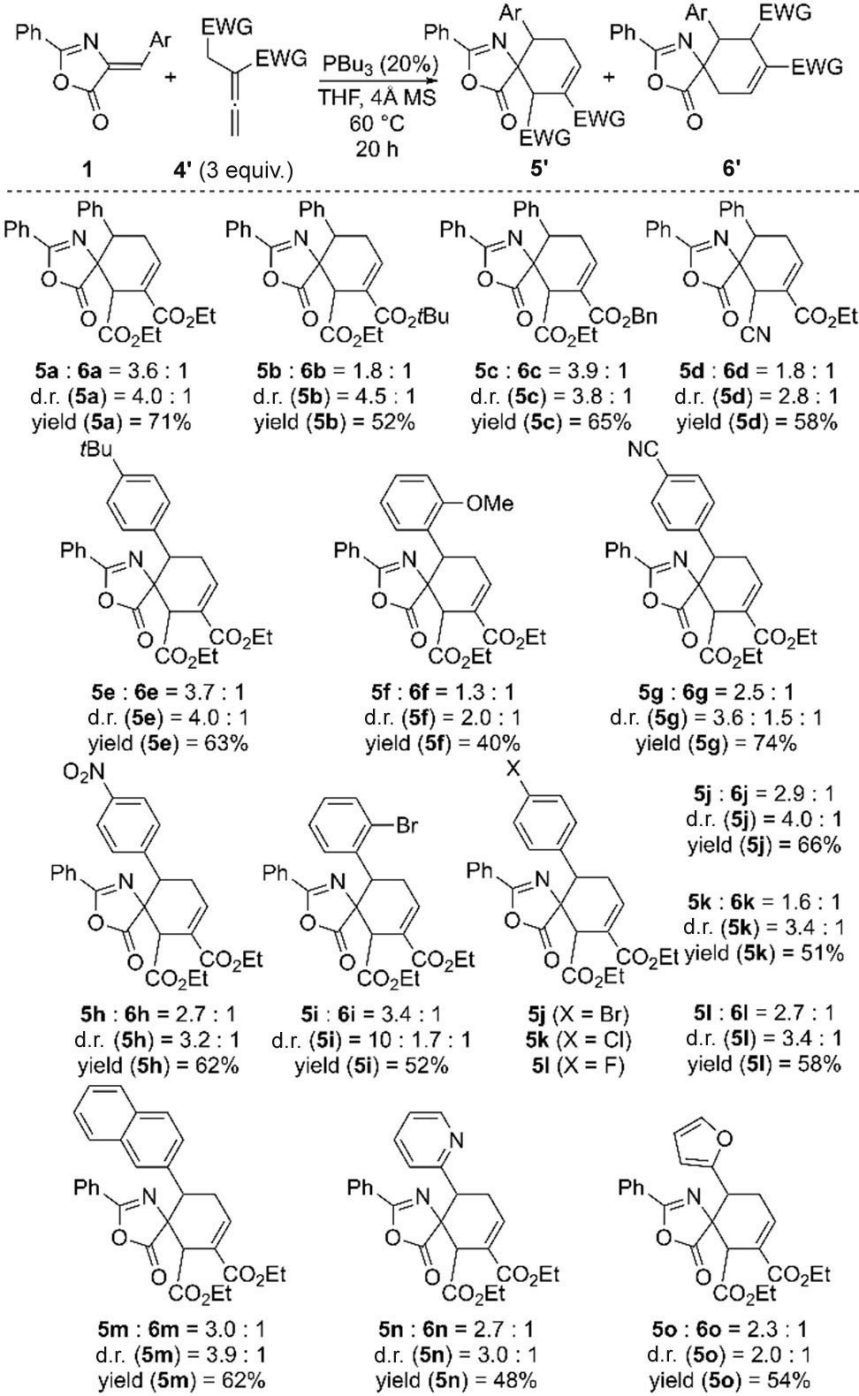
Investigation of the scope of the PBu_3_‐catalyzed [4+2] annulation of allenoate **4′** and acceptor **1**. Reactions were carried out on 0.1–0.25 mmol scale. The **5′**/**6′** ratio was determined by ^1^H NMR analysis of the crude reaction mixture. Compound **5′** was isolated in each case as a diastereomeric mixture after column chromatography. Analytically pure samples of single diastereomers of some derivatives were obtained by preparative HPLC.^16^

Product **5 a** was always obtained as a mixture of two diastereomers, and the relative configuration of the major diastereomer was unambiguously proven by single‐crystal X‐ray diffraction studies (Figure 1).^15^ Notably, the other two possible diastereomers of **5 a** were not detected, but we later observed the formation of additional diastereomers of some of the other derivatives (see Scheme 2).

Using a stoichiometric amount of PBu_3_ had no positive effect on the yield (Table 1, entry 5). Among the different examined solvents, THF and toluene led to better results than those initially obtained by using dichloromethane (DCM, Table 1, entries 6 and 7). We also found that the reaction afforded a slightly higher yield (but with somewhat lower regioselectivity) in the absence of a base (Table 1, entry 8). The yield was further increased by carrying out the reaction at an elevated temperature, and under these conditions, THF gave a slightly better yield than that obtained in toluene (Table 1, entries 9 and 10).

In the presence of different achiral tertiary phosphines (PR_3_), we realized that phosphines that contain short alkyl groups (e.g., PEt_3_ and PBu_3_) performed significantly better than bulkier ones (Table 1, cf. entries 10, 11, and 13). It was also shown that PEt_3_ in the presence of a base at a higher reaction temperature had a detrimental effect on the yield and diastereoselectivity (Table 1, entry 12). Among aryl‐containing phosphines, only PPhMe_2_ allowed us to achieve the [4+2] annulation with a reasonable product yield, whereas diaryl‐ or triarylphosphines performed significantly worse (Table 1, entries 2, 14, 15).

Unfortunately, this observed trend in the reactivity also lowered the likelihood to successfully introduce an enantioselective protocol, as a majority of chiral phosphines that can be utilized in such allenoate cyclizations are typically sterically demanding and contain aryl groups.^12^ Nevertheless, we did perform a few reactions with chiral phosphines such as compounds **P2** and **P3** (both of which have been successfully used in [3+2] annulations of azlactones **1** with allenes **2** as shown in Scheme 1, 12b, 12c), but with absolutely no success (Table 1, entries 16 and 17). This same lack of reactivity was also observed with other known chiral phosphines.^16^

Next, we investigated the scope of the racemic protocol for this reaction by using various substituted acceptors **1** and allenoates **4** in the presence of PBu_3_ (Scheme 2) The reactions with PBu_3_ were easier to handle and more robust, as PEt_3_ was found to be more sensitive towards oxidation. The isolated yields, in most cases, were within the same range as that obtained for the model reaction. However, the resulting diastereoselectivity was clearly influenced by the electronic and steric properties of the starting materials, and, in some cases, we observed the formation of a third diastereomer (Scheme 2, see compounds **5 g** and **5 i**). For all of the substrates, the formation of γ‐addition product **5′** was favored over that of β′‐addition product **6**, although the selectivity was not as pronounced in some cases. This was especially observed by changing the ester group of the allenoate (Scheme 2, see products **5 b**/**6 b** and **5 d**/**6 d**) and by introducing an *ortho*‐methoxy group to the aryl group of acceptor **1** (Scheme 2, products **5 f**/**6 f**). In all cases, the isolation of β′‐adduct **6** was difficult, as these compounds coeluted with the allenoate degradation products, the mixture of which could not be separated by column chromatography. Therefore, no isolated yields for compound **6** have been reported.

As for the potential transformations of spiro products **5′**, we carried out a quick test to determine if the selective hydrolysis of the azlactone is possible. Under ambient acidic conditions, we found that the azlactone was easily hydrolyzed without a reaction occurring at the other ester functionalities.^16^

### Computational studies

**Scheme 3:**
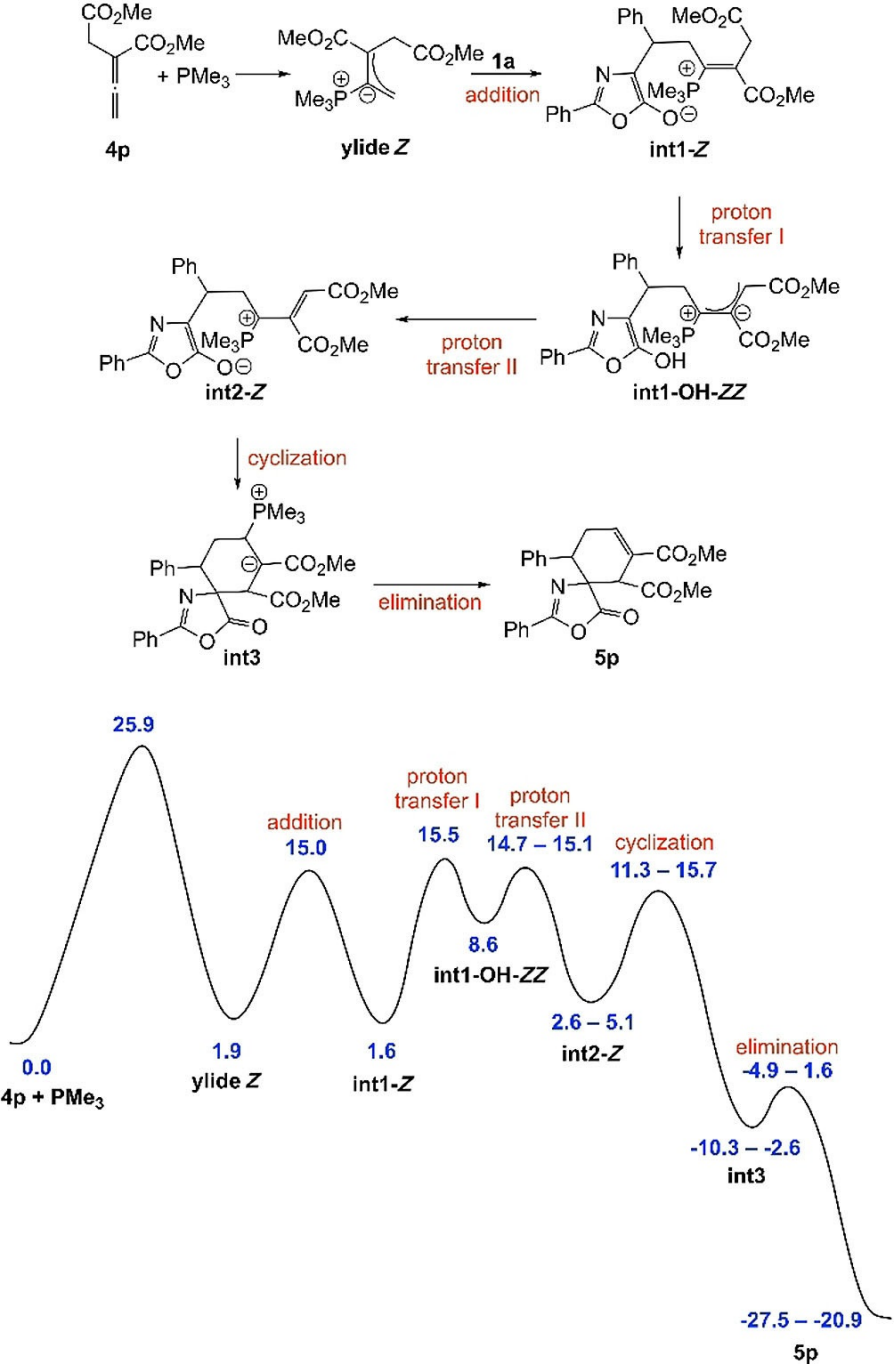
Computed global mechanistic sequence and free energy (kcal mol^−1^ relative to reactants) profile. (A range of values for the free energies of the four diastereomeric pathways are reported.)

To gain a fundamental understanding of the mechanism and the origin of the regio‐ and stereoselectiviy of this formal [4+2] annulation, we have examined the free energy profile of the reaction between acceptor **1 a** and allenoate **4 p** with trimethylphosphine as the catalyst (Scheme 3). The calculations were carried out at the M06‐2X‐D3/6–311+G**//B3LYP‐D3/6‐31G* level of theory, which included a continuum description of tetrahydrofuran as the solvent.^17^, ^18^

All potential mechanisms for the formation of **5 p** from **1 a** and **4 p** were investigated.^18^ The mechanistic sequence of the predicted most favored pathway is shown in Scheme 3. The first step involves formation of **ylide** ***Z*** by the addition of PMe_3_ to allenoate **4 p**. This step is only slightly endergonic (1.9 kcal mol^−1^) but occurs through a significant free energy barrier (25.9 kcal mol^−1^). The resulting ylide then undergoes addition to **1 a** to form isoenergetic **int1‐*Z*** through a low‐lying transition state (15.0 kcal mol^−1^). Next, migration of the double bond occurs to yield **int2** (four diastereomers are possible and the respective energy values/ranges for the different intermediates and transition states are shown in Scheme 3). Many mechanisms can be envisaged for this double‐bond migration,^18^ but our calculations indicate that the most favored pathway consists of two successive intramolecular proton transfer reactions that involve the azlactone moiety. Intermediate **int2** can then undergo ring closure to form zwitterion **int3**, which eventually undergoes rapid elimination to yield product **5 p** (as diastereomers).

The double intramolecular proton transfer process can potentially lead to four diastereomeric **int2** intermediates. Our calculations, however, predict the stereoselective formation of **int1‐OH‐*ZZ*** and then of **int2** with two methyl ester groups in a *Z* relationship. These two diastereomers can eventually lead to the four possible diastereomers of **5 p**. Thus, given the predicted irreversibility of the double proton transfer, the overall diastereoselectivity of the formal [4+2] annulation must depend on the double proton transfer and cyclization processes. The small energy differences between the diastereomeric transition states of these processes (see Supporting Information for full data^18^) make it difficult to predict the overall diastereoselectivity of the formal [4+2] annulation reaction. This complexity in the origin of the stereoselectivity and the computed small energy differences between the various diastereomeric pathways are in good agreement with the observed variations in the diastereomeric ratios, according to small structural changes in the substrates (see Scheme 2).

**Figure 2:**
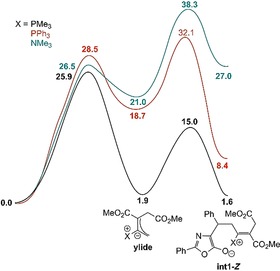
Influence of the nature of the catalyst (free energy values in kcal mol^−1^).

Our results indicate that the rate‐determining step of the formal [4+2] annulation is the formation of the ylide (see Scheme 3). Experimentally, we observed that the nature of the catalyst has a drastic influence on the reactivity. Non‐hindered trialkylphosphines catalyzed the reaction efficiently, whereas hindered phosphines such as Ph_2_PMe, PPh_3_, **P2**, and **P3** led to low or no conversion (see Table 1). In addition, tertiary amines such as DABCO (Table 1 entry 3) and Et_3_N were shown as inefficient catalysts for this [4+2] annulation reaction. To identify the factors responsible for these observations, we explored the first two steps of the process, that is, the formation of the ylide formation and its addition to azlactone **1 a** in the presence of PPh_3_ or NMe_3_ as the catalyst. The obtained results reveal that the inefficiency of these catalysts to promote the formal [4+2] annulation reaction is mainly from an increase in the endergonic character of the ylide formation (Figure 2). The destabilizing steric interactions between the ylide and PPh_3_ and the intrinsic lower stabilization of ammonium ylides (for NMe_3_) relative to phosphonium ones can be used to explain this trend.^19^ In the latter case, another factor also affects the reactivity, as this ylide is computed to be less nucleophilic than phosphorous ylides (free energy barrier to addition is 13.1, 13.4, and 17.3 kcal mol^−1^ for X=PMe_3_, PPh_3_, and NMe_3_, respectively), thereby providing a rational explanation for the observed trend in reactivity in the presence of different nucleophilic catalysts (compare with the results in Table 1).

**Scheme 4:**
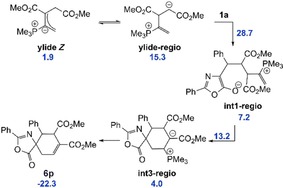
Proposed mechanism for the formation of **6 p**.

The formation of regioisomer **6 p** was also computationally investigated. On the basis of our results, the mechanism depicted in Scheme 4 mostly likely accounts for its formation. The mechanism involves the formation of **ylide‐regio** from **ylide** ***Z*** by a proton transfer. This ylide can then add to azlactone **1 a** to yield **int1‐regio**, which can undergo a cyclization to lead to **6 p** after a proton transfer followed by elimination of the phosphine. The rate‐determining step of the process is predicted to be the addition step, which has a free energy barrier of 28.7 kcal mol^−1^. Our calculations, thus, indicate that because of the lower stability of the **ylide‐regio** relative to **ylide** ***Z***, the formation of **6 p** is less favored than that of **5 p** (by 2.8 kcal mol^−1^), which is in good agreement with the experimental outcome.

## Conclusions

It was shown that α‐branched allenoates **4** can undergo formal [4+2] annulations with arylidene azlactones **1** under phosphine catalysis to access highly functionalized spirocyclohexenes **5** in a straightforward manner. This cyclization predominantly proceeds through the γ‐addition of the phosphine‐activated allenoates, and β′‐addition is clearly disfavored. The reaction requires the use of small sterically less‐hindered tertiary phosphines and does not proceed in the presence of tertiary amines or sterically hindered phosphines, which unfortunately made an enantioselective protocol not possible. Detailed computational studies support the proposed mechanism and provide a reasonable explanation for the strong influence of the catalyst as well as for the preference of the γ‐addition over a β′‐addition reaction.

## Experimental Section

**General reaction procedure**: A flame‐dried Schlenk pressure tube was charged with compound **1** (1 equiv) and molecular sieves (4 Å, 30 mg per mmol of **1**). Dry and degassed THF (ensuring a 0.01 m solution of **1**) and PBu_3_ (0.2 equiv) were added. Then, allenoate **4** (3 equiv) was dissolved in THF (0.6 m), and the solution was added to the suspension. The reaction mixture was stirred for 20 h at 60 °C. DCM and brine were added, and the phases were separated. The aqueous phase was extracted with DCM (3×), and the combined organic phases were dried with Na_2_SO_4_. The suspension was filtered, and the filtrate was evaporated to dryness. The crude product mixture was subjected to (flash) column chromatography (silica gel, heptanes/EtOAc) to afford compound **5** as a mixture of diastereomers. (With regard to the regioisomers, only **6 a** was isolated in a reasonable quantity.)

**Product 5 a**: Following the general procedure (0.25 mmol scale) above afforded **5 a** (71 % yield, 4.0:1 *dr*) as a white solid. Data of the major diastereomer: ^1^H NMR (700 MHz, CDCl_3_, 298 K): *δ*=7.70 (dd, *J*=8.2, 1.2 Hz, 2 H), 7.52 (m, 1 H), 7.47 (td, *J*=7.5, 1.2 Hz, 1 H), 7.35 (t, *J*=7.9 Hz, 2 H), 7.20–7.14 (m, 4 H), 7.10 (td, *J*=7.2, 1.2 Hz, 1 H), 4.34–4.29 (m, 1 H), 4.26–4.18 (m, 3 H), 3.95 (m, 1 H), 3.75 (s, 1 H), 3.21 (m, 1 H), 2.79 (td, *J*=19.8, 5.4 Hz, 1 H), 1.31–1.27 ppm (m, 6 H); ^13^C NMR (176 MHz, CDCl_3_, 298 K): *δ*=177.0, 170.3, 165.6, 160.2, 142.8, 137.3, 132.8, 128.8, 128.7, 128.4, 128.0, 127.9, 125.4, 125.0, 71.5, 61.6, 61.2, 47.7, 41.8, 28.4, 14.5, 14.3 ppm: HRMS (ESI): *m*/*z*: calcd for C_26_H_25_NO_6_: 448.1760 [*M*+H]^+^; found: 448.1761.

## Conflict of interest

The authors declare no conflict of interest.

## Supporting information

As a service to our authors and readers, this journal provides supporting information supplied by the authors. Such materials are peer reviewed and may be re‐organized for online delivery, but are not copy‐edited or typeset. Technical support issues arising from supporting information (other than missing files) should be addressed to the authors.

Supplementary
