# Supplementary material for: Syntheses of Highly Functionalized Spirocyclohexenes by Formal [4+2] Annulation of Arylidene Azlactones with Allenoates
Source: Asian J Org Chem. 2018 Jun 10;7(8):1620–5. doi: 10.1002/ajoc.201800275 (PMC6114148; doi:10.1002/ajoc.201800275)
Supplement: Supplementary file 1 — Supplementary [file AJOC-7-1620-s001.pdf]

ASIAN JOURNAL OF ORGANIC CHEMISTRY

Supporting Information

Syntheses of Highly Functionalized Spirocyclohexenes by Formal [4+2] Annulation of Arylidene Azlactones with Allenoates

Andreas Eitzinger,[a] Katharina Zielke,[a] Michael Widhalm,[b] Raphaël Robiette,*[c] and Mario Waser*[a]

ajoc_201800275_sm_miscellaneous_information.pdf

1. General Information:..2
1.1. General Methods..2
1.2. Single-Crystal Analysis 3
2. Syntheses 4
2.1 PBu_3-Catalyzed Formal (4+2)-Addition of Allenoates to Arylidene Azlactones...........4
2.2 Procedure for the Opening of the Azlactone Ring of Product 5a...............................10
2.3 Chiral Phosphines Tested for the Attempted Asymmetric (4+2)-Annulation..............11
3. Computational Methods 12
4. Copies of NMR Spectra...87

1. General Information:

1.1. General Methods

^{1}H- and ^{13}C-NMR spectra were recorded on a Bruker Avance III 300 MHz spectrometer with a broad band observe probe and a sample changer for 16 samples, on a Bruker Avance DRX 500 MHz spectrometer, and on a Bruker Avance III 700 MHz spectrometer with an Ascend magnet and TCI cryoprobe, which are property of the Austro-Czech NMR-Research Center "RERI-uasb". All NMR spectra were referenced on the solvent peak. High resolution mass spectra were obtained using an Agilent 6520 Q-TOF mass spectrometer with an ESI source and an Agilent G1607A coaxial sprayer or a Thermo Fisher Scientific LTQ Orbitrap XL with an Ion Max API Source. Analyses were made in the positive ionization mode if not otherwise stated. Purine (exact mass for $[M+H]^+$ = 121.050873) and 1,2,3,4,5,6-hexakis(2,2,3,3-tetrafluoropropoxy)-1,3,5,2,4,6-triazatriphosphinane (exact mass for $[M+H]^+$ = 922.009798) were used for internal mass calibration.

All chemicals were purchased from commercial suppliers and used without further purification unless otherwise stated. All reactions were carried out under an Argon atmosphere. THF was dried and degassed prior to use.

Arylidene azlactones **1** and allenoates **4** were prepared by reported procedures.[1]

[1] a) A. A. Beloglazkina, B. Wobith, E. S. Barskaia, N. A. Zefirov, A. G. Majouga, E. K. Belogazkina, N. V. Zyk, S. A. Kuznetsov, O. N. Zefirova, *Med. Chem. Res.*, **2016**, 25, 1239-1249; b) M. G. Sankar, M. Garcia-Castro, C. Golz, C. Strohmann, K. Kumar, *Angew. Chem. Int. Ed.*, **2016**, 55, 9709-9713.

1.2. Single-Crystal Analysis

Single-crystal structure analyses were carried out on a Bruker SmartX2S diffractometer operating with Mo-K$_\alpha$ radiation (λ= 0.71073 Å). Further crystallographic and refinement data can be found in Table S1. The structures were solved by direct methods (SHELXS-97)[2] and refined by full-matrix least squares on F^2 (SHELXL-97).[3] The H atoms were calculated geometrically, and a riding model was applied in the refinement process. Due to the absence of heavy atoms, the compound is a weak anomalous scatterer rendering the Flack parameter meaningless. Thus, the absolute structure could not be determined. CCDC 1813011 contain the supplementary crystallographic data for compound **5a**. These data can be obtained free of charge from The Cambridge Crystallographic Data Centre at www.ccdc.cam.ac.uk.

Table S1: Crystal Data and Data Collection and Structure Refinement Details for Compound **5a**.

**Crystal Data**	**5a**
Empirical formula	$C_{26}H_{25}NO_6$
Formula weight	447.47
Crystal size (mm)	0.54 × 0.43 × 0.21
Crystal system	orthorhombic
Space group	*Iba*2
a (Å)	19.536(2)
b (Å)	13.4782(15)
c (Å)	18.0060(17)
V (Å^3)	4741.2(9)
D_{calcd} (g cm^{-1})	1.254
Z	8
μ (mm^{-1})	0.09
T (K)	296
θ range (°)	1.8-23.3
No. of reflections measured	21458
No. of independent reflections	2837
Obs. Reflections [I > 2σ(I)]	292
Parameters refined/restraints	292/2
Absorption correction	multi-scan
T_{min}, T_{max}	0.44, 0.98
max/min σ_{fin} (e Å^{-3})	0.68/-0.27
R_1 [$I \geq 2\sigma(I)$]	0.060
wR_2	0.171
CCDC no.	1813011

[2] G. M. Sheldrick, SHELXS-97, Program for the Solution of Crystal Structures, Göttingen, Germany, 1997. See also: G. M. Sheldrick, *Acta Crystallographica*, **1990**, *A46*, 467-473.
[3] G. M. Sheldrick, SHELXL-97, Program for crystal structure refinement, Göttingen, Germany, 1997. See also: G. M. Sheldrick, *Acta Crystallographica*, **2008**, *A64*, 112-122.

2. Syntheses

2.1 PBu_3-Catalyzed Formal (4+2)-Addition of Allenoates to Arylidene Azlactones

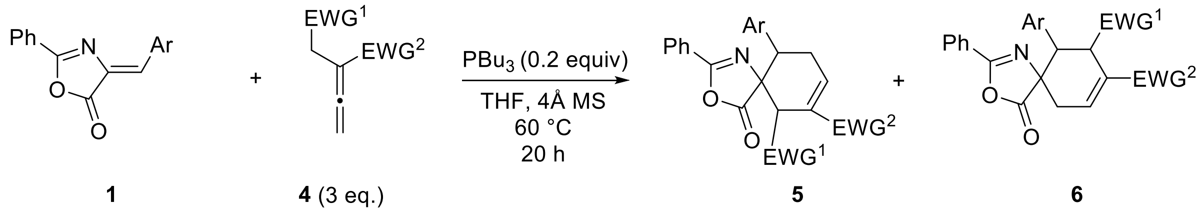

1a: Ar = Ph
1b: Ar = Ph
1c: Ar = Ph
1d: Ar = Ph
1e: Ar = 4-*t*-Bu-C_6H_4
1f: R^1 = 2-OMe-C_6H_4
1g: R^1 = 4-CN-C_6H_4
1h: R^1 = 4-NO_2-C_6H_4
1i: R^1 = 2-Br-C_6H_4
1j: R^1 = 4-Br-C_6H_4
1k: R^1 = 4-Cl-C_6H_4
1l: R^1 = 4-F-C_6H_4
1m: R^1 = β-Naphthyl
1n: R^1 = 2-Pyridyl
1o: R^1 = 2-Furyl

4a: EWG^1 = CO_2Et, EWG^2 = CO_2Et
4b: EWG^1 = CO_2Et, EWG^2 = CO_2tBu
4c: EWG^1 = CO_2Et, EWG^2 = CO_2Bn
4d: EWG^1 = CN, EWG^2 = CO_2Et
4e-4o: EWG^1 = CO_2Et, EWG^2 = CO_2Et

General Procedure:

A pressure Schlenk tube was flame dried in vacuo and charged with **1** (1 eq) and 4Å MS (30 mg per mmol of **1**; pulverized). THF (ensuring a 0.01 M solution of **1**; dry and degassed) and PBu_3 (0.2 eq) were added consecutively in an argon counterstream. Allenoates **4** (3 eq) were predissolved in THF (0.6 M) and added to the suspension. The reaction mixtures were then stirred for 20 h at 60 °C. DCM and brine were added and the phases were allowed to separate. The aqueous phase was extracted three times with DCM and the combined organic phases were dried over Na_2SO_4. The suspension was filtered and the filtrate was evaporated to dryness. The crude product mixtures were subjected to (flash) column chromatography (silica gel; heptanes/EtOAc) to afford compounds **5**. For some products, a subsequent purification by preparative HPLC was conducted. NMR data for the major diastereomers are given below. The regioisomer **6** could only be isolated in a reasonable quantity and quality for the parent system **6a** (see analytical data below).

Diethyl 4-oxo-2,10-diphenyl-3-oxa-1-azaspiro[4.5]deca-1,7-diene-6,7-dicarboxylate (5a):

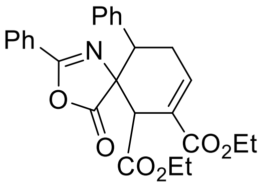

5a

Prepared according to the general procedure and isolated as white crystals in 71% yield and with a d.r. of 4.0:1 (0.25 mmol scale). m.p.: 122 – 123 °C. ^{1}H NMR (700 MHz, δ, $CDCl_3$, 298 K): 7.70 (dd, J = 8.2, 1.2 Hz, 2H), 7.52 (m, 1H), 7.47 (td, J = 7.5, 1.2 Hz, 1H), 7.35 (t, J = 7.9 Hz, 2H), 7.20 – 7.14 (m, 4H), 7.10 (td, J = 7.2, 1.2 Hz, 1H), 4.34 – 4.29 (m, 1H), 4.26 – 4.18 (m, 3H), 3.95 (m, 1H), 3.75 (s, 1H), 3.21 (m, 1H), 2.79 (td, J = 19.8, 5.4 Hz, 1H), 1.31 – 1.27 (m, 6H) ppm. ^{13}C (176 MHz, δ, $CDCl_3$, 298 K): 177.0, 170.3, 165.6, 160.2, 142.8, 137.3, 132.8, 128.8, 128.7, 128.4, 128.0, 127.9, 125.4, 125.0, 71.5, 61.6, 61.2, 47.7, 41.8, 28.4, 14.5, 14.3 ppm. HRMS (ESI): m/z calcd for $C_{26}H_{25}NO_6$: 448.1760 $[M+H]^+$; found: 448.1761.

Diethyl 4-oxo-2,6-diphenyl-3-oxa-1-azaspiro[4.5]deca-1,8-diene-7,8-dicarboxylate (6a):

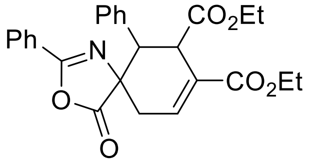

6a

Prepared according to general procedure and isolated as a brownish oil with 24% yield (0.1 mmol scale). R_f = 0.45 (heptane/ethyl acetate:3/1). ^{1}H NMR (300 MHz, δ, $CDCl_3$, 298 K): 7.77 (dd, J = 8.4, 1.4 Hz, 2H), 7.47 (td, J = 7.4, 1.3 Hz, 1H), 7.34 (t, J = 7.6 Hz, 2H), 7.25 (td, J = 6.6, 1.8 Hz, 1H), 7.12 – 7.06 (m, 5H), 4.32 – 4.29 (m, 1H), 4.18 (q, J = 7.2 Hz, 2H), 3.87 (q, J = 7.2 Hz, 2H), 3.27 (m, 1H), 3.14 – 3.01 (m, 1H), 2.49 (m, 1H), 1.22 (t, J = 7.2 Hz, 3H), 0.90 (t, J = 7.2 Hz, 3H) ppm. ^{13}C NMR (176 MHz, δ, $CDCl_3$, 298 K): 178.3, 168.9, 166.2, 162.3, 139.4, 136.3, 133.0, 128.9, 128.7, 128.3, 128.2, 128.0, 126.7, 125.3, 73.9, 61.5, 60.8, 50.0, 47.1, 28.5, 14.2, 13.8 ppm. HRMS (ESI): m/z calcd for $C_{26}H_{25}NO_6$: 448.1760 $[M+H]^+$; found: 448.1765.

7-(tert-Butyl) 6-ethyl 4-oxo-2,10-diphenyl-3-oxa-1-azaspiro[4.5]deca-1,7-diene-6,7-dicarboxylate (5b):

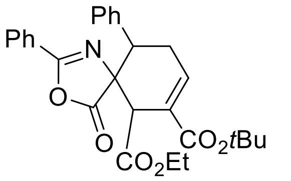

5b

Prepared according to general procedure and isolated as a brownish oil in 52% yield and with a d.r. of 4.5:1 (0.1 mmol scale). R_f = 0.55 (heptane/ethyl acetate:3/1). ^{1}H NMR (300 MHz, δ, $CDCl_3$, 298 K): 7.64 (d, J = 7.8 Hz, 2H), 7.50 – 7.43 (m, 1H), 7.38 – 7.34 (m, 2H), 7.28 (t, J = 7.8 Hz, 2H), 7.11 – 7.08 (m, 4H), 4.17 – 4.02 (m, 2H), 3.88 – 3.82 (m, 1H), 3.63 (s, 1H), 3.19 – 3.07 (m, 1H), 2.68 (td, J = 19.6, 5.4 Hz, 1H), 1.41 (s, 9H), 1.21 (t, J = 7.2 Hz, 3H) ppm. ^{13}C NMR (75 MHz, δ, $CDCl_3$, 298 K): 178.4, 176.9, 170.2, 164.5, 159.9, 141.7, 137.2, 128.7, 128.5, 128.2, 127.7, 126.1, 125.3, 81.3, 71.4, 61.3, 47.7, 41.7, 28.1, 27.8, 14.3, 14.1, 14.0, 13.9 ppm. HRMS (ESI): m/z calcd for $C_{28}H_{29}NO_6$: 476.2073 $[M+H]^+$; found: 476.2078.

7-Benzyl 6-ethyl 4-oxo-2,10-diphenyl-3-oxa-1-azaspiro[4.5]deca-1,7-diene-6,7-dicarboxylate (5c):

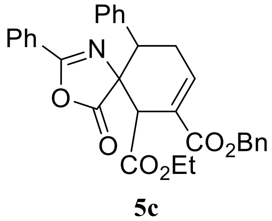

5c

Prepared according to general procedure and isolated as a white solid in 65% yield and a d.r. of 3.8:1 (0.1 mmol scale). m.p.: 117 – 118 °C. ^{1}H NMR (300 MHz, δ, $CDCl_3$, 298 K): 7.63 (d, *J* = 7.8 Hz, 2H), 7.52 – 7.49 (m, 1H), 7.41 – 7.37 (m, 1H), 7.30 – 7.25 (m, 7H), 7.10 – 7.08 (m, 4H), 7.06 – 7.02 (m, 1H), 5.21 – 5.02 (m, 2H), 4.23 – 3.99 (m, 2H), 3.91 – 3.85 (m, 1H), 3.70 (s, 1H), 3.21 – 3.09 (m, 1H), 2.72 (td, *J* = 19.7 Hz & 5.4 Hz, 1H), 1.13 (t, *J* = 7.2 Hz, 3H) ppm. ^{13}C NMR (75 MHz, δ, $CDCl_3$, 298 K): 176.8, 170.0, 165.2,

160.0, 143.4, 137.0, 135.6, 132.7, 128.6, 128.5, 128.3, 128.2, 127.7, 125.2, 124.6, 71.3, 66.8, 61.4, 47.5, 41.6, 28.3, 14.2 ppm. HRMS (ESI): m/z calcd for $C_{31}H_{27}NO_6$: 510.1917 $[M+H]^+$; found: 510.1920.

Ethyl 6-cyano-4-oxo-2,10-diphenyl-3-oxa-1-azaspiro[4.5]deca-1,7-diene-7-carboxylate

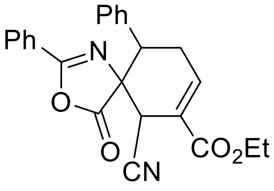

5d

(5d): Prepared according to the general procedure and isolated as a white solid in 58% yield and with a d.r. of 2.8:1 (0.25 mmol scale). m.p.: 114 – 115 °C. ^{1}H NMR (700 MHz, δ, $CDCl_3$, 298 K): 7.79 (d, *J* = 7.7 Hz, 2H), 7.52 – 7.50 (m, 2H), 7.37 (t, *J* = 7.7 Hz, 2H), 7.24 – 7.19 (m, 4H), 7.16 – 7.14 (m, 1H), 4.34 – 4.30 (m, 2H), 4.00 (s, 1H), 3.81 – 3.79 (m, 1H), 3.27 – 3.23 (m, 1H), 2.87 (td, *J* = 20.3, 5.3 Hz, 1H), 1.36 (t, *J* = 7.2 Hz, 3H) ppm. ^{13}C NMR (176 MHz, δ, $CDCl_3$, 298 K): 175.2, 164.0, 161.4, 143.8, 133.6, 128.8, 128.7, 128.5, 128.3, 124.6, 122.3, 116.6, 70.2, 61.9, 43.5, 35.0, 29.0, 14.3 ppm. HRMS (ESI): m/z calcd for $C_{24}H_{20}N_2O_4$: 401.1501 $[M+H]^+$; found: 401.1495.

Diethyl 10-(4-(tert-butyl)phenyl)-4-oxo-2-phenyl-3-oxa-1-azaspiro[4.5]deca-1,7-diene-6,7-dicarboxylate

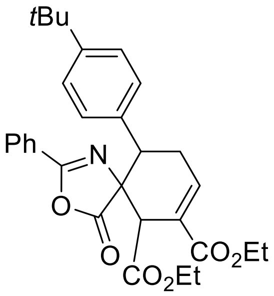

5e

(5e): Prepared according to the general procedure and isolated as a white solid in 63% yield and with a d.r. of 4.0:1 (0.1 mmol scale). m.p.: 119 – 120 °C. ^{1}H NMR (300 MHz, δ, $CDCl_3$, 298 K): 7.60 (m, 2H), 7.46 – 7.43 (m, 1H), 7.42 – 7.36 (m, 2H), 7.27 (t, *J* = 7.8 Hz, 2H), 7.09 – 6.99 (m, 3H), 4.29 – 4.08 (m, 4H), 3.85 – 3.80 (m, 1H), 3.69 (s, 1H), 3.19 – 3.07 (m, 1H), 2.72 (td, *J* = 20.0, 5.5 Hz, 1H), 1.25 – 1.20 (m, 6H), 1.04 (s, 9H) ppm. ^{13}C NMR (75 MHz, δ, $CDCl_3$, 298 K): 176.9, 170.2, 165.5, 160.0, 150.7, 142.7, 133.7, 132.5, 128.7, 128.4, 128.3, 127.7, 125.0, 71.5, 61.3, 61.0, 47.3, 41.3, 34.3, 31.2, 31.1, 28.0, 14.3, 14.1 ppm. HRMS (ESI): m/z calcd for $C_{30}H_{33}NO_6$: 504.2386 $[M+H]^+$; found: 504.2381.

Diethyl 10-(2-methoxyphenyl)-4-oxo-2-phenyl-3-oxa-1-azaspiro[4.5]deca-1,7-diene-6,7-dicarboxylate

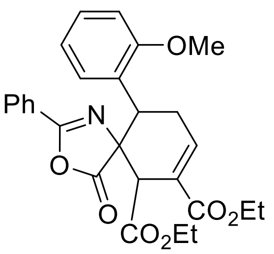

5f

(5f): Prepared according to the general procedure and isolated as a colourless oil in 40% yield with a d.r. of 2.0:1 together with 15% of the regioisomer **6f** (0.25 mmol scale). R_f = 0.42 (heptane/ethyl acetate:3/1). ^{1}H NMR (700 MHz, δ, $CDCl_3$, 298 K): 7.80 (d, *J* = 7.5 Hz, 2H), 7.50 – 4.48 (m, 2H), 7.37 (t, *J* = 7.7 Hz, 2H), 7.16 – 7.15 (m, 1H), 7.13 – 7.11 (m, 1H), 6.83 (t, *J* = 7.5 Hz, 1H), 6.73 (d, *J* = 8.0 Hz, 1H), 4.33 (bs, 1H), 4.26 – 4.19 (m, 3H), 4.17 – 4.12 (m, 1H), 3.76 (s, 1H), 3.74 (s, 3H), 3.05 – 3.00 (m, br, 1H), 2.81 – 2.77 (m, br, 1H), 1.28 (t, *J* = 7.0 Hz, 3H), 1.22 (t, *J* = 7.2 Hz, 3H) ppm. ^{13}C NMR (176 MHz, δ, $CDCl_3$, 298 K): 165.9, 160.4, 157.9, 140.2, 132.7, 128.9, 128.7, 128.2, 127.9, 126.4, 125.9, 120.9, 111.0 61.4, 61.0, 28.6, 14.3 ppm. HRMS (ESI): m/z calcd for $C_{27}H_{27}NO_7$: 478.1866 $[M+H]^+$; found: 478.1862.

Diethyl 10-(4-cyanophenyl)-4-oxo-2-phenyl-3-oxa-1-azaspiro[4.5]deca-1,7-diene-6,7-dicarboxylate (5g): Prepared according to the general procedure and isolated as a white solid in 74% yield and with a d.r. of 3.6:1.5:1 (0.25 mmol scale). m.p.: 123 – 124 °C. ^{1}H NMR (700 MHz, δ, $CDCl_3$, 298 K): 7.71 (d, J = 7.4 Hz, 1H), 7.53 (t, *J* = 7.4 Hz, 1H), 7.50 – 7.48 (m, 1H), 7.47 – 7.45 (m, 2H), 7.40 – 7.38 (m, 2H), 7.32 – 7. 31 (m, 2H), 4.35 – 4.29 (m, 1H), 4.26 – 4.18 (m, 4H), 4.07 – 4.05 (m, 1H), 3.75 (s, 1H), 3.22 – 3.17 (m, 1H), 2.80 – 2.76 (m, 1H), 1.31 – 1.29 (m, 6H) ppm. ^{13}C NMR (176 MHz, δ, $CDCl_3$, 298 K): 176.3, 175.8, 170.1, 165.3, 160.6, 143.0, 141.8, 133.3, 132.2, 129.7, 128.9, 127.9, 118.6, 111.9, 71.0, 61.7, 61.3, 53.6, 47.6, 41.8, 27.9, 14.4, 14.3 ppm. HRMS (ESI): m/z calcd for $C_{27}H_{24}N_2O_6$: 473.1713 $[M+H]^+$; found: 473.1707.

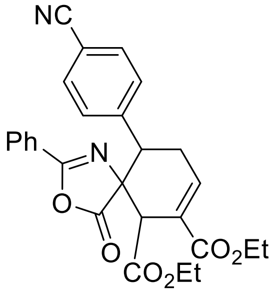

5g

Diethyl 10-(4-nitrophenyl)-4-oxo-2-phenyl-3-oxa-1-azaspiro[4.5]deca-1,7-diene-6,7-dicarboxylate (5h): Prepared according to the general procedure and isolated as a white solid in 62% yield and with a d.r. of 3.2:1 (0.25 mmol scale). m.p.: 135 – 136 °C. ^{1}H NMR (700 MHz, δ, $CDCl_3$, 298 K): 8.04 – 8.03 (m, 2H), 7.72 – 7.71 (m, 1H), 7.52 – 7.48 (m, 2H), 7.39 – 7.37 (m, 4H), 7.26 – 7.25 (m, 1H), 4.36 – 4.30 (m, 1H), 4.27 – 4.18 (m, 3H), 4.15 – 4.13 (m, 1H), 3.76 (s, 1H), 3.25 – 3.21 (m, 1H), 2.82 – 2.78 (m, 1H), 1.31 – 1.28 (m, 6H) ppm. ^{13}C NMR (176 MHz, δ, $CDCl_3$, 298 K): 176.25, 170.1, 165.4, 160.7, 147.6, 145.2, 141.7, 133.4, 129.8, 128.9, 127.9, 123.6, 71.0, 61.7, 61.3, 47.6, 41.5, 28.1, 14.4, 14.3 ppm. HRMS (ESI): m/z calcd for $C_{26}H_{24}N_2O_8$: 493.1611 $[M+H]^+$; found: 493.1606.

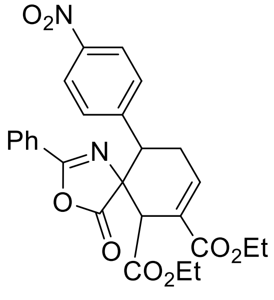

5h

Diethyl 10-(2-bromophenyl)-4-oxo-2-phenyl-3-oxa-1-azaspiro[4.5]deca-1,7-diene-6,7-dicarboxylate (5i): Prepared according to general procedure and isolated as a light yellow oil in 52% yield and a d.r. of 10:1.7:1 (0.1 mmol scale). R_f = 0.37 (heptane/ethyl acetate:3/1). ^{1}H NMR (300 MHz, δ, $CDCl_3$, 298 K): 7.69 (d, *J* = 7.8 Hz, 2H), 7.44 – 7.36 (m, 3H, Ar-H), 7.29 (t, *J* = 7.6 Hz, 2H), 7.15 – 7.07 (m, 2H), 6.95 – 6.85 (m, 1H), 4.67 – 4.64 (m, 1H), 4.18 – 4.14 (m, 2H), 4.09 – 4.05 (m, 2H), 3.69 (s, 1H), 3.06 – 2.96 (m, 1H), 2.71 (td, *J* = 19.7, 5.3 Hz, 1H), 1.23 (t, *J* = 7.2 Hz, 3H), 0.96 (t, *J* = 7.2 Hz, 3H) ppm. ^{13}C NMR (75 MHz, δ, $CDCl_3$, 298 K): 169.5, 142.1, 133.3, 132.7, 129.1, 128.6, 127.9, 127.0, 125.3, 71.8, 61.6, 61.1, 47.8, 40.2, 28.6, 14.3, 14.1 ppm. HRMS (ESI): m/z calcd for $C_{26}H_{24}NO_6Br$: 526.0865 $[M+H]^+$; found: 526.0862.

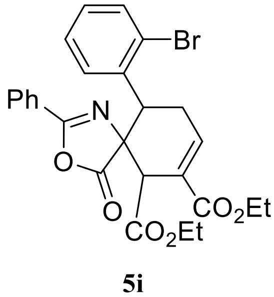

5i

Diethyl 10-(4-bromophenyl)-4-oxo-2-phenyl-3-oxa-1-azaspiro[4.5]deca-1,7-diene-6,7-dicarboxylate (5j): Prepared according to the general procedure and isolated as a light yellow oil in 66% yield and with a d.r. of 4.0:1 (0.1 mmol scale). R_f = 0.40 (heptane/ethyl acetate:3/1). ^{1}H NMR (300 MHz, δ, $CDCl_3$, 298 K): 7.67 (d, J = 8.0 Hz, 2H), 7.45 – 7.39 (m, 2H), 7.32 (t, J = 7.6 Hz, 2H), 7.24 – 7.20 (m, 2H), 7.00 (d, J = 8.0 Hz, 2H), 4.18 – 4.12 (m, 4H), 3.90 – 3.84 (m, 1H), 3.67 (s, 1H), 3.15 – 3.03 (m, 1H), 2.69 (td, J = 19.6, 5.5 Hz, 1H), 1.22 (t, J = 7.2 Hz, 3H), 1.21 (t, J = 7.2 Hz, 3H) ppm. ^{13}C NMR (75 MHz, δ, $CDCl_3$, 298 K): 176.5, 170.1, 165.3, 160.3, 142.2, 136.3, 132.9, 131.5, 131.4, 130.4, 128.7, 127.8, 125.0, 124.9, 121.8, 71.0, 61.5, 61.1, 47.5, 41.0, 28.2, 14.3, 14.1 ppm. HRMS (ESI): m/z calcd for $C_{26}H_{24}NO_6Br$: 526.0865 $[M+H]^+$; found: 526.0861.

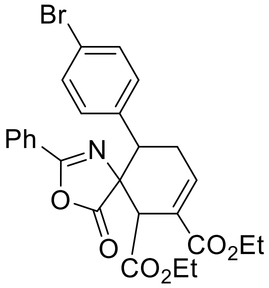

5j

Diethyl 10-(4-chlorophenyl)-4-oxo-2-phenyl-3-oxa-1-azaspiro[4.5]deca-1,7-diene-6,7-dicarboxylate (5k): Prepared according to the general procedure and isolated as a white solid in 51% yield and with a d.r. of 3.4:1 (0.25 mmol scale). m.p.: 110 – 111 °C. ^{1}H NMR (700 MHz, δ, $CDCl_3$, 298 K): 7.74 – 7.73 (m, 2H), 7.52 – 7.49 (m, 2H), 7.39 (t, J = 7.8 Hz, 2H), 7.15 – 7.12 (m, 4H), 4.34 – 4.30 (m, 1H), 4.26 – 4.17 (m, 3H), 3.96 – 3.94 (m, 1H), 3.74 (m, 1H), 3.18 – 3.14 (m, 1H), 2.76 (td, J = 19.8, 5.4 Hz, 1H), 1.30 – 1.27 (m, 6H) ppm. ^{13}C NMR (176 MHz, δ, $CDCl_3$, 298 K): 176.7, 170.2, 165.5, 160.4, 142.3, 135.9, 133.7, 133.0, 130.1, 128.8, 128.6, 127.9, 125.2, 125.0, 71.3, 61.6, 61.2, 47.6, 41.0, 28.3, 14.4, 14.3 ppm. HRMS (ESI): m/z calcd for $C_{26}H_{24}NO_6Cl$: 482.1370 $[M+H]^+$; found: 482.1385.

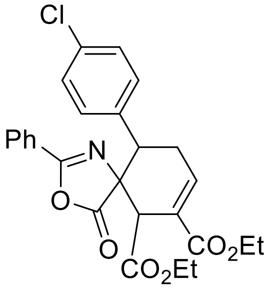

5k

Diethyl 10-(4-fluorophenyl)-4-oxo-2-phenyl-3-oxa-1-azaspiro[4.5]deca-1,7-diene-6,7-dicarboxylate (5l): Prepared according to the general procedure and isolated as a white solid in 58% yield and with a d.r. of 3.4:1 (0.25 mmol scale). m.p.: 139 – 140 °C. ^{1}H NMR (700 MHz, δ, $CDCl_3$, 298 K): 7.72 (d, J = 7.8 Hz, 2H), 7.51 – 7.49 (m, 2H), 7.38 (t, J = 7.8 Hz, 2H), 7.17 – 7.15 (m, 2H), 6.85 (m, 2H), 4.34 – 4.30 (m, 1H), 4.26 – 4.17 (m, 3H), 3.97 – 3.94 (m, 1H), 3.74 (s, 1H), 3.19 – 3.15 (m, 1H), 2.77 (td, J = 19.8, 5.4 Hz, 1H), 1.31 – 1.27 (m, 6H) ppm. ^{13}C NMR (176 MHz, δ, $CDCl_3$, 298 K): 176.8, 170.2, 165.5, 160.3, 142.5, 133.0, 130.4, 128.8, 127.9, 125.2, 125.0, 115.4, 115.2, 71.4, 61.5, 61.2, 47.6, 40.9, 28.4, 14.4, 14.3 ppm. ^{19}F NMR (282 MHz, δ, $CDCl_3$, 298 K): -114.4 ppm. HRMS (ESI): m/z calcd for $C_{26}H_{24}NO_6F$: 466.1666 $[M+H]^+$; found: 466.1646.

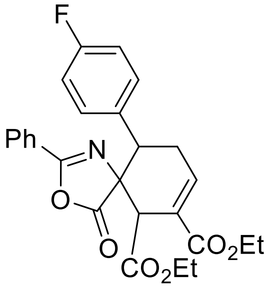

5l

Diethyl 10-(naphthalen-2-yl)-4-oxo-2-phenyl-3-oxa-1-azaspiro[4.5]deca-1,7-diene-6,7-dicarboxylate (5m): Prepared according to the general procedure and isolated as a white solid in 62% yield and with a d.r. of 3.9:1 (0.25 mmol scale). m.p.: 116 – 117 °C. ^{1}H NMR (700 MHz, δ, $CDCl_3$, 298 K): 7.74 (d, J = 7.8 Hz, 1H), 7.68 – 7.66 (m, 4H), 7.63 – 7.62 (m, 1H), 7.56 – 7.55 (m, 1H), 7.42 – 7.36 (m, 3H), 7.32 – 7.28 (m, 3H), 4.37 – 4.33 (m, 1H), 4.28 – 4.19 (m, 3H), 4.16 – 4.13 (m, 1H), 3.78 (s, 1H), 3.38 – 3.34 (m, 1H), 2.84 (td, J = 19.8, 5.4 Hz, 1H), 1.32 – 1.29 (m, 6H) ppm. ^{13}C NMR (176 MHz, δ, $CDCl_3$, 298 K): 177.0, 170.3, 165.6, 160.3, 142.8, 135.0, 133.3, 132.9, 132.8, 128.6, 128.0, 127.9, 127.6, 127.4, 127.2, 126.2, 126.1, 125.3, 125.0, 71.6, 61.5, 61.2, 47.9, 41.7, 28.7, 14.4, 14.3 ppm. HRMS (ESI): m/z calcd for $C_{30}H_{27}NO_6$: 498.1917 $[M+H]^+$; found: 498.1901.

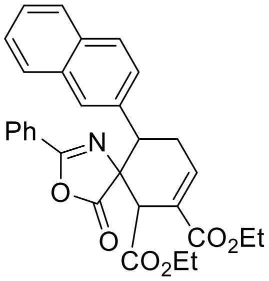

5m

Diethyl 4-oxo-2-phenyl-10-(pyridin-2-yl)-3-oxa-1-azaspiro[4.5]deca-1,7-diene-6,7-dicarboxylate (5n): Prepared according to the general procedure and isolated as a white solid in 48% yield and with a d.r. of 3.0:1 (0.25 mmol scale). m.p.: 70 – 71 °C. ^{1}H NMR (700 MHz, δ, $CDCl_3$, 298 K): 8.46 – 8.44 (m, 1H), 8.37 – 8.36 (m, 1H), 7.73 (d, J = 7.6 Hz, 2H), 7.52 – 7.48 (m, 2H), 7.46 – 7.44 (m, 1H), 7.37 (t, J = 7.7 Hz, 2H), 7.12 – 7.10 (m, 1H), 4.36 – 4.29 (m, 1H), 4.27 – 4.18 (m, 3H), 4.03 – 4.00 (m, 1H), 3.77 (s, 1H), 3.23 – 3.19 (m, 1H), 2.79 (td, J = 19.7, 5.3 Hz, 1H), 1.31 – 1.28 (m, 6H) ppm. ^{13}C NMR (176 MHz, δ, $CDCl_3$, 298 K): 176.4, 170.1, 165.4, 160.6, 151.1, 149.4, 142.0, 135.3, 133.1, 128.8, 128.0, 125.2, 124.9, 123.3, 71.1, 61.7, 61.3, 47.5, 39.3, 27.8, 14.4, 14.3 ppm. HRMS (ESI): m/z calcd for $C_{25}H_{24}N_2O_6$: 449.1713 $[M+H]^+$; found: 449.1701.

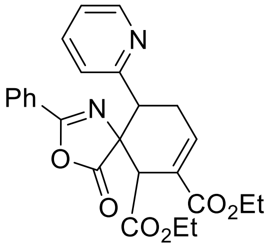

5n

Diethyl 10-(furan-2-yl)-4-oxo-2-phenyl-3-oxa-1-azaspiro[4.5]deca-1,7-diene-6,7-dicarboxylate (5o): Prepared according to the general procedure and isolated as a white solid in 54% yield and with a d.r. of 2.0:1 (0.25 mmol scale). m.p.: 95 – 96 °C. ^{1}H NMR (700 MHz, δ, $CDCl_3$, 298 K): 7.86 – 7.85 (m, 2H), 7.55 – 7.53 (m, 1H), 7.44 – 7.41 (m, 3H), 7.19 (m, 1H), 6.16 – 6.13 (m, 2H), 4.32 – 4.28 (m, 1H), 4.25 – 4.17 (m, 3H), 4.06 – 4.04 (m, 1H), 3.78 (s, 1H), 3.13 – 3.09 (m, 1H), 2.81 (td, J = 19.8, 5.4 Hz, 1H), 1.30 – 1.26 (m, 6H) ppm. ^{13}C NMR (176 MHz, δ, $CDCl_3$, 298 K): 176.3, 170.2, 165.5, 161.2, 151.7, 142.3, 141.8, 133.0, 128.8, 128.1, 125.5, 125.1, 110.4, 108.3, 70.2, 61.6, 61.2, 47.3, 36.2, 28.0, 14.4, 14.3 ppm. HRMS (ESI): m/z calcd for $C_{25}H_{24}N_2O_6$: 438.1553 $[M+H]^+$; found: 438.1546.

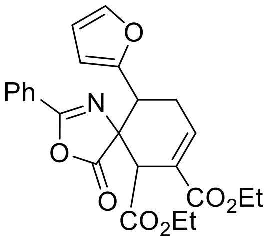

5o

2.2 Procedure for the Opening of the Azlactone Ring of Product 5a

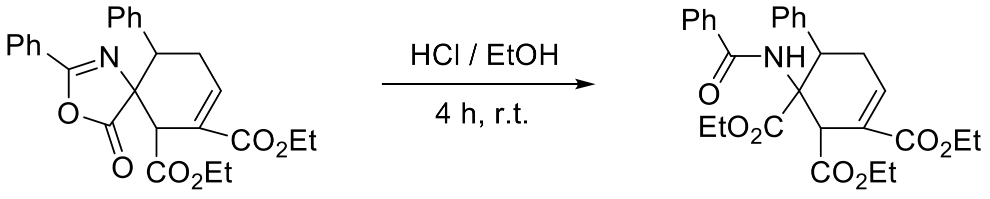

5a **7a**

70%

5a (1 mmol) was stirred in 2 mL of an ethanolic solution of HCl (1.25 M) for 4 hours at room temperature. The reaction mixture was concentrated *in vacuo* and the product **7a** was isolated by column chromatography (silica gel; heptanes/EtOAc).

Triethyl 2-benzamido-1,2,3,6-tetrahydro-[1,1'-biphenyl]-2,3,4-tricarboxylate (7a):

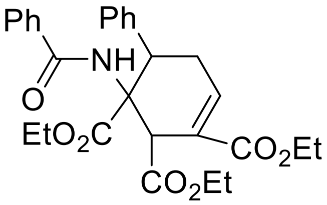

7a

Prepared according to the procedure described above and isolated as a colourless oil in 70% yield. R_f = 0.30 (heptane/ethyl acetate:3/1). ^{1}H NMR (300 MHz, δ, $CDCl_3$, 298 K): 7.56 (d, *J* = 7.7 Hz, 2H), 7.48 (d, *J* = 7.2 Hz ,1H), 7.41 – 7.28 (m, 8H), 6.63 (s, 1H), 4.88 (s, 1H), 4.32 – 3.96 (m, 7H), 2.83 – 2.65 (m, 2H), 1.31 (t, *J* = 7.1 Hz, 3H), 1.13 (t, *J* = 7.2 Hz, 3H), 1.05 (t, *J* = 7.1 Hz, 3H) ppm. ^{13}C NMR (75 MHz, δ, $CDCl_3$, 298 K): 172.5, 169.1, 167.1, 165.7, 140.1, 137.4, 133.8, 131.8, 129.6, 128.6, 128.4, 126.8, 61.6, 61.4, 61.2, 60.8, 47.9, 41.9, 29.7, 14.2, 13.9 ppm. MS (ESI): m/z calcd for $C_{28}H_{32}NO_7$: 494.2 $[M+H]^+$; found: 494.3.

2.3 Chiral Phosphines Tested for the Attempted Asymmetric (4+2)-Annulation

The chiral phosphines **P2** – **P7** were tested according to the general procedure but in neither case any product formation could be observed.

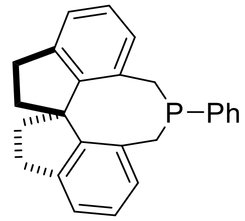

P2

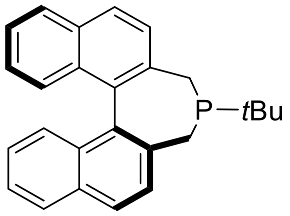

P3

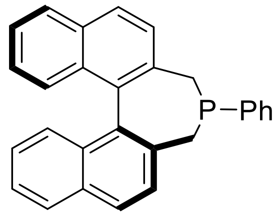

P4

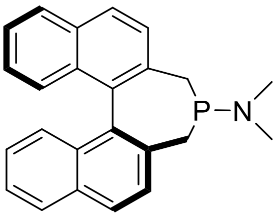

P5

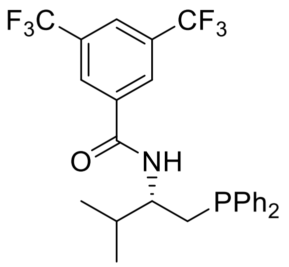

P6

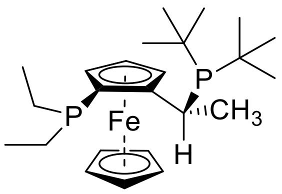

P7

3. Computational Methods

Calculations were performed using the Jaguar 8.5 pseudospectral program package.[4] Geometry optimizations and frequency calculations were performed at the B3LYP-D3/6-31+G(d) level of theory including an implicit description of tetrahydrofuran solvent using the Poisson–Boltzmann polarizable continuum method as incorporated in Jaguar.

Gas phase electronic energies were computed by single point calculations at the M06-2X-D3/6-311+G(d,p) level of theory.

The stationary points were characterized by full calculation of vibrational frequencies. Gibbs energies at 298.15 K were computed on the basis of the rigid rotor/harmonic oscillator approach to statistical mechanics. In Jaguar, Gibbs energies refer to an ideal gas in standard conditions, i.e. a pressure of 1 atmosphere at 298.15 K. For solution reactions, the standard condition is instead 1 mol/L. Accordingly, the gibbs energy value computed in Jaguar was corrected by a concentration term, equal to RT ln (V_mol_gas_1atm / V_mol_1M), i.e. 1.89 kcal/mol at 298.15 K.

For the large reaction systems there are usually several local minima or saddle points corresponding to each intermediate or transition state. This is due to the possibility of multiple conformations of substituents. We have made a systematic attempt to locate all possible local minima and saddle points, with the data presented referring to the lowest energy form unless mentioned otherwise. All species have been fully geometry optimized, and the Cartesian coordinates are supplied in Section 3.4.

In order to reduce calculations time, we have used model substrates which differ slightly from reagents and catalysts used in our experiments (methyl esters instead of ethyl esters on the allene and PMe_3 instead of PBu_3 as a catalyst). This simplification should have no influence on obtained results and their analysis. Indeed, experiments have showed that the nature of EWGs on the allene has no significant impact on the reaction (see Scheme 2) and the quasi-identical results obtained with PBu_3 and PEt_3 (see Table 1, entries 10-11) show that variations of the chain length of alkyl groups on the phosphine have no effect on the outcome of the reaction.

3.1 Detailed data for the postulated mechanism

3.1.1. Ylide formation

Ylide formation is slightly endergonic (by 1.9 kcal/mol) and involves a significant free energy barrier (25.9 kcal/mol). Two stereoisomers can be formed, **ylide-*E*** and **ylide-*Z*.** These two isomers are in equilibrium through a low lying transition state which should lead to exclusive formation of the more stable **ylide-*Z***.

[4] Jaguar 8.5; Schrodinger, Inc.: New York, NY, 2014.

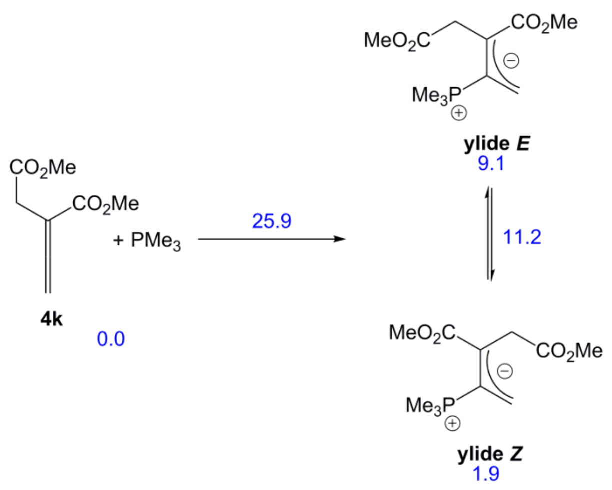

3.1.2. Addition

Addition of ylides onto the 5(4*H*)-oxazolone **1a** is predicted to involves a low free energy barrier (at least for **ylide *Z***). Calculations indicate that **ylide-*Z*** is not only the more stable stereoisomer but also the more reactive one toward addition onto **1a**. Exclusive formation of **int1-*Z*** is thus predicted.

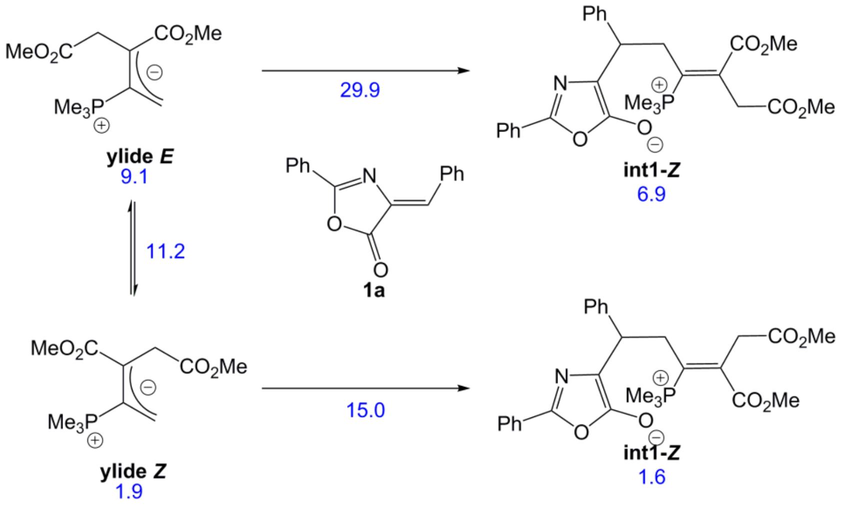

3.1.3. Double bond migration

Migration of the double bond can occur via a double intramolecular proton transfer involving the oxygen atom of the azlactone moiety (see section 3.2 for other potential mechanisms). This mechanism involves two steps: intramolecular deprotonation, to form **int-OH**, and then re-protonation. The two transition states along this pathway have a similar free energy in the case of *ZZ* isomer; the first proton transfer step may thus well be partially reversible. For the *EZ* isomer, the first proton transfer is predicted to be reversible since the transition state for the second proton transfer lies systematically higher than the one for the first proton transfer.

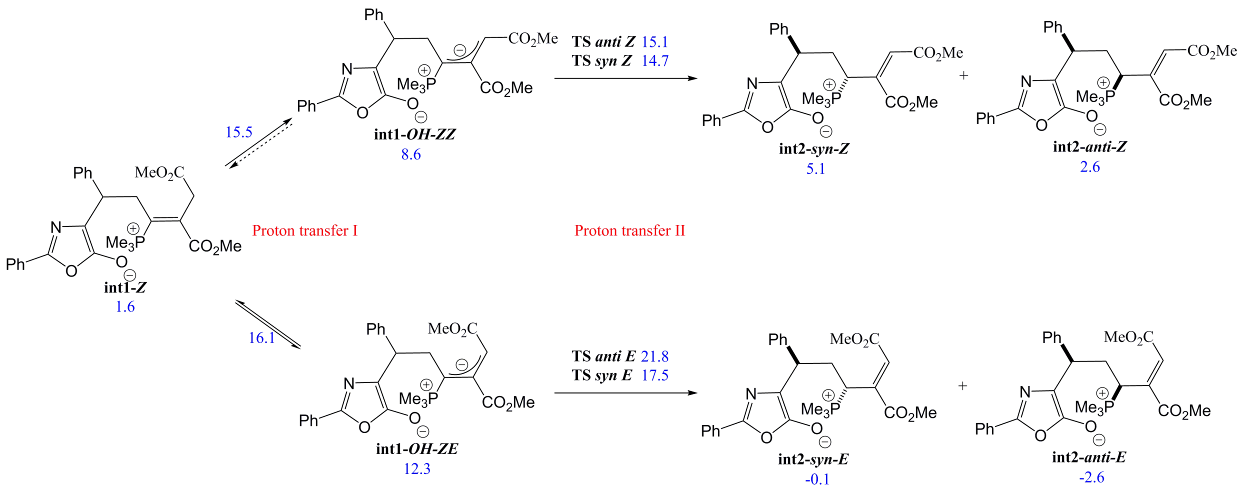

Four stereoisomers of **int2** can potentially be formed. Overall, a low kinetic stereoselectivity in favor of **int2-*syn-Z*** over **int2-*anti-Z*** is predicted; our results indicating that formation of *E* isomers should not be observed.

3.1.4. Cyclization

Our calculations suggest that cyclization does not occur in one step but instead via a two steps cyclization-elimination process (Table S2). In some cases, the intermediate formed does not allow direct elimination, due to stereoelectronic reasons, and needs to undergo a conformational equilibrium (chair ↔ boat) to yield the reactive intermediate (**int3**) which can undergo elimination. However, this equilibrium is fast and has no impact on the kinetic and selectivity of the overall process; cyclization being the rate-determining step of the cyclization/elimination process.
The overall process is, in all cases, exergonic.

Table S2. Cyclization, potential conformational equilibrium and elimination steps[a]

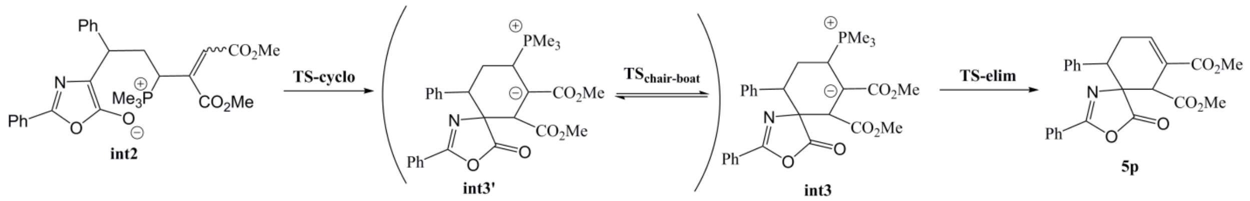

		TS-cyclo	int3'	$TS_{chair-boat}$	int3	TS-elim	5p
anti E	*cis-cis*	5.4	-6.4b	0.0	-5.6c	-0.1	-27.5
	cis-trans	8.2			-8.2c	-3.2	-23.6
	trans-cis	9.7			-10.3c	-4.9	-26.5
	trans-trans	7.9	-4.0b	2.1	-6.5c	-0.2	-20.9
anti Z	*cis-cis*	14.0			-5.6c	-0.1	-27.5
	cis-trans	11.3	-2.4b	5.2	-8.2c	-3.2	-23.6
	trans-cis	12.1	0.4b	4.0	-10.3c	-4.9	-26.5
	trans-trans	12.3			-6.5c	-0.2	-20.9

syn E	*cis-cis*	6.3			-8.4b	-1.9	-27.5
	cis-trans	9.8	4.0c	nd	-2.6b	0.9	-23.6
	trans-cis	9.3	0.5c	nd	-6.1b	1.6	-26.5
	trans-trans	9.2			-6.6b	0.7	-20.9
syn Z	*cis-cis*	15.6	-6.1c	nd	-8.4b	-1.9	-27.5
	cis-trans	15.7			-2.6b	0.9	-23.6
	trans-cis	13.6			-6.1b	1.6	-26.5
	trans-trans	14.0	1.6c	nd	-6.6b	0.7	-20.9

[a] "c" means chair conformer and "b" boat conformer. "nd" means “not yet determined”.

3.2 Investigation of the different potential mechanisms

3.2.1. Addition via C3

In the postulated mechanism, **ylide *Z*** adds onto 5(4*H*)-oxazolone **1a** via nucleophilic C1 (see Section 3.1.2). We have also investigated the possibility of addition via C3 to form **int1-C3**. Our results indicate that this addition involves in fact a lower free energy barrier than addition via C1. The barrier to reverse back to **ylide Z** is however also very low, indicating a rapid equilibrium between **ylide *Z*** and **int1-C3**.

Our calculations show that **int1-C3** can cyclize to yield **int4-C3** but no low lying pathway toward a final product could be envisioned and computed from this intermediate**.**

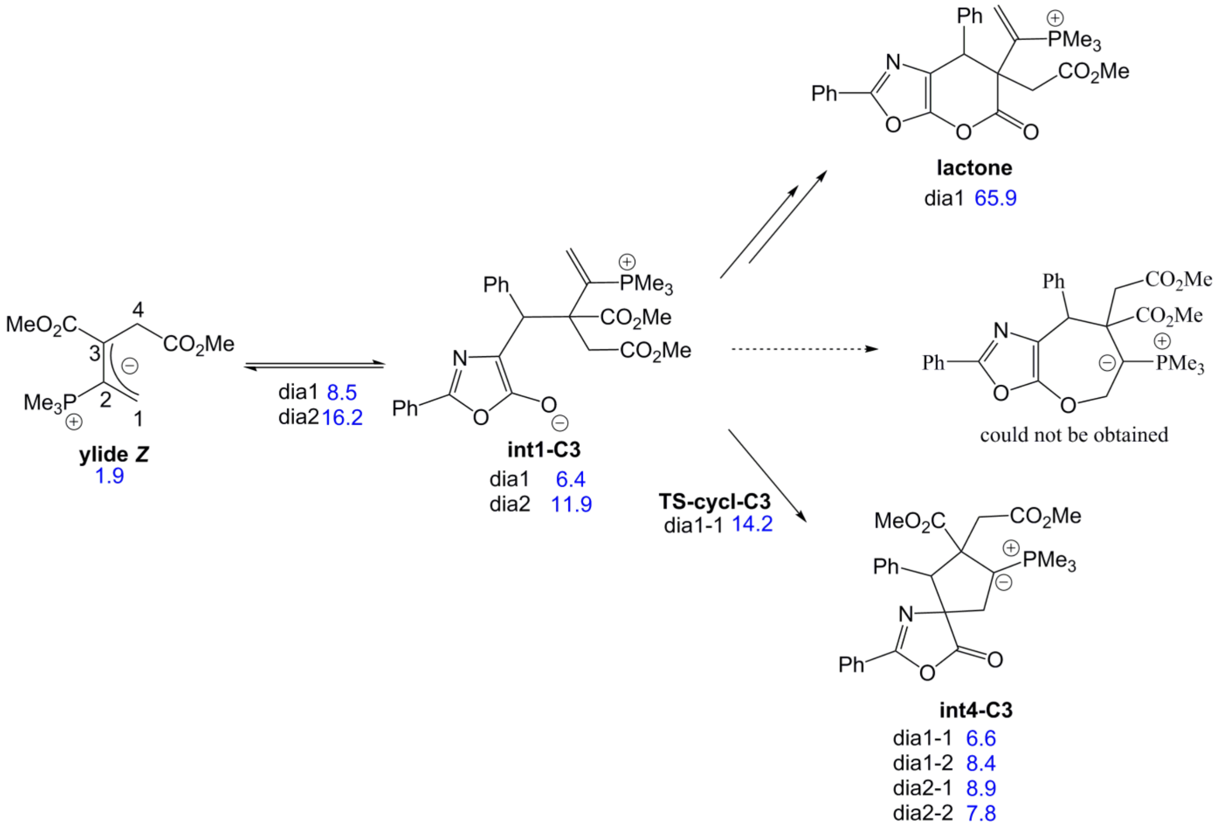

3.2.2. Cyclization of int2 via the oxygen atom

All our attempts to optimize a 8-member ring (**int5**) led to **int2** indicating the instability of such a structure.

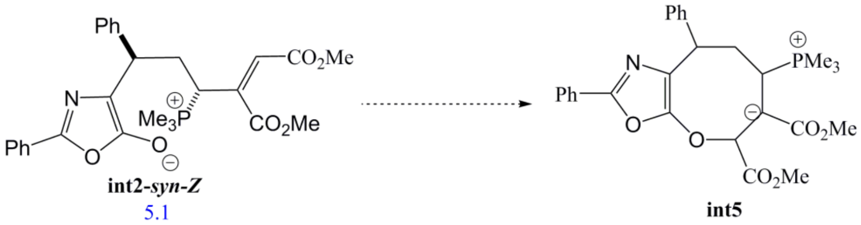

3.2.3. Cyclization from int1

We have also investigated the possibility of cyclization from **int1-Z** via either the oxygen atom or the carbon atom of the azlactone moiety. In both cases, our results show that these pathways are less favoured than the postulated mechanism yielding **5**.

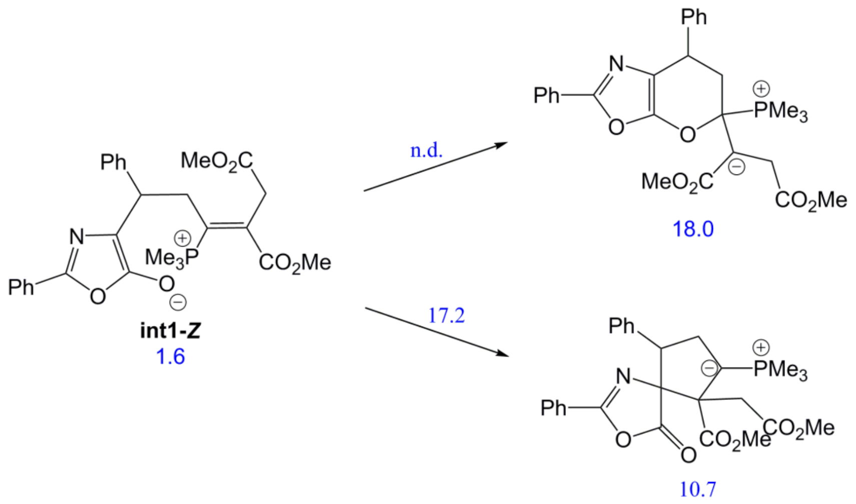

3.2.4. Mechanisms for the 1,3-hydride shift

3.2.4.1. Intramolecular double deprotonation via C

We have also envisaged intramolecular deprotonation via the carbon atom of the azlactone moeity. However, although intermediates **int-CH** are quite stable, this pathway involves a much higher free energy barrier than the deprotonation by the OH.

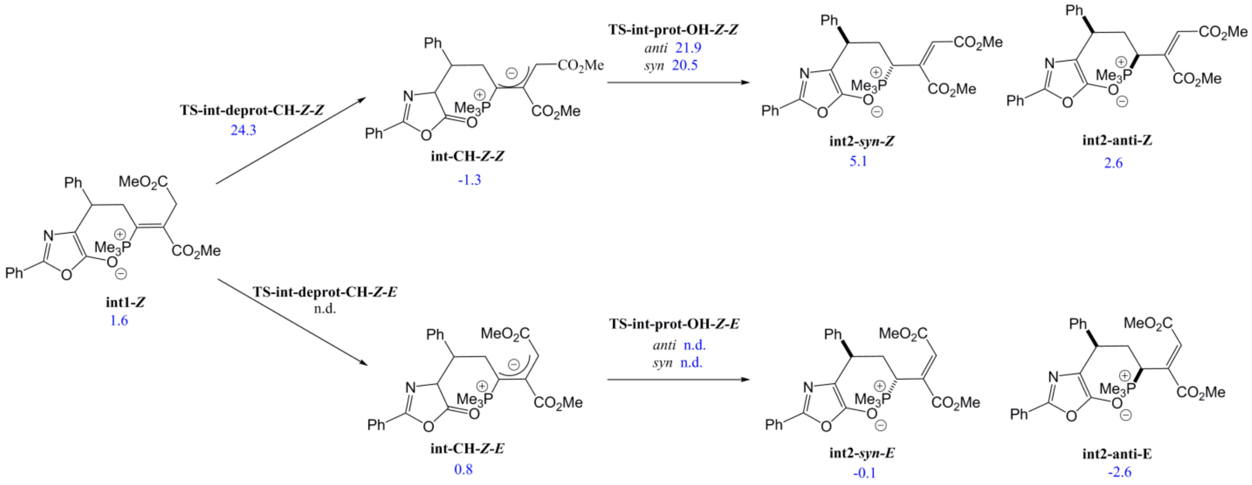

3.2.4.2. Concerted mechanism

One could also imagine this transformation to occur through a concerted pathway. This kind of 1,3-hydride shift mechanism is however not allowed according to Woodward-Hoffmann rules. Indeed, no TS connecting ***int1-Z*** and **int2** could be found.

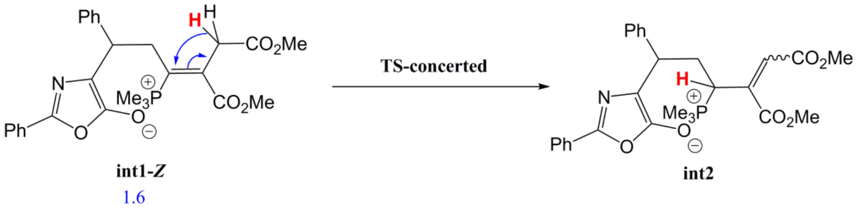

3.2.4.3. Intermolecular mechanism

We have also explored the possibility of intermolecular deprotonation-protonation, involving the ylide (probably the most basic species in the media) or other species. Intermediates formed (**int-deprot**)

are predicted to be low lying (4-8 kcal/mol) but free energy barrier to deprotonation (33.0 kcal/mol) indicate that this mechanism is disfavored over the intramolecular one (see section 3.1.3).

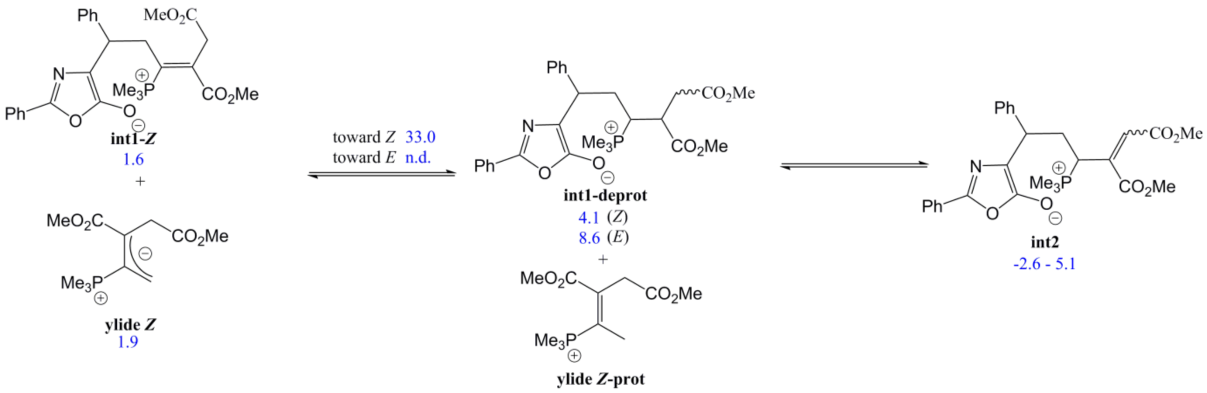

3.3 Benchmark calculations

In order to identify the most suitable method to describe the reaction of interest, we have performed single point calculations on most relevent B3LYP-D3/6-31+G(d)(THF) geometries (Table S3). DFT calculations were performed using Jaguar 8.5[5] while *ab initio* calculations were carried out using ORCA 4.0 program package.[6]

Table S3. Benchmark calculations[a]

	**B3LYP-D3/ 6-31+G***	**B3LYP-D3/ 6-311+G****	**M06-2X-D3/ 6-311+G****	**SCS-MP2/ cc-pVTZ**
TS ylide formation	26.0	25.3	25.9	29.3
Ylide Z	1.7	4.8	1.9	3.1
Addition TS	16.0	19.2	15.0	16.7
Int1	0.6	11.2	1.6	4.4
Proton transfer I TS	20.6	26.6	15.5	19.9
Proton transfer II TS	21.8	14.7	17.5	n.d.
Cyclization TS	16.7	24.7	15.7	17.7
products	-13.0	-11.8	-26.5	n.d.

[a] All energy values include solvation energy as well as entropic and thermal contributions obtained at the B3LYP-D3/6-31+G* level of theory.

[5] Jaguar 8.5; Schrodinger, Inc.: New York, NY, 2014.
[6] F. Neese. *Wiley Interdiscip. Rev.: Comput. Mol. Sci.* **2012**, *2*, 73–78

3.4 Cartesian coordinates and energies

3.4.1. Ylide formation

3.4.1.1. Allene (**4p**)
E(B3LYP-D3/6-31+G*(THF)) = -611.75570049
E(M06-2X-D3/6-311+G**) = -611.6525578
Gtot(B3LYP-D3/6-31+G*(THF)) = -611.625335

C	0.5524894	0.2424253	6.9209329
C	-0.7400943	0.1766486	6.6650410
C	-1.4483710	1.4415731	6.2986361
O	-2.7704685	1.2250792	6.1490528
O	-0.9260090	2.5343449	6.1528950
C	-1.5268624	-1.1175468	6.7893080
H	-0.8532334	-1.9339336	7.0793350
H	-2.2846929	-1.0337565	7.5754329
C	-2.2667401	-1.5772142	5.5385953
O	-1.6979783	-1.1425247	4.4044621
O	-3.2466956	-2.2987298	5.5710972
C	-2.3463159	-1.5433225	3.1750311
H	-1.7696513	-1.0764696	2.3754081
H	-2.3326743	-2.6323350	3.0729717
H	-3.3813768	-1.1903772	3.1590588
C	-3.5682420	2.3754751	5.7924862
H	-4.5838299	1.9979376	5.6642636
H	-3.5337263	3.1222140	6.5916250
H	-3.2026387	2.8208384	4.8626668
H	2.5860828	0.1655797	6.4233500
C	1.8256723	0.2993206	7.1944920
H	2.1800863	0.4940282	8.2081357

3.4.1.2. PMe_3
E(B3LYP-D3/6-31+G*(THF)) = -461.10665009
E(M06-2X-D3/6-311+G**) = -461.04687164
Gtot(B3LYP-D3/6-31+G*(THF)) = -461.022499

P	0.2354809	0.8374837	6.5007519
C	-0.0384911	2.6818154	6.5577783
H	0.5925915	3.1283978	7.3347273
H	0.2444149	3.1332750	5.5998711
H	-1.0851346	2.9342707	6.7694025
C	-1.1366219	0.3527606	5.3337976
H	-1.2244409	-0.7390188	5.2933220
H	-2.1032605	0.7713397	5.6412604
H	-0.9082662	0.7052656	4.3213812
C	-0.5482698	0.3560475	8.1233940
H	-0.6197062	-0.7353918	8.1934212
H	0.0741683	0.7028269	8.9563260
H	-1.5537045	0.7809074	8.2358989

3.4.1.3. Ylide formation TS
E(B3LYP-D3/6-31+G*(THF)) = -1072.8512744
E(M06-2X-D3/6-311+G**) = -1072.6885213
Gtot(B3LYP-D3/6-31+G*(THF)) = -1072.613327

C	-0.7622146	0.0494263	5.7249789
C	-1.5930792	1.1381530	6.1960978
O	-2.7587154	0.7120955	6.7759327
O	-1.3239036	2.3402620	6.1075888
C	-1.1490656	-1.4108178	5.8859855
H	-2.2043294	-1.4786856	6.1739583
H	-1.0421718	-1.9551919	4.9410391
C	-0.3771114	-2.1768442	6.9533314
O	-0.1715995	-3.4558209	6.5763283
O	-0.0048693	-1.7377075	8.0276173
C	0.4897215	-4.3094433	7.5369309
H	0.5520506	-5.2909300	7.0634595
H	1.4904886	-3.9293506	7.7601183
H	-0.0905370	-4.3664723	8.4622566
C	-3.6248425	1.7472124	7.2642302
H	-4.4844532	1.2324294	7.7000131
H	-3.1194340	2.3496478	8.0260953
H	-3.9501788	2.4055622	6.4513774
H	2.2574558	0.6341383	4.0860366
C	1.1914615	0.4275861	4.0943684
H	0.7103592	0.2674715	3.1270398
C	0.4578676	0.3741847	5.1930668
P	1.9844371	1.0481339	6.9920581
C	0.8575078	1.4465412	8.3897619
H	1.4164061	1.5885545	9.3219624
H	0.2905497	2.3495695	8.1506860
H	0.1595143	0.6130265	8.5074833
C	3.0969627	2.5269525	6.9002142
H	3.5564396	2.7415114	7.8739233
H	3.8893590	2.3522067	6.1642873
H	2.5144182	3.3971254	6.5802799
C	3.0799904	-0.2606805	7.7098307
H	3.5762657	0.0897170	8.6238269
H	2.4608963	-1.1300111	7.9503220
H	3.8416795	-0.5576234	6.9808553

3.4.1.4. Ylide *Z*
E(B3LYP-D3/6-31+G*(THF)) = -1072.88478072
E(M06-2X-D3/6-311+G**) = -1072.721575277
Gtot(B3LYP-D3/6-31+G*(THF)) = -1072.643991

C	-0.9457652	0.4240511	6.2381312
C	-1.7570238	1.5537093	6.0151542
O	-3.0882829	1.3438370	6.3408205
O	-1.4048594	2.6792770	5.5793144
C	-1.5723519	-0.8487879	6.7611862
H	-0.8084652	-1.4689367	7.2566943
H	-2.3293918	-0.6311728	7.5204161
C	-2.2695745	-1.7811832	5.7767110
O	-1.7446807	-1.7344619	4.5392477
O	-3.1853834	-2.5312171	6.0787432
C	-2.3266682	-2.6295817	3.5698532
H	-1.7975474	-2.4343203	2.6356672
H	-2.1892831	-3.6715165	3.8759617
H	-3.3958691	-2.4290108	3.4527962
C	-3.9560841	2.4631969	6.1577503
H	-4.9546142	2.1140039	6.4338380
H	-3.6669531	3.3054843	6.7972103
H	-3.9569469	2.8045301	5.1166230
H	2.3042280	-0.6708083	5.5960170
C	1.2262426	-0.6897226	5.6985297
H	0.7566878	-1.6645587	5.6084648

C 0.4610281 0.4221632 5.9173306
P 1.4004861 1.9952149 5.9260453
C 0.9776935 3.0369892 7.3610439
H 1.6440866 3.9062571 7.3798444
H -0.0598403 3.3587035 7.2791551
H 1.1248692 2.4490796 8.2733361
C 1.3304527 2.9311961 4.3618683
H 1.9068920 3.8571384 4.4706673
H 1.7774864 2.3178916 3.5717753
H 0.2901324 3.1545875 4.1271186
C 3.1855493 1.6485807 6.1493100
H 3.7012285 2.6104952 6.2457584
H 3.3514944 1.0601517 7.0561899
H 3.5989225 1.1183717 5.2866106

3.4.1.5. Ylide *E*
E(B3LYP-D3/6-31+G*(THF)) = -1072.87360556
E(M06-2X-D3/6-311+G**) = -1072.70480437
Gtot(B3LYP-D3/6-31+G*(THF)) = -1072.632536

C -0.4551031 0.1274064 6.1196888
C -1.4935117 1.0373391 6.4611271
O -2.7224374 0.3783738 6.5770159
O -1.4425843 2.2645903 6.6749583
C -0.8601505 -1.2900659 5.7629649
H -1.7477714 -1.2892927 5.1229240
H -0.0853791 -1.7938451 5.1707595
C -1.1656791 -2.2231572 6.9300132
O -2.1720789 -3.0717971 6.6384453
O -0.5621590 -2.2680963 7.9918265
C -2.5091295 -4.0402414 7.6555411
H -3.3210285 -4.6372483 7.2367057
H -1.6471669 -4.6746281 7.8837462
H -2.8377643 -3.5371479 8.5694636
C -3.8422634 1.2093034 6.8728728
H -4.7058796 0.5405229 6.9292578
H -3.7164313 1.7332283 7.8270719
H -4.0061073 1.9587847 6.0888976
H 0.5588976 2.6261594 5.4288241
C 1.3030994 1.8828521 5.6903555
H 2.3317375 2.2211459 5.7447476
C 0.9041212 0.6158773 6.0029096
P 2.2737197 -0.4192856 6.6447760
C 2.3188136 -2.1530838 6.0666883
H 3.2740260 -2.5891210 6.3817063
H 1.5069052 -2.7209403 6.5248316
H 2.2476614 -2.1983712 4.9754057
C 2.1657842 -0.4746425 8.4606187
H 2.9607042 -1.1093044 8.8681027
H 2.2602404 0.5414808 8.8565117
H 1.1869625 -0.8871726 8.7171844
C 3.9119246 0.2573931 6.2005973
H 4.6773392 -0.4596365 6.5162404
H 3.9767338 0.4015054 5.1175358
H 4.0890394 1.2106037 6.7065911

3.4.1.6. Isomerization TS
E(B3LYP-D3/6-31+G*(THF)) = -1072.869111185
E(M06-2X-D3/6-311+G**) = -1072.69865532
Gtot(B3LYP-D3/6-31+G*(THF)) = -1072.628671

C -0.8098670 0.3060564 6.1765029
C -1.6531770 1.3829981 5.8909837
O -2.9958002 1.0724364 6.1677777
O -1.3628567 2.5193902 5.4376747
C -1.3375597 -1.0094873 6.7058837
H -0.5051576 -1.7203765 6.8254259
H -1.8036107 -0.9266159 7.6986360
C -2.3793640 -1.7443181 5.8670848
O -2.0978336 -1.7413128 4.5415093
O -3.3389167 -2.3410779 6.3243119
C -3.0154981 -2.4773448 3.7057670
H -2.6282757 -2.3840682 2.6894123
H -3.0514546 -3.5299087 4.0047769
H -4.0209472 -2.0518184 3.7728512
C -3.9529074 2.0308208 5.7299399
H -4.9336193 1.6127236 5.9763599
H -3.8266470 2.9961570 6.2353571
H -3.8888394 2.2021277 4.6482784
H 2.5949663 1.0627716 6.6617944
C 1.5349474 0.9378351 6.8776370
H 1.2073295 1.2096202 7.8810679
C 0.6398555 0.4940237 5.9827935
P 1.2281417 0.0642316 4.2993672
C 0.0310922 0.7137729 3.0940819
H 0.4593339 0.6914105 2.0868417
H -0.8673591 0.0941577 3.1467507
H -0.2373930 1.7321450 3.3878460
C 1.2904723 -1.7475523 4.0936522
H 1.5657336 -1.9918714 3.0612520
H 2.0297680 -2.1765855 4.7780024
H 0.2990281 -2.1540324 4.3114198
C 2.8884130 0.7105623 3.8844748
H 3.1318802 0.4584599 2.8456355
H 2.8976098 1.7990222 4.0035242
H 3.6409241 0.2659860 4.5443124

3.4.2. Addition

3.4.2.1. **1a**
E(B3LYP-D3/6-31+G*(THF)) = -821.57437155
E(M06-2X-D3/6-311+G**) = -821.41320496
Gtot(B3LYP-D3/6-31+G*(THF)) = -821.385844

C -2.2393861 0.1236577 6.5519910
C -0.3506741 -0.8880306 6.3648990
C -0.0924946 0.5527173 6.1760594
O -1.3498581 1.1539337 6.3075153
O 0.9043073 1.1985507 5.9546807
C 0.6403202 -1.8176432 6.3058454
H 1.6307553 -1.4075242 6.1094675
C 0.5866538 -6.0621474 6.7442529
C 1.7797092 -5.3799475 6.4859910
C 1.7721883 -3.9942872 6.3457693
C 0.5704763 -3.2601839 6.4609566
C -0.6269011 -3.9646092 6.7228871
C -0.6130753 -5.3491116 6.8611081
H 0.5905873 -7.1443679 6.8551018
H 2.7139043 -5.9293716 6.3957312
H 2.7023699 -3.4653493 6.1444607

H -1.5561817 -3.4140302 6.8141009
H -1.5417587 -5.8789600 7.0617384
C -6.3388005 1.1260673 7.0778193
C -5.4039811 2.1379354 6.8393869
C -4.0576509 1.8209174 6.6653484
C -3.6404275 0.4802632 6.7306348
C -4.5842803 -0.5357758 6.9703629
C -5.9258218 -0.2105362 7.1425759
H -7.3885753 1.3766607 7.2130620
H -5.7239703 3.1757569 6.7887608
H -3.3296855 2.6047362 6.4798558
H -4.2543198 -1.5690976 7.0178818
H -6.6526920 -0.9977929 7.3271682
N -1.7186862 -1.0624460 6.5938849

3.4.2.2. Addition TS *E*
E(B3LYP-D3/6-31+G*(THF)) = -1894.45321610
E(M06-2X-D3/6-311+G**) = -1894.123406180
Gtot(B3LYP-D3/6-31+G*(THF)) = -1893.995970

C -0.7042227 0.4739618 10.6135001
C -0.1939640 0.0788169 8.5560134
C -0.2831108 1.5058060 8.6836340
O -0.6182561 1.7253176 10.0393583
O -0.1406807 2.4553151 7.9129950
C -1.7576383 0.1305055 14.7311398
C -1.6925713 1.3925656 14.1335517
C -1.3455982 1.5199508 12.7882195
C -1.0614942 0.3761757 12.0213460
C -1.1258628 -0.8927567 12.6302244
C -1.4715248 -1.0114362 13.9724764
H -2.0303046 0.0349338 15.7798031
H -1.9155354 2.2828378 14.7173342
H -1.2994966 2.5011910 12.3251595
H -0.9051803 -1.7738073 12.0346505
H -1.5202951 -1.9969662 14.4306996
N -0.4612017 -0.5042314 9.7922555
C 0.0592388 -0.5937526 7.3398460
H 0.0023146 0.0478524 6.4650202
C 2.1872902 -0.9010057 7.0109274
H 2.3718612 -1.2736161 8.0096707
H 1.9771145 -1.6496558 6.2624908
C 2.8163611 0.7894284 5.2484335
C 2.8009437 0.2962292 6.5640955
P 3.6427851 1.2299663 7.9012370
C 3.2968349 0.4718563 9.5219088
H 3.7444376 1.1126244 10.2892439
H 2.2187314 0.4026266 9.6894359
H 3.7410471 -0.5253025 9.5881606
C 5.4518268 1.1570975 7.6822462
H 5.9336003 1.7893764 8.4364929
H 5.7730991 0.1182148 7.8169793
H 5.7370781 1.4778911 6.6778607
C 3.1050569 2.9690002 8.0666923
H 3.4569437 3.3516254 9.0312103
H 3.5136033 3.6014070 7.2757011
H 2.0124344 2.9901270 8.0185677
C 2.1130906 0.0651467 4.1910618
O 2.3092461 0.6473334 2.9671407
O 1.4078091 -0.9370850 4.3018610
C 1.6101397 0.0378982 1.8698256
H 1.8165426 0.6693896 1.0034597
H 1.9706797 -0.9807873 1.6938635
H 0.5339722 0.0029818 2.0665210
C 3.5711499 2.0202352 4.7859731
H 2.9334540 2.6730188 4.1842944
H 3.9409997 2.6280638 5.6143086
C 4.8122881 1.6720965 3.9651225
O 4.9334070 2.4630108 2.8872382
O 5.6219673 0.8117135 4.2644218
C 6.0841499 2.2212204 2.0455329
H 6.0003690 2.9333468 1.2231409
H 7.0095311 2.3875632 2.6045200
H 6.0683083 1.1951964 1.6676246
C -1.1841251 -4.6329766 6.5483760
C -1.2715681 -3.6450793 5.5635667
C -0.8557241 -2.3431576 5.8379717
C -0.3500372 -1.9968205 7.1044032
C -0.2688421 -2.9995595 8.0887603
C -0.6799184 -4.3028639 7.8100124
H -1.5042051 -5.6506798 6.3343087
H -1.6675358 -3.8883148 4.5797556
H -0.9022790 -1.5858931 5.0612691
H 0.1057177 -2.7476148 9.0739506
H -0.6094222 -5.0649149 8.5836545

3.4.2.3. Addition TS *Z*
E(B3LYP-D3/6-31+G*(THF)) = -1894.47490944
E(M06-2X-D3/6-311+G**) = -1894.152321098
Gtot(B3LYP-D3/6-31+G*(THF)) = -1894.016565

C -0.9300165 1.2257003 7.6299388
C -0.5529850 -0.6635103 6.6604158
C -1.3171069 0.1208118 5.7356062
O -1.5556268 1.3399036 6.4105588
O -1.7345726 -0.0598966 4.5933297
C -1.0325311 4.5410789 10.3098829
C -1.7051397 4.6117568 9.0865373
C -1.6784670 3.5330263 8.2030689
C -0.9756457 2.3617950 8.5377836
C -0.2999978 2.2968332 9.7717985
C -0.3289405 3.3782567 10.6472425
H -1.0563245 5.3838319 10.9971179
H -2.2553273 5.5111549 8.8187503
H -2.2012933 3.5903211 7.2531209
H 0.2418563 1.3918363 10.0290185
H 0.1968558 3.3155789 11.5975766
N -0.3309069 0.0859544 7.8163058
C -0.0954909 -1.9699612 6.3717325
H -0.5533343 -2.3689621 5.4679895
C 1.7987173 -1.8367781 5.3618266
H 2.1725022 -2.8412779 5.5165860
H 1.3696138 -1.6964406 4.3744248
C 2.1293862 0.5857269 5.5885991
C 2.4395786 -0.7245969 5.9615778
P 3.5751501 -1.0948896 7.3515399
C 3.8570459 -2.9024762 7.4048154
H 4.5923016 -3.0892435 8.1961973
H 2.9342158 -3.4350091 7.6479641
H 4.2595960 -3.2673024 6.4552490

C	5.2367369	-0.4258790	6.9974329
H	5.9224534	-0.7515117	7.7878479
H	5.5732358	-0.8309455	6.0373778
H	5.2056160	0.6623497	6.9552877
C	3.0025210	-0.6690970	9.0402517
H	2.9155563	-1.5874512	9.6266674
H	3.7183816	0.0146609	9.5013996
H	2.0317190	-0.1767089	8.9668137
C	2.4352897	1.6897271	6.4819959
O	1.8450454	2.8553783	6.1341074
O	3.1249512	1.6023758	7.5050089
C	2.0292222	3.9547015	7.0467362
H	1.4886596	4.7929431	6.6043745
H	1.6120342	3.7058307	8.0265189
H	3.0914242	4.1955407	7.1508881
C	1.4916343	0.8645904	4.2453292
H	0.5489935	0.3347457	4.0793541
H	1.2437909	1.9277705	4.1631329
C	2.4216349	0.5461069	3.0785134
O	1.7051718	0.3122436	1.9593025
O	3.6373759	0.5206577	3.1109900
C	2.4627606	0.0491244	0.7582185
H	1.7219326	-0.0899849	-0.0311289
H	3.1185831	0.8928387	0.5239219
H	3.0662195	-0.8553963	0.8796585
C	0.7686399	-5.0695096	9.2404354
C	0.6172221	-5.3630319	7.8807465
C	0.3291794	-4.3460953	6.9725717
C	0.1880425	-3.0077992	7.3936296
C	0.3221119	-2.7319741	8.7668007
C	0.6122319	-3.7517900	9.6757623
H	0.9960064	-5.8611066	9.9511645
H	0.7214301	-6.3872693	7.5290810
H	0.2143570	-4.5852404	5.9164870
H	0.1883851	-1.7151621	9.1141657
H	0.7091400	-3.5127268	10.7331776

3.4.2.4. **int1-*Z***
E(B3LYP-D3/6-31+G*(THF)) = -1894.4898829
E(M06-2X-D3/6-311+G**) = -1894.164154971
Gtot(B3LYP-D3/6-31+G*(THF)) = -1894.028925

C	-0.8034936	0.7031491	9.7687995
C	-0.5815366	-0.1899855	7.8175463
C	-1.7837158	0.5455718	7.7528905
O	-1.8866459	1.1426587	9.0504433
O	-2.6728458	0.7544337	6.9097569
C	-0.3039084	1.9832487	13.8160193
C	-1.3333675	2.5104950	13.0293829
C	-1.5039434	2.0973997	11.7094633
C	-0.6412791	1.1372143	11.1391779
C	0.3864546	0.6015927	11.9474231
C	0.5529380	1.0238657	13.2628735
H	-0.1748306	2.3086871	14.8456090
H	-2.0106198	3.2523396	13.4481502
H	-2.3052293	2.5141308	11.1063283
H	1.0352377	-0.1648663	11.5346130
H	1.3503887	0.5942456	13.8664426
N	-0.0181963	-0.0846032	9.0686115
C	-0.0490107	-1.0795967	6.7333379
H	-0.3590111	-0.6354265	5.7824896
C	1.5123398	-1.1994426	6.7382275
H	1.7927086	-1.7857008	7.6115127
H	1.8200059	-1.7990362	5.8732594
C	2.2596427	0.9185649	5.6090976
C	2.2358224	0.1382948	6.7204128
P	3.1371374	0.5968814	8.2754452
C	3.1405659	-0.8401388	9.3992524
H	3.7115898	-0.5429882	10.2867498
H	2.1140909	-1.0812889	9.6793693
H	3.6361310	-1.7002380	8.9381488
C	4.9250581	0.8079916	7.9403784
H	5.4238862	0.9657585	8.9038814
H	5.3049891	-0.1170414	7.4931221
H	5.1254236	1.6534679	7.2844554
C	2.4412528	2.0037523	9.2159987
H	2.1362027	1.6487074	10.2023857
H	3.2016593	2.7831989	9.3022125
H	1.5729570	2.4036248	8.6916592
C	2.8047683	2.3105018	5.6556870
O	2.7476435	2.9215839	4.4698188
O	3.2316065	2.8524399	6.6668207
C	3.2019804	4.2979420	4.4301336
H	3.0438701	4.6232346	3.4014932
H	2.6185917	4.9062058	5.1268434
H	4.2617785	4.3493247	4.6949104
C	1.7430335	0.4668300	4.2584304
H	2.5088838	0.6469102	3.4975544
H	1.5399542	-0.6076214	4.2403983
C	0.4751208	1.1550071	3.7586489
O	-0.2212948	1.7083677	4.7531540
O	0.1335779	1.1459529	2.5909665
C	-1.4937159	2.3123437	4.4089485
H	-1.9884059	2.4876434	5.3612612
H	-1.3212456	3.2395323	3.8544575
H	-2.0840265	1.6226293	3.8012815
C	-1.6230097	-5.1266607	6.8969961
C	-1.6110250	-4.4271789	5.6883457
C	-1.1086489	-3.1228914	5.6376697
C	-0.6203024	-2.4974351	6.7905148
C	-0.6412303	-3.2076090	8.0004435
C	-1.1347397	-4.5110584	8.0547138
H	-2.0160096	-6.1402740	6.9399169
H	-1.9970577	-4.8942182	4.7843108
H	-1.1071042	-2.5825736	4.6919580
H	-0.2857816	-2.7205688	8.9054952
H	-1.1472401	-5.0455675	9.0027947

3.4.2.5. **int1-*E***
E(B3LYP-D3/6-31+G*(THF)) = -1894.4810110
E(M06-2X-D3/6-311+G**) = -1894.152745644
Gtot(B3LYP-D3/6-31+G*(THF)) = -1894.019519

C	-0.8139574	0.3766575	10.6546701
C	-0.5020865	0.2417184	8.5274041
C	-1.2695372	1.4026029	8.7113052
O	-1.4592230	1.4711582	10.1340457
O	-1.7220200	2.3115536	7.9840805
C	-0.8128798	-0.3914131	14.8604029
C	-1.4894411	0.7224333	14.3537611

C	-1.4957272	0.9894765	12.9835138
C	-0.8210265	0.1380496	12.0879099
C	-0.1409686	-0.9847288	12.6080878
C	-0.1382653	-1.2428281	13.9747261
H	-0.8116454	-0.5970895	15.9283498
H	-2.0193323	1.3892616	15.0309494
H	-2.0269939	1.8548355	12.5973180
H	0.3758079	-1.6458742	11.9185096
H	0.3905863	-2.1152463	14.3543219
N	-0.2425151	-0.3643769	9.7405911
C	-0.1080442	-0.3970375	7.2254630
H	-0.4102380	0.2831800	6.4231100
C	1.4435686	-0.6226766	7.1061873
H	1.8312640	-0.9100316	8.0868350
H	1.6331504	-1.4688122	6.4460648
C	2.4317326	0.7811152	5.2429996
C	2.1837441	0.5896193	6.5615754
P	2.6870661	1.8374002	7.8299084
C	2.7449151	1.0666743	9.4753048
H	3.0090215	1.8523597	10.1926441
H	1.7908764	0.6140303	9.7539672
H	3.5316425	0.3033873	9.4807732
C	4.3840396	2.4870871	7.5988152
H	4.7464979	2.8070491	8.5820190
H	5.0329133	1.7025531	7.2016604
H	4.3970652	3.3491306	6.9269186
C	1.5366747	3.2495714	7.8147219
H	1.7381772	3.8693119	8.6963054
H	1.7120042	3.8459257	6.9125165
H	0.4938045	2.9159322	7.8255202
C	2.0783156	-0.2356650	4.1774419
O	0.9078668	-0.8492962	4.3482292
O	2.8122153	-0.4192067	3.2193333
C	0.5427906	-1.8346419	3.3472208
H	-0.3894316	-2.2649442	3.7093957
H	0.4031320	-1.3480148	2.3779065
H	1.3207730	-2.5985018	3.2689610
C	3.1887194	1.9275524	4.6008762
H	2.6860329	2.2368223	3.6798093
H	3.2344726	2.8144501	5.2369070
C	4.6235567	1.5141018	4.2629716
O	5.0147021	2.0274683	3.0917110
O	5.3320359	0.8355799	4.9834867
C	6.3579395	1.6901607	2.6639694
H	6.4846689	2.1784259	1.6970090
H	7.0929682	2.0620837	3.3832476
H	6.4576951	0.6058258	2.5644828
C	-2.1965970	-4.1568972	6.5912770
C	-2.5074279	-3.0364479	5.8170623
C	-1.8304008	-1.8306705	6.0243897
C	-0.8373970	-1.7212358	7.0057858
C	-0.5418756	-2.8496538	7.7852728
C	-1.2116962	-4.0564059	7.5786754
H	-2.7197942	-5.0975122	6.4312625
H	-3.2781941	-3.0992876	5.0510961
H	-2.0779447	-0.9623843	5.4161153
H	0.2006212	-2.7724026	8.5738957
H	-0.9677545	-4.9204497	8.1942053

3.4.3. Double bond migration

3.4.3.1. TS proton transfer I - *ZZ*

E(B3LYP-D3/6-31+G*(THF)) = -1894.45957100

E(M06-2X-D3/6-311+G**) = -1894.14360221

Gtot(B3LYP-D3/6-31+G*(THF)) = -1894.004033

C	-1.0377658	1.3679955	8.5996972
C	-0.5510022	-0.2180926	7.2282750
C	-0.9392756	0.8649763	6.4666749
O	-1.2719119	1.8832232	7.3556062
O	-0.9338728	1.1236690	5.1940439
C	-1.7541128	3.7873934	12.0373236
C	-1.9346201	4.3221342	10.7583095
C	-1.7065976	3.5393671	9.6271369
C	-1.2968472	2.2015716	9.7650516
C	-1.1196613	1.6670239	11.0553292
C	-1.3445388	2.4564620	12.1797108
H	-1.9349781	4.4008103	12.9170918
H	-2.2553132	5.3547473	10.6396599
H	-1.8456826	3.9578768	8.6345459
H	-0.8141930	0.6297973	11.1589403
H	-1.2081625	2.0310444	13.1716587
N	-0.5902674	0.1377078	8.5693329
C	0.0585407	-1.4717994	6.6887604
H	-0.1791309	-1.4571633	5.6201527
C	1.6573029	-1.4915585	6.7959488
H	1.9244144	-2.1396536	7.6310464
H	2.0368146	-2.0022304	5.9019259
C	2.2126318	0.7975083	5.9211693
C	2.3105844	-0.1288175	6.9325946
P	3.0168200	0.2560694	8.5462709
C	2.4646722	-0.9447640	9.8024170
H	2.8673614	-0.6161556	10.7672792
H	1.3721118	-0.9397734	9.8386770
H	2.8379521	-1.9512747	9.5935320
C	4.8386737	0.1134381	8.5250984
H	5.2349779	0.3299028	9.5241063
H	5.1055973	-0.9110749	8.2430118
H	5.2557475	0.8083110	7.7934723
C	2.5612176	1.9052055	9.2176024
H	2.0386034	1.7579867	10.1668323
H	3.4589626	2.5048944	9.3923994
H	1.8915776	2.4326698	8.5376629
C	2.6173620	2.2388071	6.1282017
O	3.9265977	2.3772459	6.4133730
O	1.8258817	3.1593114	6.1174767
C	4.3785505	3.7386546	6.6191825
H	5.4477493	3.6634971	6.8207305
H	4.1947131	4.3298181	5.7183820
H	3.8539695	4.1903522	7.4654413
C	1.5696566	0.5442495	4.6378064
H	1.3716814	-0.5040229	4.4224860
H	0.2251644	0.9433507	4.8321001
C	2.0955467	1.2485809	3.4581783
O	1.6439820	0.6785651	2.3096309
O	2.8393880	2.2255270	3.4512669
C	2.1814221	1.2149134	1.0856480
H	1.7187772	0.6370687	0.2830174
H	1.9325283	2.2750335	0.9802271
H	3.2695130	1.0982965	1.0592477

C -1.4179078 -5.2684656 8.2018473
C -1.1890260 -5.0819956 6.8366866
C -0.7307381 -3.8488635 6.3647801
C -0.4916864 -2.7809210 7.2420333
C -0.7251629 -2.9814908 8.6117079
C -1.1859066 -4.2112329 9.0860712
H -1.7788616 -6.2256849 8.5724626
H -1.3720836 -5.8948205 6.1366726
H -0.5619823 -3.7125668 5.2974522
H -0.5716106 -2.1589953 9.3018345
H -1.3697452 -4.3420768 10.1509829

3.4.3.2. TS proton transfer I - *ZE*
E(B3LYP-D3/6-31+G*(THF)) = -1894.458957223
E(M06-2X-D3/6-311+G**) = -1894.147000317
Gtot(B3LYP-D3/6-31+G*(THF)) = -1894.004062

C -1.1961020 1.5181950 8.7671285
C -0.5918517 -0.0910880 7.4723888
C -1.0918296 0.9057680 6.6656164
O -1.5025278 1.9389193 7.4979117
O -1.1471614 1.0657868 5.3761276
C -2.2227770 3.9668314 12.1060303
C -2.5205936 4.3968846 10.8095849
C -2.1865781 3.6080455 9.7090309
C -1.5488078 2.3686306 9.8954926
C -1.2524749 1.9404354 11.2037788
C -1.5859349 2.7348763 12.2966925
H -2.4877856 4.5834012 12.9619424
H -3.0190312 5.3510759 10.6535943
H -2.4233573 3.9438083 8.7034843
H -0.7686063 0.9784414 11.3457767
H -1.3552018 2.3901995 13.3025361
N -0.6318020 0.3365637 8.7917325
C 0.0702255 -1.3283965 6.9647960
H -0.1355520 -1.3396911 5.8915075
C 1.6424858 -1.3060839 7.1265796
H 1.8698504 -1.7414536 8.0978003
H 2.0431858 -1.9896472 6.3762429
C 2.1227267 0.8499619 5.8554261
C 2.2945154 0.0563187 6.9685106
P 3.1489104 0.6473731 8.4594229
C 2.6624932 -0.3652727 9.8991043
H 3.0949332 0.1053592 10.7889611
H 1.5710255 -0.3740783 9.9821391
H 3.0468791 -1.3863495 9.8264616
C 4.9646310 0.5039258 8.3467518
H 5.4160210 0.8408303 9.2871282
H 5.2219397 -0.5470246 8.1751437
H 5.3248235 1.1116728 7.5148848
C 2.7088658 2.3418864 8.9971359
H 3.1124115 2.4911773 10.0052166
H 3.1236449 3.1015725 8.3354949
H 1.6175197 2.4181331 9.0332213
C 2.7154404 2.2364738 5.8432479
O 1.8264959 3.1612593 5.4682091
O 3.8738375 2.4988139 6.1369183
C 2.3058446 4.5228663 5.3600334
H 1.4457184 5.1032648 5.0251674
H 2.6548981 4.8802896 6.3330817
H 3.1209949 4.5820006 4.6339382
C 1.3489041 0.5761270 4.6363618
H -0.0304616 0.8036103 4.9786491
H 1.3922095 1.3935962 3.9166542
C 1.4099880 -0.7080565 3.9244720
O 0.6686996 -0.6355537 2.7793135
O 1.9955017 -1.7407915 4.2378007
C 0.6107713 -1.8383262 1.9933157
H -0.0146369 -1.5983798 1.1308892
H 1.6097113 -2.1420634 1.6646327
H 0.1624765 -2.6553176 2.5675672
C -1.3048187 -5.1729613 8.4390617
C -0.7775799 -5.0410197 7.1512156
C -0.3512332 -3.7923267 6.6952631
C -0.4449305 -2.6546182 7.5112669
C -0.9653433 -2.7992744 8.8044435
C -1.3952388 -4.0477662 9.2623321
H -1.6446729 -6.1430922 8.7957137
H -0.7041246 -5.9095445 6.4996829
H 0.0611368 -3.6924678 5.6923562
H -1.0423402 -1.9276329 9.4467202
H -1.8065279 -4.1396558 10.2660401

3.4.3.3. **int1-OH-*ZZ***
E(B3LYP-D3/6-31+G*(THF)) = -1894.464697043
E(M06-2X-D3/6-311+G**) = -1894.156988240
Gtot(B3LYP-D3/6-31+G*(THF)) = -1894.007798

C -0.8256154 0.4264477 10.3700826
C -0.6447820 0.0840050 8.2361086
C -1.6602254 0.9826404 8.4375784
O -1.7893520 1.2192362 9.7786054
C -0.5340116 0.2478997 14.6210338
C -1.4950363 1.0697989 14.0250078
C -1.5951734 1.1476288 12.6355098
C -0.7290323 0.3961624 11.8235174
C 0.2364598 -0.4314359 12.4276940
C 0.3313173 -0.5012859 13.8150916
H -0.4616459 0.1869474 15.7043199
H -2.1741986 1.6509851 14.6443463
H -2.3486017 1.7825393 12.1780893
H 0.8968607 -1.0228649 11.7999895
H 1.0789001 -1.1469835 14.2704840
N -0.1336288 -0.2411258 9.4934058
C -0.1598769 -0.5892865 6.9777872
H -0.5448509 -0.0201281 6.1253848
C 1.4053066 -0.6164540 6.8708286
H 1.7841710 -1.0762542 7.7869173
H 1.6588501 -1.3280335 6.0743800
C 2.0424711 1.2204124 5.2569856
C 2.0811990 0.7116893 6.5741947
P 2.8206155 1.5747230 7.9174493
C 3.2156630 0.4225904 9.2778828
H 3.7077468 0.9904248 10.0744876
H 2.2981147 -0.0261847 9.6666533
H 3.8928119 -0.3616186 8.9240023
C 4.4116261 2.4043583 7.5349460
H 4.9190837 2.6265219 8.4800664
H 5.0371131 1.7431863 6.9291901
H 4.2395988 3.3414445 7.0020745

C	1.7801095	2.8830222	8.6706717
H	2.3189875	3.3946581	9.4762415
H	1.5132234	3.5964809	7.8859533
H	0.8706509	2.4249009	9.0679561
C	2.6870741	2.5593988	4.9787536
O	3.8619875	2.4109477	4.3458211
O	2.2501441	3.6351516	5.3533652
C	4.4833472	3.6257995	3.8783720
H	5.4084047	3.3137424	3.3909649
H	3.8206601	4.1214341	3.1639674
H	4.6996004	4.2996716	4.7125493
C	1.4829710	0.5701221	4.1590772
H	1.0461791	-0.4141025	4.2756727
C	1.4314017	1.1296337	2.8374852
O	0.9945391	0.1921562	1.9271715
O	1.7125464	2.2794054	2.4784608
C	0.9189222	0.6346859	0.5667449
H	0.5714647	-0.2271138	-0.0083603
H	0.2158022	1.4673144	0.4574383
H	1.9005259	0.9557249	0.2014995
C	-1.3676823	-4.7419316	6.5381422
C	-1.5634497	-3.8341466	5.4950312
C	-1.2089165	-2.4911471	5.6562039
C	-0.6556267	-2.0319055	6.8584681
C	-0.4649534	-2.9533684	7.9008063
C	-0.8186191	-4.2944137	7.7433883
H	-1.6381545	-5.7885144	6.4141942
H	-1.9894373	-4.1712940	4.5522470
H	-1.3585892	-1.7929990	4.8344950
H	-0.0385840	-2.6131581	8.8400955
H	-0.6606544	-4.9932812	8.5629373
O	-2.4997606	1.6097208	7.6060406
H	-3.0526337	2.2577390	8.0897069

3.4.3.4. **int1-OH-*ZE***
E(B3LYP-D3/6-31+G*(THF)) = -1894.465632839
E(M06-2X-D3/6-311+G**) = -1894.161737998
Gtot(B3LYP-D3/6-31+G*(THF)) = -1894.006561

C	-1.1664637	1.2435051	9.0645626
C	-0.5326627	-0.1730199	7.5698377
C	-1.0801505	0.8986886	6.9247633
O	-1.5029675	1.8071832	7.8580323
O	-1.2496101	1.2340710	5.6417707
C	-2.1766101	3.2398551	12.6926345
C	-2.5233596	3.8140535	11.4661950
C	-2.1963041	3.1753965	10.2699248
C	-1.5173830	1.9456184	10.2928093
C	-1.1713513	1.3707939	11.5297318
C	-1.4984390	2.0159454	12.7188277
H	-2.4355786	3.7403209	13.6229260
H	-3.0537922	4.7631295	11.4402002
H	-2.4710626	3.6220663	9.3186324
H	-0.6536445	0.4161500	11.5420351
H	-1.2287120	1.5611930	13.6695092
N	-0.5673935	0.0893723	8.9359250
C	0.1140110	-1.3554846	6.9227306
H	-0.0670918	-1.2309030	5.8532358
C	1.6726985	-1.3797638	7.1273742
H	1.8571163	-1.8420590	8.0959961
H	2.0694203	-2.0613565	6.3711005
C	2.1530041	0.8006697	5.9029543
C	2.3418876	-0.0187068	7.0223241
P	3.1906131	0.5379751	8.4948010
C	2.8898057	-0.6209496	9.8740703
H	3.3872090	-0.2118385	10.7600043
H	1.8144605	-0.6964906	10.0610090
H	3.3056969	-1.6107280	9.6656911
C	5.0102791	0.6038387	8.3201606
H	5.4627734	0.9423897	9.2595380
H	5.3758708	-0.4001427	8.0796351
H	5.2664721	1.2898775	7.5112340
C	2.6511124	2.1449665	9.1998285
H	3.1185719	2.2620536	10.1841987
H	2.9486520	2.9814930	8.5676619
H	1.5638918	2.1248783	9.3182781
C	2.5592545	2.2516942	5.9920815
O	1.5472762	3.0783698	5.6932855
O	3.6733162	2.6490466	6.3027416
C	1.8450022	4.4951045	5.6913627
H	0.9111387	4.9848806	5.4128638
H	2.1645868	4.8169373	6.6867532
H	2.6313259	4.7190153	4.9649221
C	1.5571124	0.5115050	4.6452729
H	-0.4134528	0.9972604	5.1616066
H	1.3486824	1.3632333	4.0048272
C	1.4969912	-0.7496522	3.9540689
O	0.7496044	-0.6227731	2.8004206
O	2.0159276	-1.8318974	4.2463883
C	0.6409982	-1.8077059	2.0000965
H	0.0398103	-1.5273681	1.1319106
H	1.6257283	-2.1619511	1.6772053
H	0.1441493	-2.6119003	2.5534465
C	-1.3650116	-5.3191635	7.8924004
C	-0.7990232	-5.0434289	6.6442470
C	-0.3443264	-3.7567733	6.3513236
C	-0.4466966	-2.7223108	7.2948657
C	-1.0043900	-3.0115161	8.5474251
C	-1.4637064	-4.2987726	8.8411684
H	-1.7258165	-6.3195560	8.1223493
H	-0.7152600	-5.8300975	5.8969999
H	0.1061978	-3.5443899	5.3833347
H	-1.0817058	-2.2250939	9.2915372
H	-1.9018252	-4.5032179	9.8164805

3.4.3.5. TS proton transfer II – *syn-Z*
E(B3LYP-D3/6-31+G*(THF)) = -1894.45692654
E(M06-2X-D3/6-311+G**) = -1894.144147
Gtot(B3LYP-D3/6-31+G*(THF)) = -1894.003292

C	-1.9545452	0.4397265	9.3219579
C	-0.3449629	-0.1664104	8.0223256
C	0.0931389	0.9495362	8.7152110
O	-0.9379623	1.3442797	9.5461559
C	-5.5081876	0.6610064	11.6733723
C	-4.4842357	1.5917488	11.8701725
C	-3.3218611	1.5374444	11.1004522
C	-3.1687184	0.5420594	10.1193568
C	-4.2045435	-0.3920283	9.9248221
C	-5.3618076	-0.3301348	10.6954921

H	-6.4111753	0.7039049	12.2781773
H	-4.5887520	2.3628284	12.6303161
H	-2.5259999	2.2588453	11.2616717
H	-4.0807970	-1.1664305	9.1733483
H	-6.1516755	-1.0615591	10.5379306
N	-1.6474092	-0.4512317	8.4247148
C	0.3576210	-1.0726685	7.0403150
H	-0.1624275	-1.0145539	6.0786641
C	3.7906353	0.9006242	6.8039218
C	2.3293352	0.7100743	6.6092143
C	4.3432793	2.3012507	6.6382746
O	4.1894888	3.0134327	7.7553376
O	4.7873229	2.7386342	5.5898931
C	4.7555066	4.3461219	7.7510566
H	4.5130548	4.7670354	8.7275243
H	5.8381715	4.2820550	7.6116525
H	4.3138292	4.9482962	6.9524758
C	4.6602380	-0.0626883	7.2082373
H	4.3527771	-1.0965507	7.3013664
C	6.0514861	0.2190703	7.5861567
O	6.6719052	-0.9133650	7.9974066
O	6.6134325	1.3081297	7.5587859
C	8.0391419	-0.7636592	8.4327314
H	8.3480622	-1.7525685	8.7770640
H	8.6739204	-0.4322411	7.6048316
H	8.1054033	-0.0383425	9.2492817
O	1.1652396	1.6822116	8.7066354
H	1.7886992	1.3243201	7.6878209
P	1.6361070	1.6055555	5.1886190
C	1.6168653	3.4052660	5.5080416
H	0.9294279	3.8803711	4.8001597
H	1.2654015	3.5762734	6.5304273
H	2.6106689	3.8348599	5.3761052
C	-0.1077085	1.1901135	4.8484222
H	-0.4835151	1.9294962	4.1328144
H	-0.1923993	0.1978781	4.3992184
H	-0.7015510	1.2378502	5.7645639
C	2.5355672	1.2980129	3.6294378
H	2.0860330	1.8747302	2.8130710
H	3.5807994	1.5900391	3.7608820
H	2.4816480	0.2299859	3.3899426
C	0.1319465	-5.2534678	8.2126990
C	-0.1881118	-4.8689450	6.9095075
C	-0.1245497	-3.5212958	6.5418154
C	0.2547473	-2.5415999	7.4654496
C	0.5747446	-2.9391384	8.7704640
C	0.5151203	-4.2818545	9.1430323
H	0.0786653	-6.3004975	8.5040865
H	-0.4928283	-5.6160529	6.1791972
H	-0.3830419	-3.2291808	5.5248711
H	0.8550387	-2.1851882	9.5030876
H	0.7606008	-4.5713789	10.1628239
C	1.8654240	-0.7513682	6.8294716
H	2.3581812	-1.0629689	7.7570640
H	2.2463426	-1.4254367	6.0492610

3.4.3.6. TS proton transfer II – *anti-Z*

E(B3LYP-D3/6-31+G*(THF)) = -1894.455825

E(M06-2X-D3/6-311+G**) = -1894.141414

Gtot(B3LYP-D3/6-31+G*(THF)) = -1894.001567

C	-1.9185430	0.3288340	9.3056859
C	-0.2158889	-0.3391964	8.1762768
C	0.2396113	0.6092389	9.0662961
O	-0.8490762	1.0449808	9.7970903
C	-5.7975120	0.8987976	10.9856140
C	-4.7192061	1.6534229	11.4560954
C	-3.4445123	1.4776102	10.9169227
C	-3.2334002	0.5367381	9.8944455
C	-4.3231495	-0.2217771	9.4252507
C	-5.5917993	-0.0395543	9.9670503
H	-6.7900781	1.0393544	11.4077342
H	-4.8710693	2.3851590	12.2465527
H	-2.6092422	2.0680072	11.2824157
H	-4.1555159	-0.9500704	8.6372240
H	-6.4247134	-0.6327527	9.5953086
N	-1.5866321	-0.4864144	8.3458947
C	0.5126481	-1.1495460	7.1383517
H	0.0294604	-0.9399495	6.1770655
C	2.0298341	-0.8140497	7.0102823
H	2.5161448	-1.2297434	7.9001216
H	2.4226244	-1.3890878	6.1583604
C	1.7592554	1.4751066	5.8369831
C	2.3754882	0.6917404	6.9311807
P	4.1270229	0.9681624	7.2081344
C	4.6082365	0.2033454	8.7946444
H	5.5884802	0.5858480	9.0980102
H	3.8597319	0.4540246	9.5527549
H	4.6709785	-0.8843836	8.6931670
C	5.2048318	0.2568442	5.9126579
H	6.2593949	0.4215039	6.1608427
H	5.0177950	-0.8196191	5.8342568
H	4.9791198	0.7285411	4.9501078
C	4.5266011	2.7413864	7.3247139
H	5.5742846	2.8489819	7.6242657
H	4.3818788	3.2227941	6.3541615
H	3.8801677	3.2156200	8.0692781
C	1.6770417	2.9717268	6.0362778
O	2.2746328	3.6415204	5.0325010
O	1.1845893	3.5003105	7.0118568
C	2.0773637	5.0738677	5.0395468
H	2.6151145	5.4493935	4.1676025
H	1.0093755	5.2928049	4.9565467
H	2.4737057	5.5141575	5.9586099
C	1.1523182	0.9239298	4.7524078
H	1.2533431	-0.1375927	4.5566577
C	0.2439401	1.6423146	3.8437525
O	-0.0682628	0.8608214	2.7789172
O	-0.2201403	2.7630992	3.9998044
C	-1.0201772	1.4141831	1.8475047
H	-1.1811221	0.6393854	1.0954758
H	-1.9596658	1.6528382	2.3549393
H	-0.6231854	2.3212061	1.3816230
C	0.2053902	-5.4340471	7.7942260
C	0.0570276	-4.9057126	6.5097281
C	0.1425000	-3.5252660	6.3059196
C	0.3742167	-2.6539970	7.3765010
C	0.5225613	-3.1954300	8.6603430
C	0.4391958	-4.5721909	8.8704683
H	0.1324725	-6.5073452	7.9574908

H	-0.1322913	-5.5671945	5.6664588
H	0.0164839	-3.1204589	5.3026763
H	0.6838958	-2.5255286	9.5020921
H	0.5481925	-4.9734996	9.8763112
O	1.3938547	1.1760950	9.2761394
H	1.8993586	1.1257200	8.1682247

3.4.3.7. TS proton transfer II – *syn-E*
E(B3LYP-D3/6-31+G*(THF)) = -1894.457022
E(M06-2X-D3/6-311+G**) = -1894.144147
Gtot(B3LYP-D3/6-31+G*(THF)) = -1894.000301

C	-2.6413034	1.1383207	4.9556371
C	-0.7954967	0.3837651	5.7735660
C	-0.5815846	1.7047298	5.4382562
O	-1.7518010	2.1925140	4.9018471
C	-6.6608346	1.7359781	3.6587889
C	-5.7378772	2.7821439	3.5707411
C	-4.4186627	2.5976927	3.9847414
C	-4.0041455	1.3547044	4.4958588
C	-4.9389577	0.3048535	4.5833315
C	-6.2532251	0.4967828	4.1674140
H	-7.6891111	1.8841266	3.3368511
H	-6.0464244	3.7488603	3.1787591
H	-3.7060313	3.4146382	3.9178221
H	-4.6205666	-0.6530017	4.9847010
H	-6.9651307	-0.3224788	4.2428160
N	-2.1108831	0.0586943	5.4596985
C	0.0977462	-0.5846912	6.4963682
H	-0.1776082	-1.5853071	6.1329978
C	2.5490324	1.1742995	8.0056286
C	2.2821772	0.9036672	6.5493198
C	2.2160917	2.5971525	8.4251522
O	1.9280571	2.7275698	9.7315699
O	2.2113687	3.5419875	7.6539743
C	1.6083704	4.0624512	10.1799819
H	1.3781595	3.9644095	11.2420090
H	2.4604648	4.7322289	10.0301746
H	0.7441338	4.4497085	9.6326282
C	3.0253083	0.3790951	8.9962950
H	2.9885993	0.7628328	10.0096095
C	3.5205392	-1.0135242	8.9975192
O	3.9750050	-1.4627019	7.8087062
O	3.5644828	-1.6873176	10.0155178
C	4.4623954	-2.8227176	7.7975366
H	4.8258636	-2.9944205	6.7833422
H	5.2724063	-2.9448507	8.5223064
H	3.6534754	-3.5186398	8.0388448
O	0.4323865	2.5185896	5.5109693
H	1.2988571	1.9107840	6.0444824
P	3.6427977	1.2790425	5.4576438
C	4.6749598	2.6591967	6.0458243
H	5.5008652	2.8043354	5.3416223
H	4.0733529	3.5663524	6.1172339
H	5.0818357	2.4205337	7.0338282
C	3.0171835	1.7428157	3.8047416
H	3.8636752	1.9736736	3.1493383
H	2.4521306	0.9107923	3.3717644
H	2.3548886	2.6073797	3.8926227
C	4.8100914	-0.1000381	5.1403826
H	5.5125143	0.1869396	4.3500100
H	5.3525925	-0.3373709	6.0578037
H	4.2506898	-0.9849243	4.8210812
C	-0.3253149	-0.6439289	10.8192547
C	0.1524261	-1.7631051	10.1306158
C	0.2433928	-1.7394697	8.7395545
C	-0.1215081	-0.5988749	8.0104800
C	-0.6160889	0.5078100	8.7082710
C	-0.7144927	0.4875048	10.1014631
H	-0.4009093	-0.6601374	11.9044026
H	0.4542555	-2.6534008	10.6777359
H	0.6154718	-2.6151010	8.2087836
H	-0.9215461	1.3956074	8.1614407
H	-1.0965888	1.3609123	10.6254285
C	1.6107339	-0.4351178	6.1457772
H	2.1340978	-1.2961789	6.5680155
H	1.6778158	-0.5470517	5.0545803

3.4.3.8. TS proton transfer II – *anti-E*
E(B3LYP-D3/6-31+G*(THF)) = -1894.461230
E(M06-2X-D3/6-311+G**) = -1894.141414
Gtot(B3LYP-D3/6-31+G*(THF)) = -1894.004537

C	-1.7390257	-0.4011279	9.2421918
C	-0.0895656	-0.3875183	7.8552869
C	0.3048169	0.3172635	8.9703755
O	-0.7397008	0.3171801	9.8649467
C	-5.3524913	-1.2505455	11.3440044
C	-4.3640535	-0.4695671	11.9499081
C	-3.1807742	-0.1751454	11.2715616
C	-2.9720066	-0.6648140	9.9707478
C	-3.9717320	-1.4498385	9.3647673
C	-5.1500637	-1.7377139	10.0471634
H	-6.2721699	-1.4808118	11.8773212
H	-4.5136592	-0.0886592	12.9577748
H	-2.4136432	0.4289611	11.7476165
H	-3.8036612	-1.8336036	8.3627901
H	-5.9124645	-2.3490348	9.5686516
N	-1.3998785	-0.8111738	8.0536364
C	0.6661699	-0.7192777	6.5939702
H	0.2533394	-0.1109483	5.7783582
C	1.8874711	2.0854013	6.0710701
C	2.5255248	1.0682240	6.9286908
C	1.6007204	3.3962816	6.7591597
O	0.6508491	4.1344346	6.1649496
O	2.1953876	3.7630692	7.7640442
C	0.3513401	5.4104922	6.7781397
H	-0.4325796	5.8530279	6.1611426
H	-0.0007631	5.2652683	7.8036106
H	1.2405424	6.0473955	6.7854469
C	1.4806087	1.9278600	4.7794679
H	0.7828685	2.6302412	4.3350625
C	1.9911394	0.8887753	3.8700945
O	1.1133150	0.6390723	2.8683718
O	3.0831731	0.3357950	3.9385858
C	1.5405392	-0.3228811	1.8789792
H	0.7098345	-0.4094968	1.1766631
H	2.4405072	0.0277524	1.3649916
H	1.7468617	-1.2872623	2.3519569
O	1.3813811	0.9876872	9.3098084

H	1.9136613	1.1827441	8.3341133
C	0.2770669	-4.8577328	5.3489696
C	0.0616283	-3.8217368	4.4375112
C	0.1668627	-2.4916748	4.8535685
C	0.4871097	-2.1758702	6.1789310
C	0.6966373	-3.2224449	7.0861598
C	0.5938612	-4.5524681	6.6766674
H	0.1931224	-5.8946864	5.0300849
H	-0.1948792	-4.0482941	3.4043759
H	-0.0108291	-1.6850918	4.1444326
H	0.9281388	-2.9900667	8.1234941
H	0.7561947	-5.3534483	7.3954073
C	2.1892394	-0.4192995	6.7024266
H	2.5621334	-0.9929291	7.5626209
H	2.6723252	-0.8457552	5.8179436
P	4.2464224	1.3616655	7.3020445
C	4.5432828	1.6608205	9.0769407
H	5.6140636	1.7810798	9.2724491
H	4.0010234	2.5644817	9.3681168
H	4.1542888	0.8151238	9.6532715
C	5.2741481	-0.0780129	6.8410516
H	6.3290740	0.1577765	7.0189644
H	4.9977180	-0.9531534	7.4362676
H	5.1172002	-0.2984022	5.7809484
C	4.9461061	2.7695877	6.3789354
H	6.0161189	2.8510822	6.5978131
H	4.8016059	2.5918817	5.3087270
H	4.4423151	3.6925703	6.6721782

3.4.3.9. **int2-*syn-Z***
E(B3LYP-D3/6-31+G*(THF)) = -1894.485841
E(M06-2X-D3/6-311+G**) = -1894.159688
Gtot(B3LYP-D3/6-31+G*(THF)) = -1894.023953

C	-0.5656674	0.7315512	10.4661999
C	-0.5523998	0.4154722	8.3316037
C	-1.2788748	1.6093701	8.5248099
O	-1.2529081	1.8002362	9.9433369
O	-1.8542614	2.4480577	7.8083472
C	-0.1766826	0.3254176	14.7030950
C	-0.8373579	1.4318460	14.1598441
C	-0.9616929	1.5780274	12.7782918
C	-0.4258766	0.6105510	11.9051171
C	0.2378042	-0.5038084	12.4617445
C	0.3598889	-0.6404353	13.8410671
H	-0.0844485	0.2129060	15.7807846
H	-1.2631239	2.1872441	14.8172786
H	-1.4808113	2.4375056	12.3641478
H	0.6407514	-1.2609722	11.7948710
H	0.8712267	-1.5097523	14.2502170
N	-0.1417402	-0.0998317	9.5450628
C	-0.4006705	-0.3374999	7.0431831
H	-0.6218406	0.3580288	6.2292947
C	1.0242608	-0.9088280	6.8440586
H	1.2856309	-1.5147883	7.7183526
H	1.0125944	-1.5938817	5.9878920
C	2.0091552	0.8644602	5.2580762
P	2.9073421	1.0411602	7.9811213
C	3.3101195	-0.1990478	9.2489591
H	3.8859248	0.2865759	10.0450374
H	2.3698736	-0.5816660	9.6595759
H	3.8991406	-1.0169127	8.8208829
C	4.4536437	1.7541275	7.3340813
H	5.0229334	2.1980977	8.1579225
H	5.0564074	0.9758623	6.8540779
H	4.2005247	2.5310124	6.6064677
C	1.9859258	2.3717815	8.8173245
H	1.3947370	1.9390870	9.6244598
H	2.7317164	3.0578949	9.2371165
H	1.3457090	2.9137419	8.1240576
C	1.2134106	2.1449691	5.2997047
O	0.0353615	2.0402800	4.7019303
O	1.6344823	3.1346081	5.8762783
C	-0.8266022	3.2125233	4.7571161
H	-1.6719005	2.9692415	4.1124356
H	-1.1527445	3.3526727	5.7894125
H	-0.2883913	4.0856430	4.3811744
C	2.5644815	0.4292910	4.1148632
H	3.1480089	-0.4890654	4.0999808
C	2.4333026	1.1427787	2.8161695
O	2.7460880	0.3237679	1.7963330
O	2.1062883	2.3090792	2.6787994
C	2.6543036	0.8969990	0.4695820
H	2.9396883	0.0964164	-0.2143165
H	1.6307733	1.2269105	0.2700147
H	3.3363925	1.7468591	0.3758960
C	-3.1180366	-3.7067989	6.5893125
C	-2.9914729	-2.7586184	5.5717927
C	-2.1275016	-1.6708249	5.7306289
C	-1.3833198	-1.5076550	6.9061253
C	-1.5206607	-2.4645100	7.9232346
C	-2.3783239	-3.5544839	7.7663017
H	-3.7890595	-4.5547537	6.4688828
H	-3.5663444	-2.8640485	4.6536123
H	-2.0323465	-0.9379154	4.9308667
H	-0.9614373	-2.3377586	8.8460724
H	-2.4730350	-4.2857510	8.5670827
C	2.1964375	0.0732585	6.5468876
H	3.0622924	-0.5843549	6.3737398

3.4.3.10. **int2-*anti-Z***
E(B3LYP-D3/6-31+G*(THF)) = -1894.484719
E(M06-2X-D3/6-311+G**) = -1894.156946
Gtot(B3LYP-D3/6-31+G*(THF)) = -1894.025911

C	-0.1296778	-1.4041093	10.6288963
C	-0.3204900	-0.4820618	8.6878357
C	-0.6499784	0.5352805	9.6053806
O	-0.4862485	-0.1043589	10.8893073
O	-0.9857742	1.7296564	9.5503080
C	0.3975529	-4.2316700	13.7883566
C	0.0756584	-2.9039941	14.0850295
C	-0.0914497	-1.9670846	13.0644812
C	0.0588486	-2.3462211	11.7167605
C	0.3793212	-3.6908113	11.4268132
C	0.5480813	-4.6172276	12.4499134
H	0.5243465	-4.9596179	14.5865289
H	-0.0496759	-2.5951274	15.1208308
H	-0.3471849	-0.9384989	13.3022992
H	0.4768914	-3.9945218	10.3885003

H	0.7913461	-5.6494441	12.2043192
N	-0.0231303	-1.6563912	9.3488061
C	-0.4688946	-0.4247006	7.1974540
H	-0.5059136	0.6349791	6.9118562
C	0.7045864	-1.0913121	6.4338793
H	0.8316864	-2.1205252	6.7864339
H	0.4139418	-1.1672071	5.3803600
C	2.7842085	-0.5683206	5.1315753
H	1.8749766	0.7492578	6.5367938
C	3.0203392	-2.0148976	4.7632531
O	2.2373305	-2.4126403	3.7654947
O	3.7895841	-2.7336856	5.3797771
C	2.4307010	-3.7730203	3.2948387
H	1.6880245	-3.9123185	2.5087658
H	3.4427726	-3.8805594	2.8956966
H	2.2733532	-4.4835433	4.1105854
C	3.1529376	0.4384714	4.3240156
H	2.9505687	1.4708237	4.6003413
C	3.8398187	0.2220182	3.0228399
O	3.7749117	1.3313765	2.2681800
O	4.3974935	-0.8077523	2.6811784
C	4.3912153	1.2530379	0.9604712
H	4.2489017	2.2380124	0.5137068
H	5.4558152	1.0214831	1.0558262
H	3.9016348	0.4824086	0.3581541
C	-4.1295614	-2.3070634	5.7817229
C	-3.8633047	-0.9704244	5.4782168
C	-2.6895368	-0.3649471	5.9394261
C	-1.7700494	-1.0798951	6.7161988
C	-2.0517390	-2.4210455	7.0219853
C	-3.2174237	-3.0302301	6.5572124
H	-5.0408372	-2.7810663	5.4225822
H	-4.5701106	-0.3960232	4.8823926
H	-2.4912678	0.6780733	5.6967336
H	-1.3601252	-2.9775275	7.6483002
H	-3.4185779	-4.0709265	6.8053877
C	2.0673591	-0.3286263	6.4510301
P	3.2128877	-0.6811235	7.8992788
C	3.0452787	-2.3744206	8.5249984
H	3.7555299	-2.5081762	9.3491391
H	2.0195166	-2.5058940	8.8884341
H	3.2724334	-3.0851467	7.7270938
C	4.9312062	-0.3943169	7.3694892
H	5.5856754	-0.5134317	8.2403529
H	5.2028433	-1.1213649	6.6009946
H	5.0377226	0.6224412	6.9768264
C	2.9009938	0.4912458	9.2525468
H	3.6619464	0.3352750	10.0262303
H	2.9741873	1.5166619	8.8752072
H	1.9054030	0.3268597	9.6646349

3.4.3.11. **int2-*syn-E***

E(B3LYP-D3/6-31+G*(THF)) = -1894.493218

E(M06-2X-D3/6-311+G**) = -1894.168531

Gtot(B3LYP-D3/6-31+G*(THF)) = -1894.033737

C	0.0784666	-0.0993929	10.3441994
C	-0.8678102	0.3191902	8.4554283
C	-1.4150048	1.2555116	9.3462416
O	-0.7650765	0.9577780	10.5966819
O	-2.2155029	2.2101624	9.2750240
C	2.5495596	-1.7526414	13.4140870
C	1.6677644	-0.7100136	13.7135831
C	0.8554564	-0.1573525	12.7218397
C	0.9112031	-0.6424188	11.4020842
C	1.8048062	-1.6955309	11.1074756
C	2.6099419	-2.2410987	12.1015479
H	3.1779667	-2.1830696	14.1903476
H	1.6077833	-0.3244654	14.7293962
H	0.1696692	0.6493181	12.9635663
H	1.8439869	-2.0750949	10.0905890
H	3.2856857	-3.0583224	11.8560691
N	0.0409599	-0.4962765	9.0990826
C	-1.1907918	0.1803873	7.0007940
H	-1.7610236	1.0726045	6.7235615
C	0.0808975	0.1027362	6.0987134
H	0.3754446	-0.9498551	6.0166348
H	-0.1779340	0.4329874	5.0901402
C	2.5314077	0.4720935	5.6908528
P	1.2654957	2.6434767	6.9834810
C	1.5863494	2.9222121	8.7517292
H	1.5771137	4.0010379	8.9435534
H	0.8221162	2.4372138	9.3599770
H	2.5656944	2.5046922	8.9963209
C	2.5759320	3.5590396	6.1078003
H	2.5535732	4.6000476	6.4480250
H	3.5501949	3.1241220	6.3515331
H	2.4106714	3.5188567	5.0295260
C	-0.3357175	3.3866643	6.5489642
H	-0.2190696	4.4765838	6.5426058
H	-0.6431452	3.0540982	5.5529976
H	-1.0861422	3.1111929	7.2988072
C	2.3171331	0.7323220	4.2251711
O	3.0590012	-0.0393761	3.4269657
O	1.5435228	1.5883038	3.8195528
C	2.9301464	0.1931750	2.0002618
H	3.5971575	-0.5306640	1.5303567
H	1.8959083	0.0266310	1.6867362
H	3.2306655	1.2154561	1.7544834
C	3.6991441	-0.0591204	6.1023124
H	4.4315415	-0.3903047	5.3713052
C	4.0956521	-0.2199551	7.5291675
O	4.8657145	-1.3037088	7.6922666
O	3.7882562	0.5426734	8.4308486
C	5.3148095	-1.5690929	9.0467172
H	5.8359505	-2.5257260	8.9958047
H	5.9928049	-0.7777093	9.3784980
H	4.4583011	-1.6227463	9.7236129
C	-3.6558109	-3.2596886	5.9929916
C	-4.0051724	-1.9869239	5.5380496
C	-3.2174244	-0.8801698	5.8735439
C	-2.0747352	-1.0259856	6.6682368
C	-1.7338438	-2.3103615	7.1220783
C	-2.5155247	-3.4162908	6.7888654
H	-4.2661714	-4.1223494	5.7336234
H	-4.8918749	-1.8528698	4.9213431
H	-3.4987791	0.1086602	5.5139142
H	-0.8561512	-2.4301505	7.7518336
H	-2.2368012	-4.4035672	7.1531714
C	1.3681321	0.8134419	6.6090571

H 1.5818756 0.3917610 7.5930967

3.4.3.12. **int2-*anti-E***
E(B3LYP-D3/6-31+G*(THF)) = -1894.501143
E(M06-2X-D3/6-311+G**) = -1894.176424
Gtot(B3LYP-D3/6-31+G*(THF)) = -1894.040436

C	-0.5534106	1.2874252	8.6908420
C	-0.6229491	-0.2757477	7.2266999
C	-1.2665105	0.8459783	6.6211039
O	-1.1662009	1.8653118	7.6018364
O	-1.8141534	1.0645187	5.5390076
C	-0.1249545	3.5261013	12.2942739
C	-0.5949456	4.1514324	11.1356607
C	-0.7383158	3.4300862	9.9512483
C	-0.4086794	2.0631161	9.9087757
C	0.0699849	1.4393746	11.0784112
C	0.2057046	2.1652954	12.2577626
H	-0.0191404	4.0912381	13.2177689
H	-0.8551414	5.2077070	11.1548885
H	-1.1063487	3.9179346	9.0533657
H	0.3283028	0.3854775	11.0404705
H	0.5692133	1.6691646	13.1554795
N	-0.2708730	0.0165528	8.5146221
C	-0.4310305	-1.6048514	6.5685198
H	-0.6433490	-1.4305731	5.5087526
C	1.0493829	-2.1153506	6.6619233
H	1.1245041	-2.8788465	7.4437092
H	1.2814534	-2.6022631	5.7133455
C	1.8388689	0.3920075	6.4743466
H	2.0812220	-0.8945184	8.1011834
C	1.4307663	0.4989203	5.0521544
O	0.9967576	1.7183169	4.7049144
O	1.4992807	-0.4427514	4.2643237
C	0.4357692	1.8481125	3.3812328
H	0.1188989	2.8890180	3.3036281
H	1.1865076	1.6156875	2.6202444
H	-0.4231338	1.1819024	3.2843761
C	2.1134114	1.5216619	7.1991557
H	1.8743200	2.4839296	6.7631957
C	2.7188897	1.5778665	8.5283371
O	2.6881560	2.8451826	9.0031849
O	3.2531772	0.6570234	9.1537854
C	3.2770721	3.0569869	10.2998054
H	3.1604818	4.1228820	10.5011863
H	2.7506930	2.4699707	11.0549155
H	4.3369303	2.7834069	10.2921620
C	-3.1793266	-4.6598034	7.9810048
C	-3.1052976	-4.3545221	6.6202762
C	-2.2189053	-3.3709169	6.1674649
C	-1.3973947	-2.6778533	7.0637384
C	-1.4799177	-2.9909813	8.4297228
C	-2.3616250	-3.9713216	8.8848726
H	-3.8699060	-5.4216212	8.3361623
H	-3.7397151	-4.8787711	5.9081367
H	-2.1721101	-3.1349700	5.1053444
H	-0.8625784	-2.4399678	9.1347827
H	-2.4166870	-4.1951982	9.9486373
C	2.0835794	-1.0062453	7.0155175
P	3.8116361	-1.5970733	6.6805981
C	4.3644427	-2.5575678	8.1241726
H	5.3801597	-2.9332553	7.9582788
H	4.3509107	-1.9033591	9.0018901
H	3.6882211	-3.4034222	8.2885588
C	4.9672006	-0.2139978	6.4430709
H	5.9736421	-0.6191909	6.2910385
H	4.6750290	0.3730682	5.5674371
H	4.9532019	0.4173014	7.3338220
C	3.9084419	-2.6466300	5.1962013
H	4.9610609	-2.8406214	4.9617390
H	3.3975376	-3.5984134	5.3697023
H	3.4267228	-2.1241783	4.3647001

3.4.4. Cyclization
3.4.4.1. **TS-cyclo-*anti E cis-cis***
E(B3LYP-D3/6-31+G*(THF)) = -1894.478473
E(M06-2X-D3/6-311+G**) = -1894.162166
Gtot(B3LYP-D3/6-31+G*(THF)) = -1894.02042

C	-0.9784704	1.6732638	8.3139795
C	0.2123065	0.2445656	7.1969346
C	-0.3920009	1.1747258	6.2270879
O	-1.0952231	2.1092332	7.0107407
O	-0.3514239	1.2682730	5.0175165
C	-2.9208971	3.8640680	11.4103607
C	-3.1010374	4.2435084	10.0772675
C	-2.4647279	3.5421688	9.0524587
C	-1.6431820	2.4454439	9.3588011
C	-1.4623372	2.0684280	10.7026446
C	-2.0976020	2.7745780	11.7194456
H	-3.4192202	4.4132401	12.2061524
H	-3.7411284	5.0883852	9.8337451
H	-2.6047261	3.8373058	8.0168123
H	-0.8258631	1.2178514	10.9276808
H	-1.9544841	2.4755593	12.7555130
N	-0.2935977	0.5877621	8.4714273
C	0.3119211	-1.2341722	6.8502721
H	0.8733664	-1.2834915	5.9124488
C	1.0809697	-2.0611994	7.9254004
H	0.4085961	-2.2905563	8.7596843
H	1.3788905	-3.0038351	7.4570797
C	2.8869087	-0.4603635	7.3081425
H	1.9617849	-0.5813305	9.2092654
C	3.5443600	-1.1619364	6.2617961
O	3.6973821	-0.4418365	5.0999031
O	4.0089607	-2.3207920	6.3524069
C	4.3687886	-1.1149553	4.0292317
H	4.3723512	-0.4112277	3.1934219
H	5.3964605	-1.3740370	4.3061870
H	3.8412468	-2.0315673	3.7445567
C	2.1742852	0.7618611	7.0935701
H	2.2362605	1.1772311	6.0897597
C	2.1204450	1.8848873	8.0834862
O	2.6552201	1.5949527	9.2896784
O	1.6319740	2.9743704	7.8291951
C	2.5379239	2.6344679	10.2845183
H	3.0355291	2.2466013	11.1750214
H	3.0254808	3.5520181	9.9437274

H	1.4836238	2.8405694	10.4909807
C	-3.5841038	-2.9910374	6.0778490
C	-2.6282229	-2.9158132	5.0628327
C	-1.3784502	-2.3443352	5.3170116
C	-1.0621965	-1.8409882	6.5851381
C	-2.0343887	-1.9145746	7.5958383
C	-3.2830088	-2.4849737	7.3458447
H	-4.5573506	-3.4364077	5.8828363
H	-2.8547799	-3.3006610	4.0706875
H	-0.6412390	-2.2844980	4.5186243
H	-1.8133214	-1.5081024	8.5789583
H	-4.0238095	-2.5312320	8.1417201
C	2.3191715	-1.2791967	8.4477179
P	3.5150319	-2.3126526	9.3933296
C	3.0262742	-2.2503409	11.1521378
H	3.6993798	-2.8698910	11.7551875
H	3.0706378	-1.2169781	11.5115408
H	2.0022529	-2.6241055	11.2640154
C	5.1837461	-1.6089041	9.2639892
H	5.8588845	-2.1215722	9.9570114
H	5.5276650	-1.7366477	8.2353941
H	5.1382497	-0.5422907	9.5039395
C	3.5369502	-4.0708091	8.9279520
H	4.3081010	-4.5855788	9.5117958
H	2.5625662	-4.5210132	9.1440745
H	3.7561106	-4.1461569	7.8615988

3.4.4.2. **TS-cyclo-*anti E cis-trans***

E(B3LYP-D3/6-31+G*(THF)) = -1894.478799

E(M06-2X-D3/6-311+G**) = -1894.162866

Gtot(B3LYP-D3/6-31+G*(THF)) = -1894.018447

C	-1.5734542	1.7830707	7.1693621
C	-0.2096835	0.1230519	7.4417370
C	-0.5702619	0.6110994	8.7744653
O	-1.3674213	1.7504861	8.5367329
O	-0.2685149	0.2740245	9.9039392
C	-4.0824549	4.8231051	5.5470395
C	-3.9084925	4.7341324	6.9310472
C	-3.0868491	3.7468567	7.4758834
C	-2.4310007	2.8337824	6.6329373
C	-2.6064832	2.9295101	5.2396346
C	-3.4276240	3.9167770	4.7039531
H	-4.7245106	5.5934952	5.1257059
H	-4.4153522	5.4360106	7.5893893
H	-2.9520430	3.6786128	8.5513168
H	-2.0985851	2.2177917	4.5956991
H	-3.5605318	3.9815836	3.6262279
N	-0.9720401	0.8504494	6.5072211
C	0.0414872	-1.3473384	7.1173022
H	0.5005984	-1.3659345	6.1239811
C	0.9956258	-2.0507938	8.1104724
H	0.5707037	-1.9791895	9.1155154
H	1.0182065	-3.1196061	7.8598395
C	2.4620501	0.0257816	8.3346331
H	2.8487705	-1.8432249	9.1508758
C	3.0331747	0.5183527	9.5561105
O	2.8645454	1.8704413	9.7399130
O	3.6798291	-0.1536374	10.3813235
C	3.3892460	2.4056472	10.9583209
H	3.1577913	3.4734487	10.9356150
H	2.9189082	1.9399678	11.8310141
H	4.4728295	2.2579563	11.0224314
C	1.6935123	0.8714432	7.4841090
H	1.5334440	1.8783477	7.8602206
C	1.8448019	0.8801249	6.0056993
O	1.4425487	2.0489095	5.4787472
O	2.2758769	-0.0395949	5.3165931
C	1.3891400	2.1140746	4.0391800
H	1.0783126	3.1329899	3.8036639
H	2.3703385	1.9042942	3.6039873
H	0.6588814	1.3923805	3.6621382
C	-3.7012730	-3.5254534	6.8121083
C	-2.8715891	-3.3861058	5.6975034
C	-1.6695078	-2.6814485	5.8036463
C	-1.2776637	-2.1068709	7.0183914
C	-2.1188036	-2.2526298	8.1319256
C	-3.3205495	-2.9548194	8.0303939
H	-4.6394726	-4.0706147	6.7322654
H	-3.1615242	-3.8210717	4.7429644
H	-1.0327527	-2.5664240	4.9281222
H	-1.8381968	-1.8038766	9.0818062
H	-3.9631027	-3.0540372	8.9029297
C	2.4300675	-1.4722659	8.2076601
P	3.6458204	-2.2543039	7.0204135
C	4.4312039	-3.6169742	7.9510149
H	5.1381926	-4.1583937	7.3135409
H	4.9602155	-3.2079950	8.8185440
H	3.6601109	-4.3116454	8.3027607
C	4.9720272	-1.0928342	6.5826185
H	5.7814189	-1.6332139	6.0798726
H	4.5712656	-0.3193371	5.9262464
H	5.3488962	-0.6339505	7.5019297
C	2.9528845	-3.0276240	5.5195168
H	3.7610615	-3.5374673	4.9826188
H	2.2003427	-3.7694663	5.8059768
H	2.5051486	-2.2660682	4.8816633

3.4.4.3. **TS-cyclo-*anti E trans-cis***

E(B3LYP-D3/6-31+G*(THF)) = -1894.475554

E(M06-2X-D3/6-311+G**) = -1894.157121

Gtot(B3LYP-D3/6-31+G*(THF)) = -1894.018072

C	-1.4147760	1.4047217	8.6888771
C	-0.2620026	0.0702293	7.4343318
C	-1.0890186	0.8758161	6.5510876
O	-1.7482358	1.7922549	7.4080722
O	-1.2706276	0.9074970	5.3452706
C	-3.1803271	3.4554165	11.9812372
C	-3.5166362	3.8221507	10.6752338
C	-2.9400279	3.1643900	9.5885079
C	-2.0173111	2.1263032	9.8029731
C	-1.6785205	1.7633664	11.1201622
C	-2.2582103	2.4238451	12.1985893
H	-3.6327396	3.9695698	12.8263233
H	-4.2315149	4.6233488	10.5015249
H	-3.2033492	3.4478106	8.5736546
H	-0.9605398	0.9635950	11.2755837
H	-1.9916587	2.1359925	13.2131613
N	-0.5949559	0.4040606	8.7568169

C	0.0609185	-1.3760784	7.0816046
H	0.5316202	-1.3778063	6.0947775
C	1.0319931	-2.0158228	8.1003562
H	0.6031433	-1.8989962	9.1005009
H	1.0781726	-3.0958381	7.9094132
C	2.4943966	0.0877106	8.2709347
H	2.8795845	-1.7692361	9.1310051
C	2.9204115	0.5894227	9.5594629
O	2.8337361	1.9523502	9.6764592
O	3.3632425	-0.0972546	10.4928291
C	3.2154461	2.4910413	10.9486974
H	3.0801795	3.5717513	10.8640118
H	2.5852159	2.0938082	11.7512811
H	4.2612703	2.2592533	11.1767995
C	1.7656377	0.9129351	7.3914073
H	1.5456566	1.9119486	7.7527036
C	1.8686085	0.8572218	5.9187907
O	1.4164922	1.9900561	5.3548991
O	2.3105345	-0.0802401	5.2579735
C	1.3260875	1.9958043	3.9137585
H	0.8859119	2.9592889	3.6530048
H	2.3201917	1.8943588	3.4686891
H	0.6801756	1.1810937	3.5818956
C	-3.5948953	-3.6948835	6.7500581
C	-2.7752356	-3.5061822	5.6348739
C	-1.5970152	-2.7630983	5.7487396
C	-1.2205269	-2.1969368	6.9729341
C	-2.0545212	-2.3875010	8.0857615
C	-3.2300576	-3.1313397	7.9766089
H	-4.5138722	-4.2709736	6.6641167
H	-3.0544367	-3.9335693	4.6738516
H	-0.9688964	-2.6101883	4.8729346
H	-1.7879320	-1.9332706	9.0361333
H	-3.8662563	-3.2670786	8.8490681
C	2.4636597	-1.4180819	8.1791356
P	3.6656925	-2.2398661	7.0077293
C	4.4246734	-3.5925493	7.9761275
H	5.1107325	-4.1702389	7.3474297
H	4.9757103	-3.1757707	8.8262290
H	3.6399025	-4.2571609	8.3547829
C	5.0102882	-1.1156475	6.5298650
H	5.8133830	-1.6884600	6.0535373
H	4.6234693	-0.3623950	5.8419300
H	5.3945257	-0.6262509	7.4304786
C	2.9528068	-3.0572631	5.5408520
H	3.7581313	-3.5803476	5.0123757
H	2.2077179	-3.7931224	5.8602621
H	2.4962024	-2.3183768	4.8836596

3.4.4.4. **TS-cyclo-*anti E trans-trans***
E(B3LYP-D3/6-31+G*(THF)) = -1894.477694
E(M06-2X-D3/6-311+G**) = -1894.162453
Gtot(B3LYP-D3/6-31+G*(THF)) = -1894.019860

C	-0.8547658	2.0160159	6.8713294
C	0.3294103	0.2365091	7.2262425
C	-0.2046381	0.6744914	8.5192698
O	-0.8879063	1.8673847	8.2520949
O	-0.1258931	0.2373518	9.6587158
C	-2.9308338	5.3074197	5.1361123
C	-2.9588164	5.1226905	6.5210963
C	-2.2751424	4.0547036	7.1030014
C	-1.5539711	3.1581652	6.2968737
C	-1.5250253	3.3498422	4.9028531
C	-2.2107831	4.4166853	4.3302598
H	-3.4676263	6.1392415	4.6851377
H	-3.5178599	5.8110924	7.1510355
H	-2.3019155	3.9082216	8.1786505
H	-0.9666942	2.6508645	4.2865095
H	-2.1863932	4.5559538	3.2516091
N	-0.2112982	1.0887889	6.2452130
C	0.4477611	-1.2432163	6.8916996
H	1.0321374	-1.2772444	5.9651643
C	1.2201398	-2.0808607	7.9529903
H	0.5640078	-2.3191265	8.7958412
H	1.4951611	-3.0221781	7.4659129
C	3.0843600	-0.5238555	7.3395525
H	2.1677080	-0.6463969	9.2446903
C	3.7027019	-1.2296528	6.2598099
O	3.9005359	-0.4787472	5.1302585
O	4.0967624	-2.4120534	6.3150863
C	4.5199361	-1.1549960	4.0280654
H	4.5980337	-0.4116270	3.2313015
H	5.5158550	-1.5208087	4.2997305
H	3.9129189	-2.0027887	3.6921262
C	2.4375853	0.7222916	7.1700476
H	2.4613048	1.1644404	6.1776401
C	2.3958083	1.7442063	8.2507641
O	1.9232329	2.9171827	7.7680313
O	2.7167678	1.5876068	9.4216266
C	1.6830061	3.9552412	8.7386332
H	1.2556278	4.7854603	8.1746133
H	0.9777770	3.6064606	9.4976757
H	2.6177741	4.2604083	9.2185205
C	-3.4419935	-2.9251115	5.9508136
C	-2.4793648	-2.7634557	4.9519610
C	-1.2272650	-2.2290655	5.2660238
C	-0.9134171	-1.8536945	6.5779758
C	-1.8901483	-2.0135124	7.5724716
C	-3.1429003	-2.5453835	7.2626490
H	-4.4190220	-3.3384355	5.7089188
H	-2.7034360	-3.0487622	3.9258688
H	-0.4875199	-2.0919222	4.4795300
H	-1.6757827	-1.7058008	8.5930765
H	-3.8897226	-2.6587339	8.0459192
C	2.4914121	-1.3449404	8.4664460
P	3.6528790	-2.4239030	9.3976005
C	3.1510064	-2.3523709	11.1508769
H	3.7728900	-3.0213475	11.7555252
H	3.2507576	-1.3263287	11.5211544
H	2.1033613	-2.6608978	11.2402270
C	5.3409013	-1.7669663	9.2635728
H	5.9979429	-2.2649853	9.9841826
H	5.6945494	-1.9381993	8.2437860
H	5.3160388	-0.6910482	9.4641247
C	3.6213340	-4.1760650	8.9094223
H	4.3659279	-4.7261841	9.4955497
H	2.6291251	-4.5944077	9.1074102
H	3.8493634	-4.2466080	7.8441955

3.4.4.5. **TS-cyclo-*anti Z cis-cis***
E(B3LYP-D3/6-31+G*(THF)) = -1894.468822
E(M06-2X-D3/6-311+G**) = -1894.143717
Gtot(B3LYP-D3/6-31+G*(THF)) = -1894.008444

C	-1.5801602	1.3723750	8.7815417
C	-0.3060034	0.0341294	7.6725984
C	-0.9517124	0.8948312	6.7010720
O	-1.7149572	1.7990648	7.4736514
O	-0.8834455	0.9922244	5.4839006
C	-3.8465719	3.3050190	11.8308770
C	-3.9807795	3.7167558	10.5019481
C	-3.2336750	3.1040294	9.4952924
C	-2.3418746	2.0649755	9.8125929
C	-2.2061399	1.6583177	11.1538296
C	-2.9542994	2.2742258	12.1519051
H	-4.4337424	3.7815393	12.6130166
H	-4.6733266	4.5162037	10.2474755
H	-3.3442626	3.4192445	8.4619360
H	-1.5114084	0.8581961	11.3914821
H	-2.8450423	1.9501551	13.1846773
N	-0.8028214	0.3481956	8.9385871
C	0.0293425	-1.4126398	7.3487051
H	0.4765451	-1.4270569	6.3459261
C	1.0245611	-1.9971785	8.3756294
H	0.6383664	-1.7874579	9.3784300
H	1.0525204	-3.0876179	8.2721799
C	2.5473045	0.0887147	8.3468667
H	2.9502368	-1.7680415	9.2710850
C	3.3935544	0.6577877	9.3681331
O	3.5274850	2.0164922	9.2878968
O	4.0050122	0.0108148	10.2320094
C	4.3098255	2.6286813	10.3216952
H	4.2824270	3.7012633	10.1175371
H	3.8869800	2.4223444	11.3104825
H	5.3433516	2.2674693	10.2979911
C	1.7270161	0.7889370	7.4359057
H	1.6464245	0.4280206	6.4120234
C	1.5499049	2.2779407	7.4386693
O	0.9542374	2.7312498	8.5472121
O	1.8544374	2.9838674	6.4891970
C	0.7633729	4.1537157	8.6310732
H	0.2365771	4.3200210	9.5714885
H	1.7304084	4.6657783	8.6363305
H	0.1650004	4.5127463	7.7884563
C	-3.5964326	-3.7976060	7.1415422
C	-2.8491684	-3.5488237	5.9881192
C	-1.6802898	-2.7852225	6.0610380
C	-1.2409636	-2.2574310	7.2815384
C	-2.0032991	-2.5081449	8.4335929
C	-3.1683841	-3.2726405	8.3647688
H	-4.5071993	-4.3905660	7.0880853
H	-3.1767556	-3.9463527	5.0295910
H	-1.1106283	-2.5871447	5.1544613
H	-1.6935740	-2.0829468	9.3833172
H	-3.7475159	-3.4545141	9.2681057
C	2.4580734	-1.4206993	8.3532014
P	3.5887911	-2.1611693	7.0776871
C	3.3767738	-3.9713279	6.9702641
H	4.1771695	-4.4008652	6.3571728
H	3.4169607	-4.4068942	7.9751206
H	2.4114864	-4.2122396	6.5133403
C	5.2904163	-1.8157380	7.6226502
H	6.0031528	-2.2264192	6.8994720
H	5.4259987	-0.7329930	7.7000041
H	5.4601414	-2.2665474	8.6057074
C	3.4149745	-1.4767067	5.3974857
H	4.1789733	-1.9291777	4.7546237
H	2.4271487	-1.6988185	4.9833959
H	3.5689995	-0.3940809	5.4239959

3.4.4.6. **TS-cyclo-*anti Z cis-trans***
E(B3LYP-D3/6-31+G*(THF)) = -1894.468053
E(M06-2X-D3/6-311+G**) = -1894.155570
Gtot(B3LYP-D3/6-31+G*(THF)) = -1894.010218

C	-0.8051607	1.9947167	6.7913167
C	0.4355936	0.2315007	7.1172759
C	0.0301038	0.7664204	8.4414654
O	-0.6843209	1.9317923	8.1775912
O	0.2785364	0.4405479	9.5888324
C	-3.0947941	5.1713973	5.1110860
C	-2.9696900	5.0688337	6.4991755
C	-2.2168106	4.0386285	7.0641824
C	-1.5829381	3.0981522	6.2355980
C	-1.7100906	3.2062542	4.8385838
C	-2.4616500	4.2366719	4.2828824
H	-3.6830802	5.9755114	4.6746930
H	-3.4612655	5.7923914	7.1453229
H	-2.1220239	3.9584546	8.1432269
H	-1.2201730	2.4713191	4.2074343
H	-2.5582671	4.3115878	3.2021187
N	-0.2324248	1.0387222	6.1523935
C	0.4263096	-1.2709678	6.8355029
H	1.0017936	-1.3751509	5.9096497
C	1.1539814	-2.1087175	7.9217858
H	0.4900057	-2.2807047	8.7762857
H	1.3796285	-3.0804746	7.4713189
C	3.0495052	-0.6234244	7.2222058
H	2.1579859	-0.6692623	9.1480597
C	3.8938377	-1.3148468	6.3254845
O	4.4373252	-0.5612142	5.3142847
O	4.2577222	-2.5099993	6.4706840
C	5.2311852	-1.2730373	4.3622790
H	5.5554392	-0.5280774	3.6317024
H	6.1035252	-1.7365656	4.8350735
H	4.6495317	-2.0555297	3.8620030
C	2.3328252	0.6271012	7.0471659
H	2.3655784	1.3035905	7.9033576
C	2.4583424	1.4526307	5.7931981
O	2.1923860	0.7651294	4.6814094
O	2.7153693	2.6471923	5.8088059
C	2.2523351	1.5026000	3.4507450
H	1.9938309	0.7900202	2.6659204
H	1.5357780	2.3294863	3.4665305
H	3.2590952	1.9002337	3.2898828
C	-3.5626668	-2.8187434	6.1129426
C	-2.5968822	-2.8529978	5.1051951
C	-1.3152987	-2.3513593	5.3456332
C	-0.9763066	-1.8106439	6.5916865

C	-1.9555406	-1.7799608	7.5963386
C	-3.2375530	-2.2785660	7.3607531
H	-4.5623069	-3.2060923	5.9276090
H	-2.8414930	-3.2668103	4.1289582
H	-0.5699637	-2.3731980	4.5527196
H	-1.7161065	-1.3634760	8.5729848
H	-3.9847831	-2.2407597	8.1509392
C	2.4568944	-1.3942616	8.3794875
P	3.5675823	-2.5071977	9.3412267
C	2.9856664	-2.4172364	11.0727779
H	3.5400073	-3.1279583	11.6954364
H	3.1303029	-1.4037453	11.4622275
H	1.9190855	-2.6652231	11.1156572
C	5.2847830	-1.9113142	9.3387421
H	5.8602324	-2.4495920	10.0994815
H	5.7089850	-2.0832227	8.3484608
H	5.2909395	-0.8398522	9.5627649
C	3.4912185	-4.2662023	8.8802808
H	4.2345020	-4.8197234	9.4648777
H	2.4950859	-4.6596466	9.1079117
H	3.7005002	-4.3600147	7.8143538

3.4.4.7. **TS-cyclo-*anti Z trans-cis***
E(B3LYP-D3/6-31+G*(THF)) = -1894.468312
E(M06-2X-D3/6-311+G**) = -1894.154178
Gtot(B3LYP-D3/6-31+G*(THF)) = -1894.008319

C	-0.8958891	1.4725376	8.3217800
C	0.2519663	0.1050150	7.0668611
C	-0.5096489	1.0142114	6.1767537
O	-1.1181734	1.9481548	7.0469653
O	-0.6757391	1.0970986	4.9816100
C	-2.7616363	3.4969975	11.5757815
C	-2.9480564	3.9640507	10.2717750
C	-2.3363171	3.3159914	9.1981936
C	-1.5303053	2.1883845	9.4270847
C	-1.3439106	1.7231007	10.7411659
C	-1.9571793	2.3742284	11.8068209
H	-3.2412110	4.0039254	12.4101708
H	-3.5733163	4.8348023	10.0886235
H	-2.4838178	3.6770729	8.1846745
H	-0.7214229	0.8487617	10.9053463
H	-1.8114353	2.0075616	12.8204056
N	-0.1757975	0.4051108	8.3922796
C	0.3517299	-1.3663732	6.6775444
H	0.9169044	-1.3790397	5.7399970
C	1.1320280	-2.2018486	7.7352130
H	0.4457204	-2.5156532	8.5308324
H	1.4978703	-3.1034153	7.2339116
C	2.9245277	-0.5209138	7.2620207
H	1.8751003	-0.7236659	9.0953364
C	3.8964746	-1.1046540	6.4173981
O	4.4430080	-0.2637404	5.4792688
O	4.3537521	-2.2676999	6.5511730
C	5.3408927	-0.8664651	4.5440352
H	5.6576548	-0.0618753	3.8761592
H	6.2115444	-1.2977525	5.0494001
H	4.8443311	-1.6551424	3.9673944
C	2.1206622	0.6621850	7.0403777
H	2.0011199	1.3315988	7.8944281
C	2.2984893	1.4894350	5.7962955
O	2.0734414	0.7893181	4.6806929
O	2.5450299	2.6864418	5.8130573
C	2.1243859	1.5246564	3.4462534
H	1.8790421	0.8031007	2.6657979
H	1.3902497	2.3347393	3.4577209
H	3.1257623	1.9369776	3.2891012
C	-3.5209540	-3.1666513	5.9004320
C	-2.5597351	-3.0944469	4.8898178
C	-1.3167823	-2.5076928	5.1435704
C	-1.0123972	-1.9881688	6.4074061
C	-1.9885108	-2.0609019	7.4141273
C	-3.2311955	-2.6440836	7.1644652
H	-4.4890535	-3.6232743	5.7051630
H	-2.7773495	-3.4935504	3.9011774
H	-0.5748890	-2.4501618	4.3493365
H	-1.7739565	-1.6468350	8.3957665
H	-3.9755051	-2.6888281	7.9572579
C	2.3088674	-1.3885276	8.3362801
P	3.4357381	-2.4421824	9.3496938
C	2.7187094	-2.4970465	11.0306630
H	3.3011457	-3.1762644	11.6629114
H	2.7316171	-1.4952884	11.4734897
H	1.6847876	-2.8564140	10.9822216
C	5.0907615	-1.7083970	9.5162760
H	5.6441860	-2.2380628	10.2995596
H	5.6083008	-1.7976381	8.5596004
H	4.9907336	-0.6523968	9.7870992
C	3.5528649	-4.1768352	8.8094493
H	4.3087033	-4.6882811	9.4160880
H	2.5857770	-4.6692612	8.9544305
H	3.8351461	-4.1983046	7.7565135

3.4.4.8. **TS-cyclo-*anti Z trans-trans***
E(B3LYP-D3/6-31+G*(THF)) = -1894.471136
E(M06-2X-D3/6-311+G**) = -1894.146467
Gtot(B3LYP-D3/6-31+G*(THF)) = -1894.010287

C	-1.6802276	1.7166342	7.3153285
C	-0.2959286	0.0793769	7.5840653
C	-0.6892028	0.5224214	8.9093025
O	-1.5662656	1.5987744	8.6858102
O	-0.3790067	0.1876570	10.0447391
C	-4.2532924	4.7555547	5.7861427
C	-4.1345484	4.5721617	7.1668016
C	-3.2875937	3.5879252	7.6761303
C	-2.5489645	2.7695654	6.8025427
C	-2.6711507	2.9603301	5.4135121
C	-3.5162082	3.9462249	4.9128610
H	-4.9162745	5.5227769	5.3916664
H	-4.7066403	5.1956627	7.8507035
H	-3.1990860	3.4442855	8.7489460
H	-2.0979876	2.3277462	4.7419649
H	-3.6058707	4.0837586	3.8374307
N	-1.0006683	0.8443168	6.6388262
C	0.0084545	-1.3738018	7.2473202
H	0.4550564	-1.3880039	6.2425985
C	0.9777581	-2.0353250	8.2579129
H	0.5513511	-1.9205261	9.2574494
H	1.0133530	-3.1114418	8.0530797

C	2.4962765	0.0506548	8.3931087
H	2.8334781	-1.8284652	9.3020085
C	3.1958556	0.6040659	9.5263745
O	3.2238820	1.9690949	9.5429813
O	3.7810848	-0.0577094	10.3981742
C	3.9107950	2.5712515	10.6466955
H	3.8089610	3.6494146	10.5048342
H	3.4639255	2.2716495	11.6002230
H	4.9701697	2.2922931	10.6494775
C	1.7300298	0.7441576	7.4328348
H	1.6923816	0.3265687	6.4301690
C	1.6791229	2.2405130	7.2997692
O	0.9280185	2.8447526	8.2198115
O	2.2361191	2.8250730	6.3830864
C	0.8516112	4.2802129	8.1439311
H	0.1807689	4.5759825	8.9514952
H	1.8432755	4.7204988	8.2852604
H	0.4484219	4.5919568	7.1760682
C	-3.6815129	-3.6593660	6.9974729
C	-2.9052199	-3.4438662	5.8568291
C	-1.7156438	-2.7154734	5.9469451
C	-1.2833926	-2.1892264	7.1697474
C	-2.0736580	-2.4079650	8.3090135
C	-3.2599387	-3.1380371	8.2245679
H	-4.6096237	-4.2237290	6.9315731
H	-3.2274721	-3.8378288	4.8948166
H	-1.1242289	-2.5406303	5.0496680
H	-1.7668745	-1.9997456	9.2682970
H	-3.8608407	-3.2943562	9.1184537
C	2.4093646	-1.4573008	8.3596344
P	3.6327685	-2.1559968	7.1492005
C	3.4709027	-3.9688634	7.0053557
H	4.3273699	-4.3725576	6.4533867
H	3.4419188	-4.4145253	8.0062363
H	2.5513566	-4.2268013	6.4701234
C	5.2894057	-1.7824748	7.8023331
H	6.0534932	-2.1709176	7.1203542
H	5.3976674	-0.6982751	7.9004003
H	5.4049582	-2.2415780	8.7893420
C	3.5457279	-1.4523668	5.4693253
H	4.3454636	-1.8937672	4.8631641
H	2.5815531	-1.6713630	5.0010104
H	3.6928604	-0.3695450	5.5168990

3.4.4.9. **TS-cyclo-*syn E cis-cis***
E(B3LYP-D3/6-31+G*(THF)) = -1894.481668
E(M06-2X-D3/6-311+G**) = -1894.163098
Gtot(B3LYP-D3/6-31+G*(THF)) = -1894.02062

C	0.4473144	-2.5944883	3.5413250
C	0.7769044	-1.6620495	5.4671915
C	0.5313678	-0.5875855	4.4987140
O	0.4324729	-1.2412418	3.2527969
O	0.4526320	0.6209187	4.5896437
C	-0.0243000	-5.3608889	0.3372470
C	-0.0143126	-3.9885560	0.0721503
C	0.1418833	-3.0721244	1.1120509
C	0.2884723	-3.5267585	2.4327123
C	0.2750280	-4.9092357	2.6962459
C	0.1205997	-5.8173725	1.6534658
H	-0.1463318	-6.0727011	-0.4761850
H	-0.1291835	-3.6295110	-0.9482613
H	0.1498568	-2.0058428	0.9063313
H	0.3806020	-5.2514365	3.7219896
H	0.1099753	-6.8845629	1.8649415
N	0.5918145	-2.8846825	4.7912455
C	0.2404999	-1.5313536	6.8866946
C	1.0731689	-2.3789128	7.8801333
H	0.4751068	-2.5865181	8.7743371
H	1.3128719	-3.3387857	7.4166854
C	2.9471389	-0.8632345	7.0925968
H	2.0467811	-0.8428578	9.0091139
C	3.4542674	0.4667008	7.2398022
O	3.5231804	0.8556853	8.5732379
O	3.8575603	1.2273215	6.3493800
C	3.9992230	2.1879868	8.7987328
H	3.9855347	2.3298113	9.8821272
H	5.0166228	2.3157056	8.4143459
H	3.3490201	2.9260505	8.3161091
C	2.7891679	-1.3749784	5.7756871
H	3.0493241	-0.6704917	4.9888295
C	3.2442757	-2.7552103	5.4497228
O	3.3924401	-2.9233930	4.1239774
O	3.4972542	-3.6398566	6.2590040
C	3.7457655	-4.2529939	3.6893897
H	3.8044723	-4.1994181	2.6020261
H	4.7089153	-4.5510731	4.1126506
H	2.9742762	-4.9628676	3.9967758
H	0.3529946	-0.4739487	7.1540244
C	2.3466751	-1.5934825	8.2690940
P	3.5529930	-2.6245643	9.2680567
C	3.4704097	-2.0289893	10.9918558
H	4.1786802	-2.5746035	11.6242352
H	3.7091141	-0.9606483	11.0081790
H	2.4554220	-2.1721660	11.3797759
C	5.2556291	-2.3824648	8.6867526
H	5.9482720	-2.9094855	9.3517139
H	5.3387303	-2.7695426	7.6690005
H	5.4790120	-1.3115936	8.6888093
C	3.1708889	-4.4074444	9.3083991
H	3.8992488	-4.9163222	9.9499626
H	2.1671661	-4.5590852	9.7188870
H	3.2180369	-4.8119739	8.2954771
C	-3.9950460	-2.4669202	7.0288011
C	-3.5627814	-1.1419767	7.1125485
C	-2.1974600	-0.8453487	7.0660368
C	-1.2452875	-1.8641966	6.9351347
C	-1.6916791	-3.1915588	6.8428864
C	-3.0532804	-3.4912662	6.8920631
H	-5.0568634	-2.7008253	7.0675968
H	-4.2877556	-0.3371505	7.2164154
H	-1.8675520	0.1900399	7.1288907
H	-0.9677107	-3.9906956	6.7147371
H	-3.3807811	-4.5264467	6.8192801

3.4.4.10. **TS-cyclo-*syn E cis-trans***
E(B3LYP-D3/6-31+G*(THF)) = -1894.476041
E(M06-2X-D3/6-311+G**) = -1894.154592
Gtot(B3LYP-D3/6-31+G*(THF)) = -1894.015212

C	0.4945569	-1.1506160	3.4802383
C	0.9000794	-1.3626823	5.5967463
C	1.3507532	-2.5907889	4.9398393
O	1.1642502	-2.3613654	3.5681974
O	1.8489281	-3.6249521	5.3500130
C	-0.5840282	0.2648440	-0.3909199
C	0.2494747	-0.8504074	-0.2676914
C	0.6084754	-1.3243982	0.9941989
C	0.1286061	-0.6827430	2.1488232
C	-0.7073780	0.4413929	2.0206440
C	-1.0597459	0.9086953	0.7581196
H	-0.8627601	0.6316553	-1.3763550
H	0.6236798	-1.3523144	-1.1572448
H	1.2579950	-2.1896367	1.0901486
H	-1.0733702	0.9300902	2.9187986
H	-1.7104097	1.7755503	0.6663252
N	0.2772687	-0.5677720	4.6125213
C	0.2690783	-1.3825788	6.9889274
C	1.1425756	-2.0891462	8.0633746
H	0.5491240	-2.1126815	8.9876821
H	1.3449226	-3.1214880	7.7664069
C	3.3179628	-1.3244401	7.0054854
H	2.2357223	-0.2968294	8.4971274
C	4.2437656	-2.3388178	6.6015566
O	4.4637071	-3.2913915	7.5881820
O	4.8705583	-2.3963553	5.5353064
C	5.3660675	-4.3475785	7.2363661
H	5.4644060	-4.9683705	8.1304439
H	4.9606709	-4.9403486	6.4098187
H	6.3434718	-3.9523628	6.9432884
C	2.7528161	-0.5593770	5.9471883
H	3.2280166	-0.7100564	4.9814120
C	2.2898109	0.8530898	6.1080117
O	2.3600391	1.5068252	4.9344423
O	1.8852307	1.3854373	7.1355891
C	1.7723158	2.8231142	4.9051664
H	1.9559356	3.2044355	3.8994854
H	0.6981498	2.7527009	5.0992623
H	2.2370814	3.4736818	5.6514631
H	0.1611649	-0.3348191	7.2897871
C	2.4883607	-1.3432079	8.2782501
P	3.2761621	-1.8403827	9.8664296
C	2.4076718	-0.9102700	11.1773671
H	2.8019688	-1.1953735	12.1590830
H	2.5523300	0.1652067	11.0297516
H	1.3362323	-1.1340126	11.1469377
C	5.0213020	-1.3356876	9.9485732
H	5.4088533	-1.5205157	10.9567877
H	5.5977443	-1.8998952	9.2146860
H	5.0895078	-0.2657304	9.7241282
C	3.0611083	-3.5978875	10.2920104
H	3.6002791	-3.8146996	11.2208457
H	1.9951604	-3.7955772	10.4460180
H	3.4345892	-4.2239124	9.4819935
C	-3.7366030	-3.0555346	6.8745699
C	-3.5405449	-1.7034131	7.1642625
C	-2.2473893	-1.1756446	7.1989452
C	-1.1323745	-1.9834447	6.9439009
C	-1.3402561	-3.3405921	6.6528569
C	-2.6309449	-3.8717775	6.6175338
H	-4.7422496	-3.4701653	6.8451891
H	-4.3939708	-1.0572999	7.3608899
H	-2.1023577	-0.1192107	7.4177488
H	-0.4899172	-3.9827554	6.4387801
H	-2.7743038	-4.9253041	6.3854973

3.4.4.11. **TS-cyclo-*syn E trans-cis***
E(B3LYP-D3/6-31+G*(THF)) = -1894.475684
E(M06-2X-D3/6-311+G**) = -1894.153886
Gtot(B3LYP-D3/6-31+G*(THF)) = -1894.016079

C	0.8878143	-2.5665411	3.8300892
C	0.7760164	-1.3991385	5.6530785
C	0.0727269	-0.6487617	4.6111967
O	0.2540091	-1.4046012	3.4306532
O	-0.5346764	0.4061920	4.5933693
C	1.6614454	-5.6082604	0.9500403
C	0.8687070	-4.5112915	0.5997201
C	0.6162274	-3.5004061	1.5274993
C	1.1559555	-3.5853564	2.8215156
C	1.9604146	-4.6866711	3.1679328
C	2.2083499	-5.6903760	2.2364977
H	1.8559001	-6.3948697	0.2242665
H	0.4441580	-4.4436715	-0.3993358
H	-0.0027287	-2.6497593	1.2575332
H	2.3867370	-4.7283439	4.1655843
H	2.8321235	-6.5387181	2.5095045
N	1.1703981	-2.6302409	5.0876229
C	0.2000710	-1.4094328	7.0690276
C	1.1385992	-2.0849414	8.1048272
H	0.5900656	-2.1271229	9.0559269
H	1.3557277	-3.1128824	7.7983007
C	3.2201825	-1.2770076	6.9286852
H	2.2054190	-0.2623836	8.4772945
C	4.0736187	-2.3299575	6.4668058
O	4.3161832	-3.2875414	7.4470689
O	4.6171030	-2.4324256	5.3613337
C	5.2033442	-4.3495411	7.0721269
H	5.3073276	-4.9803074	7.9586666
H	4.7858436	-4.9320010	6.2447935
H	6.1809475	-3.9604244	6.7704775
C	2.6045722	-0.4971262	5.9118013
H	3.0066371	-0.6562237	4.9156755
C	2.1459478	0.9089423	6.1137148
O	2.1191439	1.5627703	4.9373190
O	1.8154456	1.4365414	7.1696927
C	1.5246958	2.8779307	4.9534886
H	1.5679718	3.2301443	3.9219575
H	0.4886289	2.8079349	5.2909139
H	2.0877523	3.5464370	5.6106643
H	0.0680361	-0.3626248	7.3641158
C	2.4702486	-1.3028452	8.2494653
P	3.3629895	-1.7794790	9.7902322
C	2.6064537	-0.8068421	11.1385802
H	3.0528778	-1.0889787	12.0985246
H	2.7712322	0.2620742	10.9657035
H	1.5289178	-0.9997602	11.1777369
C	5.1202417	-1.3160468	9.7388972
H	5.5653840	-1.4791715	10.7263587
H	5.6347340	-1.9199673	8.9909131

H	5.2020500	-0.2571323	9.4725800
C	3.1537106	-3.5240500	10.2728145
H	3.7772789	-3.7317578	11.1495071
H	2.1047058	-3.6991676	10.5329518
H	3.4383969	-4.1752800	9.4467389
C	-3.7473381	-3.2174024	7.0415209
C	-3.5953563	-1.8523778	7.2952938
C	-2.3205950	-1.2799686	7.3092221
C	-1.1802608	-2.0576138	7.0698932
C	-1.3451494	-3.4270939	6.8091691
C	-2.6167015	-4.0025109	6.7972902
H	-4.7385625	-3.6659254	7.0287443
H	-4.4691839	-1.2306180	7.4795601
H	-2.2093297	-0.2135610	7.4967349
H	-0.4743676	-4.0396508	6.5953424
H	-2.7263155	-5.0653621	6.5906211

3.4.4.12. **TS-cyclo-*syn E trans-trans***
E(B3LYP-D3/6-31+G*(THF)) = -1894.475852
E(M06-2X-D3/6-311+G**) = -1894.158318
Gtot(B3LYP-D3/6-31+G*(THF)) = -1894.018002

C	0.2044505	-1.1506981	3.4539369
C	0.7516445	-1.7091897	5.4757598
C	0.5456540	-2.9455974	4.7202815
O	0.2712401	-2.5332991	3.4037819
O	0.6020582	-4.1322990	5.0064042
C	-0.8814558	1.0049882	-0.0586390
C	-0.8131665	-0.3904623	-0.0970149
C	-0.4493166	-1.1098501	1.0419806
C	-0.1497783	-0.4322305	2.2358733
C	-0.2183448	0.9739329	2.2693307
C	-0.5820016	1.6835740	1.1294726
H	-1.1669100	1.5626308	-0.9479197
H	-1.0459775	-0.9214315	-1.0173806
H	-0.3996926	-2.1943697	1.0133313
H	0.0131970	1.4895427	3.1968471
H	-0.6345117	2.7695287	1.1656997
N	0.4366996	-0.6396650	4.6183694
C	0.2888572	-1.5925959	6.9246130
C	1.1420738	-2.4469308	7.8945140
H	0.5630418	-2.6554057	8.8013932
H	1.3789299	-3.4050509	7.4290472
C	3.0153002	-0.9271023	7.0885742
H	2.1117505	-0.8923608	8.9976652
C	3.4078993	0.4538900	7.2069263
O	3.4110849	0.8888893	8.5217111
O	3.7689076	1.2068534	6.2973612
C	3.7602198	2.2657838	8.7129955
H	3.6974067	2.4420147	9.7895268
H	4.7738373	2.4695433	8.3542491
H	3.0632574	2.9232027	8.1835194
C	2.8916049	-1.4643529	5.7934517
H	3.0819255	-0.7756868	4.9760281
C	3.2928492	-2.8590550	5.4874250
O	3.3587203	-3.0698447	4.1596041
O	3.5690254	-3.7264943	6.3088285
C	3.6297447	-4.4248409	3.7425735
H	3.6460769	-4.3959733	2.6521979
H	4.5953802	-4.7594669	4.1317670
H	2.8339130	-5.0821032	4.0979630
H	0.4345457	-0.5364654	7.1844140
C	2.4182170	-1.6552093	8.2739803
P	3.6345166	-2.6523222	9.2850091
C	3.5521024	-2.0158822	10.9935772
H	4.2582171	-2.5500296	11.6380320
H	3.7952871	-0.9484781	10.9863467
H	2.5366834	-2.1467147	11.3842457
C	5.3333899	-2.4024367	8.6944762
H	6.0360382	-2.9135576	9.3619557
H	5.4166401	-2.8009081	7.6816896
H	5.5473521	-1.3297802	8.6826693
C	3.2615639	-4.4336553	9.3650483
H	3.9993939	-4.9258112	10.0085386
H	2.2629260	-4.5808177	9.7887908
H	3.3014823	-4.8563989	8.3600989
C	-3.9783420	-2.3860291	6.9950777
C	-3.4985169	-1.0875335	6.8025014
C	-2.1244404	-0.8412650	6.8024375
C	-1.2060223	-1.8834268	6.9973533
C	-1.6976360	-3.1845019	7.1798808
C	-3.0723126	-3.4332413	7.1825612
H	-5.0489164	-2.5807578	6.9947558
H	-4.1951208	-0.2652108	6.6506385
H	-1.7542095	0.1682289	6.6368374
H	-1.0031382	-4.0114390	7.2978821
H	-3.4356487	-4.4492819	7.3238334

3.4.4.13. **TS-cyclo-*syn Z cis-cis***
E(B3LYP-D3/6-31+G*(THF)) = -1894.466650
E(M06-2X-D3/6-311+G**) = -1894.145365
Gtot(B3LYP-D3/6-31+G*(THF)) = -1894.007495

C	0.7621194	-2.5477404	3.6555353
C	0.7950904	-1.5071601	5.5462462
C	0.2250547	-0.5921611	4.5695722
O	0.3021207	-1.2804782	3.3404269
O	-0.1988738	0.5524780	4.6350105
C	1.0207349	-5.4581473	0.5543640
C	0.5218833	-4.1839952	0.2693027
C	0.4402932	-3.2186669	1.2732279
C	0.8597070	-3.5229605	2.5790211
C	1.3611477	-4.8069179	2.8613295
C	1.4401573	-5.7645286	1.8550468
H	1.0835723	-6.2086862	-0.2306244
H	0.1938997	-3.9407129	-0.7390026
H	0.0486892	-2.2303346	1.0522218
H	1.6872795	-5.0308466	3.8725070
H	1.8316993	-6.7539153	2.0819160
N	1.0242516	-2.7293688	4.9105530
C	0.2573423	-1.5017512	6.9714156
C	1.2289838	-2.1154228	8.0104952
H	0.6845419	-2.1761417	8.9621496
H	1.5059803	-3.1331671	7.7173078
C	3.3632295	-1.2722769	6.9037009
H	2.2068065	-0.2336120	8.3620364
C	4.4231593	-2.2316547	6.8003020
O	5.2783261	-2.0263219	5.7655977
O	4.6354722	-3.1331742	7.6356434
C	6.2296100	-3.0686624	5.5162262

H	6.7953546	-2.7492849	4.6384136
H	6.9027635	-3.2009172	6.3698155
H	5.7243840	-4.0184828	5.3117830
C	2.7483295	-0.5630090	5.8565766
H	2.2444283	0.3663919	6.1238152
C	3.1649340	-0.5677398	4.4217226
O	3.0507615	0.6887095	3.9085769
O	3.4623140	-1.5305057	3.7430340
C	3.1763887	0.7739062	2.4752515
H	3.0302841	1.8264692	2.2264124
H	4.1653031	0.4376713	2.1499973
H	2.4099734	0.1564347	1.9979460
H	0.1036079	-0.4478979	7.2401135
C	2.5225151	-1.2736373	8.1715056
P	3.3651431	-1.6956278	9.7641659
C	2.4754987	-0.7342980	11.0394843
H	2.8721582	-0.9778751	12.0314277
H	2.6016493	0.3374817	10.8524066
H	1.4082544	-0.9784083	11.0147837
C	5.0999568	-1.1328446	9.8004950
H	5.3237213	-0.6869901	10.7746201
H	5.7644491	-1.9750427	9.5997716
H	5.2346198	-0.3807875	9.0157623
C	3.2033924	-3.4360803	10.2760823
H	3.8188514	-3.5969119	11.1683879
H	2.1578287	-3.6467526	10.5225269
H	3.5413417	-4.0821683	9.4661714
C	-3.6162591	-3.4640559	7.0925313
C	-3.5193544	-2.0812960	7.2627665
C	-2.2694154	-1.4560596	7.2266781
C	-1.0996553	-2.1987873	7.0225180
C	-1.2093605	-3.5870045	6.8448923
C	-2.4552303	-4.2139176	6.8814455
H	-4.5873468	-3.9541121	7.1192550
H	-4.4168140	-1.4867653	7.4216150
H	-2.2029265	-0.3766456	7.3512437
H	-0.3178268	-4.1733694	6.6472546
H	-2.5206660	-5.2908524	6.7385721

3.4.4.14. **TS-cyclo-*syn Z cis-trans***
E(B3LYP-D3/6-31+G*(THF)) = -1894.469052
E(M06-2X-D3/6-311+G**) = -1894.146615
Gtot(B3LYP-D3/6-31+G*(THF)) = -1894.00763

C	0.1936830	-1.3261349	3.4506188
C	0.8349161	-1.4624192	5.5220615
C	0.8761810	-2.8119618	4.9486895
O	0.5069283	-2.6700445	3.6095662
O	1.2360444	-3.8989468	5.3858175
C	-1.3001238	-0.0008604	-0.3140799
C	-1.0368417	-1.3588220	-0.1127604
C	-0.5356761	-1.8058604	1.1101393
C	-0.2947323	-0.8897081	2.1479761
C	-0.5609279	0.4772011	1.9407342
C	-1.0591332	0.9148137	0.7173020
H	-1.6929387	0.3433432	-1.2682926
H	-1.2250664	-2.0734934	-0.9107591
H	-0.3338471	-2.8615193	1.2661509
H	-0.3844801	1.1761147	2.7531375
H	-1.2650997	1.9722193	0.5668174
N	0.3403123	-0.6100000	4.5138703
C	0.2888234	-1.1933305	6.9177920
C	1.0105471	-2.0164090	8.0073816
H	0.4025670	-2.0196748	8.9174874
H	1.0859286	-3.0536728	7.6683670
C	3.0544723	-0.6372346	7.2164758
H	2.2832485	-0.7095594	9.1803637
C	3.7490611	0.5541249	7.6284335
O	4.5830411	1.0747245	6.6794466
O	3.6992545	1.0517416	8.7670409
C	5.1716498	2.3424844	6.9915064
H	5.7724597	2.6117982	6.1197366
H	4.4030795	3.1040462	7.1647592
H	5.8076420	2.2763522	7.8804882
C	2.9287559	-1.1816181	5.9133889
H	3.0876270	-2.2510896	5.7974665
C	3.2259127	-0.4848513	4.6269839
O	2.8518597	0.7991072	4.6091741
O	3.6647709	-1.0742103	3.6467003
C	2.9693778	1.4707774	3.3434360
H	2.6643556	2.5018995	3.5292397
H	4.0003990	1.4379260	2.9786591
H	2.3085761	1.0050168	2.6065286
H	0.4942495	-0.1301376	7.1044817
C	2.3998417	-1.4094108	8.3442610
P	3.5330890	-2.6950296	9.0451919
C	2.7258194	-3.5552014	10.4371039
H	3.4120279	-4.2804951	10.8895162
H	2.4228428	-2.8213625	11.1919790
H	1.8374227	-4.0818303	10.0729812
C	5.0561184	-1.8975111	9.6363497
H	5.7206102	-2.6473659	10.0786202
H	5.5561700	-1.4274710	8.7839002
H	4.8088578	-1.1273729	10.3727515
C	3.9944815	-3.9407092	7.7986133
H	4.5157636	-4.7683508	8.2920897
H	3.1035589	-4.3180878	7.2872575
H	4.6652607	-3.4861877	7.0628103
C	-4.0220331	-1.6906359	6.8786583
C	-3.4549902	-0.4139940	6.8633909
C	-2.0667922	-0.2632494	6.8904012
C	-1.2234201	-1.3804297	6.9379566
C	-1.8021822	-2.6583264	6.9445877
C	-3.1897654	-2.8129495	6.9176419
H	-5.1032431	-1.8105979	6.8562347
H	-4.0934927	0.4665381	6.8271787
H	-1.6304201	0.7331675	6.8637115
H	-1.1661507	-3.5404559	6.9539908
H	-3.6219189	-3.8116220	6.9215142

3.4.4.15. **TS-cyclo-*syn Z trans-cis***
E(B3LYP-D3/6-31+G*(THF)) = -1894.467339
E(M06-2X-D3/6-311+G**) = -1894.143194
Gtot(B3LYP-D3/6-31+G*(THF)) = -1894.008143

C	0.2857929	-2.3805480	3.5872554
C	0.6179046	-1.5003740	5.5334796
C	0.2696906	-0.4154145	4.6374151
O	0.1379745	-1.0262094	3.3661165
O	0.0881085	0.7836007	4.7829043

C	-0.1446739	-5.0617972	0.2962407
C	-0.3353119	-3.6904292	0.1057651
C	-0.1877920	-2.8025413	1.1717714
C	0.1522187	-3.2811587	2.4489310
C	0.3446292	-4.6637332	2.6342226
C	0.1964813	-5.5434101	1.5664793
H	-0.2609062	-5.7517267	-0.5367219
H	-0.6025485	-3.3097294	-0.8776342
H	-0.3393138	-1.7377435	1.0228687
H	0.6060976	-5.0281347	3.6234213
H	0.3460107	-6.6098168	1.7223968
N	0.5103930	-2.7051615	4.8225480
C	0.1863486	-1.4087538	6.9898548
C	1.0663934	-2.2316369	7.9658368
H	0.5031691	-2.4146130	8.8867790
H	1.2752710	-3.2135702	7.5239725
C	3.0232542	-0.8358359	7.0949821
H	2.1172293	-0.6585575	9.0214299
C	3.5543338	0.4965886	7.2730502
O	4.1277642	1.0068217	6.1483424
O	3.5504803	1.1255745	8.3406772
C	4.6621590	2.3334753	6.2547612
H	5.0510772	2.5748711	5.2635002
H	3.8823888	3.0481109	6.5368860
H	5.4661202	2.3721392	6.9972529
C	2.8040211	-1.4939757	5.8801783
H	2.6787057	-2.5714617	5.9297518
C	3.5153854	-1.1770852	4.5934179
O	2.9426447	-0.2348148	3.8497732
O	4.5143872	-1.7984119	4.2623776
C	3.5865304	0.0760871	2.5981697
H	2.9535079	0.8286152	2.1267605
H	4.5910864	0.4708790	2.7765079
H	3.6538488	-0.8174999	1.9705561
H	0.2956773	-0.3499664	7.2568798
C	2.3605758	-1.4574507	8.3097490
P	3.5728075	-2.5118700	9.2329593
C	2.7915991	-3.2953469	10.6823763
H	3.5401951	-3.8516185	11.2572981
H	2.3483014	-2.5215816	11.3190360
H	2.0070463	-3.9849937	10.3539094
C	4.9621514	-1.4835706	9.7934706
H	5.6790985	-2.1017178	10.3441161
H	5.4538788	-1.0426846	8.9218807
H	4.5864779	-0.6798318	10.4333841
C	4.2278673	-3.8208238	8.1497142
H	4.9885943	-4.3968572	8.6878193
H	3.4229527	-4.4965300	7.8419828
H	4.6777669	-3.3612127	7.2641340
C	-4.0288097	-2.4013217	7.2753043
C	-3.6113965	-1.0700586	7.3416207
C	-2.2520665	-0.7559642	7.2607275
C	-1.2904860	-1.7637458	7.1129999
C	-1.7215049	-3.0968367	7.0347715
C	-3.0777211	-3.4140740	7.1191458
H	-5.0864453	-2.6485044	7.3392102
H	-4.3441273	-0.2737680	7.4560087
H	-1.9325949	0.2836774	7.3012098
H	-0.9918933	-3.8872551	6.8822511
H	-3.3941037	-4.4534768	7.0551853

3.4.4.16. **TS-cyclo-*syn Z trans-trans***
E(B3LYP-D3/6-31+G*(THF)) = -1894.471923
E(M06-2X-D3/6-311+G**) = -1894.143511
Gtot(B3LYP-D3/6-31+G*(THF)) = -1894.012007

C	0.2755336	-1.4135269	3.4248709
C	0.7748444	-1.5322770	5.5235469
C	1.1940787	-2.7728258	4.9251321
O	0.8831963	-2.6447740	3.5578107
O	1.7510923	-3.7824841	5.3558748
C	-1.0454904	-0.2359856	-0.4567746
C	-0.4183681	-1.4719678	-0.2772389
C	0.0241219	-1.8612591	0.9866239
C	-0.1587506	-1.0134225	2.0951392
C	-0.7890551	0.2318468	1.9064530
C	-1.2271970	0.6131775	0.6420606
H	-1.3914907	0.0641852	-1.4433567
H	-0.2735194	-2.1377431	-1.1254780
H	0.5092960	-2.8228717	1.1244067
H	-0.9309249	0.8851998	2.7626217
H	-1.7155302	1.5764548	0.5102375
N	0.1612275	-0.7519176	4.5400705
C	0.2604016	-1.4455292	6.9526241
C	1.2101948	-2.0713390	8.0118206
H	0.6603123	-2.0847753	8.9621755
H	1.4356993	-3.1055264	7.7386782
C	3.3873168	-1.3246754	6.9123603
H	2.2855852	-0.2440995	8.3735298
C	4.3860877	-2.3589633	6.7624498
O	5.2122915	-2.1935680	5.7047489
O	4.5632892	-3.2703645	7.5865197
C	6.1581204	-3.2481674	5.4698021
H	6.7328673	-2.9373831	4.5952155
H	6.8211142	-3.3789349	6.3307428
H	5.6424626	-4.1925547	5.2672030
C	2.8604488	-0.5050165	5.9278025
H	2.2898052	0.3582023	6.2621187
C	3.2845911	-0.2737384	4.5155888
O	3.4644609	-1.3758745	3.7884250
O	3.4047568	0.8594043	4.0694877
C	3.9040390	-1.1811856	2.4314031
H	4.0013354	-2.1833859	2.0126867
H	3.1651217	-0.6024264	1.8698740
H	4.8656354	-0.6594237	2.4131047
H	0.1410985	-0.3771704	7.1860435
C	2.5448498	-1.2977450	8.1817877
P	3.3524529	-1.7601258	9.7825705
C	2.4946712	-0.7654151	11.0531161
H	2.8677789	-1.0354828	12.0472958
H	2.6753582	0.3000647	10.8753273
H	1.4170881	-0.9568783	11.0162313
C	5.1044713	-1.2690097	9.8447630
H	5.4135891	-1.1567981	10.8895175
H	5.7129495	-2.0251310	9.3464217
H	5.2204561	-0.3090287	9.3297396
C	3.1252400	-3.4991842	10.2719775
H	3.7408667	-3.6977631	11.1564981
H	2.0737897	-3.6738661	10.5219489
H	3.4328975	-4.1477186	9.4513989

C	-3.7031738	-3.2112010	7.2136490
C	-3.5294913	-1.8364794	7.3865141
C	-2.2513906	-1.2758087	7.3074175
C	-1.1297758	-2.0745034	7.0540030
C	-1.3156512	-3.4556686	6.8809081
C	-2.5900081	-4.0185127	6.9603357
H	-4.6968946	-3.6505069	7.2713025
H	-4.3893480	-1.1975813	7.5784252
H	-2.1252724	-0.2016062	7.4329935
H	-0.4617458	-4.0899967	6.6605475
H	-2.7149005	-5.0903550	6.8191067

3.4.4.17. **int3 *anti cis-cis* chair**
E(B3LYP-D3/6-31+G*(THF)) = -1894.489938
E(M06-2X-D3/6-311+G**) = -1894.177987
Gtot(B3LYP-D3/6-31+G*(THF)) = -1894.029778

C	0.4750419	0.5439785	7.6775108
C	-0.4704922	0.7038328	8.8701739
O	-1.4892962	1.5427543	8.4420168
O	-0.4880273	0.2081125	9.9663877
C	-3.9743344	4.3848867	5.0953123
C	-4.2332281	3.9892160	6.4101016
C	-3.3196206	3.1862372	7.0937344
C	-2.1389541	2.7742025	6.4570234
C	-1.8810148	3.1731969	5.1345883
C	-2.7955878	3.9761978	4.4599948
H	-4.6892546	5.0098206	4.5644886
H	-5.1490830	4.3045105	6.9044058
H	-3.5219321	2.8740527	8.1137237
H	-0.9654880	2.8447373	4.6512092
H	-2.5928811	4.2832920	3.4366182
N	-0.1020211	1.4087606	6.6513902
C	0.4702178	-0.9432232	7.1681746
H	1.1215113	-0.9230238	6.2831673
C	1.0885925	-1.8756589	8.2209609
H	0.4674737	-1.8722651	9.1200068
H	1.0798032	-2.9033998	7.8369940
C	2.6575080	0.0217768	8.9229306
H	2.7356975	-1.9978451	9.5923978
C	3.3608426	0.5200792	10.0300502
O	3.8773388	-0.4995670	10.8532635
O	3.5813497	1.7096446	10.3415885
C	4.4917840	-0.0477652	12.0574721
H	4.8511359	-0.9466679	12.5669169
H	5.3320092	0.6261635	11.8567444
H	3.7767471	0.4792329	12.7006446
C	1.9724416	0.9480387	7.9460666
H	2.4103677	0.8919068	6.9382974
C	2.1025529	2.4274888	8.3028321
O	1.1724508	2.8060700	9.1963737
O	2.8921974	3.1904312	7.7782849
C	1.2939727	4.1417377	9.7146669
H	0.4626068	4.2659488	10.4106496
H	2.2495217	4.2398215	10.2369112
H	1.2331712	4.8778304	8.9076612
C	-3.4522193	-2.1302095	5.7392445
C	-2.4818010	-1.6547680	4.8535357
C	-1.2228646	-1.2874932	5.3291642
C	-0.9051242	-1.3884472	6.6915640
C	-1.8885548	-1.8636725	7.5715385
C	-3.1512504	-2.2314008	7.0988217
H	-4.4352031	-2.4176162	5.3720589
H	-2.7059827	-1.5672498	3.7923867
H	-0.4765717	-0.9024151	4.6374578
H	-1.6809096	-1.9408822	8.6351218
H	-3.9010987	-2.5969359	7.7972832
C	2.4976633	-1.4432986	8.6781018
P	3.8713856	-2.0772523	7.5431075
C	3.5971341	-3.7691660	6.8922239
H	4.5025397	-4.1331128	6.3927431
H	3.3496925	-4.4418678	7.7211790
H	2.7701616	-3.7700509	6.1744405
C	5.3885265	-2.1304199	8.5459051
H	6.2471996	-2.3754181	7.9123995
H	5.5299979	-1.1548845	9.0172520
H	5.2779336	-2.8860812	9.3302705
C	4.2353578	-0.9968205	6.1180496
H	5.1277382	-1.3765257	5.6075273
H	3.4025943	-0.9754454	5.4091312
H	4.4359059	0.0146171	6.4828407

3.4.4.18. **int3 *anti cis-cis* boat**
E(B3LYP-D3/6-31+G*(THF)) = -1894.491766
E(M06-2X-D3/6-311+G**) = -1894.186982
Gtot(B3LYP-D3/6-31+G*(THF)) = -1894.031034

C	-0.9978290	1.5707998	8.2468933
C	0.5219247	0.3522706	7.2098151
C	-0.2980269	1.1296406	6.1891422
O	-1.2404082	1.8478412	6.9028873
O	-0.2287156	1.1777265	4.9880323
C	-3.6479338	3.3252980	11.0822019
C	-3.8578949	3.5383855	9.7173668
C	-2.9895890	2.9809749	8.7783541
C	-1.9039608	2.2015795	9.2079450
C	-1.6938325	1.9909297	10.5814954
C	-2.5629864	2.5519850	11.5121051
H	-4.3284800	3.7595371	11.8112917
H	-4.7010430	4.1376872	9.3820372
H	-3.1554798	3.1416233	7.7174637
H	-0.8509443	1.3851457	10.9005111
H	-2.3982696	2.3858878	12.5741244
N	-0.0286856	0.7735683	8.4955120
C	0.3150699	-1.1864608	7.0021451
H	0.7992454	-1.3904974	6.0418067
C	1.0630545	-2.0076108	8.0874824
H	0.4304597	-2.1413696	8.9715765
H	1.2458543	-3.0006318	7.6613296
C	2.9391804	-0.5169038	7.3028712
H	2.1555075	-0.5723180	9.2600952
C	3.7326339	-1.1188435	6.3334686
O	3.9544112	-0.3221412	5.2037022
O	4.2982413	-2.2493572	6.3986096
C	4.6700905	-0.9479255	4.1428498
H	4.7435226	-0.2001875	3.3477255
H	5.6749075	-1.2543603	4.4557834
H	4.1440815	-1.8335341	3.7646789
C	2.0881977	0.6816683	7.0367763
H	2.2103673	1.0031410	5.9986469

C	2.3928260	1.8911239	7.9239653
O	1.7211756	2.9786112	7.4800769
O	3.1163395	1.9108115	8.9008184
C	1.8251086	4.1710639	8.2859583
H	1.2309782	4.9291393	7.7726658
H	1.4258019	3.9840376	9.2872820
H	2.8680788	4.4908548	8.3647208
C	-3.8724709	-2.2473018	6.5872143
C	-3.0271513	-2.2358864	5.4754053
C	-1.6796514	-1.8994516	5.6240168
C	-1.1506190	-1.5718094	6.8809718
C	-2.0121373	-1.5855819	7.9898946
C	-3.3595610	-1.9191107	7.8448279
H	-4.9225295	-2.5092619	6.4751771
H	-3.4155858	-2.4888224	4.4910290
H	-1.0286376	-1.8856588	4.7520959
H	-1.6321889	-1.3199077	8.9717518
H	-4.0106311	-1.9212109	8.7165144
C	2.3950905	-1.3000577	8.4700061
P	3.5875015	-2.3888943	9.3428643
C	3.0525045	-2.4731854	11.0901509
H	3.6967392	-3.1599301	11.6506723
H	3.1072163	-1.4781142	11.5446358
H	2.0193966	-2.8341113	11.1425674
C	5.2393111	-1.6373014	9.3150671
H	5.8806265	-2.1099780	10.0661578
H	5.6481118	-1.7700184	8.3110264
H	5.1332093	-0.5685509	9.5266263
C	3.6245986	-4.1089743	8.7504610
H	4.4330889	-4.6479980	9.2569767
H	2.6713464	-4.5953672	8.9824329
H	3.7938019	-4.0918217	7.6725955

3.4.4.19. **int3** ***anti cis-cis*** **TS**$_{\text{boat-chair}}$
E(B3LYP-D3/6-31+G*(THF)) = -1894.480540
E(M06-2X-D3/6-311+G**) = -1894.175856
Gtot(B3LYP-D3/6-31+G*(THF)) = -1894.021110

C	-1.0102802	2.1218481	8.4308909
C	0.6005358	0.9375093	7.4892200
C	-0.1473682	1.7344232	6.4239180
O	-1.1369658	2.4448953	7.0793638
O	0.0039394	1.7982603	5.2319572
C	-3.8513609	3.8236299	11.1098683
C	-3.9529808	4.0841749	9.7402660
C	-3.0244523	3.5405422	8.8514207
C	-1.9876046	2.7287376	9.3367752
C	-1.8879771	2.4674232	10.7146566
C	-2.8162253	3.0146596	11.5951014
H	-4.5768385	4.2495127	11.7998736
H	-4.7569686	4.7115503	9.3623363
H	-3.1036577	3.7410941	7.7869384
H	-1.0829632	1.8343014	11.0760131
H	-2.7360115	2.8116091	12.6607848
N	-0.0730928	1.3089009	8.7335506
C	0.4532008	-0.5920930	7.2309093
H	0.9664501	-0.7769822	6.2800420
C	1.1970244	-1.3321978	8.3525357
H	0.7999600	-1.0075672	9.3169648
H	0.9525267	-2.3940965	8.2682476
C	3.0784786	0.1313191	7.4606233
H	3.0463211	-0.8893779	9.3901664
C	4.2778960	0.2174856	6.7712312
O	4.4886777	1.4447359	6.1376493
O	5.1684907	-0.6789613	6.6532297
C	5.5342450	1.4662010	5.1713639
H	5.5727671	2.4934484	4.7982109
H	6.5001079	1.1951928	5.6123719
H	5.3303396	0.7817685	4.3376735
C	2.1462174	1.3133596	7.4737836
H	2.3119633	1.8698673	6.5486944
C	-3.7009339	-1.7491271	6.7611842
C	-2.8773738	-1.5824498	5.6455689
C	-1.5386518	-1.2204771	5.8116892
C	-0.9988397	-1.0176005	7.0900526
C	-1.8379850	-1.1864672	8.2027351
C	-3.1757678	-1.5496898	8.0401562
H	-4.7443917	-2.0305459	6.6356235
H	-3.2762343	-1.7329640	4.6447287
H	-0.9047332	-1.0822502	4.9381936
H	-1.4500293	-1.0197438	9.2023868
H	-3.8105807	-1.6729972	8.9150658
C	2.7433406	-1.0539475	8.3391729
P	3.6674687	-2.6296622	7.9721867
C	2.8171786	-3.9807975	8.8882172
H	3.5075270	-4.8283179	8.9621824
H	2.5599282	-3.6474261	9.8991571
H	1.9098800	-4.3192229	8.3789299
C	5.3315739	-2.6784732	8.7185001
H	5.8267406	-3.6082514	8.4172504
H	5.9067150	-1.8168671	8.3859006
H	5.2134182	-2.6790589	9.8073805
C	3.5947975	-3.1562361	6.2296230
H	4.0178953	-4.1629385	6.1385243
H	2.5448921	-3.1867683	5.9173917
H	4.1535731	-2.4443407	5.6226988
C	2.3555879	2.3589626	8.5719224
O	2.8380809	1.8404351	9.7141857
O	2.0932765	3.5426945	8.4336497
C	2.9672267	2.7643169	10.8129664
H	3.3479982	2.1770907	11.6507072
H	3.6654455	3.5685949	10.5616446
H	1.9934350	3.1989188	11.0595273

3.4.4.20. **int3** ***anti cis-trans*** **chair**
E(B3LYP-D3/6-31+G*(THF)) = -1894.499146
E(M06-2X-D3/6-311+G**) = -1894.191963
Gtot(B3LYP-D3/6-31+G*(THF)) = -1894.037189

C	-1.6665023	1.5980906	7.1806405
C	0.1019679	0.3331385	7.5847066
C	-0.6282132	0.6317840	8.8956625
O	-1.7217316	1.4149461	8.5646124
O	-0.4040825	0.3146839	10.0352759
C	-4.8961748	3.7253661	5.3910935
C	-4.8490277	3.5835982	6.7803508
C	-3.7920035	2.8984879	7.3807235
C	-2.7733080	2.3509229	6.5855536
C	-2.8238652	2.4941168	5.1884218
C	-3.8806614	3.1796671	4.5968751

H	-5.7234770	4.2571693	4.9260674
H	-5.6381137	4.0052317	7.3984669
H	-3.7583931	2.7850608	8.4600540
H	-2.0364475	2.0564036	4.5827581
H	-3.9179772	3.2860097	3.5151567
N	-0.6759230	1.0600728	6.5820396
C	0.0939129	-1.1944311	7.2338585
H	0.6141433	-1.2470169	6.2734433
C	0.8928055	-1.9951828	8.2757060
H	0.4194479	-1.8985518	9.2564388
H	0.8475834	-3.0607943	8.0142388
C	2.4914896	-0.0170821	8.4630934
H	2.7423762	-1.8920215	9.3790300
C	3.5163221	0.4904064	9.2713411
O	3.5818524	1.8847677	9.2911772
O	4.3521716	-0.1771135	9.9386148
C	4.6136298	2.4423851	10.0995716
H	4.5438631	3.5264823	9.9696825
H	4.4832402	2.1905687	11.1589835
H	5.6047518	2.0955912	9.7842390
C	1.5817374	0.8705875	7.6602129
H	1.5434995	1.8626838	8.1162976
C	2.0503408	1.0880032	6.2173605
O	2.0304854	2.3841447	5.8711700
O	2.4185651	0.2073590	5.4506367
C	2.3875932	2.6876003	4.5047122
H	2.2792227	3.7688533	4.4048683
H	3.4191240	2.3863031	4.3001079
H	1.7147124	2.1696343	3.8153079
C	-3.9486808	-2.5965596	6.5206945
C	-3.0999309	-2.2888905	5.4542925
C	-1.7981346	-1.8521898	5.7018036
C	-1.3166291	-1.7170920	7.0117590
C	-2.1776652	-2.0289750	8.0739065
C	-3.4831460	-2.4639303	7.8305838
H	-4.9654660	-2.9346912	6.3322084
H	-3.4530979	-2.3840797	4.4294444
H	-1.1475101	-1.5962279	4.8682043
H	-1.8385592	-1.9261155	9.1012435
H	-4.1373258	-2.6973884	8.6679986
C	2.3435077	-1.4983291	8.4353919
P	3.5245989	-2.3949799	7.2413862
C	4.1481373	-3.8760484	8.1116931
H	4.8470769	-4.4319705	7.4777282
H	4.6573328	-3.5615711	9.0291265
H	3.3081517	-4.5275210	8.3778987
C	4.9502297	-1.3352867	6.8682120
H	5.7137025	-1.9119900	6.3357384
H	4.6087220	-0.5019176	6.2512467
H	5.3429371	-0.9533973	7.8148679
C	2.8144546	-2.9867602	5.6637175
H	3.5783772	-3.5461108	5.1114789
H	1.9628027	-3.6468666	5.8576409
H	2.4933874	-2.1265841	5.0738947

3.4.4.21. **int3** ***anti cis-trans*** **boat**

E(B3LYP-D3/6-31+G*(THF)) = -1894.482403

E(M06-2X-D3/6-311+G**) = -1894.175138

Gtot(B3LYP-D3/6-31+G*(THF)) = -1894.022736

C	-0.7158154	1.9588663	6.7300106
C	0.8315405	0.4101067	7.0731792
C	0.3428011	0.9735829	8.4147899
O	-0.6262379	1.9075830	8.1274173
O	0.6523765	0.7254603	9.5550104
C	-3.7030686	4.4928362	5.0622746
C	-3.5023805	4.4832276	6.4450450
C	-2.5216236	3.6649384	7.0067024
C	-1.7349316	2.8510234	6.1774821
C	-1.9399884	2.8611970	4.7872821
C	-2.9194735	3.6800641	4.2345319
H	-4.4702763	5.1304139	4.6284066
H	-4.1117963	5.1130293	7.0886465
H	-2.3681833	3.6551087	8.0816270
H	-1.3322401	2.2173426	4.1594710
H	-3.0773102	3.6833232	3.1585211
N	0.0779824	1.1916893	6.0936958
C	0.4680282	-1.1058491	6.9018912
H	0.9396478	-1.3568416	5.9483530
C	1.1200429	-1.9836986	7.9926106
H	0.4835880	-2.0292764	8.8818561
H	1.1761167	-2.9999502	7.5837368
C	3.1759408	-0.6630796	7.2534310
H	2.3617235	-0.7471941	9.2211228
C	4.3300090	-1.0842553	6.6267343
O	4.8875437	-0.1213970	5.7678545
O	4.9479322	-2.1831005	6.7771900
C	5.5874845	-0.6446200	4.6419087
H	6.0176927	0.2146542	4.1200696
H	6.3834282	-1.3303464	4.9493326
H	4.9040743	-1.1743829	3.9639929
C	2.4101589	0.5962878	6.8804853
H	2.6883043	1.4712660	7.4874639
C	2.6171022	1.0681904	5.4344323
O	2.3357253	0.1092901	4.5321436
O	2.9194343	2.2068320	5.1309077
C	2.3527401	0.5321951	3.1576531
H	2.1188615	-0.3578565	2.5704650
H	1.6000750	1.3091247	2.9919764
H	3.3384807	0.9203555	2.8857498
C	-3.7981232	-1.7274889	6.4259191
C	-2.9330578	-1.8462067	5.3355249
C	-1.5614336	-1.6526476	5.5070684
C	-1.0267171	-1.3451861	6.7653991
C	-1.9054446	-1.2321237	7.8533807
C	-3.2793196	-1.4179353	7.6852295
H	-4.8682121	-1.8739962	6.2952403
H	-3.3265714	-2.0851326	4.3495189
H	-0.8936181	-1.7313053	4.6517435
H	-1.5248495	-0.9913953	8.8435301
H	-3.9447085	-1.3200598	8.5404269
C	2.5293699	-1.4356877	8.3767065
P	3.5104715	-2.7370051	9.2255636
C	2.5826785	-3.0917034	10.7648691
H	3.1257408	-3.8401384	11.3522851
H	2.4741865	-2.1794449	11.3608131
H	1.5892147	-3.4853370	10.5272839
C	5.1358916	-2.1243124	9.7618123
H	5.5750482	-2.8359711	10.4696217
H	5.7678570	-2.0157577	8.8786861

H	5.0034073	-1.1561264	10.2569690
C	3.6237216	-4.3439868	8.3759919
H	4.2556459	-5.0116595	8.9727015
H	2.6246679	-4.7842490	8.2942827
H	4.0602886	-4.1848373	7.3903744

3.4.4.22. **int3** ***anti cis-trans*** $\mathbf{TS}_{\mathbf{boat\text{-}chair}}$
E(B3LYP-D3/6-31+G*(THF)) = -1894.474483
E(M06-2X-D3/6-311+G**) = -1894.168995
Gtot(B3LYP-D3/6-31+G*(THF)) = -1894.012656

C	-1.1515000	2.1759988	7.1837572
C	0.5842696	0.8169653	7.3842339
C	0.0169318	1.1511247	8.7687585
O	-1.0750387	1.9683119	8.5684591
O	0.3788403	0.8358656	9.8749998
C	-4.3980777	4.5116214	5.7264259
C	-4.2042199	4.3885086	7.1047927
C	-3.1429542	3.6295321	7.5997680
C	-2.2679745	2.9921749	6.7076643
C	-2.4657322	3.1156956	5.3218706
C	-3.5268040	3.8729449	4.8358789
H	-5.2276163	5.1026260	5.3444185
H	-4.8810015	4.8841199	7.7965526
H	-2.9926325	3.5326195	8.6708406
H	-1.7860411	2.6074683	4.6447875
H	-3.6789343	3.9644580	3.7629081
N	-0.2532430	1.6064720	6.4824540
C	0.4437393	-0.6989066	7.0281405
H	0.9868740	-0.8007002	6.0838977
C	1.1454780	-1.5393185	8.0995206
H	0.6453696	-1.4411777	9.0631869
H	1.0075602	-2.5822028	7.7987060
C	3.0736610	0.0705965	7.4891976
H	2.8027321	-1.0072119	9.3576318
C	4.3561024	0.1500871	6.9878509
O	4.6958630	1.4032252	6.4614512
O	5.2376142	-0.7645220	6.9606253
C	5.5927412	1.3696801	5.3514790
H	5.7750803	2.4113881	5.0746252
H	6.5374030	0.8823997	5.6128892
H	5.1434017	0.8397715	4.5011134
C	2.1096804	1.2350879	7.3061464
H	2.2525644	2.0311466	8.0522343
C	2.2687995	1.9759456	5.9655293
O	2.2417052	1.1385744	4.9123755
O	2.3147255	3.1873030	5.8600461
C	2.1895350	1.7692778	3.6213012
H	2.1688790	0.9552767	2.8942133
H	1.2867571	2.3819746	3.5356648
H	3.0689760	2.3999064	3.4616080
C	-3.6903325	-1.7982616	6.3114625
C	-2.7978185	-1.6446184	5.2478893
C	-1.4675833	-1.3019265	5.4952611
C	-1.0015531	-1.1118978	6.8033901
C	-1.9064364	-1.2736220	7.8634874
C	-3.2399253	-1.6106181	7.6197910
H	-4.7289847	-2.0606400	6.1220599
H	-3.1385454	-1.7865510	4.2242797
H	-0.7812751	-1.1663442	4.6618529
H	-1.5801750	-1.1326918	8.8913444
H	-3.9274000	-1.7253250	8.4551067
C	2.6594381	-1.1635111	8.2699380
P	3.7177798	-2.6860126	8.0642984
C	2.7898376	-4.0318953	8.9195533
H	3.4795725	-4.8626732	9.1050186
H	2.4070616	-3.6720707	9.8806553
H	1.9556073	-4.4049223	8.3195161
C	5.2565336	-2.6713518	9.0471917
H	5.7836419	-3.6179171	8.8810802
H	5.8785781	-1.8290835	8.7510014
H	4.9791586	-2.6026687	10.1054147
C	3.8869030	-3.3026582	6.3568282
H	4.3005977	-4.3174441	6.3766109
H	2.8894431	-3.3406376	5.9043501
H	4.5298855	-2.6260075	5.7955097

3.4.4.23. **int3** ***anti trans-cis*** **chair**
E(B3LYP-D3/6-31+G*(THF)) = -1894.499533
E(M06-2X-D3/6-311+G**) = -1894.194215
Gtot(B3LYP-D3/6-31+G*(THF)) = -1894.039844

C	-1.5646251	1.2073545	8.7018093
C	0.0982276	0.2613244	7.6011421
C	-0.8578528	0.9408089	6.6152626
O	-1.8709100	1.4979599	7.3671546
O	-0.8425012	1.0112606	5.4121188
C	-4.3796421	2.4433229	11.6482609
C	-4.6541883	2.6674859	10.2966438
C	-3.7362827	2.2787607	9.3203735
C	-2.5356481	1.6590803	9.6996018
C	-2.2616106	1.4343665	11.0596323
C	-3.1809767	1.8272016	12.0276117
H	-5.0979957	2.7474839	12.4064750
H	-5.5854732	3.1447261	10.0009831
H	-3.9507840	2.4490238	8.2697532
H	-1.3286038	0.9537683	11.3389011
H	-2.9653120	1.6534991	13.0792507
N	-0.4847688	0.5573834	8.9051759
C	0.0936776	-1.2842867	7.3248407
H	0.5355931	-1.3938947	6.3294170
C	0.9859887	-1.9901345	8.3601028
H	0.5724288	-1.8176056	9.3577718
H	0.9439151	-3.0735994	8.1866187
C	2.5488853	0.0181176	8.3117760
H	2.8998600	-1.7886598	9.3252028
C	3.6064233	0.5946605	9.0245085
O	3.6448980	1.9884117	8.9540323
O	4.4926005	-0.0154341	9.6833728
C	4.7048718	2.6118357	9.6723701
H	4.6011421	3.6853481	9.4889607
H	4.6387136	2.4153341	10.7492697
H	5.6861196	2.2705826	9.3218020
C	1.5668556	0.8327723	7.5131998
H	1.5304006	1.8486263	7.9117115
C	1.9552555	0.9642036	6.0369832
O	1.8577786	2.2339137	5.6116516
O	2.3209332	0.0491764	5.3113545
C	2.0831087	2.4565442	4.2019960
H	1.9599226	3.5300497	4.0500387

H	3.0907915	2.1415948	3.9176293
H	1.3449366	1.8993416	3.6186040
C	-3.9357982	-2.8821225	7.1132986
C	-3.1960693	-2.6602282	5.9494974
C	-1.8977435	-2.1525662	6.0321129
C	-1.3137491	-1.8589366	7.2734120
C	-2.0690081	-2.0846110	8.4350987
C	-3.3673459	-2.5912667	8.3557713
H	-4.9477739	-3.2771214	7.0531640
H	-3.6294282	-2.8804797	4.9760958
H	-1.3324240	-1.9701532	5.1204499
H	-1.6489010	-1.8520542	9.4083465
H	-3.9368683	-2.7563766	9.2679308
C	2.4321856	-1.4628513	8.3871287
P	3.5521574	-2.4129258	7.1782936
C	4.2512052	-3.8326046	8.0930566
H	4.9146430	-4.4193820	7.4488633
H	4.8153988	-3.4573850	8.9536882
H	3.4400324	-4.4753329	8.4531766
C	4.9385199	-1.3614102	6.6601000
H	5.6785160	-1.9611205	6.1195170
H	4.5523459	-0.5679486	6.0174593
H	5.3794252	-0.9217046	7.5594185
C	2.7511496	-3.1066953	5.6886614
H	3.4894139	-3.6784457	5.1150307
H	1.9332805	-3.7744881	5.9786839
H	2.3673415	-2.2888876	5.0765061

3.4.4.24. **int3** ***anti trans-cis*** **boat**
E(B3LYP-D3/6-31+G*(THF)) = -1894.483451
E(M06-2X-D3/6-311+G**) = -1894.175698
Gtot(B3LYP-D3/6-31+G*(THF)) = -1894.020514

C	-0.8093385	1.4445900	8.2704842
C	0.5797087	0.2469356	7.0277602
C	-0.3682904	1.0587062	6.1283646
O	-1.1962713	1.7731115	6.9736652
O	-0.4901222	1.1169950	4.9336625
C	-3.0753590	3.1582469	11.4425664
C	-3.4389600	3.4144177	10.1179531
C	-2.6996648	2.8668606	9.0691191
C	-1.5889932	2.0554528	9.3484196
C	-1.2251523	1.7998248	10.6812089
C	-1.9661946	2.3507888	11.7221498
H	-3.6539175	3.5870295	12.2578464
H	-4.3003156	4.0410990	9.8996273
H	-2.9829843	3.0637362	8.0397456
H	-0.3617184	1.1728343	10.8825332
H	-1.6812998	2.1533296	12.7528961
N	0.1661040	0.6265453	8.3792847
C	0.3482176	-1.2802418	6.7737412
H	0.8108523	-1.4558869	5.7992433
C	1.1010273	-2.1342344	7.8271962
H	0.4434059	-2.3464331	8.6758646
H	1.3365684	-3.0933090	7.3500010
C	3.0067421	-0.5508067	7.2490072
H	2.0631335	-0.7615126	9.1552612
C	4.2487958	-0.8045960	6.7068167
O	4.7088577	0.2216559	5.8613304
O	5.0125184	-1.7966333	6.9127753
C	5.5418049	-0.2088161	4.7870323
H	5.8652939	0.6960423	4.2652904
H	6.4142221	-0.7589313	5.1537001
H	4.9849283	-0.8496279	4.0896338
C	2.1189268	0.5992426	6.8011508
H	2.2771103	1.5254651	7.3726995
C	2.3179841	1.0310387	5.3425407
O	2.2107803	0.0114347	4.4720974
O	2.4485858	2.1929407	5.0023132
C	2.1872873	0.3838069	3.0803860
H	2.1029141	-0.5530650	2.5260268
H	1.3242508	1.0249944	2.8819715
H	3.1064695	0.9093778	2.8073583
C	-3.8528356	-2.3160089	6.4325546
C	-3.0234299	-2.3259592	5.3087506
C	-1.6720961	-1.9949529	5.4322541
C	-1.1225231	-1.6550806	6.6764240
C	-1.9680291	-1.6481287	7.7979614
C	-3.3200174	-1.9730954	7.6776981
H	-4.9061471	-2.5718858	6.3391142
H	-3.4283210	-2.5889621	4.3337094
H	-1.0342453	-1.9938166	4.5509449
H	-1.5718925	-1.3760430	8.7715025
H	-3.9582895	-1.9574722	8.5587045
C	2.3869452	-1.4110498	8.3234504
P	3.4725852	-2.5880562	9.2305955
C	2.5043913	-3.1071750	10.6980072
H	3.1046953	-3.7972811	11.3014396
H	2.2431941	-2.2374112	11.3096889
H	1.5872996	-3.6191940	10.3899194
C	4.9604494	-1.7803099	9.8955264
H	5.4447856	-2.4515375	10.6138169
H	5.6297785	-1.5563795	9.0631429
H	4.6648712	-0.8574396	10.4067100
C	3.8554054	-4.1431361	8.3635171
H	4.5363847	-4.7343851	8.9863639
H	2.9318701	-4.7115277	8.2126827
H	4.3244622	-3.9017723	7.4098542

3.4.4.25. **int3** ***anti trans-cis*** **TS$_{boat-chair}$**
E(B3LYP-D3/6-31+G*(THF)) = -1894.474705
E(M06-2X-D3/6-311+G**) = -1894.169507
Gtot(B3LYP-D3/6-31+G*(THF)) = -1894.012317

C	-0.8431985	1.9717154	8.6087165
C	0.6207356	0.8855960	7.3520520
C	-0.3221838	1.7196123	6.4633527
O	-1.1937397	2.3669885	7.3189617
O	-0.4249864	1.8242073	5.2702651
C	-3.2126389	3.4949810	11.8016174
C	-3.5273254	3.8393450	10.4843778
C	-2.7568459	3.3512686	9.4286690
C	-1.6630189	2.5133891	9.6938893
C	-1.3477695	2.1682736	11.0192449
C	-2.1214427	2.6586479	12.0669149
H	-3.8153432	3.8777324	12.6222770
H	-4.3742834	4.4891019	10.2775293
H	-3.0012912	3.6166126	8.4046530
H	-0.4971757	1.5203184	11.2101016
H	-1.8750309	2.3916874	13.0919196

N	0.1443494	1.1680251	8.7056141
C	0.4460799	-0.6255526	7.0037623
H	0.9685300	-0.7616188	6.0508330
C	1.1394051	-1.4513023	8.0941686
H	0.6417764	-1.2826202	9.0499132
H	0.9732737	-2.5016410	7.8400447
C	3.0971466	0.0869827	7.4362839
H	2.8118822	-0.9284606	9.3510247
C	4.3843912	0.1371680	6.9420396
O	4.7547182	1.3868953	6.4278590
O	5.2474148	-0.7942311	6.9201059
C	5.7024214	1.3488226	5.3614330
H	5.8980922	2.3896036	5.0902911
H	6.6329513	0.8618645	5.6696413
H	5.2941525	0.8157838	4.4929746
C	2.1579535	1.2679222	7.2319938
H	2.2997932	2.0657785	7.9740877
C	2.3480103	1.9813920	5.8855666
O	2.3788326	1.1315028	4.8462033
O	2.3395778	3.1942221	5.7649576
C	2.3444200	1.7419657	3.5428836
H	2.3783084	0.9158998	2.8300996
H	1.4202531	2.3132641	3.4234878
H	3.2051398	2.4022614	3.4035593
C	-3.7053070	-1.7526700	6.4269756
C	-2.8478599	-1.6065007	5.3339379
C	-1.5131002	-1.2492715	5.5339990
C	-1.0075791	-1.0364142	6.8244970
C	-1.8804835	-1.1842765	7.9147535
C	-3.2164822	-1.5390434	7.7177733
H	-4.7463211	-2.0294131	6.2743902
H	-3.2186017	-1.7675497	4.3238736
H	-0.8547102	-1.1228531	4.6773238
H	-1.5225094	-1.0133309	8.9252723
H	-3.8771677	-1.6465663	8.5754504
C	2.6572749	-1.1010484	8.2674486
P	3.6738049	-2.6605177	8.0818531
C	2.7239801	-3.9735219	8.9669783
H	3.4037300	-4.8063310	9.1791563
H	2.3390656	-3.5847776	9.9156497
H	1.8897429	-4.3558485	8.3722835
C	5.2208012	-2.6637259	9.0523671
H	5.7242931	-3.6261879	8.9050527
H	5.8612638	-1.8440531	8.7326767
H	4.9536775	-2.5639908	10.1107923
C	3.8190288	-3.3071638	6.3832940
H	4.2071512	-4.3316893	6.4190568
H	2.8199005	-3.3274765	5.9333844
H	4.4772147	-2.6564455	5.8091271

3.4.4.26. **int3** ***anti trans-trans*** **chair**
E(B3LYP-D3/6-31+G*(THF)) = -1894.491511
E(M06-2X-D3/6-311+G**) = -1894.179970
Gtot(B3LYP-D3/6-31+G*(THF)) = -1894.031206

C	-1.5863633	1.5938189	7.2063822
C	0.1781991	0.3322699	7.6123647
C	-0.5042983	0.6928514	8.9279602
O	-1.6128276	1.4559422	8.5905168
O	-0.2673219	0.3991958	10.0708349
C	-4.8548485	3.6743637	5.4313465
C	-4.7761078	3.5686869	6.8223462
C	-3.7037679	2.9019420	7.4156880
C	-2.7011679	2.3359746	6.6127007
C	-2.7824464	2.4450174	5.2139891
C	-3.8547270	3.1123521	4.6289638
H	-5.6947556	4.1907725	4.9714745
H	-5.5529627	4.0031793	7.4471114
H	-3.6456894	2.8139069	8.4962204
H	-2.0062281	1.9961545	4.6011844
H	-3.9165026	3.1911646	3.5460184
N	-0.6139140	1.0275945	6.5988982
C	0.1032271	-1.2143895	7.3292187
H	0.5274404	-1.3152921	6.3199173
C	0.9749582	-2.0103978	8.3137211
H	0.5637179	-1.9099127	9.3220429
H	0.9208534	-3.0754205	8.0542225
C	2.5693718	-0.0234993	8.4857840
H	2.8746152	-1.9361552	9.3188195
C	3.3570346	0.4824200	9.5323403
O	3.3628200	1.8691441	9.6119233
O	4.0459147	-0.1831740	10.3474424
C	4.1667427	2.4372301	10.6403851
H	4.0658079	3.5209171	10.5329142
H	3.8256162	2.1316390	11.6363212
H	5.2200488	2.1526415	10.5335010
C	1.6954034	0.7515388	7.5262936
H	1.9365665	0.4858783	6.4869111
C	1.8855654	2.2652248	7.5149506
O	1.0171612	2.9035977	8.3157263
O	2.6892998	2.8362965	6.8011036
C	1.1171740	4.3393827	8.3501542
H	0.3497238	4.6710541	9.0510365
H	2.1105394	4.6402169	8.6946682
H	0.9355759	4.7582123	7.3555774
C	-4.0179513	-2.5511036	7.0653803
C	-3.2864847	-2.2611511	5.9108814
C	-1.9583430	-1.8448650	6.0111998
C	-1.3336982	-1.7126286	7.2593234
C	-2.0792312	-2.0048839	8.4113179
C	-3.4095698	-2.4197719	8.3152555
H	-5.0542870	-2.8739256	6.9914748
H	-3.7515894	-2.3549063	4.9317439
H	-1.3996376	-1.6033693	5.1093698
H	-1.6319678	-1.8986505	9.3952378
H	-3.9706780	-2.6376951	9.2216208
C	2.4291178	-1.5102662	8.4107023
P	3.6119291	-2.1939275	7.1243314
C	3.3295634	-3.9512805	6.6923992
H	4.1545157	-4.3257142	6.0750101
H	3.2685417	-4.5454719	7.6109479
H	2.3920623	-4.0561688	6.1360474
C	5.2644879	-2.0604548	7.8763572
H	6.0410385	-2.1527284	7.1104178
H	5.3330114	-1.0938286	8.3831389
H	5.3876831	-2.8518624	8.6231667
C	3.6742245	-1.2647109	5.5536251
H	4.4838463	-1.6778368	4.9411302
H	2.7333655	-1.3484445	5.0015035
H	3.8886094	-0.2134009	5.7656033

3.4.4.27. **int3 *anti trans-trans* boat**
E(B3LYP-D3/6-31+G*(THF)) = -1894.488382
E(M06-2X-D3/6-311+G**) = -1894.185029
Gtot(B3LYP-D3/6-31+G*(THF)) = -1894.027745

C	-0.8207904	1.8879067	6.7271424
C	0.7621044	0.4287430	7.2217097
C	0.0688063	0.8971293	8.4998501
O	-0.9307470	1.7615005	8.1192633
O	0.2572144	0.6106386	9.6593535
C	-3.7244009	4.3099767	4.7672607
C	-3.7746540	4.1611601	6.1556374
C	-2.8211881	3.3827487	6.8127086
C	-1.8102577	2.7468075	6.0757632
C	-1.7611245	2.8982994	4.6790133
C	-2.7146461	3.6781265	4.0313754
H	-4.4710633	4.9150503	4.2573734
H	-4.5594725	4.6495479	6.7285252
H	-2.8640947	3.2618392	7.8908737
H	-0.9772219	2.3969811	4.1183900
H	-2.6750595	3.7919289	2.9505161
N	0.1086541	1.2088138	6.1771777
C	0.4714477	-1.1005681	6.9761347
H	1.0090666	-1.2996957	6.0442835
C	1.0980821	-2.0099607	8.0628438
H	0.4369994	-2.1008800	8.9307645
H	1.1917683	-3.0075428	7.6179580
C	3.1053086	-0.6291589	7.3882583
H	2.2918573	-0.7544805	9.3300699
C	3.9283467	-1.1932621	6.4246539
O	4.2539342	-0.3262362	5.3729051
O	4.4385652	-2.3532257	6.4333370
C	5.0300108	-0.8999662	4.3255594
H	5.1925029	-0.0993945	3.5977158
H	5.9962387	-1.2680203	4.6898706
H	4.5074104	-1.7340821	3.8406726
C	2.3562991	0.6507813	7.2053408
H	2.5588668	1.0698803	6.2151778
C	2.7393721	1.7409738	8.2155576
O	1.9985236	2.8557866	8.0184360
O	3.5863641	1.6567887	9.0839309
C	2.2164202	3.9475233	8.9374908
H	1.5633150	4.7530452	8.5976645
H	1.9528853	3.6440222	9.9551154
H	3.2622524	4.2666214	8.9115861
C	-3.7339510	-1.8323362	6.1934196
C	-2.8084297	-1.7895525	5.1478408
C	-1.4580241	-1.5646927	5.4182237
C	-1.0028838	-1.3840754	6.7321879
C	-1.9427135	-1.4289878	7.7730626
C	-3.2961350	-1.6494216	7.5066970
H	-4.7879655	-2.0046428	5.9860639
H	-3.1382100	-1.9270581	4.1200732
H	-0.7447129	-1.5155324	4.5981417
H	-1.6258781	-1.2818777	8.8026185
H	-4.0089498	-1.6764629	8.3282307
C	2.4847814	-1.4424773	8.4926824
P	3.5690566	-2.6716619	9.3106508
C	2.9506042	-2.8610648	11.0210814
H	3.5295028	-3.6277980	11.5476048
H	3.0403740	-1.9108213	11.5580619
H	1.8973522	-3.1619191	11.0012941
C	5.2557591	-2.0099421	9.4100884
H	5.8328285	-2.5545349	10.1641509
H	5.7090465	-2.1061150	8.4211720
H	5.1872999	-0.9498845	9.6750192
C	3.5297534	-4.3386681	8.5829806
H	4.2942245	-4.9597201	9.0631499
H	2.5456636	-4.7877745	8.7531601
H	3.7317114	-4.2441888	7.5151031

3.4.4.28. **int3 *anti trans-trans* TS$_{boat-chair}$**
E(B3LYP-D3/6-31+G*(THF)) = -1894.480082
E(M06-2X-D3/6-311+G**) = -1894.175452
Gtot(B3LYP-D3/6-31+G*(THF)) = -1894.017580

C	-1.1936904	2.1885527	7.0482997
C	0.5142529	0.8604127	7.5120428
C	-0.2778409	1.1630357	8.7882637
O	-1.3449093	1.9473767	8.4181906
O	-0.1119200	0.8091956	9.9303044
C	-4.2077399	4.5452676	5.1766832
C	-4.2939313	4.2727541	6.5442520
C	-3.3084707	3.5096072	7.1709097
C	-2.2287326	3.0134666	6.4240382
C	-2.1444030	3.2883968	5.0484030
C	-3.1304021	4.0520390	4.4308470
H	-4.9776624	5.1409740	4.6911075
H	-5.1300603	4.6550062	7.1250262
H	-3.3762505	3.2966291	8.2333340
H	-1.3044688	2.9003033	4.4794548
H	-3.0614653	4.2640259	3.3664309
N	-0.1841514	1.6406083	6.4906285
C	0.4072894	-0.6691508	7.1819298
H	0.9270979	-0.7694802	6.2225682
C	1.1772095	-1.4838595	8.2322324
H	0.7430561	-1.3405870	9.2223362
H	1.0202095	-2.5378608	7.9818670
C	2.9991790	0.1081682	7.4251989
H	2.9257722	-0.8785970	9.3569388
C	4.1797367	0.2488209	6.7101665
O	4.3064807	1.4794602	6.0533885
O	5.1141299	-0.5971923	6.5734751
C	5.4332755	1.6005955	5.1908822
H	5.3875768	2.6128802	4.7786290
H	6.3754057	1.4645873	5.7339455
H	5.4013424	0.8704554	4.3725560
C	2.0500363	1.2638061	7.5692972
H	2.1810579	1.9208664	6.7057703
C	-3.7323542	-1.8277017	6.5778640
C	-2.9444823	-1.3964569	5.5070623
C	-1.6108233	-1.0456894	5.7178238
C	-1.0359167	-1.1155923	6.9960176
C	-1.8384494	-1.5468302	8.0630372
C	-3.1743779	-1.9011437	7.8555134
H	-4.7729671	-2.1019173	6.4174348
H	-3.3692270	-1.3308979	4.5074415
H	-1.0051531	-0.6979624	4.8840608
H	-1.4320476	-1.5964173	9.0693812

H	-3.7803106	-2.2308970	8.6970239
C	2.7027668	-1.0923793	8.2934185
P	3.7518316	-2.5964985	8.0498606
C	2.9820225	-3.9370557	9.0460480
H	3.7174497	-4.7402439	9.1674919
H	2.7050982	-3.5605537	10.0366943
H	2.0956946	-4.3501889	8.5567615
C	5.4022011	-2.4358115	8.8037885
H	5.9841283	-3.3356468	8.5746248
H	5.8952021	-1.5532553	8.3981119
H	5.2767195	-2.3573044	9.8896771
C	3.7600308	-3.2403407	6.3461946
H	4.2111903	-4.2390114	6.3373105
H	2.7237955	-3.3185820	5.9987248
H	4.3202471	-2.5518651	5.7133739
C	2.2543467	2.2192780	8.7558891
O	3.1800258	1.8161690	9.6353550
O	1.6281605	3.2619680	8.8764518
C	3.3973838	2.6975855	10.7595651
H	4.1936834	2.2364255	11.3458963
H	3.7011225	3.6902337	10.4148990
H	2.4819440	2.7865459	11.3524340

3.4.4.29. **int3** ***syn cis-cis*** **chair**
E(B3LYP-D3/6-31+G*(THF)) = -1894.491907
E(M06-2X-D3/6-311+G**) = -1894.187196
Gtot(B3LYP-D3/6-31+G*(THF)) = -1894.030023

C	0.7561575	-2.7203989	3.8755656
C	1.1032084	-1.3326024	5.5435390
C	0.1449798	-0.7002479	4.5388822
O	-0.0629547	-1.6353465	3.5475494
O	-0.4030291	0.3759801	4.5420456
C	0.6706873	-6.0830549	1.2613707
C	-0.1310833	-4.9734162	0.9822077
C	-0.1103097	-3.8620070	1.8260575
C	0.7180479	-3.8601005	2.9580179
C	1.5226048	-4.9776498	3.2371437
C	1.4979449	-6.0820666	2.3909961
H	0.6532707	-6.9473661	0.6009185
H	-0.7741654	-4.9717893	0.1051607
H	-0.7329558	-2.9991999	1.6102433
H	2.1589169	-4.9595944	4.1169815
H	2.1238611	-6.9443530	2.6090875
N	1.4376322	-2.6165945	4.9490398
C	0.3334862	-1.5143874	6.9080147
C	1.2561036	-2.0393322	8.0292736
H	0.6553002	-2.1028258	8.9480171
H	1.6066498	-3.0456267	7.7826833
C	3.2489615	-1.0377519	6.8735797
H	2.1224756	-0.1100650	8.4470846
C	4.2454089	-1.9697769	6.6003327
O	4.6626837	-1.9857245	5.2741359
O	4.8231717	-2.7331501	7.4289064
C	5.4925809	-3.0771982	4.8976936
H	5.6952764	-2.9456777	3.8313046
H	6.4356277	-3.0829654	5.4566687
H	4.9883751	-4.0381591	5.0590686
C	2.3707251	-0.4335487	5.8085216
H	1.9577666	0.5212102	6.1791451
C	3.0445071	-0.0463909	4.4975007
O	4.0734957	0.7925764	4.7006560
O	2.6504679	-0.3595108	3.3870772
C	4.7846560	1.1969118	3.5148388
H	5.5626278	1.8824341	3.8558232
H	5.2303147	0.3240085	3.0285406
H	4.1143022	1.6987456	2.8100462
H	0.0145536	-0.4999984	7.1859182
C	2.4945501	-1.1161400	8.1795588
P	3.4572157	-1.5539891	9.6899419
C	2.5301545	-0.8211863	11.0895321
H	3.0248453	-1.0689221	12.0353723
H	2.4883307	0.2675341	10.9801600
H	1.5085840	-1.2154788	11.1110583
C	5.0993973	-0.7767429	9.7150240
H	5.5333608	-0.8682708	10.7167864
H	5.7220147	-1.2774688	8.9723163
H	4.9892084	0.2819666	9.4586381
C	3.5283183	-3.3355346	10.0605546
H	4.1236929	-3.4900269	10.9674706
H	2.5126723	-3.7050889	10.2359597
H	3.9809439	-3.8478001	9.2113733
C	-3.2612594	-3.8936873	6.4032643
C	-3.3426155	-2.5070552	6.5462518
C	-2.1798496	-1.7510595	6.7161566
C	-0.9189511	-2.3632754	6.7475199
C	-0.8512425	-3.7582068	6.6019124
C	-2.0104438	-4.5159838	6.4312720
H	-4.1643184	-4.4855157	6.2685850
H	-4.3110108	-2.0115189	6.5236598
H	-2.2509797	-0.6697516	6.8170937
H	0.1146193	-4.2531714	6.6003356
H	-1.9355115	-5.5951652	6.3145171

3.4.4.30. **int3** ***syn cis-cis*** **boat**
E(B3LYP-D3/6-31+G*(THF)) = -1894.497989
E(M06-2X-D3/6-311+G**) = -1894.191061
Gtot(B3LYP-D3/6-31+G*(THF)) = -1894.036148

C	0.8296808	-2.7166003	3.9194766
C	1.0918105	-1.2606770	5.5619382
C	0.1254339	-0.7013694	4.5202066
O	-0.0193876	-1.6759405	3.5501122
O	-0.4601811	0.3478007	4.4675698
C	0.7982391	-6.1882614	1.4526949
C	-0.0252504	-5.1062474	1.1309997
C	-0.0154199	-3.9571150	1.9222920
C	0.8238772	-3.8909642	3.0454341
C	1.6512706	-4.9803956	3.3659278
C	1.6379542	-6.1223427	2.5711271
H	0.7857272	-7.0836027	0.8349022
H	-0.6794559	-5.1578713	0.2639064
H	-0.6594682	-3.1184782	1.6760006
H	2.2919505	-4.9163330	4.2400640
H	2.2784272	-6.9645796	2.8222328
N	1.4997138	-2.5484893	4.9957873
C	0.3317327	-1.4911178	6.9133872
C	1.2943989	-2.1622403	7.9111177
H	0.7557163	-2.3654702	8.8424696
H	1.5862643	-3.1316189	7.4980535

C	2.7038989	-0.1286359	7.2342047
H	2.4310284	-0.8132906	9.1969453
C	3.1224101	1.0920566	7.7752939
O	3.2068316	2.1166208	6.8258381
O	3.4192459	1.3373646	8.9754915
C	3.5122032	3.4093366	7.3396880
H	3.5557254	4.0749696	6.4724623
H	2.7406716	3.7644893	8.0342353
H	4.4752686	3.4181931	7.8627319
C	2.2955895	-0.2576114	5.7987872
H	1.9302491	0.7188922	5.4624886
C	3.4003178	-0.6258941	4.7984450
O	2.9694396	-0.4177996	3.5348137
O	4.5118710	-1.0535952	5.0519432
C	3.8520391	-0.8301721	2.4692912
H	3.3241210	-0.5988407	1.5426598
H	4.7981112	-0.2831717	2.5174114
H	4.0488272	-1.9043042	2.5389245
H	0.0850987	-0.4889883	7.2816431
C	2.5262011	-1.2562653	8.1969167
P	4.0915851	-2.2678211	8.4094221
C	4.1255437	-3.0602366	10.0572764
H	5.0546861	-3.6246003	10.1945106
H	4.0541897	-2.2873933	10.8308350
H	3.2734760	-3.7417168	10.1560580
C	5.5160926	-1.1514571	8.2802466
H	6.4289078	-1.6641183	8.6006231
H	5.5937311	-0.8379760	7.2365924
H	5.3206706	-0.2715114	8.8999233
C	4.2668814	-3.5913068	7.1644185
H	5.3090466	-3.9271928	7.1331371
H	3.6252977	-4.4394424	7.4231882
H	3.9809673	-3.1954576	6.1870104
C	-3.4011176	-3.6217504	6.3133813
C	-3.3922443	-2.2393312	6.5128693
C	-2.1841902	-1.5692416	6.7158229
C	-0.9647528	-2.2629696	6.7227654
C	-0.9881656	-3.6522359	6.5207015
C	-2.1943691	-4.3253345	6.3184533
H	-4.3405296	-4.1471254	6.1547385
H	-4.3258460	-1.6807667	6.5101219
H	-2.1835587	-0.4912790	6.8633689
H	-0.0593171	-4.2139361	6.5063620
H	-2.1902622	-5.4019088	6.1615161

3.4.4.31. **int3 *syn cis-trans* chair**
E(B3LYP-D3/6-31+G*(THF)) = -1894.480207
E(M06-2X-D3/6-311+G**) = -1894.171369
Gtot(B3LYP-D3/6-31+G*(THF)) = -1894.016690

C	1.3207936	-1.9271465	3.5416418
C	2.4838897	-2.4618869	5.3424135
C	3.0009602	-3.2454003	4.1304797
O	2.1850113	-2.9148057	3.0672259
O	3.9226376	-4.0118740	3.9969266
C	-1.5038521	-0.3251181	0.7876687
C	-0.6075599	-1.3083019	0.3606009
C	0.3284465	-1.8382397	1.2495673
C	0.3663751	-1.3816695	2.5751409
C	-0.5350908	-0.3922742	3.0027772
C	-1.4648512	0.1324826	2.1104134
H	-2.2324574	0.0857802	0.0923762
H	-0.6362414	-1.6640074	-0.6665114
H	1.0265211	-2.6016954	0.9197111
H	-0.4915927	-0.0484057	4.0320741
H	-2.1606277	0.8987601	2.4440560
N	1.4402856	-1.6104905	4.7697424
C	1.7737227	-3.3804333	6.4062751
C	1.2416226	-2.4776411	7.5555342
H	0.4380324	-1.8658762	7.1350841
H	0.7910501	-3.1085332	8.3299354
C	3.0647369	-0.7510666	7.1425622
H	1.6339695	-0.7295081	8.7063303
C	3.1123101	0.6462298	7.0494925
O	2.5538544	1.2882942	8.1756989
O	3.5930887	1.3492377	6.1367627
C	2.5595753	2.7121785	8.1210213
H	2.1007566	3.0511551	9.0546759
H	3.5786126	3.1089458	8.0448462
H	1.9830468	3.0876201	7.2675567
C	3.6116308	-1.6250532	6.0426113
H	4.2867692	-2.3750203	6.4729304
C	4.4817117	-0.8837878	5.0380358
O	5.6536882	-0.5403241	5.6050926
O	4.2277703	-0.7227653	3.8581107
C	6.4960346	0.3189458	4.8151420
H	7.3864272	0.5004840	5.4202752
H	6.7677726	-0.1627032	3.8710825
H	5.9704288	1.2569314	4.6168337
H	0.8776384	-3.7547241	5.8939528
C	2.2380215	-1.4602666	8.1588822
P	3.3151684	-2.0897982	9.5909948
C	2.6548327	-3.5031755	10.5485843
H	3.2948645	-3.7017810	11.4161677
H	1.6428608	-3.2695940	10.8969398
H	2.6182963	-4.3918678	9.9118383
C	3.4372501	-0.6724062	10.7298184
H	4.1933413	-0.8609619	11.4986802
H	3.6979234	0.2127763	10.1421775
H	2.4649899	-0.5037029	11.2056939
C	5.0232706	-2.4963682	9.1074936
H	5.6541612	-2.5090679	10.0028149
H	5.0594058	-3.4755450	8.6255820
H	5.3780188	-1.7232289	8.4191998
C	3.5330609	-7.0010610	8.0904567
C	2.1642848	-6.7258299	8.1708281
C	1.6521958	-5.5621643	7.5995003
C	2.4809725	-4.6318814	6.9410610
C	3.8456566	-4.9377761	6.8482474
C	4.3649319	-6.1062283	7.4191093
H	3.9391225	-7.9080586	8.5329475
H	1.4930554	-7.4205770	8.6715385
H	0.5815772	-5.3719517	7.6615905
H	4.5191228	-4.2958150	6.2973916
H	5.4285142	-6.3145217	7.3239835

3.4.4.32. **int3 *syn cis-trans* boat**
E(B3LYP-D3/6-31+G*(THF)) = -1894.486885
E(M06-2X-D3/6-311+G**) = -1894.179241
Gtot(B3LYP-D3/6-31+G*(THF)) = -1894.027332

C	0.2352893	-1.6678520	3.6508862
C	1.2694903	-1.2270577	5.5592296
C	1.1322218	-2.7398179	5.3728671
O	0.4621325	-2.9447117	4.1942894
O	1.4877922	-3.6651136	6.0657575
C	-2.0350748	-1.4648768	0.0562146
C	-1.6970759	-2.7126993	0.5866246
C	-0.9445007	-2.7968641	1.7585432
C	-0.5267883	-1.6227544	2.4036826
C	-0.8685111	-0.3690221	1.8686118
C	-1.6184356	-0.2933490	0.6993909
H	-2.6248892	-1.4031383	-0.8557237
H	-2.0219003	-3.6229150	0.0883450
H	-0.6854858	-3.7666527	2.1727184
H	-0.5485041	0.5307008	2.3844493
H	-1.8838478	0.6784472	0.2898640
N	0.6881028	-0.6895621	4.3289172
C	0.4304277	-0.7283152	6.7855333
C	0.8982252	-1.4249854	8.0734019
H	0.3724648	-0.9651546	8.9171902
H	0.5841532	-2.4702271	8.0557879
C	3.1720574	-0.5865075	7.1861913
H	2.6134999	-0.7088812	9.1982264
C	4.2681240	0.1935569	7.5714587
O	5.0194805	0.6482021	6.4823324
O	4.6321704	0.5001497	8.7371863
C	5.9227913	1.7167122	6.7524868
H	6.4390263	1.9201887	5.8102519
H	5.3907156	2.6187823	7.0808120
H	6.6494734	1.4415184	7.5242383
C	2.7901988	-0.8019375	5.7335697
H	3.3871671	-1.5751030	5.2255241
C	2.9976679	0.4371830	4.8466292
O	2.4371462	1.5420545	5.3693250
O	3.5457062	0.4087511	3.7607633
C	2.5028260	2.7173085	4.5423059
H	2.0214158	3.5118787	5.1153742
H	3.5435639	2.9777062	4.3284851
H	1.9729818	2.5492510	3.5997055
H	0.6827315	0.3341881	6.8536161
C	2.4303092	-1.2765328	8.2795732
P	3.3074674	-2.9119222	8.7444844
C	2.2469196	-4.3064875	9.2786857
H	2.8733491	-5.1270085	9.6474164
H	1.5783109	-3.9776102	10.0815138
H	1.6544152	-4.6590234	8.4302310
C	4.4135506	-2.4941910	10.1331545
H	5.0846514	-3.3288406	10.3616757
H	4.9814401	-1.6033581	9.8453273
H	3.8135495	-2.2554573	11.0182816
C	4.3841956	-3.5109697	7.4033144
H	5.0232656	-4.3140673	7.7851730
H	3.7759912	-3.8829863	6.5774944
H	5.0027582	-2.6772916	7.0591458
C	-3.8398369	-0.9921594	6.0686009
C	-3.1833991	0.2406007	6.0343093
C	-1.8105532	0.3082446	6.2753647
C	-1.0682325	-0.8461673	6.5601570
C	-1.7397042	-2.0774758	6.5950713
C	-3.1126375	-2.1507930	6.3486703
H	-4.9093877	-1.0492147	5.8779958
H	-3.7395780	1.1500566	5.8162828
H	-1.3015000	1.2688932	6.2349959
H	-1.1962332	-2.9945608	6.8101444
H	-3.6138575	-3.1159725	6.3762355

3.4.4.33. **int3** ***syn trans-cis*** **chair**

E(B3LYP-D3/6-31+G*(THF)) = -1894.485705

E(M06-2X-D3/6-311+G**) = -1894.174157

Gtot(B3LYP-D3/6-31+G*(THF)) = -1894.022524

C	2.2535790	-3.0114282	3.2992456
C	2.3795278	-2.4055071	5.4234941
C	1.2996655	-1.5799178	4.7043872
O	1.2519164	-2.0544513	3.3997676
O	0.5281999	-0.7426134	5.0916021
C	2.7337463	-4.9735066	-0.4519614
C	1.7809722	-3.9596528	-0.3214878
C	1.6180850	-3.3014630	0.8984066
C	2.4147048	-3.6604142	1.9957894
C	3.3740845	-4.6788508	1.8615105
C	3.5310270	-5.3307624	0.6422166
H	2.8553615	-5.4870159	-1.4032058
H	1.1615162	-3.6812180	-1.1706027
H	0.8770197	-2.5146798	1.0020350
H	3.9805073	-4.9496406	2.7207095
H	4.2719367	-6.1207988	0.5431681
N	2.9217384	-3.2495582	4.3604108
C	1.6759995	-3.2950591	6.5091611
C	1.1971114	-2.4141893	7.6911468
H	0.3790167	-1.7879024	7.3238418
H	0.7739480	-3.0540029	8.4733670
C	3.0128658	-0.6858559	7.2343384
H	1.6642079	-0.7039956	8.8706304
C	3.2060298	0.7007672	7.2857085
O	2.6847676	1.2843836	8.4595657
O	3.7759505	1.4434112	6.4563612
C	2.8253480	2.7005132	8.5417980
H	2.3779959	2.9911853	9.4968129
H	3.8783566	3.0044306	8.5192270
H	2.3060607	3.2074342	7.7202929
C	3.4998167	-1.5098430	6.0623262
H	4.2652229	-2.2267894	6.3841378
C	4.1616792	-0.6713256	4.9756704
O	5.4761555	-0.5264252	5.2183315
O	3.6053515	-0.2603061	3.9732605
C	6.1670290	0.3907334	4.3487099
H	7.2055232	0.3942166	4.6856551
H	6.1036391	0.0602257	3.3077176
H	5.7256116	1.3858985	4.4491041
H	0.7618622	-3.6734519	6.0299863
C	2.2299166	-1.4219320	8.2681373
P	3.3694535	-2.1179895	9.6250025
C	2.7187742	-3.5340774	10.5850220
H	3.3862137	-3.7518411	11.4271047
H	1.7242683	-3.2862930	10.9719007
H	2.6440707	-4.4148649	9.9416436
C	3.6032317	-0.7509327	10.8067827
H	4.3647800	-1.0169327	11.5472448

H	3.9025532	0.1404535	10.2496046
H	2.6543450	-0.5446576	11.3139185
C	5.0349044	-2.5611561	9.0369636
H	5.6972697	-2.6772580	9.9016265
H	5.0038446	-3.4961919	8.4741485
H	5.4044232	-1.7513795	8.4007749
C	3.5648826	-6.9604339	7.9560883
C	2.2187282	-6.6692654	8.1977438
C	1.6593900	-5.4888236	7.7076740
C	2.4183260	-4.5585557	6.9719714
C	3.7576243	-4.8823232	6.7110025
C	4.3241318	-6.0646196	7.2029086
H	4.0071566	-7.8792558	8.3348536
H	1.5996440	-7.3643891	8.7612914
H	0.6047007	-5.2905213	7.8936802
H	4.3652585	-4.2371122	6.0905620
H	5.3665770	-6.2841233	6.9814741

3.4.4.35. **int3 *syn trans-cis* boat**
E(B3LYP-D3/6-31+G*(THF)) = -1894.490641
E(M06-2X-D3/6-311+G**) = -1894.179280
Gtot(B3LYP-D3/6-31+G*(THF)) = -1894.029995

C	0.3530086	-2.2348692	3.7095611
C	1.2455770	-1.5570362	5.6097502
C	0.8087052	-0.3449826	4.7863768
O	0.2486029	-0.8476668	3.6216083
O	0.7989368	0.8326726	5.0353568
C	-1.3837190	-4.5253279	0.5633531
C	-1.4066726	-3.1299140	0.4933108
C	-0.8296883	-2.3661651	1.5090429
C	-0.2248565	-3.0027921	2.6036289
C	-0.2022612	-4.4064653	2.6706425
C	-0.7787962	-5.1614564	1.6539114
H	-1.8382005	-5.1177094	-0.2279630
H	-1.8780234	-2.6344660	-0.3522636
H	-0.8537037	-1.2817253	1.4587593
H	0.2621474	-4.8870661	3.5264844
H	-0.7621259	-6.2473827	1.7113122
N	0.9050971	-2.6963803	4.7645745
C	0.3997199	-1.5397874	6.9489917
C	1.1255753	-2.3026384	8.0751506
H	0.4523246	-2.4199220	8.9305213
H	1.3871524	-3.3142357	7.7402747
C	3.0514718	-0.8634692	7.2754358
H	2.0747615	-0.6741091	9.1654292
C	3.4228645	0.4824531	7.3383622
O	3.9158773	0.9604995	6.1163824
O	3.3963812	1.2390479	8.3397784
C	3.7466283	2.3563018	5.8849140
H	4.2933686	2.5842091	4.9654258
H	2.6853179	2.5969951	5.7433447
H	4.1464931	2.9522213	6.7114634
C	2.7696029	-1.6193637	6.0042024
H	2.9064002	-2.6918907	6.1972688
C	3.7672819	-1.3935268	4.8787526
O	3.2198036	-0.8515701	3.7796857
O	4.9336249	-1.7409827	4.9434401
C	4.0996396	-0.6328293	2.6597363
H	3.4772292	-0.1867764	1.8824971
H	4.9096198	0.0455426	2.9423716
H	4.5226355	-1.5816963	2.3162568
H	0.3883959	-0.4849822	7.2467426
C	2.3747409	-1.4726331	8.4729188
P	3.6311558	-2.4383568	9.4339985
C	2.9221742	-3.3891386	10.8271206
H	3.7156872	-3.8772694	11.4042194
H	2.3684156	-2.7080408	11.4828944
H	2.2362722	-4.1523758	10.4436476
C	4.8298859	-1.2477387	10.1038034
H	5.7074208	-1.7714473	10.4963528
H	5.1204442	-0.5650678	9.3009734
H	4.3553049	-0.6685525	10.9026235
C	4.5109437	-3.6015821	8.3430535
H	5.3418865	-4.0621620	8.8881538
H	3.8308690	-4.3889588	8.0008210
H	4.8959448	-3.0466978	7.4813605
C	-3.6994685	-2.7125807	6.1866970
C	-3.3361618	-1.3645298	6.1973876
C	-2.0136592	-0.9992899	6.4617580
C	-1.0350140	-1.9705490	6.7135711
C	-1.4106533	-3.3211531	6.6936898
C	-2.7315582	-3.6897639	6.4370428
H	-4.7287284	-3.0011181	5.9834820
H	-4.0809410	-0.5955299	6.0025852
H	-1.7346308	0.0529523	6.4679722
H	-0.6634531	-4.0915023	6.8616706
H	-3.0055932	-4.7426871	6.4256070

3.4.4.36. **int3 *syn trans-trans* chair**
E(B3LYP-D3/6-31+G*(THF)) = -1894.494202
E(M06-2X-D3/6-311+G**) = -1894.157239
Gtot(B3LYP-D3/6-31+G*(THF)) = -1894.031097

C	0.6725073	-2.1232758	3.5311656
C	0.3470599	-2.2842191	5.6486382
C	0.8026865	-3.5582188	5.2407414
O	0.9945968	-3.4233809	3.8356101
O	1.0620056	-4.6460540	5.7936663
C	1.0722460	-0.7043206	-0.4767598
C	1.2962890	-2.0538068	-0.1866570
C	1.1732574	-2.5291416	1.1191626
C	0.8203439	-1.6557879	2.1657895
C	0.6038414	-0.2940045	1.8668594
C	0.7262424	0.1710082	0.5614994
H	1.1649015	-0.3379794	-1.4966401
H	1.5646263	-2.7436790	-0.9843958
H	1.3410701	-3.5797989	1.3376853
H	0.3502845	0.3833231	2.6766045
H	0.5516941	1.2241835	0.3494552
N	0.2624051	-1.4359786	4.5671732
C	-0.0747272	-1.8428990	7.0238524
C	1.0236612	-2.1682789	8.0722442
H	0.5955600	-2.1255873	9.0827154
H	1.3865825	-3.1868888	7.9047410
C	2.8504689	-1.0187245	6.6162085
H	1.7812090	-0.1624531	8.2348269
C	3.5019710	-2.2570778	6.0946828
O	3.5347526	-2.3389431	4.7718597
O	3.9553500	-3.1123395	6.8518655

C	4.0770196	-3.5553133	4.2107132
H	3.9817512	-3.4345045	3.1322424
H	5.1254616	-3.6701433	4.4996787
H	3.4879805	-4.4065088	4.5585045
C	2.8531648	0.1791115	5.9916248
H	2.2744459	0.9945805	6.4212335
C	3.5714443	0.5459965	4.7425272
O	2.8332054	1.4253315	4.0340179
O	4.6900566	0.1855492	4.4211443
C	3.4182130	1.8929909	2.7988705
H	2.7227292	2.6394483	2.4118890
H	4.3990483	2.3411589	2.9836456
H	3.5183669	1.0643347	2.0933161
H	-0.1767871	-0.7506667	6.9715453
C	2.1890176	-1.1504002	7.9814788
P	3.4053318	-1.4422941	9.3674740
C	2.8224045	-0.4295018	10.7685874
H	3.4730213	-0.5900148	11.6356332
H	2.8392441	0.6312254	10.4949249
H	1.7985114	-0.7164058	11.0326297
C	5.0727405	-0.8681624	8.9250828
H	5.7105367	-0.8920345	9.8157241
H	5.4861219	-1.5242890	8.1551016
H	5.0178307	0.1576228	8.5459271
C	3.4658145	-3.1667848	9.9427759
H	4.2021509	-3.2366027	10.7517251
H	2.4845473	-3.4634979	10.3266305
H	3.7542263	-3.8170490	9.1155153
C	-3.9473280	-3.3824508	8.2512881
C	-3.7180584	-2.0060975	8.2009247
C	-2.4672868	-1.5190135	7.8086844
C	-1.4313683	-2.3934509	7.4563139
C	-1.6726753	-3.7755897	7.5074893
C	-2.9186378	-4.2643507	7.9026484
H	-4.9200952	-3.7659771	8.5523329
H	-4.5129573	-1.3095717	8.4612801
H	-2.2983207	-0.4439770	7.7638274
H	-0.8835459	-4.4613042	7.2112215
H	-3.0916570	-5.3384717	7.9306571

3.4.4.37. **int3** ***syn trans-trans*** **boat**
E(B3LYP-D3/6-31+G*(THF)) = -1894.495256
E(M06-2X-D3/6-311+G**) = -1894.190127
Gtot(B3LYP-D3/6-31+G*(THF)) = -1894.033208

C	0.3733196	-1.2573082	3.5822396
C	1.4359331	-1.2515939	5.5234566
C	1.5908915	-2.6156615	4.8421307
O	0.8589727	-2.5672397	3.6811065
O	2.1856677	-3.6119314	5.1793590
C	-2.1320383	-0.4072822	0.2438191
C	-1.6436577	-1.7030383	0.4292879
C	-0.8092567	-1.9897364	1.5097557
C	-0.4625561	-0.9739687	2.4147354
C	-0.9542427	0.3289976	2.2255460
C	-1.7837016	0.6076943	1.1429407
H	-2.7841365	-0.1870078	-0.5986797
H	-1.9148746	-2.4933414	-0.2668049
H	-0.4352155	-2.9981647	1.6567395
H	-0.6826285	1.1083119	2.9319411
H	-2.1628999	1.6167119	0.9991666
N	0.6858329	-0.4712169	4.5390463
C	0.5566442	-1.4273208	6.8205350
C	1.3669241	-2.1761329	7.8933283
H	0.7125660	-2.4189210	8.7374256
H	1.7063641	-3.1268209	7.4753763
C	2.9995704	-0.2900300	7.3626189
H	2.2633615	-0.7574222	9.2784942
C	3.4439036	0.9340385	7.8722338
O	3.7942915	1.8630032	6.8854781
O	3.5624220	1.2527175	9.0864031
C	4.1832292	3.1478156	7.3626072
H	4.4347690	3.7347884	6.4741362
H	3.3727603	3.6424816	7.9116297
H	5.0557287	3.0846711	8.0230144
C	2.8133333	-0.5395708	5.8985442
H	2.7542582	0.4301941	5.3939482
C	3.9764584	-1.2488738	5.1867432
O	3.7544814	-1.2864765	3.8534757
O	4.9801633	-1.7093313	5.6997025
C	4.7085949	-2.0189440	3.0545109
H	4.3646190	-1.9212579	2.0237029
H	5.7096615	-1.5937579	3.1663828
H	4.7192023	-3.0702437	3.3568482
H	0.3849388	-0.4017820	7.1662332
C	2.5542408	-1.3000862	8.3692259
P	3.9632082	-2.3626940	9.0195868
C	3.6204216	-2.8689105	10.7416838
H	4.4395375	-3.4826997	11.1320087
H	3.5068796	-1.9757253	11.3658573
H	2.6913388	-3.4486979	10.7752160
C	5.4979130	-1.3974303	8.9964998
H	6.2940911	-1.9441869	9.5122560
H	5.7596358	-1.2275072	7.9495626
H	5.3062749	-0.4336862	9.4759784
C	4.2227866	-3.8876507	8.0505911
H	5.1968088	-4.3200950	8.3038737
H	3.4377973	-4.6156349	8.2778042
H	4.1980936	-3.6431932	6.9863210
C	-3.3019069	-3.1514794	5.8371791
C	-3.1138741	-1.7669603	5.8316370
C	-1.8709365	-1.2293650	6.1660729
C	-0.7923963	-2.0579099	6.5103772
C	-0.9940067	-3.4460259	6.5119349
C	-2.2381077	-3.9883602	6.1791954
H	-4.2700332	-3.5741985	5.5769957
H	-3.9350644	-1.1047796	5.5658773
H	-1.7251966	-0.1519003	6.1485467
H	-0.1773664	-4.1171406	6.7630495
H	-2.3741697	-5.0678031	6.1856868

3.4.4.38. TSelim *anti cis-cis*
E(B3LYP-D3/6-31+G*(THF)) = -1894.486171
E(M06-2X-D3/6-311+G**) = -1894.175185
Gtot(B3LYP-D3/6-31+G*(THF)) = -1894.027279

C	-1.1865073	1.8552355	7.1010666
C	0.4581526	0.5311300	7.7440832
C	-0.5305115	0.7237407	8.9016485
O	-1.5369028	1.5414605	8.4136957

O	-0.5804617	0.2617585	10.0110548
C	-3.9083447	4.2572579	4.8761655
C	-4.2142869	3.9120049	6.1951210
C	-3.3275242	3.1338444	6.9400857
C	-2.1271420	2.6970447	6.3596642
C	-1.8214728	3.0447177	5.0326515
C	-2.7098534	3.8230195	4.2972176
H	-4.6024182	4.8632639	4.2979428
H	-5.1459112	4.2474151	6.6444969
H	-3.5657320	2.8607350	7.9636223
H	-0.8902782	2.6970959	4.5947920
H	-2.4709112	4.0910897	3.2707531
N	-0.0927429	1.3545992	6.6717062
C	0.4804657	-0.9743160	7.2951430
H	1.1813855	-0.9864130	6.4479657
C	1.0659150	-1.8479309	8.4116079
H	0.4138760	-1.8273121	9.2911334
H	1.1065533	-2.8895137	8.0706927
C	2.6178387	0.0519019	9.0388599
H	2.8168891	-1.9501946	9.7005991
C	3.5981127	0.5868036	9.9228869
O	4.2522711	-0.3858931	10.6722637
O	3.8953182	1.7813240	10.0774223
C	5.1836991	0.1120062	11.6382610
H	5.6334607	-0.7703937	12.1007726
H	5.9586369	0.7245852	11.1661884
H	4.6791704	0.7168269	12.4001371
C	1.9327193	0.9689304	8.0443693
H	2.4126320	0.9088426	7.0557100
C	1.9988159	2.4581410	8.3808689
O	1.2043332	2.7621186	9.4225766
O	2.6163694	3.2827023	7.7356629
C	1.2346068	4.1317586	9.8658922
H	0.5186742	4.1913219	10.6868872
H	2.2404814	4.3821102	10.2146224
H	0.9484532	4.8069127	9.0543376
C	-3.3481324	-2.2485319	5.7051434
C	-2.3383093	-1.7875408	4.8562398
C	-1.1101368	-1.3904862	5.3848633
C	-0.8634031	-1.4476921	6.7645398
C	-1.8851177	-1.9086066	7.6073893
C	-3.1174555	-2.3060711	7.0811930
H	-4.3075678	-2.5588700	5.2965767
H	-2.5078269	-1.7346216	3.7826656
H	-0.3332276	-1.0166723	4.7210940
H	-1.7300723	-1.9541460	8.6819845
H	-3.8985480	-2.6611626	7.7503306
C	2.4341435	-1.3566399	8.8706051
P	3.8531588	-2.1346705	7.4234497
C	3.5015416	-3.3915791	6.1148422
H	4.4274177	-3.7124269	5.6200408
H	3.0203549	-4.2653690	6.5674396
H	2.8244317	-2.9677755	5.3658696
C	5.1448445	-2.8954700	8.4806404
H	6.0371486	-3.1573269	7.9012183
H	5.4047296	-2.1808778	9.2672423
H	4.7406964	-3.7990665	8.9512465
C	4.6846062	-0.7418693	6.5702850
H	5.6856055	-1.0346825	6.2351579
H	4.0949087	-0.4230525	5.7047566
H	4.7712773	0.0964138	7.2697089

3.4.4.39. TSelim *anti cis-trans*
E(B3LYP-D3/6-31+G*(THF)) = -1894.494387
E(M06-2X-D3/6-311+G**) = -1894.188468
Gtot(B3LYP-D3/6-31+G*(THF)) = -1894.035259

C	-1.6673802	1.5735704	7.1830827
C	0.1008648	0.3425629	7.6782176
C	-0.6893919	0.6473879	8.9542830
O	-1.7862290	1.3951761	8.5654228
O	-0.5029727	0.3486891	10.1051854
C	-4.8319102	3.6243292	5.2048244
C	-4.8587332	3.4960481	6.5960778
C	-3.8247193	2.8347282	7.2599593
C	-2.7559062	2.2986022	6.5254494
C	-2.7316323	2.4277641	5.1260418
C	-3.7660573	3.0892092	4.4714999
H	-5.6413140	4.1376953	4.6902287
H	-5.6874314	3.9095110	7.1658563
H	-3.8473849	2.7313683	8.3407291
H	-1.9048018	1.9976187	4.5688545
H	-3.7465154	3.1849141	3.3883603
N	-0.6363021	1.0571667	6.6376330
C	0.1007059	-1.1908905	7.3460575
H	0.6631652	-1.2570766	6.4093240
C	0.8760778	-1.9700795	8.4168325
H	0.3738535	-1.8880378	9.3886325
H	0.8828538	-3.0342500	8.1545989
C	2.4709350	-0.0334643	8.5952918
H	2.8550374	-1.9455544	9.3686865
C	3.6439390	0.4777309	9.2479007
O	3.7575838	1.8441599	9.1464498
O	4.5021460	-0.1862902	9.8519656
C	4.9073298	2.4240499	9.7735467
H	4.8351294	3.4988154	9.5906641
H	4.9137354	2.2257589	10.8506841
H	5.8320890	2.0293480	9.3391181
C	1.5667553	0.8868155	7.7993550
H	1.5146822	1.8662447	8.2812219
C	2.0777262	1.1337135	6.3762126
O	2.1185840	2.4405780	6.0788706
O	2.4118304	0.2613769	5.5892800
C	2.5228528	2.7736633	4.7319065
H	2.4587315	3.8608379	4.6660487
H	3.5471657	2.4385654	4.5441407
H	1.8495365	2.3040964	4.0091926
C	-3.8994418	-2.6385072	6.5097792
C	-3.0253727	-2.3116057	5.4697121
C	-1.7386577	-1.8556210	5.7572201
C	-1.2982379	-1.7206545	7.0814881
C	-2.1844707	-2.0499882	8.1170864
C	-3.4750888	-2.5046006	7.8335199
H	-4.9039247	-2.9940174	6.2898276
H	-3.3463768	-2.4088931	4.4345502
H	-1.0665944	-1.5874532	4.9448416
H	-1.8751670	-1.9494533	9.1544228
H	-4.1493007	-2.7545152	8.6499849
C	2.2776678	-1.4268111	8.6037305
P	3.5024875	-2.5037668	6.9754646

C	4.1974964	-4.0714885	7.6613985
H	4.9687642	-4.4885663	7.0019122
H	4.6410466	-3.8736797	8.6437094
H	3.3973464	-4.8103421	7.7830548
C	4.9732439	-1.4429136	6.6991327
H	5.7168105	-1.9547350	6.0776958
H	4.6482960	-0.5254846	6.2026489
H	5.4071444	-1.1832552	7.6689717
C	2.9419902	-2.9862211	5.2814666
H	3.7609603	-3.4445860	4.7123223
H	2.1166477	-3.7026725	5.3545685
H	2.5940844	-2.0893468	4.7631890

3.4.4.40. TSelim *anti trans-cis*
E(B3LYP-D3/6-31+G*(THF)) = -1894.494317
E(M06-2X-D3/6-311+G**) = -1894.189817
Gtot(B3LYP-D3/6-31+G*(THF)) = -1894.036582

C	-1.5854765	1.1900288	8.7056629
C	0.1240427	0.2719006	7.6524758
C	-0.8223785	0.9274758	6.6373395
O	-1.8692854	1.4569599	7.3615616
O	-0.7794202	0.9914603	5.4358983
C	-4.5097172	2.3522904	11.5732418
C	-4.7491748	2.5787086	10.2152798
C	-3.7950559	2.2148566	9.2644186
C	-2.5933792	1.6187432	9.6764223
C	-2.3544640	1.3915015	11.0427551
C	-3.3099445	1.7591729	11.9850008
H	-5.2565164	2.6364274	12.3114940
H	-5.6813243	3.0377956	9.8947406
H	-3.9822752	2.3860483	8.2087362
H	-1.4207284	0.9276768	11.3468906
H	-3.1221100	1.5830928	13.0415143
N	-0.4924686	0.5737320	8.9412351
C	0.1337923	-1.2776122	7.3998083
H	0.6229820	-1.4043989	6.4281196
C	0.9937775	-1.9510151	8.4786717
H	0.5428930	-1.7780565	9.4625889
H	0.9960344	-3.0355243	8.3160527
C	2.5591009	0.0192129	8.4035920
H	3.0047605	-1.8115575	9.3362501
C	3.7396269	0.5997976	8.9579222
O	3.8221978	1.9620888	8.7487747
O	4.6430214	0.0033115	9.5751496
C	4.9800908	2.6044101	9.2907269
H	4.8905223	3.6590633	9.0187081
H	5.0201688	2.5023181	10.3810817
H	5.8991525	2.1847715	8.8673050
C	1.5837024	0.8522938	7.5967914
H	1.5291242	1.8622324	8.0087987
C	2.0111536	0.9985103	6.1330023
O	1.8884070	2.2650687	5.7061635
O	2.4085002	0.0905993	5.4207037
C	2.1342258	2.4875483	4.2992439
H	1.9857794	3.5563973	4.1400149
H	3.1553145	2.1989801	4.0365270
H	1.4237612	1.9072635	3.7049515
C	-3.8691827	-2.9113963	7.0577288
C	-3.0927520	-2.6863725	5.9183293
C	-1.8032703	-2.1646184	6.0427165
C	-1.2659479	-1.8602188	7.3021856
C	-2.0572550	-2.0881141	8.4389732
C	-3.3466377	-2.6091961	8.3179158
H	-4.8738696	-3.3186727	6.9650137
H	-3.4900857	-2.9161005	4.9317989
H	-1.2076495	-1.9820417	5.1505148
H	-1.6707580	-1.8491704	9.4249745
H	-3.9448195	-2.7780518	9.2108547
C	2.4007589	-1.3845923	8.5342018
P	3.5368377	-2.5458810	7.0046978
C	4.2570300	-4.0514998	7.7874545
H	4.9779727	-4.5384440	7.1199913
H	4.7653510	-3.7712352	8.7165443
H	3.4590272	-4.7625024	8.0270800
C	4.9829301	-1.5186238	6.5529830
H	5.6997404	-2.0938862	5.9557373
H	4.6300423	-0.6567204	5.9819909
H	5.4556277	-1.1622998	7.4729464
C	2.8277567	-3.1517221	5.4143660
H	3.5907721	-3.6699495	4.8201047
H	2.0055721	-3.8462551	5.6178011
H	2.4478757	-2.2946327	4.8534652

3.4.4.41. TSelim *anti trans-trans*
E(B3LYP-D3/6-31+G*(THF)) = -1894.486720
E(M06-2X-D3/6-311+G**) = -1894.177491
Gtot(B3LYP-D3/6-31+G*(THF)) = -1894.027399

C	-1.6100506	1.5398661	7.1973192
C	0.1673717	0.3118556	7.6481384
C	-0.5503793	0.6742418	8.9512918
O	-1.6654113	1.4089677	8.5827198
O	-0.3366790	0.3870274	10.0993857
C	-4.8493873	3.5801949	5.3322762
C	-4.8232321	3.4529493	6.7234640
C	-3.7621531	2.7969719	7.3476167
C	-2.7193739	2.2638991	6.5742119
C	-2.7480557	2.3927229	5.1752607
C	-3.8093255	3.0497580	4.5600528
H	-5.6797823	4.0896420	4.8484935
H	-5.6317108	3.8624344	7.3241266
H	-3.7438599	2.6925739	8.4280524
H	-1.9395183	1.9697578	4.5863812
H	-3.8303337	3.1463180	3.4771969
N	-0.6143770	0.9885603	6.6148896
C	0.1194266	-1.2396458	7.4039430
H	0.6116020	-1.3695957	6.4295584
C	0.9537515	-1.9738102	8.4610254
H	0.4826830	-1.8754828	9.4456502
H	0.9746408	-3.0439376	8.2238708
C	2.5306313	0.0074091	8.5750388
H	2.9055461	-1.8767938	9.4283776
C	3.5985158	0.5579242	9.3532703
O	3.7432761	1.9151834	9.2010930
O	4.3544240	-0.0686653	10.1186401
C	4.7481299	2.5398134	10.0048264
H	4.7347386	3.5960698	9.7258860
H	4.5261449	2.4301712	11.0718928
H	5.7355917	2.1109838	9.8049050

C	1.6668238	0.7636626	7.5777279
H	1.9549347	0.4928648	6.5514810
C	1.7678400	2.2872589	7.5631814
O	1.0466620	2.8493895	8.5471417
O	2.3622111	2.9220617	6.7140029
C	1.0433374	4.2901136	8.5940516
H	0.3940556	4.5560997	9.4290759
H	2.0576467	4.6629286	8.7606303
H	0.6552825	4.7011790	7.6577544
C	-3.9610301	-2.6337591	6.9299419
C	-3.1759427	-2.3294567	5.8148590
C	-1.8607640	-1.8959705	5.9843974
C	-1.3031430	-1.7594139	7.2638282
C	-2.1018600	-2.0644637	8.3757240
C	-3.4197078	-2.4983726	8.2098230
H	-4.9872499	-2.9715741	6.8020904
H	-3.5887490	-2.4258819	4.8128093
H	-1.2597394	-1.6443075	5.1131706
H	-1.7053873	-1.9570296	9.3816715
H	-4.0235867	-2.7291447	9.0848964
C	2.3553938	-1.4047902	8.6142519
P	3.6040944	-2.2983565	7.0350452
C	3.3197320	-3.8352821	6.0406704
H	4.2147501	-4.0958773	5.4603852
H	3.0770938	-4.6644309	6.7133811
H	2.4801371	-3.6853016	5.3538394
C	5.1510425	-2.6220657	7.9684157
H	5.9826797	-2.8489677	7.2919432
H	5.3804324	-1.7387273	8.5721325
H	4.9958181	-3.4686641	8.6462060
C	4.0924912	-1.0270168	5.7999441
H	5.0519565	-1.2931661	5.3423513
H	3.3340392	-0.9407321	5.0158307
H	4.1991442	-0.0616338	6.3051865

3.4.4.42. TSelim *syn cis-cis*
E(B3LYP-D3/6-31+G*(THF)) = -1894.492107
E(M06-2X-D3/6-311+G**) = -1894.185948
Gtot(B3LYP-D3/6-31+G*(THF)) = -1894.031963

C	0.8524361	-2.7162399	3.9689099
C	1.0228918	-1.2314535	5.5960892
C	0.0701131	-0.7155791	4.5163105
O	-0.0252901	-1.7106866	3.5655044
O	-0.5425465	0.3160320	4.4347639
C	1.0379882	-6.1943942	1.5216886
C	0.1801015	-5.1491208	1.1702086
C	0.1164697	-3.9990608	1.9571343
C	0.9162810	-3.8953533	3.1054697
C	1.7777394	-4.9479959	3.4572151
C	1.8378194	-6.0912437	2.6663006
H	1.0837620	-7.0901794	0.9062203
H	-0.4430457	-5.2297913	0.2827916
H	-0.5530471	-3.1882765	1.6873344
H	2.3876218	-4.8538105	4.3505591
H	2.5053706	-6.9048574	2.9399927
N	1.4900079	-2.5106876	5.0582891
C	0.2198827	-1.4646959	6.9222678
C	1.1786446	-2.0580207	7.9738699
H	0.6269233	-2.2304471	8.9046573
H	1.5093330	-3.0363110	7.6180317
C	2.6124173	-0.0907414	7.2879057
H	2.4124395	-0.7505354	9.2637303
C	3.2729749	1.0889616	7.7514385
O	3.4404810	2.0283996	6.7532982
O	3.6682634	1.3194380	8.9074342
C	4.0491817	3.2597193	7.1527333
H	4.1033452	3.8716134	6.2489947
H	3.4500604	3.7711401	7.9142647
H	5.0544472	3.0919522	7.5538786
C	2.1818105	-0.1962864	5.8477393
H	1.7759514	0.7741502	5.5389115
C	3.3030118	-0.4951220	4.8443992
O	2.8604733	-0.2877805	3.5857743
O	4.4283993	-0.8789666	5.0956287
C	3.7624657	-0.6438282	2.5149811
H	3.2215897	-0.4294820	1.5920537
H	4.6784740	-0.0486595	2.5694573
H	4.0148498	-1.7067473	2.5740148
H	-0.0922760	-0.4671191	7.2520269
C	2.3567198	-1.1116201	8.2389408
P	4.1373513	-2.4426592	8.4598696
C	4.4934379	-3.3886569	10.0079739
H	5.4523025	-3.9184385	9.9383736
H	4.5303463	-2.6977765	10.8577368
H	3.6948796	-4.1175571	10.1846935
C	5.5768969	-1.3372563	8.2257912
H	6.5139172	-1.9033361	8.2576019
H	5.4668423	-0.8570263	7.2503688
H	5.5723217	-0.5654603	8.9993304
C	4.2544029	-3.6810940	7.1053102
H	5.2721611	-4.0813155	7.0264113
H	3.5604027	-4.5066536	7.2963264
H	3.9770351	-3.1943675	6.1666484
C	-3.3856514	-3.7879762	6.2568523
C	-3.4490169	-2.4026743	6.4207544
C	-2.2811953	-1.6700826	6.6426278
C	-1.0308711	-2.3033057	6.7051732
C	-0.9815285	-3.6966798	6.5386418
C	-2.1474472	-4.4317166	6.3166938
H	-4.2933449	-4.3621628	6.0838104
H	-4.4076736	-1.8903252	6.3762465
H	-2.3375849	-0.5902835	6.7634115
H	-0.0274856	-4.2136050	6.5679704
H	-2.0870060	-5.5102453	6.1878370

3.4.4.43. TSelim *syn cis-trans*
E(B3LYP-D3/6-31+G*(THF)) = -1894.484824
E(M06-2X-D3/6-311+G**) = -1894.178517
Gtot(B3LYP-D3/6-31+G*(THF)) = -1894.026456

C	0.2472665	-1.6627113	3.6673386
C	1.2613047	-1.1636463	5.5705194
C	1.1689994	-2.6834387	5.4093406
O	0.5053988	-2.9232361	4.2309087
O	1.5506414	-3.5858835	6.1150196
C	-2.0008325	-1.5578228	0.0559849
C	-1.6455614	-2.7913519	0.6082047
C	-0.9008796	-2.8442748	1.7870245
C	-0.5088626	-1.6528800	2.4159169

C	-0.8678702	-0.4132929	1.8594730
C	-1.6100247	-0.3690246	0.6837159
H	-2.5839636	-1.5207541	-0.8615547
H	-1.9505587	-3.7144999	0.1213651
H	-0.6278075	-3.8025987	2.2186385
H	-0.5664149	0.4995970	2.3638771
H	-1.8890942	0.5912926	0.2565929
N	0.6720786	-0.6611734	4.3297834
C	0.4002812	-0.6609376	6.7789884
C	0.9105644	-1.2700133	8.0954279
H	0.4060633	-0.7569959	8.9230467
H	0.6063240	-2.3170234	8.1582415
C	3.1715634	-0.5186567	7.1828379
H	2.7043413	-0.6682572	9.2152748
C	4.4014678	0.1120779	7.5247752
O	5.1050507	0.5439817	6.4163627
O	4.8628058	0.2974331	8.6683776
C	6.2858924	1.3056402	6.6784488
H	6.6997759	1.5586808	5.6994610
H	6.0525812	2.2208429	7.2337214
H	7.0118450	0.7228771	7.2550803
C	2.7626126	-0.6989890	5.7306530
H	3.3841733	-1.4415480	5.2098772
C	2.9327962	0.5643592	4.8715181
O	2.3852411	1.6479144	5.4476115
O	3.4573792	0.5744815	3.7747914
C	2.4165300	2.8546226	4.6613700
H	1.9421713	3.6211270	5.2768263
H	3.4483932	3.1338602	4.4295087
H	1.8624495	2.7143265	3.7283523
H	0.5900858	0.4165599	6.7984287
C	2.4293486	-1.1059697	8.2557998
P	3.2767614	-3.0418522	8.7712166
C	2.3177852	-4.5673964	9.1651544
H	2.9887537	-5.3930247	9.4339246
H	1.6440943	-4.3680334	10.0056015
H	1.7264060	-4.8506314	8.2902972
C	4.3139406	-2.7112877	10.2495353
H	4.9461884	-3.5704540	10.5010263
H	4.9333091	-1.8356520	10.0314174
H	3.6681773	-2.4768901	11.1034949
C	4.4639390	-3.5146482	7.4603645
H	5.2279467	-4.1926549	7.8552775
H	3.9259747	-3.9973255	6.6417414
H	4.9463702	-2.6074825	7.0822889
C	-3.8516875	-1.1976844	6.0968277
C	-3.2666332	0.0675329	6.0049897
C	-1.8991095	0.2239408	6.2349154
C	-1.0907629	-0.8720862	6.5657174
C	-1.6912373	-2.1368561	6.6591584
C	-3.0584344	-2.2994768	6.4238167
H	-4.9168467	-1.3240643	5.9148520
H	-3.8747558	0.9332299	5.7508660
H	-1.4469077	1.2096326	6.1497071
H	-1.0961838	-3.0109374	6.9119447
H	-3.5030297	-3.2897613	6.4969676

3.4.4.44. TSelim *syn trans-cis*
E(B3LYP-D3/6-31+G*(THF)) = -1894.481557
E(M06-2X-D3/6-311+G**) = -1894.173717
Gtot(B3LYP-D3/6-31+G*(THF)) = -1894.021944

C	0.3215285	-2.2449600	3.7241333
C	1.1628226	-1.4924116	5.6200669
C	0.7284922	-0.3142152	4.7465600
O	0.1910044	-0.8632077	3.5925442
O	0.7107580	0.8693063	4.9567971
C	-1.2707852	-4.6641187	0.5997429
C	-1.3188433	-3.2722517	0.4858318
C	-0.7915975	-2.4665661	1.4959519
C	-0.2115029	-3.0576082	2.6284482
C	-0.1645173	-4.4578616	2.7405426
C	-0.6915829	-5.2550135	1.7290289
H	-1.6857228	-5.2892037	-0.1881068
H	-1.7703487	-2.8124657	-0.3900598
H	-0.8339191	-1.3849462	1.4112358
H	0.2805562	-4.9026445	3.6255876
H	-0.6557081	-6.3381976	1.8203391
N	0.8629119	-2.6640155	4.8022159
C	0.2826094	-1.4458224	6.9390265
C	1.0323096	-2.1144204	8.1144287
H	0.3715224	-2.1868069	8.9844142
H	1.3027480	-3.1402985	7.8394187
C	2.9367789	-0.7432230	7.2986363
H	2.1131472	-0.5394081	9.2395434
C	3.5587081	0.5389631	7.3655344
O	3.9391428	1.0058658	6.1321677
O	3.7506574	1.2166254	8.3900916
C	4.5576403	2.2942646	6.1067055
H	4.7823506	2.4941854	5.0566177
H	3.8837402	3.0637598	6.4977805
H	5.4807038	2.3009474	6.6966821
C	2.6766823	-1.5303800	6.0349531
H	2.8149602	-2.5929061	6.2781976
C	3.6945057	-1.3480240	4.9178294
O	3.1689931	-0.8417772	3.7938936
O	4.8575411	-1.6950185	5.0209059
C	4.0671609	-0.6787793	2.6767833
H	3.4650949	-0.2388084	1.8807467
H	4.8922360	-0.0153282	2.9497709
H	4.4666180	-1.6492985	2.3677066
H	0.2031447	-0.3805248	7.1824656
C	2.2619076	-1.2609728	8.4366240
P	3.6779024	-2.5610108	9.5395365
C	3.3811061	-3.8364529	10.8511075
H	4.3282054	-4.2492731	11.2225339
H	2.8373700	-3.3820020	11.6863198
H	2.7746707	-4.6512777	10.4405144
C	4.8282347	-1.3506354	10.2982184
H	5.7912318	-1.8141002	10.5401499
H	4.9701085	-0.5202954	9.6006172
H	4.3789161	-0.9519222	11.2145626
C	4.6731098	-3.4384676	8.2676880
H	5.6195364	-3.7861931	8.6970259
H	4.1191161	-4.3048914	7.8897828
H	4.8849065	-2.7582773	7.4357728
C	-3.7249812	-2.9053470	6.1792411
C	-3.4502922	-1.5370380	6.1540196
C	-2.1566292	-1.0784115	6.4167276
C	-1.1190923	-1.9754910	6.7029608

C	-1.4065420	-3.3485039	6.7197620
C	-2.6979215	-3.8098378	6.4643319
H	-4.7312250	-3.2662599	5.9769235
H	-4.2417877	-0.8243940	5.9320709
H	-1.9479388	-0.0101301	6.3957490
H	-0.6133182	-4.0646163	6.9136516
H	-2.9021856	-4.8784145	6.4811944

3.4.4.45. TSelim *syn trans-trans*
E(B3LYP-D3/6-31+G*(THF)) = -1894.488353
E(M06-2X-D3/6-311+G**) = -1894.182890
Gtot(B3LYP-D3/6-31+G*(THF)) = -1894.028053

C	0.3691391	-1.2781721	3.6015989
C	1.3713036	-1.1828861	5.5726979
C	1.5952066	-2.5590785	4.9326414
O	0.8928917	-2.5666780	3.7526836
O	2.2142243	-3.5222921	5.3129098
C	-2.0485770	-0.6118580	0.1605480
C	-1.4715124	-1.8676209	0.3667159
C	-0.6665539	-2.0938435	1.4831420
C	-0.4382434	-1.0568770	2.4018050
C	-1.0185719	0.2056973	2.1918364
C	-1.8187036	0.4240383	1.0741622
H	-2.6783838	-0.4393838	-0.7095023
H	-1.6506391	-2.6739240	-0.3410480
H	-0.2237119	-3.0715932	1.6463507
H	-0.8392255	1.0012751	2.9097373
H	-2.2677506	1.4015342	0.9143423
N	0.6309882	-0.4531636	4.5407971
C	0.4465036	-1.3552705	6.8385284
C	1.2507936	-2.0225237	7.9708150
H	0.5893161	-2.2206173	8.8210773
H	1.6111331	-2.9920782	7.6202888
C	2.9037902	-0.2041827	7.4340291
H	2.2836227	-0.6682445	9.3858363
C	3.6077686	0.9437538	7.9151447
O	4.0513684	1.7669818	6.8990975
O	3.8267634	1.2352077	9.1041210
C	4.7440381	2.9486098	7.3122335
H	5.0381105	3.4586504	6.3915414
H	4.0980937	3.6009027	7.9102659
H	5.6316826	2.6996150	7.9034524
C	2.7071013	-0.4231721	5.9552606
H	2.5984781	0.5558612	5.4765917
C	3.9073597	-1.0513737	5.2319220
O	3.6735788	-1.1023199	3.9019389
O	4.9389712	-1.4474011	5.7374087
C	4.6754220	-1.7570948	3.0915665
H	4.3200541	-1.6739318	2.0632626
H	5.6436356	-1.2616977	3.2043582
H	4.7641219	-2.8077696	3.3835544
H	0.2039022	-0.3299837	7.1386777
C	2.4018577	-1.1114699	8.3990679
P	4.0017229	-2.5367550	9.1010523
C	3.9491523	-3.2595365	10.8004165
H	4.8598944	-3.8322450	11.0183149
H	3.8499698	-2.4539886	11.5367422
H	3.0816004	-3.9227438	10.8888245
C	5.5607226	-1.5811711	9.0264387
H	6.4199962	-2.2145571	9.2722192
H	5.6620377	-1.1991682	8.0073144
H	5.5009649	-0.7336937	9.7135240
C	4.2678429	-3.9718160	7.9812049
H	5.2224651	-4.4658849	8.1987589
H	3.4548262	-4.6958319	8.1009669
H	4.2676386	-3.6135608	6.9489930
C	-3.2826074	-3.3151739	5.7971444
C	-3.1736475	-1.9226014	5.7686212
C	-1.9711693	-1.3085631	6.1195555
C	-0.8553874	-2.0675875	6.5029175
C	-0.9780862	-3.4649942	6.5281101
C	-2.1812118	-4.0833613	6.1788795
H	-4.2188121	-3.7975012	5.5242884
H	-4.0250243	-1.3137361	5.4723827
H	-1.8872022	-0.2248916	6.0859282
H	-0.1318784	-4.0842624	6.8117779
H	-2.2553778	-5.1685081	6.2043455

3.4.5. Products

3.4.5.1. **5p** ***cis-cis***
E(B3LYP-D3/6-31+G*(THF)) = -1433.400275
E(M06-2X-D3/6-311+G**) = -1433.154773
Gtot(B3LYP-D3/6-31+G*(THF)) = -1433.049344

C	0.0082776	-0.0138457	5.3119067
C	-1.1593357	0.6294639	5.1640179
C	-2.0615439	0.9755447	6.3064361
C	-1.8048473	0.0698039	7.5218870
C	-0.2774498	0.0976584	7.8733267
C	0.5785578	-0.4000883	6.6603650
H	-1.4864170	0.8859911	4.1588752
H	-3.1091334	0.8759741	5.9970136
H	-1.9213151	2.0317974	6.5711763
H	-2.0088691	-0.9650657	7.2181442
H	0.6377790	-1.4925162	6.7390279
C	0.7447450	-0.4179433	4.0786822
O	1.6709508	-1.3613601	4.3431288
O	0.5243353	0.0045505	2.9541788
C	2.0040627	0.1413144	6.8298104
O	2.5685555	-0.3916675	7.9285275
O	2.5483040	0.9497082	6.1089773
C	-4.2973095	0.9190517	10.9693666
C	-4.1436712	-0.3898781	10.5071983
C	-3.3448389	-0.6483652	9.3911692
C	-2.6876487	0.3917368	8.7178071
C	-2.8506523	1.7033032	9.1916825
C	-3.6480801	1.9641325	10.3071544
H	-4.9186202	1.1245320	11.8385532
H	-4.6453844	-1.2114961	11.0139438
H	-3.2257708	-1.6711937	9.0397804
H	-2.3449824	2.5264220	8.6963997
H	-3.7607155	2.9867625	10.6605924
C	2.4311272	-1.8418435	3.2134387
H	3.1141427	-2.5900294	3.6185328
H	1.7656951	-2.2920009	2.4706738
H	2.9880641	-1.0203469	2.7532940
C	3.8749632	0.1147266	8.2896763
H	4.1473137	-0.4111195	9.2054252
H	4.5963733	-0.0955783	7.4954232

H	3.8237020	1.1935185	8.4613576
C	0.4105606	1.3286834	9.5678321
N	0.1177958	1.4290357	8.3268940
C	1.5174548	4.4918588	12.1884888
C	1.4171472	3.1893596	12.6852053
C	1.0617915	2.1412339	11.8364272
C	0.8043446	2.4001622	10.4810374
C	0.9054373	3.7108192	9.9829864
C	1.2618368	4.7503601	10.8362871
H	1.7939689	5.3066619	12.8540549
H	1.6144174	2.9890858	13.7355479
H	0.9796269	1.1294034	12.2213160
H	0.7023103	3.8974531	8.9324240
H	1.3399181	5.7636628	10.4496208
C	-0.0372452	-0.7930025	9.0974018
O	0.3312450	0.0473398	10.1226430
O	-0.1570762	-1.9813753	9.2359891

3.4.5.2. **5p** ***cis-trans***
E(B3LYP-D3/6-31+G*(THF)) = -1433.398195
E(M06-2X-D3/6-311+G**) = -1433.151302
Gtot(B3LYP-D3/6-31+G*(THF)) = -1433.045311

C	0.0031174	0.2625719	5.4895171
C	-1.1114139	1.0040235	5.3912588
C	-2.1269028	1.1553763	6.4779113
C	-1.9796371	0.1022122	7.5887803
C	-0.4696545	-0.0776860	7.9744506
C	0.3606699	-0.5331628	6.7306833
H	-1.2820786	1.5549910	4.4687926
H	-3.1375088	1.0915746	6.0534131
H	-2.0395997	2.1748056	6.8774594
H	-2.2600451	-0.8664295	7.1640102
C	0.9134907	0.2006874	4.3114839
O	1.9277910	-0.6641412	4.5169915
O	0.7760771	0.8310577	3.2744533
H	1.4212663	-0.4027269	6.9646115
C	-4.4272810	0.6956297	11.1179430
C	-4.3761467	-0.5477131	10.4830654
C	-3.5948954	-0.7159740	9.3395325
C	-2.8557972	0.3490760	8.8059089
C	-2.9158504	1.5934318	9.4508032
C	-3.6946012	1.7646618	10.5981174
H	-5.0331862	0.8302567	12.0113516
H	-4.9417339	-1.3882702	10.8797498
H	-3.5477100	-1.6896406	8.8566690
H	-2.3516066	2.4404995	9.0677611
H	-3.7273549	2.7364378	11.0857712
C	0.1139058	1.2133465	8.5655976
O	0.4230740	0.9343698	9.8791370
O	0.2929703	2.3007578	8.0819666
C	0.1124978	-0.4175245	10.0766087
N	-0.3465298	-1.0424474	9.0628169
C	0.6837040	-1.9643605	13.9994883
C	1.0550555	-0.6470535	13.7180428
C	0.8786710	-0.1277579	12.4354427
C	0.3276826	-0.9343517	11.4275034
C	-0.0491369	-2.2577513	11.7133627
C	0.1310512	-2.7681057	12.9951906
H	0.8219412	-2.3653682	15.0011088
H	1.4814868	-0.0217963	14.4986479
H	1.1640882	0.8969006	12.2171046
H	-0.4855924	-2.8665924	10.9272126
H	-0.1622632	-3.7917607	13.2152681
C	2.8654783	-0.8172040	3.4294952
H	3.5922723	-1.5547591	3.7724498
H	2.3516773	-1.1706765	2.5307394
H	3.3570820	0.1366641	3.2164242
C	0.1318966	-2.0344124	6.5023262
O	1.1493186	-2.7647447	6.9729523
O	-0.8552125	-2.5041533	5.9680917
C	0.9969904	-4.1988647	6.8582803
H	1.9097335	-4.6248554	7.2771706
H	0.1208783	-4.5302655	7.4226410
H	0.8838225	-4.4874951	5.8095161

3.4.5.3. **5p** ***trans-cis***
E(B3LYP-D3/6-31+G*(THF)) = -1433.396324
E(M06-2X-D3/6-311+G**) = -1433.153605
Gtot(B3LYP-D3/6-31+G*(THF)) = -1433.045828

C	0.0742080	-0.0099668	5.4324753
C	-1.1001162	0.6248450	5.2994796
C	-2.0997259	0.7959514	6.3970380
C	-1.8254360	-0.1002897	7.6154665
C	-0.2900100	-0.0701027	7.9393435
C	0.5316904	-0.6347009	6.7368362
H	-1.3416380	1.0624880	4.3330723
H	-3.1090061	0.5890634	6.0167578
H	-2.1045830	1.8562809	6.6834597
H	-2.0329927	-1.1367399	7.3291956
C	0.9557618	-0.1142262	4.2363798
O	2.0329541	-0.8880365	4.4816768
O	0.7471332	0.4110760	3.1534448
H	1.5803121	-0.3850426	6.9190213
C	-4.2232223	0.8002081	11.1148335
C	-4.1040136	-0.5117714	10.6510217
C	-3.3386232	-0.7853719	9.5157788
C	-2.6813987	0.2421586	8.8238155
C	-2.8098842	1.5571535	9.2992592
C	-3.5734069	1.8333256	10.4346736
H	-4.8180446	1.0170660	11.9995816
H	-4.6054401	-1.3241722	11.1726260
H	-3.2428948	-1.8105344	9.1646766
H	-2.3023174	2.3708963	8.7900787
H	-3.6590673	2.8580610	10.7893472
C	2.9434193	-1.0783190	3.3769431
H	3.7248000	-1.7404543	3.7526950
H	2.4238102	-1.5367436	2.5304929
H	3.3679908	-0.1188607	3.0667710
C	0.4635453	1.3102447	9.4868607
N	0.1365577	1.2912185	8.2514983
C	1.5190303	4.7351130	11.7833972
C	1.4891192	3.4788688	12.3947719
C	1.1565893	2.3454095	11.6524659
C	0.8530245	2.4710442	10.2878460
C	0.8841092	3.7356806	9.6746016
C	1.2168448	4.8611014	10.4220729
H	1.7758798	5.6169702	12.3662457
H	1.7225532	3.3810715	13.4522123

H	1.1268739	1.3697774	12.1277902
H	0.6426775	3.8201855	8.6190584
H	1.2386776	5.8386183	9.9461906
C	-0.0368447	-0.8405709	9.2417964
O	0.4019513	0.0862185	10.1620567
O	-0.1956555	-2.0002453	9.5176377
C	0.4311502	-2.1644731	6.6719506
O	1.5128001	-2.7421253	7.2087903
O	-0.5131700	-2.7744726	6.2078961
C	1.4705433	-4.1842514	7.3162541
H	2.4206914	-4.4713756	7.7687057
H	0.6336386	-4.4817406	7.9536703
H	1.3607309	-4.6384251	6.3278101

3.4.5.4. **5p** ***trans-trans***
E(B3LYP-D3/6-31+G*(THF)) = -1433.399327
E(M06-2X-D3/6-311+G**) = -1433.148004
Gtot(B3LYP-D3/6-31+G*(THF)) = -1433.043471

C	0.1180840	0.3017598	5.5052029
C	-1.0356831	0.9765628	5.3798278
C	-2.1022384	1.0448961	6.4254333
C	-1.9147999	0.0044367	7.5442191
C	-0.4064943	-0.0631860	7.9748402
C	0.4599926	-0.4993000	6.7526569
H	-1.2170794	1.5123047	4.4506788
H	-3.0854348	0.8971999	5.9594073
H	-2.1176276	2.0661332	6.8262346
H	-2.1192082	-0.9859412	7.1187876
H	0.2342304	-1.5589647	6.5903349
C	1.0305763	0.2729899	4.3267146
O	1.9890734	-0.6649091	4.4658497
O	0.9271807	0.9761195	3.3340488
C	1.9380597	-0.4021288	7.1411059
O	2.4724901	-1.6082924	7.3641889
O	2.5358720	0.6474493	7.2915624
C	-4.4974579	0.4591497	11.0014449
C	-4.3307951	-0.7869931	10.3924589
C	-3.5091013	-0.9123504	9.2712701
C	-2.8436372	0.1985807	8.7340972
C	-3.0228247	1.4460118	9.3518907
C	-3.8404922	1.5738827	10.4771779
H	-5.1337132	0.5603086	11.8780292
H	-4.8377060	-1.6629360	10.7918595
H	-3.3734131	-1.8877162	8.8089411
H	-2.5197650	2.3294442	8.9679537
H	-3.9620683	2.5486930	10.9443834
C	0.0309435	1.2765144	8.5949745
O	0.3664298	0.9978038	9.9067002
O	0.0473062	2.3953864	8.1580779
C	0.1706369	-0.3737728	10.0837023
N	-0.2301039	-1.0247430	9.0595274
C	0.8707263	-1.9332778	13.9811701
C	1.1170929	-0.5815250	13.7256740
C	0.8965052	-0.0571279	12.4518610
C	0.4280832	-0.8935490	11.4265880
C	0.1800771	-2.2524687	11.6857279
C	0.4018693	-2.7672716	12.9589862
H	1.0417995	-2.3378410	14.9763980
H	1.4796807	0.0664385	14.5199299
H	1.0838575	0.9937855	12.2531146
H	-0.1882247	-2.8877898	10.8857440
H	0.2068772	-3.8182892	13.1582237
C	2.9265676	-0.7881700	3.3742680
H	3.5874183	-1.6104895	3.6509187
H	2.3971338	-1.0156673	2.4446436
H	3.4921363	0.1403769	3.2540748
C	3.8552795	-1.6166891	7.7893366
H	4.1035604	-2.6651497	7.9587718
H	4.4918693	-1.1897470	7.0093020
H	3.9739933	-1.0383221	8.7096241

3.4.6. Alternative mechanisms

3.4.6.1. Addition via C3

3.4.6.1.1. Addition TS diastereomer 1

E(B3LYP-D3/6-31+G*(THF)) = -1894.478692
E(M06-2X-D3/6-311+G**) = -1894.161138
Gtot(B3LYP-D3/6-31+G*(THF)) = -1894.021213

C	-0.2915125	1.6159992	8.0770820
C	0.0367235	-0.3229192	7.1949169
C	-0.0077318	-0.5329072	8.6053709
O	-0.2329396	0.7548605	9.1499855
O	0.0674163	-1.5159767	9.3564594
C	-0.8963941	5.7856584	8.7483805
C	-0.8791527	4.9016696	9.8309708
C	-0.6819339	3.5358647	9.6254622
C	-0.4985450	3.0352364	8.3241490
C	-0.5158740	3.9321049	7.2374007
C	-0.7134272	5.2925548	7.4503468
H	-1.0507071	6.8497956	8.9121153
H	-1.0212350	5.2770440	10.8420098
H	-0.6712970	2.8511098	10.4684757
H	-0.3710898	3.5437098	6.2334946
H	-0.7241295	5.9736385	6.6019427
N	-0.1358671	1.0311996	6.9259447
C	0.2912721	-1.3533669	6.2313756
H	0.2320307	-2.3385380	6.6883686
C	2.3201547	-1.3840316	5.9054714
C	3.0467017	-0.3032955	8.0051734
C	2.8311685	-1.4376186	7.2960693
P	3.0220901	-2.9898850	8.2588327
C	4.5551882	-3.8712231	7.8088979
H	4.6578337	-4.7489344	8.4570568
H	4.5143222	-4.1823109	6.7649280
H	5.4074264	-3.2030006	7.9718779
C	3.2176584	-2.5592470	10.0227063
H	3.2839632	-3.5002914	10.5803074
H	4.1268637	-1.9789051	10.2025997
H	2.3332018	-2.0064714	10.3535808
C	1.5975189	-4.1267139	8.2673792
H	1.4018632	-4.5089221	7.2679055
H	1.8391411	-4.9538339	8.9449831
H	0.7412685	-3.5682280	8.6557743
H	3.3149319	-0.2892309	9.0534764
H	2.9753534	0.6755753	7.5447210
C	-1.4402153	-1.4662706	2.2855662
C	-1.1741566	-2.6429425	2.9909197
C	-0.6168542	-2.5809217	4.2673541
C	-0.3146629	-1.3468044	4.8710574

C	-0.5970021	-0.1698290	4.1543431
C	-1.1511170	-0.2328620	2.8752439
H	-1.8744144	-1.5100293	1.2889018
H	-1.4030313	-3.6097133	2.5473704
H	-0.4075058	-3.5036028	4.8057901
H	-0.3924974	0.7894351	4.6149874
H	-1.3621850	0.6895254	2.3374352
C	2.4989390	-2.5902612	5.0697201
O	2.4486029	-2.3430366	3.7500491
O	2.6072728	-3.7384539	5.5060941
C	2.4917758	-3.4885326	2.8787631
H	2.4463974	-3.0846972	1.8661989
H	3.4184591	-4.0504406	3.0273345
H	1.6347659	-4.1408476	3.0658225
C	2.5449135	-0.0582908	5.1836591
H	2.0212907	0.7591149	5.6834539
H	2.1166656	-0.1266589	4.1795846
C	3.9906902	0.3752074	5.0041938
O	4.8151219	-0.6590427	4.7654971
O	4.3632637	1.5355105	5.0312505
C	6.1965347	-0.3245394	4.5138801
H	6.6981408	-1.2737098	4.3192572
H	6.2774767	0.3365439	3.6458961
H	6.6346497	0.1705322	5.3856415

3.4.6.1.2. Addition TS diastereomer 2
E(B3LYP-D3/6-31+G*(THF)) = -1894.467475
E(M06-2X-D3/6-311+G**) = -1894.143315
Gtot(B3LYP-D3/6-31+G*(THF)) = -1894.008978

C	-0.4224734	1.2172074	9.0831932
C	-0.2599340	-0.2421220	7.5047240
C	-0.6509364	0.9743267	6.8712333
O	-0.7663490	1.9061421	7.9471839
O	-0.9000246	1.3253774	5.7149801
C	-0.3965044	3.2344442	12.8481770
C	-0.7192551	3.9350379	11.6822290
C	-0.7308541	3.2868823	10.4465900
C	-0.4180971	1.9182531	10.3600655
C	-0.0988094	1.2172241	11.5403006
C	-0.0870093	1.8707417	12.7686296
H	-0.3905915	3.7422408	13.8099125
H	-0.9664078	4.9931210	11.7344041
H	-0.9853490	3.8349049	9.5440013
H	0.1284902	0.1571216	11.4782914
H	0.1586940	1.3143923	13.6709180
N	-0.1145804	-0.0304935	8.8710615
C	-0.0119743	-1.4651259	6.8056742
H	-0.3073998	-1.3622127	5.7616748
C	2.0178679	-1.6284198	6.3658013
C	2.3192304	0.8174158	6.5183165
C	2.6443860	-0.4199738	6.9626945
P	3.8294128	-0.4482534	8.3514793
C	5.2548725	-1.5353931	8.0186730
H	5.9717538	-1.4298620	8.8409020
H	4.9453629	-2.5764585	7.9331329
H	5.7130448	-1.2226073	7.0764647
C	4.5722824	1.2121962	8.5307903
H	5.3502941	1.1370490	9.2989751
H	5.0259586	1.5387694	7.5907227
H	3.8311278	1.9470754	8.8587260
C	3.0764506	-0.8114658	9.9811932
H	3.3330706	-1.8209324	10.3063834
H	3.4478706	-0.0855233	10.7120916
H	1.9897881	-0.7213273	9.8817278
H	2.7295619	1.7353152	6.9215426
H	1.6250143	0.9549380	5.6964667
C	-1.2659162	-5.3085162	8.3073391
C	-1.2214375	-5.0509538	6.9350274
C	-0.8045612	-3.8030415	6.4708587
C	-0.4268849	-2.7830567	7.3628985
C	-0.4735939	-3.0574199	8.7422300
C	-0.8905082	-4.3045040	9.2062041
H	-1.5943130	-6.2789961	8.6739848
H	-1.5150513	-5.8208843	6.2245446
H	-0.7801400	-3.6113908	5.3997231
H	-0.1919061	-2.2798655	9.4419581
H	-0.9255471	-4.4937068	10.2777580
C	2.3628322	-2.9765337	6.8656284
O	2.5517245	-3.0131215	8.2101370
O	2.4035637	-3.9901616	6.1815101
C	2.7627801	-4.3211560	8.7811051
H	2.8736207	-4.1608475	9.8553262
H	1.8963994	-4.9549016	8.5802099
H	3.6635978	-4.7836670	8.3661543
C	2.0074421	-1.6454249	4.8389947
H	1.5960858	-0.7202930	4.4211881
H	1.3704214	-2.4563859	4.4757668
C	3.3934608	-1.8511211	4.2399274
O	3.3006792	-2.1867270	2.9400657
O	4.4547343	-1.7245878	4.8244110
C	4.5493201	-2.4041435	2.2442905
H	4.2723169	-2.7007635	1.2308729
H	5.1415458	-1.4843542	2.2264901
H	5.1233020	-3.1972562	2.7328171

*3.4.6.1.3. **int1-C3** diastereomer 1*
E(B3LYP-D3/6-31+G*(THF)) = -1894.463709
E(M06-2X-D3/6-311+G**) = -1894.134939
Gtot(B3LYP-D3/6-31+G*(THF)) = -1894.003540

C	-0.7866822	0.5609935	9.8386884
C	-0.4571085	-0.2788323	7.8845708
C	-1.2891659	0.8337279	7.6740432
O	-1.4973652	1.3682491	8.9808438
O	-1.7768459	1.4041998	6.6751003
C	-0.7950884	1.3264151	14.0454630
C	-1.5134338	2.1577544	13.1802396
C	-1.5194079	1.9204133	11.8051076
C	-0.8006310	0.8358065	11.2625114
C	-0.0797264	0.0008346	12.1447206
C	-0.0787517	0.2452562	13.5139889
H	-0.7935271	1.5142508	15.1167006
H	-2.0761542	2.9994947	13.5792922
H	-2.0820953	2.5708593	11.1414316
H	0.4719713	-0.8388974	11.7319864
H	0.4824622	-0.4127272	14.1751222
N	-0.1621381	-0.4118747	9.2222661

C	0.0201658	-1.2106753	6.8023446
H	-0.2994770	-0.7607360	5.8587960
C	1.6162308	-1.3219488	6.7289974
C	1.5849444	1.2032665	6.3527464
C	2.2573101	0.0974017	6.7178141
P	4.0582237	0.4465881	6.9113423
C	5.1597939	-0.8319200	7.6024231
H	6.1790623	-0.4349944	7.5198988
H	4.9445960	-1.0155166	8.6594859
H	5.0831040	-1.7502916	7.0175905
C	4.7396777	0.8716014	5.2749344
H	5.7940364	1.1508506	5.3791785
H	4.6509557	-0.0064884	4.6290972
H	4.1788715	1.7060754	4.8423312
C	4.2579113	1.8889269	8.0134764
H	3.8325707	1.6571116	8.9956371
H	5.3286847	2.0953657	8.1228311
H	3.7653152	2.7765861	7.6067222
H	2.0561365	2.1825505	6.2874564
H	0.5327125	1.2059399	6.0894065
C	-2.0711781	-5.0398800	7.0364046
C	-1.8928146	-4.3987554	5.8080406
C	-1.2146243	-3.1797921	5.7393691
C	-0.6959267	-2.5706206	6.8904582
C	-0.9168023	-3.2064354	8.1232693
C	-1.5855268	-4.4300823	8.1956788
H	-2.5999222	-5.9891728	7.0926054
H	-2.2887863	-4.8453400	4.8978884
H	-1.0880921	-2.6938592	4.7774686
H	-0.5791952	-2.7295799	9.0361321
H	-1.7394237	-4.8984908	9.1661175
C	2.1693315	-1.9095469	5.4062192
O	1.2595236	-2.0626785	4.4515066
O	3.3666892	-2.1049256	5.2386657
C	2.1294745	-2.1951483	7.9043707
H	1.6289847	-1.8793893	8.8219781
H	3.1978184	-2.0645707	8.0665271
C	1.9616977	-3.6938315	7.6975934
O	1.9565251	-4.3321234	8.8808599
O	1.9089189	-4.2647671	6.6247733
C	1.7176784	-2.6294339	3.2011548
H	0.8393885	-2.6490835	2.5547590
H	2.5049668	-2.0087264	2.7649490
H	2.0951859	-3.6409346	3.3730832
C	1.7920729	-5.7673912	8.8193498
H	1.8248907	-6.1118535	9.8543257
H	0.8274947	-6.0060334	8.3631041
H	2.5970277	-6.2230699	8.2358951

3.4.6.1.4. ***int1-C3*** *diastereomer 2*

E(B3LYP-D3/6-31+G*(THF)) = -1894.468604

E(M06-2X-D3/6-311+G**) = -1894.138255

Gtot(B3LYP-D3/6-31+G*(THF)) = -1894.009250

C	-0.6755415	0.9506852	9.5285562
C	-0.5451971	-0.3022012	7.7837598
C	-1.2622407	0.8400656	7.3710613
O	-1.3431281	1.6452551	8.5494791
O	-1.7569076	1.2482749	6.3040696
C	-0.3389653	2.5892252	13.4612819
C	-0.9896465	3.3090744	12.4541511
C	-1.1068989	2.7845488	11.1669176
C	-0.5710751	1.5179422	10.8590219
C	0.0796266	0.7962201	11.8833052
C	0.1937276	1.3275113	13.1636562
H	-0.2517004	3.0006435	14.4641870
H	-1.4123002	4.2878946	12.6725127
H	-1.6162778	3.3489558	10.3909333
H	0.4813048	-0.1865075	11.6527978
H	0.6963227	0.7533316	13.9398384
N	-0.1942874	-0.1942372	9.1114518
C	-0.1906692	-1.4209895	6.8614802
H	-0.6094719	-1.1199045	5.8978731
C	1.4094629	-1.6103406	6.5623616
C	1.6255598	0.8408549	6.1082434
C	2.1387048	-0.2608764	6.6834263
P	3.6628795	-0.0139987	7.6510528
C	4.8667057	-1.3621050	7.3943866
H	5.7534942	-1.1492745	8.0024097
H	4.4673939	-2.3364480	7.6799957
H	5.1562367	-1.3886968	6.3378848
C	4.5183874	1.4915889	7.0764076
H	5.4803015	1.5396247	7.5999848
H	4.6953109	1.4486962	5.9976395
H	3.9435384	2.3892038	7.3247979
C	3.3192830	0.2277364	9.4344879
H	3.8718539	-0.5078592	10.0257953
H	3.6370900	1.2352075	9.7214190
H	2.2457033	0.1045726	9.6031434
H	2.0991226	1.8165366	6.1493314
H	0.6772701	0.8219023	5.5779265
C	-1.9647039	-5.2775782	7.7902680
C	-1.8826312	-4.8329738	6.4689560
C	-1.3175708	-3.5877637	6.1807538
C	-0.8232317	-2.7679064	7.2059309
C	-0.9269090	-3.2165387	8.5318972
C	-1.4891318	-4.4608682	8.8205311
H	-2.4058133	-6.2460793	8.0172857
H	-2.2643454	-5.4525342	5.6597189
H	-1.2666709	-3.2397699	5.1513446
H	-0.5785487	-2.5722579	9.3321075
H	-1.5654157	-4.7899012	9.8553070
C	1.9448074	-2.6911140	7.5187193
O	2.0185521	-2.2496964	8.7831359
O	2.2589964	-3.8191907	7.1920404
C	2.3735698	-3.2288471	9.7870601
H	2.3089562	-2.7036877	10.7403966
H	1.6654179	-4.0600413	9.7547421
H	3.3872078	-3.6039895	9.6169153
C	1.6346751	-2.2119777	5.1302814
H	1.1516709	-3.1901786	5.1007466
H	2.7059511	-2.3647409	4.9738315
C	1.0731335	-1.4043616	3.9798827
O	2.0026465	-0.6219441	3.4024907
O	-0.0857538	-1.4497699	3.6042106
C	1.5402287	0.2003083	2.3033599
H	2.4060516	0.7866201	1.9916266
H	1.1838868	-0.4281677	1.4820679
H	0.7294279	0.8552964	2.6343860

3.4.6.1.5. ***lactone***
E(B3LYP-D3/6-31+G*(THF)) = -1779.156651
E(M06-2X-D3/6-311+G**) = -1778.813887
Gtot(B3LYP-D3/6-31+G*(THF)) = -1778.732783

C	-1.4978068	1.5806833	5.5303538
C	-0.4222064	-0.0467470	6.4115843
C	0.2236751	0.3296736	5.2930554
O	-0.4075882	1.3612748	4.6967300
O	1.3708184	-0.1568856	4.7552813
C	-4.2849138	4.6384757	4.5352176
C	-3.2299371	4.3550210	3.6626858
C	-2.3062069	3.3584041	3.9757222
C	-2.4366861	2.6351124	5.1730437
C	-3.4990785	2.9218126	6.0495552
C	-4.4157565	3.9192636	5.7286609
H	-5.0035277	5.4160518	4.2864478
H	-3.1271732	4.9099269	2.7332626
H	-1.4905171	3.1366441	3.2934126
H	-3.5932111	2.3590718	6.9738185
H	-5.2346142	4.1367559	6.4105237
N	-1.5319363	0.7679856	6.5549393
C	0.0897480	-1.2191008	7.2049690
H	-0.2345233	-2.0977554	6.6360999
C	1.6625251	-1.2373797	7.0363814
C	1.7274549	0.9187252	8.4064625
C	2.3899740	-0.0485421	7.7471764
P	4.2056961	0.2797758	7.5710089
C	5.2748505	-1.1944265	7.5436228
H	6.3109924	-0.8359983	7.5117718
H	5.1359735	-1.7934407	8.4498080
H	5.0823820	-1.7902598	6.6483488
C	4.5381907	1.2501112	6.0631389
H	5.5991141	1.5279171	6.0614539
H	4.3196795	0.6590929	5.1704400
H	3.9264523	2.1587145	6.0705894
C	4.7585086	1.2874716	8.9871807
H	4.4729678	0.8121056	9.9310224
H	5.8511670	1.3545605	8.9318118
H	4.3482604	2.3012676	8.9418956
H	2.2158549	1.7736277	8.8656289
H	0.6506459	0.9030407	8.5312376
C	-1.9302730	-1.8396793	11.0105418
C	-2.6226922	-1.8062064	9.7966557
C	-1.9348724	-1.5986818	8.6026524
C	-0.5420695	-1.4051815	8.5904661
C	0.1402348	-1.4453482	9.8133755
C	-0.5474594	-1.6635669	11.0119188
H	-2.4649537	-2.0040278	11.9434382
H	-3.7013307	-1.9455053	9.7778154
H	-2.4875783	-1.5812507	7.6664757
H	1.2129154	-1.2984985	9.8600122
H	0.0073535	-1.6904092	11.9471640
O	3.1076708	-1.4800829	5.0847619
C	2.1904961	-2.6316722	7.4663055
H	1.9751801	-2.8160723	8.5197039
H	3.2707554	-2.7090881	7.3451679
C	1.6022455	-3.7712187	6.6371020
O	1.7916572	-4.9440178	7.2455441
O	1.0499528	-3.6399438	5.5589866
C	1.2873600	-6.1192984	6.5533139
H	1.5103566	-6.9590096	7.2123864
H	0.2093627	-6.0260534	6.3959818
H	1.7922671	-6.2361702	5.5907987
C	2.0852930	-1.0299536	5.5413278

3.4.6.1.6. ***TS-cycl-C3*** *diastereomer 1-1*
E(B3LYP-D3/6-31+G*(THF)) = -
1894.4668530461
E(M06-2X-D3/6-311+G**) = -1894.160707
Gtot(B3LYP-D3/6-31+G*(THF)) = -1894.007375

C	1.1758846	-0.6605333	6.9184116
C	-0.8787278	0.1878691	7.0540117
O	0.4415694	0.4790053	6.6466655
O	-1.7773265	1.0057922	6.8968050
N	0.5047443	-1.6131913	7.4771343
C	-0.7983444	-1.1154161	7.6816979
C	-0.8619682	-0.5147846	9.6590351
C	-2.1618404	-0.9684070	9.9747848
C	-2.3920394	-2.3232887	9.3198365
C	-2.0199381	-2.0071426	7.7562195
H	-0.5918177	0.5345205	9.7555547
H	-0.0044819	-1.1573176	9.8457616
H	-2.8485072	-1.3601602	7.4472320
C	-2.1873179	-5.4822414	5.1618626
C	-3.2444329	-4.5715451	5.2137937
C	-3.1629674	-3.4522135	6.0443219
C	-2.0304421	-3.2164859	6.8388116
C	-0.9736853	-4.1395762	6.7757569
C	-1.0526399	-5.2593081	5.9462162
H	-2.2441632	-6.3539150	4.5132131
H	-4.1322180	-4.7287397	4.6045756
H	-3.9934342	-2.7485448	6.0760660
H	-0.0776483	-3.9636955	7.3593969
H	-0.2190530	-5.9581132	5.9092909
P	-3.3830455	0.2387795	10.3598039
C	-2.5141746	1.7300751	10.9726342
H	-3.2613074	2.4091907	11.3964529
H	-1.7880726	1.4634840	11.7454427
H	-2.0049926	2.2440842	10.1515892
C	-4.4360511	-0.2792778	11.7607892
H	-5.1687514	0.5056160	11.9796443
H	-4.9505051	-1.2078692	11.5142621
H	-3.7962119	-0.4419300	12.6332233
C	-4.4759721	0.9262334	9.0520654
H	-4.9818424	1.8199883	9.4353853
H	-3.8390475	1.1939140	8.2032975
H	-5.2109459	0.1846759	8.7413558
C	-3.8362193	-2.8177467	9.2975387
O	-3.9275265	-4.1413392	9.1416094
O	-4.8155786	-2.0871738	9.3065860
C	-1.4748801	-3.4088975	9.9278327
H	-1.6000558	-4.3567868	9.3951701
H	-0.4209149	-3.1432589	9.8332610
C	-1.8091950	-3.6614155	11.3862096
O	-0.7350767	-4.1150246	12.0625725
O	-2.9042478	-3.5114195	11.8977456
C	-5.2554571	-4.6870262	9.0011857
H	-5.1149717	-5.7618515	8.8796927

H	-5.8469847	-4.4747160	9.8961893
H	-5.7497506	-4.2612825	8.1235091
C	-0.9536690	-4.4476098	13.4526219
H	0.0246859	-4.7302905	13.8455284
H	-1.3489949	-3.5841064	13.9950346
H	-1.6572683	-5.2812550	13.5389260
C	5.2878040	-0.7486740	5.7859222
C	4.5092658	0.3730343	5.4886644
C	3.1651982	0.4168433	5.8606128
C	2.5849400	-0.6699429	6.5361135
C	3.3739584	-1.7961673	6.8357610
C	4.7140845	-1.8326984	6.4616895
H	6.3350964	-0.7802550	5.4935660
H	4.9493757	1.2176011	4.9632671
H	2.5611643	1.2880453	5.6255636
H	2.9202501	-2.6326956	7.3593202
H	5.3154762	-2.7082951	6.6960854

*3.4.6.1.7. **int4-C3** diastereomer 1-1*
E(B3LYP-D3/6-31+G*(THF)) = -1894.474468
E(M06-2X-D3/6-311+G**) = -1894.172058
Gtot(B3LYP-D3/6-31+G*(THF)) = -1894.014631

C	-0.1878748	0.3384151	6.1284599
C	0.7039037	-0.7844673	7.8279239
O	0.9981445	-0.2376131	6.5885660
O	1.5817976	-1.2264374	8.5229759
C	-0.0820476	2.2633920	2.3269305
C	1.0843337	1.6936929	2.8437590
C	1.0670218	1.0656406	4.0902409
C	-0.1263855	1.0063374	4.8253817
C	-1.2981928	1.5808908	4.3033957
C	-1.2732873	2.2062614	3.0604554
H	-0.0655458	2.7503261	1.3543452
H	2.0102199	1.7368351	2.2752363
H	1.9727045	0.6207378	4.4911027
H	-2.2153795	1.5268006	4.8824744
H	-2.1825186	2.6484419	2.6597271
N	-1.2035851	0.2176554	6.8898613
C	-0.8074765	-0.6067726	8.0397181
C	-1.1337667	0.0634350	9.4099693
C	-2.3450675	-0.6778970	9.9514831
C	-2.3353275	-2.1085050	9.4251850
C	-1.7037000	-1.9029654	7.9974261
H	-1.2957024	1.1384566	9.2543967
H	-0.2353550	-0.0421438	10.0385050
H	-2.5543653	-1.5266648	7.4302408
C	-0.4201164	-5.3375108	5.6643652
C	-1.5862083	-4.6362953	5.3505552
C	-1.9695494	-3.5376719	6.1222844
C	-1.2058669	-3.1121465	7.2234602
C	-0.0392268	-3.8320502	7.5296005
C	0.3486669	-4.9285956	6.7567548
H	-0.1132246	-6.1914530	5.0644701
H	-2.1973655	-4.9405367	4.5031606
H	-2.8801290	-2.9979006	5.8742091
H	0.5728311	-3.5265718	8.3669398
H	1.2609528	-5.4646087	7.0105709
P	-3.1990013	-0.0079158	11.2634655
C	-2.1041058	0.8792351	12.4528917
H	-2.6963208	1.3962805	13.2164612
H	-1.4322633	0.1612450	12.9344103
H	-1.4996478	1.6186491	11.9160896
C	-4.1302363	-1.2241665	12.2574048
H	-4.4976948	-0.7335490	13.1646870
H	-4.9780953	-1.5907086	11.6750720
H	-3.4769148	-2.0590412	12.5243600
C	-4.4346188	1.2935178	10.8293636
H	-4.8792578	1.7419536	11.7262923
H	-3.9423194	2.0779216	10.2435076
H	-5.2131885	0.8289614	10.2185126
C	-3.7033457	-2.8436393	9.2841009
H	-3.5621207	-3.7127672	8.6308445
H	-4.0179875	-3.2144110	10.2585238
C	-1.4870116	-2.9217188	10.4146475
O	-0.1857579	-2.5889759	10.3343150
O	-1.9156930	-3.6960078	11.2641052
C	-4.8380203	-2.0009995	8.7397274
O	-4.8005441	-1.8847267	7.3993321
O	-5.7268713	-1.5031310	9.4128793
C	0.7008325	-3.1468321	11.3181262
H	1.6884822	-2.7629989	11.0603866
H	0.4112937	-2.8238535	12.3232300
H	0.6877673	-4.2401801	11.2745280
C	-5.8389942	-1.0808144	6.7953312
H	-5.6478850	-1.1167681	5.7219197
H	-6.8244320	-1.4968475	7.0238676
H	-5.7835342	-0.0523295	7.1624004

*3.4.6.1.8. **int4-C3** diastereomer 1-2*
E(B3LYP-D3/6-31+G*(THF)) = -1894.475084
E(M06-2X-D3/6-311+G**) = -1894.177402
Gtot(B3LYP-D3/6-31+G*(THF)) = -1894.012354

C	-0.0284020	0.2260437	6.4533518
C	0.5991125	-1.3552052	7.8773827
O	1.0169888	-0.6537676	6.7650472
O	1.3291754	-2.1282848	8.4492202
N	-1.0478119	0.1756275	7.2205779
C	-0.8159968	-0.8910155	8.1930962
C	-0.9451086	-0.4310658	9.6968499
C	-2.2358043	-1.0285408	10.2232645
C	-2.3933425	-2.3597887	9.4957509
C	-1.9467199	-1.9675564	8.0207854
H	-0.8736389	0.6592243	9.7458311
H	-0.0574672	-0.8231030	10.2169959
H	-2.7811389	-1.3481110	7.6787704
C	-1.5152050	-4.9769271	4.9263424
C	-2.5003398	-3.9909115	4.8483825
C	-2.6107032	-3.0347692	5.8587430
C	-1.7482138	-3.0385064	6.9667877
C	-0.7608859	-4.0359999	7.0302086
C	-0.6471282	-4.9929971	6.0207277
H	-1.4223332	-5.7241935	4.1410786
H	-3.1817706	-3.9618382	4.0004586
H	-3.3790464	-2.2671598	5.7865528
H	-0.0647953	-4.0649540	7.8600242
H	0.1277650	-5.7535733	6.0901837
P	-3.4525574	-0.0105537	10.8489789
C	-2.6367045	1.3722558	11.7412838

H	-3.3946354	1.9408992	12.2909143
H	-1.8919332	0.9831490	12.4409715
H	-2.1452543	2.0494581	11.0352331
C	-4.5242986	-0.8142151	12.0986497
H	-5.1765642	-0.0619592	12.5561347
H	-5.1322827	-1.5841972	11.6221452
H	-3.8957481	-1.2837842	12.8587370
C	-4.6546194	0.9161488	9.7751225
H	-5.2897277	1.5857728	10.3684765
H	-4.0848555	1.5078790	9.0499957
H	-5.2749970	0.1967138	9.2377814
C	-3.8133493	-2.9058772	9.3311830
O	-3.8246982	-4.2190680	9.0591326
O	-4.8288673	-2.2280520	9.3266709
C	-1.4725505	-3.4185165	10.1572586
H	-1.5427988	-4.3882526	9.6549648
H	-0.4314523	-3.0944627	10.1067403
C	-1.8762183	-3.6131994	11.6032168
O	-0.8067215	-3.6388976	12.4225647
O	-3.0199813	-3.7627432	11.9990196
C	-5.1128441	-4.8027490	8.7798665
H	-4.9155252	-5.8519868	8.5548783
H	-5.7663637	-4.7135629	9.6526521
H	-5.5790495	-4.3070419	7.9235965
C	-1.0870792	-3.8457024	13.8234401
H	-0.1173068	-3.8274848	14.3237493
H	-1.7294532	-3.0468771	14.2052683
H	-1.5808081	-4.8096510	13.9792332
C	0.4582475	2.7135935	3.0281165
C	1.4850479	1.8404611	3.3966441
C	1.3446992	1.0253325	4.5208277
C	0.1670071	1.0825052	5.2818831
C	-0.8650745	1.9612256	4.9083294
C	-0.7167148	2.7724901	3.7875237
H	0.5704190	3.3462415	2.1502870
H	2.3972860	1.7921176	2.8067891
H	2.1414814	0.3449423	4.8055898
H	-1.7742680	1.9915663	5.5014962
H	-1.5182305	3.4496486	3.5015135

3.4.6.1.9. ***int4-C3*** *diastereomer 2-1*

E(B3LYP-D3/6-31+G*(THF)) = -1894.478676
E(M06-2X-D3/6-311+G**) = -1894.180858
Gtot(B3LYP-D3/6-31+G*(THF)) = -1894.01548

C	1.0851239	-0.6910967	7.0151646
C	-0.9058444	0.2331487	7.3396882
O	0.2545116	0.3543683	6.5951657
O	-1.8288525	1.0001531	7.2053027
N	0.6162096	-1.4452680	7.9328410
C	-0.7204993	-0.9636126	8.2577314
C	-0.8463845	-0.5490913	9.7822003
C	-2.1843818	-1.0638764	10.2600142
C	-2.4006445	-2.3687578	9.5061740
C	-1.8872635	-1.9911741	8.0382362
H	-0.6847208	0.5259251	9.9065406
H	0.0082694	-1.0402698	10.2709211
H	-2.7083410	-1.3753161	7.6559172
C	-1.3915056	-5.1923367	5.1583210
C	-2.4607184	-4.2993611	5.0665940
C	-2.5897640	-3.2676957	5.9982558
C	-1.6622667	-3.1055168	7.0390103
C	-0.5906228	-4.0105103	7.1175729
C	-0.4582274	-5.0417800	6.1871835
H	-1.2838940	-5.9962535	4.4330773
H	-3.1931003	-4.4021274	4.2684922
H	-3.4263202	-2.5751943	5.9166940
H	0.1526632	-3.8974325	7.8977898
H	0.3820328	-5.7287925	6.2647460
P	-3.3667877	0.0377927	10.8159873
C	-2.5066824	1.3905001	11.7127624
H	-3.2501027	1.9933261	12.2453336
H	-1.7917894	0.9747454	12.4284157
H	-1.9768457	2.0430555	11.0114414
C	-4.5153024	-0.6802359	12.0486626
H	-5.2018283	0.0951361	12.4073486
H	-5.0840655	-1.4912369	11.5926255
H	-3.9336295	-1.0827059	12.8819757
C	-4.4665189	1.0032974	9.6726949
H	-5.0175196	1.7832019	10.2121077
H	-3.8300656	1.4602577	8.9080282
H	-5.1659464	0.3216162	9.1857713
C	-3.8455562	-2.8273103	9.2994380
O	-3.9291078	-4.1327633	9.0077988
O	-4.8185900	-2.0897559	9.2796373
C	-1.5657563	-3.4936117	10.1696302
H	-1.6488357	-4.4371509	9.6203822
H	-0.5086866	-3.2210039	10.1929637
C	-2.0554957	-3.7281773	11.5847307
O	-1.0334133	-3.9086318	12.4456833
O	-3.2245382	-3.7804839	11.9255992
C	-5.2431204	-4.6374392	8.6982828
H	-5.1040053	-5.6968304	8.4776824
H	-5.9103891	-4.5082704	9.5554916
H	-5.6580530	-4.1154099	7.8312560
C	-1.4028749	-4.1642877	13.8183705
H	-0.4632372	-4.2462463	14.3677063
H	-2.0039604	-3.3393593	14.2112745
H	-1.9746035	-5.0940948	13.8956205
C	4.8236292	-1.1037093	5.0138416
C	3.9561298	-0.0707354	4.6491133
C	2.7339546	0.0831832	5.3044504
C	2.3753615	-0.8039163	6.3316130
C	3.2503050	-1.8412187	6.6974084
C	4.4683490	-1.9872688	6.0401799
H	5.7745184	-1.2227305	4.4990028
H	4.2296403	0.6154712	3.8511034
H	2.0577837	0.8828152	5.0177408
H	2.9606979	-2.5235279	7.4912265
H	5.1421984	-2.7921028	6.3241950

3.4.6.1.10. ***int4-C3*** *diastereomer 2-2*

E(B3LYP-D3/6-31+G*(THF)) = -1894.477663
E(M06-2X-D3/6-311+G**) = -1894.175517
Gtot(B3LYP-D3/6-31+G*(THF)) = -1894.015848

C	1.0475112	-0.3727073	6.8044844
C	-1.0926511	0.2067485	6.8267925
O	0.0807681	0.3668788	6.1120412
O	-2.1221250	0.7258790	6.4771686

C	4.9604371	-0.4666779	5.1200637
C	3.9622270	0.2925309	4.5038637
C	2.6782496	0.3378488	5.0483138
C	2.3899481	-0.3817980	6.2180228
C	3.3956301	-1.1444283	6.8358864
C	4.6741901	-1.1846170	6.2875993
H	5.9597550	-0.5016373	4.6917188
H	4.1825123	0.8495354	3.5963468
H	1.9017700	0.9250902	4.5676706
H	3.1585944	-1.7001346	7.7381105
H	5.4494953	-1.7774614	6.7672805
N	0.6548111	-0.9709180	7.8602789
C	-0.7724677	-0.7017234	8.0152051
C	-1.0877282	0.0044551	9.3767539
C	-2.3163198	-0.6919808	9.9277310
C	-2.3731383	-2.1164839	9.3976290
C	-1.7305135	-1.9458095	7.9660816
H	-1.2174124	1.0869441	9.2307459
H	-0.1801125	-0.1268650	9.9862827
H	-2.5750987	-1.5761895	7.3848060
C	-0.5176858	-5.5619175	5.8940177
C	-1.7371770	-4.9415615	5.6171392
C	-2.0939608	-3.7717880	6.2918250
C	-1.2502047	-3.2001676	7.2596249
C	-0.0269193	-3.8367997	7.5278158
C	0.3339716	-5.0023026	6.8498649
H	-0.2319749	-6.4710445	5.3685954
H	-2.4105040	-5.3636884	4.8737297
H	-3.0456968	-3.2933034	6.0717235
H	0.6447964	-3.4049551	8.2564679
H	1.2895855	-5.4747646	7.0689580
P	-3.1153506	-0.0048080	11.2640359
C	-1.9619756	0.7513560	12.4902840
H	-2.5141354	1.2872693	13.2707805
H	-1.3533024	-0.0360945	12.9471138
H	-1.2954749	1.4574416	11.9825653
C	-4.1415368	-1.1795733	12.2129421
H	-4.4703174	-0.6954171	13.1383873
H	-5.0148874	-1.4555041	11.6182435
H	-3.5572134	-2.0727525	12.4506195
C	-4.2416325	1.4011233	10.8684126
H	-4.6645710	1.8441503	11.7780861
H	-3.6829251	2.1701813	10.3230722
H	-5.0416914	1.0202813	10.2282299
C	-3.7807558	-2.7765324	9.2598763
H	-3.6869330	-3.6663110	8.6267470
H	-4.1279730	-3.1080186	10.2369223
C	-1.5620833	-2.9929787	10.3643604
O	-0.2470712	-2.6991912	10.3325443
O	-2.0316846	-3.7943553	11.1646156
C	-4.8459088	-1.8673017	8.6839446
O	-4.7811395	-1.7919378	7.3412702
O	-5.6960811	-1.2792258	9.3331743
C	0.5844943	-3.3796039	11.2890017
H	1.5962594	-3.0135406	11.1079797
H	0.2691935	-3.1443512	12.3103539
H	0.5361044	-4.4627594	11.1402940
C	-5.6985580	-0.8803218	6.6970997
H	-5.5074573	-0.9816029	5.6279017
H	-6.7322190	-1.1498192	6.9308906
H	-5.4997120	0.1429060	7.0264623

3.4.6.2. Cyclization from **int1**

3.4.6.2.1. cyclized product via C

E(B3LYP-D3/6-31+G*(THF)) = -1894.469217

E(M06-2X-D3/6-311+G**) = -1894.1675252908

Gtot(B3LYP-D3/6-31+G*(THF)) = -1894.010377

C	-1.4900262	0.5701716	9.7991663
C	0.2031100	-0.8502858	9.6263379
O	-0.9847242	-0.6315226	10.2973523
O	0.9038872	-1.7994369	9.8645945
C	-5.1359108	2.0046190	11.4592019
C	-4.6366335	0.7644883	11.8653518
C	-3.4410216	0.2808893	11.3329670
C	-2.7425572	1.0443425	10.3861908
C	-3.2456783	2.2912800	9.9783999
C	-4.4377434	2.7674618	10.5150800
H	-6.0670130	2.3793666	11.8785385
H	-5.1782848	0.1723567	12.5989513
H	-3.0507495	-0.6823925	11.6472379
H	-2.6852811	2.8703457	9.2500868
H	-4.8243149	3.7344609	10.2018804
N	-0.8069553	1.1317616	8.8783619
C	0.2467297	-0.3345202	7.1504483
H	0.0800734	0.5300882	6.5028645
C	1.6870668	-0.8115099	6.9116689
H	1.8487917	-1.7918901	7.3862997
H	1.8523785	-0.9572747	5.8338569
C	2.5165966	0.3009364	7.5370183
P	3.9565527	0.7418967	6.7786540
C	3.8503403	1.3670153	5.0344137
H	4.8423931	1.5962964	4.6277054
H	3.3777620	0.6043947	4.4049065
H	3.2173247	2.2579557	5.0266830
C	4.9101443	2.0444901	7.6416114
H	5.9350323	2.0429552	7.2558323
H	4.4748243	3.0305953	7.4530483
H	4.9259747	1.8430454	8.7147115
C	5.1154500	-0.6786290	6.5786324
H	5.9913241	-0.3973317	5.9822205
H	5.4306827	-1.0283698	7.5665767
H	4.5898221	-1.4956544	6.0712494
C	2.2725986	0.8643432	10.0812810
O	3.5128103	0.3723041	10.1716078
O	1.6168330	1.1859485	11.0622917
C	4.0157932	0.1789745	11.5135193
H	5.0072459	-0.2600828	11.3938778
H	4.0785369	1.1341142	12.0435434
H	3.3589047	-0.4985724	12.0656881
C	1.5396363	2.5677844	8.4974775
H	1.0110795	2.9371205	9.3767044
H	2.5301343	3.0386434	8.4992811
C	0.8181470	3.0867579	7.2677971
O	-0.0223708	4.0890930	7.6060065
O	0.9784214	2.7320372	6.1137728
C	-0.7386524	4.7024749	6.5136685
H	-1.3462421	5.4896255	6.9635089
H	-0.0417006	5.1270643	5.7846577
H	-1.3752916	3.9659321	6.0150962

C	-3.1437376	-2.9846896	6.6351762
C	-3.2780303	-1.6030448	6.4763751
C	-2.1688357	-0.7726055	6.6352117
C	-0.9067716	-1.2976039	6.9596515
C	-0.7872762	-2.6857326	7.1213721
C	-1.8952059	-3.5211273	6.9565641
H	-4.0047603	-3.6373910	6.5078795
H	-4.2453335	-1.1721827	6.2262845
H	-2.2759835	0.3028448	6.5139629
H	0.1727849	-3.1216287	7.3809540
H	-1.7805741	-4.5957861	7.0816626
C	0.3296770	0.2761698	8.5938384
C	1.7419816	1.0127522	8.6223787

3.4.6.2.2. cyclized product via O

E(B3LYP-D3/6-31+G*(THF)) = -1894.456555

E(M06-2X-D3/6-311+G**) = -1894.147899

Gtot(B3LYP-D3/6-31+G*(THF)) = -1893.996371

C	-1.4623559	2.4209274	8.5207207
C	-0.3690417	0.7231455	7.7985844
C	0.5221994	1.7208999	8.0300201
O	-0.1314053	2.8223330	8.4858733
O	1.8489491	1.7667450	7.8641987
C	-4.4212523	5.0767520	10.0571769
C	-3.0947648	5.5049096	9.9534476
C	-2.1144664	4.6514502	9.4462228
C	-2.4566195	3.3517886	9.0371215
C	-3.7938137	2.9251275	9.1429262
C	-4.7658636	3.7833282	9.6484266
H	-5.1821865	5.7446565	10.4544967
H	-2.8210148	6.5088286	10.2697060
H	-1.0848921	4.9886027	9.3686644
H	-4.0532409	1.9180158	8.8303064
H	-5.7955283	3.4415587	9.7275651
N	-1.6361286	1.1944238	8.1105276
C	0.0574437	-0.6306555	7.3224385
H	-0.1535392	-0.7431805	6.2543333
C	1.6022970	-0.6684451	7.5194537
H	1.7904381	-0.7347342	8.5965549
H	2.0139048	-1.5549387	7.0399970
C	2.1608910	0.9502036	5.5639285
P	4.1259519	0.5397428	7.5953517
C	4.9015205	2.1859878	7.5403191
H	5.9110352	2.1183447	7.9610570
H	4.9654885	2.5145715	6.5017889
H	4.3055987	2.8888839	8.1292769
C	4.3316845	-0.0782281	9.3076245
H	5.3865398	-0.0045280	9.5971941
H	3.7288177	0.5329486	9.9870036
H	4.0161874	-1.1234344	9.3799551
C	5.0088652	-0.5684389	6.4607379
H	6.0391176	-0.7101610	6.8037234
H	4.5000886	-1.5365622	6.4170732
H	4.9960396	-0.1028023	5.4719745
C	1.8953634	0.0655832	4.4916085
O	1.8096883	-1.2859703	4.8110756
O	1.7617112	0.4181467	3.3010755
C	1.4784861	-2.1550318	3.7267202
H	1.4523897	-3.1619616	4.1521156
H	0.5005264	-1.9075941	3.2984397
H	2.2290390	-2.1050274	2.9305234
C	2.3947858	2.3755658	5.1062750
H	1.5061883	2.7937623	4.6184211
H	2.6134476	3.0317200	5.9574487
C	3.5536343	2.5195683	4.1281840
O	3.2755044	3.3843191	3.1349239
O	4.6433061	1.9714929	4.2302584
C	4.3224662	3.5926600	2.1633636
H	3.9169965	4.3014877	1.4394235
H	5.2156011	4.0040541	2.6430555
H	4.5790950	2.6491341	1.6733264
C	-1.6667439	-3.9845328	9.4625304
C	-1.4525277	-4.0598180	8.0841222
C	-0.9159656	-2.9672427	7.3972515
C	-0.5854874	-1.7869633	8.0740565
C	-0.8044735	-1.7223812	9.4577100
C	-1.3407471	-2.8102365	10.1476482
H	-2.0890086	-4.8317747	9.9989141
H	-1.7076805	-4.9678902	7.5416249
H	-0.7553040	-3.0287373	6.3224550
H	-0.5646788	-0.8078104	9.9950818
H	-1.5102945	-2.7397167	11.2202645
C	2.3002517	0.6073685	6.9842633

3.4.6.2.3. cyclized TS via C

E(B3LYP-D3/6-31+G*(THF)) = -1894.463867

E(M06-2X-D3/6-311+G**) = -1894.1542899274

Gtot(B3LYP-D3/6-31+G*(THF)) = -1894.005407

C	1.6321183	0.0318050	11.8979362
C	3.3225281	0.0012500	10.4555786
O	2.9308018	0.4749577	11.7214588
O	4.4578519	0.1580404	10.0477703
C	-0.2146623	0.6722047	15.6818849
C	1.0991935	1.0990495	15.4696854
C	1.7100915	0.9055759	14.2311581
C	1.0051311	0.2792315	13.1888784
C	-0.3176804	-0.1488356	13.4066865
C	-0.9198744	0.0481960	14.6454262
H	-0.6874853	0.8223965	16.6498436
H	1.6517846	1.5816670	16.2725798
H	2.7331289	1.2309386	14.0681305
H	-0.8537189	-0.6376918	12.5982633
H	-1.9416303	-0.2877877	14.8070416
N	1.1365945	-0.6080787	10.8866145
C	2.1675746	-1.7501696	8.8965183
H	1.1287852	-2.0849257	8.8177003
C	2.6275604	-1.2654142	7.4674008
H	3.6326766	-1.6381977	7.2467906
H	1.9688423	-1.7581293	6.7364456
C	2.5777581	0.2566565	7.4179233
P	3.8572135	1.1073398	6.6042653
C	4.2679550	0.1628871	5.0871483
H	5.0845115	0.6666524	4.5585527
H	4.5893824	-0.8539804	5.3340954
H	3.3883091	0.1066533	4.4382638
C	3.3334417	2.7519123	6.0036252
H	3.9849400	3.0549029	5.1772847
H	2.2967112	2.6855969	5.6675850

H	3.4002404	3.4873184	6.8052381
C	5.4941008	1.3592820	7.4081700
H	6.1697478	1.8903500	6.7274225
H	5.3453157	1.9238945	8.3289610
H	5.9224140	0.3870363	7.6679178
C	1.7710635	2.1037140	9.0650322
O	3.0211869	2.6125685	9.0507286
O	0.8540549	2.6338797	9.6746685
C	3.2303892	3.7898202	9.8590561
H	4.2830777	4.0482051	9.7330612
H	2.5863119	4.6078454	9.5224922
H	3.0145419	3.5624546	10.9071439
C	0.1736869	0.4207188	8.1981216
H	0.0540773	-0.5810001	7.7753392
H	-0.3582528	0.4278065	9.1528905
C	-0.5334252	1.3763941	7.2488121
O	-1.8509821	1.0937213	7.2101626
O	-0.0256023	2.2692113	6.5956747
C	-2.6551700	1.9161582	6.3356543
H	-3.6768865	1.5490110	6.4452602
H	-2.5921958	2.9665456	6.6345943
H	-2.3203399	1.8120680	5.2991281
C	4.3424168	-5.1307073	10.5455282
C	2.9461474	-5.1164217	10.5324399
C	2.2617737	-4.0269279	9.9879469
C	2.9536616	-2.9352981	9.4439843
C	4.3573639	-2.9589801	9.4685199
C	5.0442282	-4.0453251	10.0126254
H	4.8794006	-5.9772809	10.9682008
H	2.3869224	-5.9530212	10.9468900
H	1.1733276	-4.0199951	9.9902872
H	4.9131540	-2.1106471	9.0816732
H	6.1324200	-4.0428282	10.0240685
C	2.1116350	-0.5841485	9.8754844
C	1.6497369	0.7799829	8.3701426

3.4.6.3. Double bond migration by intramolecular double deprotonation via C

3.4.6.3.1. TS of deprotonation by ylide toward Z

E(B3LYP-D3/6-31+G*(THF)) = -2967.365476
E(M06-2X-D3/6-311+G**) = -2966.871601
Gtot(B3LYP-D3/6-31+G*(THF)) = -2966.612761

C	3.1005081	4.4009921	9.6125883
C	2.5643593	2.3700908	9.1674846
C	1.3919281	2.9461311	9.6919582
O	1.7760256	4.2994023	9.9718815
O	0.2303483	2.5689233	9.9389148
C	5.2136186	8.0869945	10.1197381
C	3.8567805	8.0016301	10.4506146
C	3.1549788	6.8069413	10.2890404
C	3.7992823	5.6562223	9.7868464
C	5.1728573	5.7522712	9.4630381
C	5.8637252	6.9480874	9.6245241
H	5.7566681	9.0206006	10.2488149
H	3.3382058	8.8761783	10.8396215
H	2.1016953	6.7514414	10.5487895
H	5.6830202	4.8656671	9.0984870
H	6.9209293	6.9937175	9.3677952
N	3.5897184	3.2799101	9.1405697
C	2.7530619	0.9433853	8.7368643
H	1.7567511	0.4841292	8.7622555
C	3.3310835	0.8509373	7.2837946
H	4.0201589	1.6844636	7.1392649
H	3.9505473	-0.0513065	7.2044367
C	2.0918717	1.9223535	5.3207191
C	2.3029521	0.8530338	6.1585161
P	1.4774273	-0.7149188	5.8531558
C	1.9919514	-1.9807786	7.0617624
H	1.5056059	-2.9236272	6.7874416
H	3.0754304	-2.1277526	7.0299637
H	1.6958961	-1.6997551	8.0750457
C	-0.3398643	-0.6088649	6.0277843
H	-0.7686352	-1.6113081	5.9205785
H	-0.5635162	-0.2253774	7.0291048
H	-0.7875704	0.0603322	5.2896272
C	1.8973421	-1.4203583	4.2185656
H	1.2608167	-2.2884650	4.0143227
H	1.7795622	-0.6764966	3.4281513
H	2.9465830	-1.7359728	4.2416687
C	2.6748167	3.2490471	5.7808727
O	3.9592536	3.4130293	5.4183666
O	2.0315666	4.0447981	6.4251800
C	4.5729641	4.6360303	5.8967313
H	5.6186016	4.5758183	5.5913395
H	4.0858651	5.5013073	5.4387163
H	4.4813839	4.6911592	6.9823698
C	1.2557080	1.9737748	4.1267246
H	2.2099892	1.6283876	3.0515196
H	0.4984579	1.1989829	4.0365703
C	0.7574200	3.2229308	3.5377853
O	1.6919510	4.2101511	3.5325393
O	-0.3355137	3.3573656	2.9933282
C	1.3061966	5.4458133	2.9116120
H	2.1581464	6.1163326	3.0385008
H	1.0992012	5.2841275	1.8492134
H	0.4194824	5.8656934	3.3957186
C	5.2708144	-1.1182928	11.6271311
C	3.8831278	-1.2801634	11.6543660
C	3.0797705	-0.6281152	10.7139606
C	3.6416962	0.1921914	9.7269734
C	5.0361209	0.3572990	9.7180301
C	5.8424651	-0.2916222	10.6543371
H	5.8989444	-1.6231587	12.3581086
H	3.4234878	-1.9134654	12.4109692
H	1.9979125	-0.7486105	10.7540318
H	5.4916390	1.0167209	8.9845705
H	6.9212136	-0.1463973	10.6282732
C	-0.0127902	0.9498775	-0.7399349
O	0.9084155	0.5174511	-1.6237898
O	-1.2122710	0.8508174	-0.9317774
C	0.3829140	-0.0796651	-2.8294029
H	1.2541607	-0.3697485	-3.4188730
H	-0.2259242	-0.9563290	-2.5893560
H	-0.2298871	0.6418335	-3.3781916
C	0.6063843	1.5191115	0.5260966
H	0.9042469	0.6621885	1.1420765
H	-0.1964971	2.0256567	1.0720384
C	1.8191020	2.4011805	0.3209822

C	2.8753810	2.2416516	1.1872015
O	3.9543946	3.0713527	1.0425893
O	2.9429186	1.3622059	2.1573227
C	4.9867129	2.9902049	2.0452635
H	5.6904066	3.7876540	1.7960495
H	4.5687787	3.1469758	3.0418902
H	5.4938778	2.0215851	2.0093305
C	0.4973543	4.0184533	-1.0230673
C	1.6917671	3.4880265	-0.6590516
H	0.3794974	4.7575174	-1.8078091
H	-0.4209986	3.7216873	-0.5240410
P	3.1325654	4.0918734	-1.6201831
C	2.5717694	4.7544560	-3.2282363
H	3.4617192	4.9928101	-3.8218415
H	1.9698621	4.0140289	-3.7627244
H	1.9895880	5.6711413	-3.0931571
C	4.0205474	5.4816271	-0.8416459
H	4.8560983	5.7768504	-1.4872403
H	3.3288107	6.3259785	-0.7475125
H	4.3849309	5.1930582	0.1433496
C	4.2634004	2.7202917	-2.0079231
H	5.0646375	3.0870927	-2.6592442
H	4.6868998	2.3174310	-1.0874765
H	3.6988398	1.9382360	-2.5251515

3.4.6.3.2. TS of deprotonation by ylide toward E

E(B3LYP-D3/6-31+G*(THF)) = -2967.354908

C	1.6234530	2.7647811	9.6104327
C	2.9686394	1.1922537	9.0300630
C	2.2173791	0.6548094	10.0911876
O	1.3163043	1.7132102	10.4514536
O	2.1473886	-0.4454637	10.6838275
C	-0.4706102	6.4790133	9.7978327
C	-0.6933302	5.5068085	10.7780423
C	-0.0237454	4.2827549	10.7287971
C	0.8884162	4.0124437	9.6912862
C	1.1024987	4.9953261	8.6982469
C	0.4310744	6.2115063	8.7571356
H	-0.9919300	7.4333314	9.8411980
H	-1.3901438	5.7044279	11.5911634
H	-0.1938611	3.5328774	11.4972325
H	1.7962288	4.7742823	7.8907141
H	0.6091594	6.9605085	7.9869893
N	2.5850330	2.4887660	8.7739907
C	4.1071474	0.5231866	8.3148238
H	4.0973010	-0.5196324	8.6489681
C	3.9711448	0.5364397	6.7395330
H	4.6587810	1.2854413	6.3458816
H	4.3477881	-0.4256150	6.3622121
C	2.3106778	1.9043801	5.3605419
C	2.5910283	0.8230391	6.1571334
P	1.2762625	-0.3606673	6.5301460
C	1.8340278	-1.7256903	7.5988327
H	1.0667570	-2.5068479	7.5463696
H	2.7759019	-2.1365646	7.2219636
H	1.9489443	-1.4091951	8.6399348
C	-0.1641932	0.4072550	7.3462951
H	-0.9369367	-0.3550810	7.4932500
H	0.1570621	0.7965576	8.3146861
H	-0.5679964	1.2250108	6.7466272
C	0.7132366	-1.2305561	5.0141681
H	-0.1825881	-1.8185459	5.2448844
H	0.4953780	-0.5408737	4.1971116
H	1.5141333	-1.9092054	4.7007555
C	3.4229594	2.9092963	5.1159799
O	3.7458937	2.9120727	3.8053194
O	3.9716397	3.6075319	5.9398785
C	4.7359151	3.8785835	3.4052272
H	4.8523505	3.7400215	2.3317605
H	4.3829058	4.8898044	3.6269041
H	5.6799280	3.7018347	3.9291736
C	1.0842255	2.1655513	4.6049152
H	1.4032739	1.8595856	3.1811356
H	0.2763874	1.4533390	4.7427607
C	0.4900690	3.4988789	4.4746139
O	1.3752637	4.5228486	4.6196336
O	-0.6895174	3.7000769	4.1917506
C	0.8262451	5.8499313	4.5589661
H	1.6745050	6.5236452	4.6944778
H	0.3462236	6.0313324	3.5928293
H	0.0916609	5.9989055	5.3564210
C	7.9719720	2.1713818	9.4395360
C	7.6999773	0.8179602	9.6471182
C	6.4564704	0.2881518	9.2833299
C	5.4672156	1.0987337	8.7131389
C	5.7502252	2.4606033	8.5098406
C	6.9898247	2.9897201	8.8688516
H	8.9378059	2.5866619	9.7216606
H	8.4545834	0.1722832	10.0934586
H	6.2523392	-0.7682933	9.4528176
H	4.9848288	3.0945677	8.0734141
H	7.1904497	4.0474420	8.7060585
C	0.6000772	4.7991430	-0.2546585
O	0.4944834	3.9432489	-1.2934355
O	0.2279015	5.9575298	-0.3111339
C	-0.1071020	4.4852845	-2.4908563
H	-0.1314495	3.6620856	-3.2067301
H	-1.1212677	4.8409356	-2.2832059
H	0.4926575	5.3150841	-2.8782942
C	1.1968543	4.1436273	0.9808905
H	0.4058439	3.5484228	1.4534065
H	1.4253763	4.9571979	1.6801837
C	2.3918505	3.2522388	0.7198226
C	2.4511286	2.0386653	1.3607630
O	3.6053944	1.3108623	1.2192095
O	1.4944396	1.4996373	2.0732551
C	3.7424256	0.1355110	2.0348897
H	4.7282531	-0.2704985	1.7961954
H	3.6933006	0.4035598	3.0937436
H	2.9677769	-0.6007323	1.8037704
C	3.7688864	5.1120397	-0.2085522
C	3.4692247	3.7933284	-0.1188015
H	4.4809200	5.5221802	-0.9186708
H	3.2839789	5.8387804	0.4395769
P	4.3257581	2.7083132	-1.3282246
C	4.7177891	3.6558889	-2.8422166
H	5.1170442	2.9585843	-3.5883287
H	3.8136444	4.1299043	-3.2366061
H	5.4735977	4.4226849	-2.6442917

C	5.9194049	2.0514946	-0.7319398
H	6.3325447	1.3669812	-1.4828418
H	6.6156150	2.8848256	-0.5844162
H	5.7669267	1.5268329	0.2125671
C	3.2183472	1.3599034	-1.8413113
H	3.5995248	0.9096030	-2.7640985
H	3.1628443	0.6072488	-1.0532356
H	2.2210345	1.7786522	-2.0088307

3.4.6.3.3. ***int1-deprot-Z-Z***
E(B3LYP-D3/6-31+G*(THF)) = -1893.994148
E(M06-2X-D3/6-311+G**) = -1893.643088
Gtot(B3LYP-D3/6-31+G*(THF)) = -1893.547168

C	-0.8150837	0.4953797	10.2971300
C	-0.6491823	0.0193707	8.1951472
C	-1.6892404	0.9722920	8.2781084
O	-1.7579651	1.2832026	9.6814908
O	-2.4700623	1.5339666	7.4911070
C	-0.3218792	0.6130090	14.5440491
C	-1.2605002	1.4414127	13.9199512
C	-1.4266508	1.4156295	12.5354511
C	-0.6521187	0.5510169	11.7333713
C	0.2915917	-0.2839895	12.3746787
C	0.4520699	-0.2498062	13.7555204
H	-0.1969744	0.6341935	15.6242365
H	-1.8721939	2.1143598	14.5181249
H	-2.1601874	2.0610526	12.0609406
H	0.8848533	-0.9628718	11.7690748
H	1.1834397	-0.9054668	14.2252829
N	-0.1526826	-0.2556503	9.4483765
C	-0.2095219	-0.7109908	6.9574630
H	-0.6120860	-0.1546980	6.1037640
C	1.3470789	-0.7831542	6.7959193
H	1.7367686	-1.3109315	7.6709211
H	1.5631182	-1.4457221	5.9462890
C	2.0643373	1.1316500	5.2997342
C	2.0735839	0.5363907	6.5757841
P	2.7976587	1.3119993	7.9850395
C	3.0865252	0.0912872	9.3091147
H	3.5645617	0.6073940	10.1484127
H	2.1245383	-0.3184573	9.6311655
H	3.7447453	-0.7080150	8.9532800
C	4.4467779	2.0670208	7.6924873
H	4.9260207	2.2306035	8.6638403
H	5.0604072	1.3917151	7.0902739
H	4.3474774	3.0281323	7.1850969
C	1.8036923	2.6487737	8.7454665
H	2.3406203	3.0887623	9.5939358
H	1.6195775	3.4102971	7.9828767
H	0.8517851	2.2324148	9.0804831
C	2.8182333	2.4252779	5.0959714
O	3.9928089	2.2058022	4.4762327
O	2.4693578	3.5188302	5.5075736
C	4.7204790	3.3852390	4.0815114
H	5.6206953	3.0227189	3.5824353
H	4.1112928	3.9771921	3.3933327
H	4.9865872	3.9904161	4.9531372
C	1.4511444	0.6007856	4.1622570
H	0.9092001	-0.3340240	4.2298153
C	1.4279046	1.2538859	2.8889156
O	0.8317771	0.4488634	1.9350377
O	1.8489019	2.3791506	2.5853326
C	0.7372578	1.0125370	0.6232861
H	0.2764588	0.2426824	-0.0011789
H	0.1176608	1.9161706	0.6213742
H	1.7264550	1.2686467	0.2285737
C	-1.6336341	-4.8188777	6.7228949
C	-1.8315593	-3.9426849	5.6538348
C	-1.3897799	-2.6179483	5.7385670
C	-0.7498208	-2.1412977	6.8900872
C	-0.5607522	-3.0325475	7.9599716
C	-0.9957923	-4.3556180	7.8784880
H	-1.9746990	-5.8502870	6.6591841
H	-2.3314815	-4.2895046	4.7512742
H	-1.5487391	-1.9428573	4.8991843
H	-0.0850513	-2.6689961	8.8665539
H	-0.8398121	-5.0277453	8.7208957

3.4.6.3.4. ***int1-deprot-Z-E***
E(B3LYP-D3/6-31+G*(THF)) = -1893.986952
E(M06-2X-D3/6-311+G**) = -1893.635222
Gtot(B3LYP-D3/6-31+G*(THF)) = -1893.541070

C	-0.4196956	0.6158760	10.3077344
C	-0.4215611	-0.1492174	8.2895942
C	-1.4162256	0.8535269	8.3056426
O	-1.3813848	1.3503118	9.6555005
O	-2.2290537	1.3393857	7.4986578
C	0.5354370	1.4082027	14.3972885
C	-0.4411722	2.1666573	13.7429945
C	-0.7644283	1.9177993	12.4098455
C	-0.1134063	0.8917940	11.6910359
C	0.8674299	0.1268744	12.3654515
C	1.1855842	0.3852392	13.6935644
H	0.7842063	1.6058995	15.4374907
H	-0.9571475	2.9620556	14.2778727
H	-1.5230259	2.5134294	11.9101096
H	1.3627337	-0.6750423	11.8269107
H	1.9437677	-0.2189900	14.1891112
N	0.1579518	-0.2695064	9.5282406
C	-0.0339600	-1.0081585	7.1209245
H	-0.2811206	-0.4536739	6.2093961
C	1.5038218	-1.3096857	7.1013544
H	1.7433828	-1.7643942	8.0622193
H	1.7014659	-2.0723639	6.3448210
C	2.1867999	0.6194282	5.6127045
C	2.3346730	-0.0669943	6.8358663
P	3.3029700	0.5474810	8.1955911
C	3.5515707	-0.8157299	9.3933254
H	4.2456967	-0.4601254	10.1629186
H	2.5956571	-1.0696366	9.8553452
H	3.9788905	-1.6944273	8.9010604
C	5.0361979	0.9554919	7.7494308
H	5.5648839	1.2927456	8.6485287
H	5.5161413	0.0469834	7.3712415
H	5.0736937	1.7366900	6.9913654
C	2.6158802	1.9071526	9.2389817
H	2.4921274	1.5497279	10.2643789
H	3.2790980	2.7744066	9.2115476

H	1.6399309	2.1900610	8.8422623
C	2.4500509	2.0991293	5.6275940
O	2.0435876	2.7577937	4.5280487
O	2.9713161	2.6943471	6.5660128
C	2.1930916	4.1930737	4.5600682
H	1.7508747	4.5521507	3.6293086
H	1.6683747	4.6149026	5.4225548
H	3.2509254	4.4671539	4.6136514
C	1.6815972	0.0915638	4.4066218
H	1.2677462	0.7872240	3.6880659
C	1.7126470	-1.2686597	3.9714619
O	0.8918472	-1.4468564	2.8610756
O	2.3641408	-2.2261367	4.4117297
C	0.8680908	-2.7753901	2.3310177
H	0.1586563	-2.7548495	1.4995373
H	1.8560626	-3.0831035	1.9693748
H	0.5368746	-3.4938101	3.0886405
C	-2.0142272	-4.8667644	6.8578966
C	-1.7526083	-4.1212615	5.7045963
C	-1.1326225	-2.8735275	5.8024072
C	-0.7709003	-2.3422052	7.0490460
C	-1.0365740	-3.0995732	8.1983213
C	-1.6509563	-4.3505438	8.1053557
H	-2.4979198	-5.8388995	6.7855138
H	-2.0350592	-4.5113118	4.7283421
H	-0.9211773	-2.2987463	4.9020156
H	-0.7586454	-2.6968313	9.1689437
H	-1.8489811	-4.9235012	9.0098042

3.4.6.3.5. ***ylide-Z-prot***
E(B3LYP-D3/6-31+G*(THF)) = -1073.376202
E(M06-2X-D3/6-311+G**) = -1073.149981
Gtot(B3LYP-D3/6-31+G*(THF)) = -1073.123310

C	-2.2044163	0.2674782	6.2537430
O	-3.3315596	0.1773637	6.9570352
O	-2.1743491	0.6048368	5.0770588
C	-1.1931111	-0.4405820	8.5100768
H	-2.2197531	-0.7677034	8.6826401
H	-0.5577602	-1.2824641	8.8102972
C	-0.8953208	0.7219780	9.4576014
O	-1.3259895	0.4353896	10.6945526
O	-0.3283817	1.7517712	9.1495924
C	-1.0708338	1.4368697	11.7126245
H	-1.4303502	1.0001282	12.6453924
H	-0.0007598	1.6524158	11.7746305
H	-1.6171616	2.3559633	11.4825286
C	-4.5636349	0.4959919	6.2580789
H	-5.3542231	0.3513407	6.9947902
H	-4.5391275	1.5325249	5.9107770
H	-4.6949299	-0.1785352	5.4073749
H	2.1396037	-1.0312568	6.8310476
H	1.3059131	-0.5703145	8.3145379
C	0.2541800	-0.0007162	6.4983654
P	0.5851275	0.4465230	4.7263255
C	-0.0885087	-0.7559229	3.5281186
H	0.2897297	-0.4840291	2.5354287
H	0.2904435	-1.7494643	3.7936007
H	-1.1771530	-0.7570168	3.5250564
C	2.3911226	0.3313810	4.4628719
H	2.5785519	0.6310021	3.4249620
H	2.9388433	1.0068180	5.1261070
H	2.7490007	-0.6935801	4.6004441
C	0.2063386	2.1908218	4.3408486
H	0.4713458	2.3659033	3.2909982
H	-0.8464337	2.4194719	4.5019838
H	0.8345216	2.8265354	4.9756132
C	-0.9820358	-0.0726140	7.0530498
C	1.5174554	-0.2563782	7.2931336
H	2.1233170	0.6563726	7.3588234

3.4.7. Regioisomer formation
3.4.7.1. **ylide-regio**
E(B3LYP-D3/6-31+G*(THF)) = -1072.863620
E(M06-2X-D3/6-311+G**) = -1072.689242
Gtot(B3LYP-D3/6-31+G*(THF)) = -1072.623095

C	0.5439986	0.0366521	7.2184153
O	0.2057658	0.3527543	8.4882682
O	1.5594657	-0.5786390	6.9384888
C	-1.7510744	-0.4130103	6.4080672
H	-2.2910078	-0.3090583	7.3420311
C	-2.1330586	-1.3852229	5.4878063
O	-3.2832429	-2.0786761	5.9095414
O	-1.6213151	-1.6661323	4.3731009
C	-3.8058381	-3.0191201	4.9750698
H	-4.6897209	-3.4572594	5.4487338
H	-4.0960500	-2.5379368	4.0330127
H	-3.0845896	-3.8116690	4.7435813
C	1.0943202	-0.1291409	9.5194650
H	0.6642229	0.2154576	10.4616616
H	1.1453414	-1.2221134	9.5006271
H	2.1000048	0.2809200	9.3857234
H	-0.8473324	3.9087713	6.9854197
C	-0.4676397	2.8908215	7.0141718
H	0.3035124	2.6939610	7.7532709
C	-0.9222470	1.9132687	6.2145276
P	-2.2817794	2.2818287	5.0516961
C	-2.0365962	1.4138034	3.4740515
H	-2.8836165	1.6454167	2.8178709
H	-1.1137447	1.7811003	3.0108954
H	-1.9629157	0.3299779	3.6243373
C	-2.2432651	4.0688859	4.6664153
H	-2.9715913	4.2645365	3.8714904
H	-2.5142541	4.6680735	5.5420089
H	-1.2454492	4.3558115	4.3204411
C	-3.9603088	1.9645694	5.6962546
H	-4.6940925	2.4081588	5.0121607
H	-4.1132929	0.8879307	5.7853504
H	-4.0582050	2.4292512	6.6833648
C	-0.5269156	0.4386223	6.2079132
H	-0.0998068	0.1873033	5.2304762

3.4.7.2. TS intramolecular migration
E(B3LYP-D3/6-31+G*(THF)) = -1072.794035
E(M06-2X-D3/6-311+G**) = -1072.628809
Gtot(B3LYP-D3/6-31+G*(THF)) = -1072.556494

C	-0.8819647	0.5064281	5.4822428
C	-1.6122289	-0.8569379	5.7044590

H	-1.2526588	0.0666998	6.5957163
H	-2.6393571	-0.9297365	5.3720113
H	2.0840299	-0.4301558	3.8623522
C	1.0310197	-0.3647391	4.1170254
H	0.3677520	-1.0636070	3.6120527
C	0.5252718	0.4958961	5.0203937
P	1.6814415	1.4399687	6.0486973
C	1.5290279	0.7974957	7.7482814
H	2.3953525	1.1041690	8.3443019
H	0.6152792	1.1917156	8.2037746
H	1.4531094	-0.2935870	7.6936681
C	1.3840758	3.2348383	6.0997585
H	2.1072668	3.6845835	6.7902270
H	1.5044947	3.6592531	5.0989580
H	0.3649037	3.4295572	6.4383199
C	3.4048933	1.2022489	5.5029081
H	4.0612397	1.8064760	6.1381329
H	3.6890280	0.1492021	5.5928648
H	3.5188128	1.5256459	4.4627356
C	-1.6651491	1.6952222	5.2441768
O	-2.9746522	1.5668058	5.6411218
O	-1.2484730	2.7505929	4.7442384
C	-3.7908326	2.7370388	5.4811637
H	-4.7889067	2.4506177	5.8230659
H	-3.4118489	3.5690854	6.0858034
H	-3.8295611	3.0530887	4.4333024
C	-0.9153048	-2.0114900	6.1267241
O	-1.7239259	-3.1448637	6.0343071
O	0.2551865	-2.1080439	6.5454759
C	-1.1434926	-4.3506964	6.5379136
H	-1.9045330	-5.1266538	6.4127473
H	-0.2399626	-4.6266187	5.9817839
H	-0.8795965	-4.2596876	7.5982828

3.4.7.3. Addition TS *syn-syn*

E(B3LYP-D3/6-31+G*(THF)) = -1894.450887

E(M06-2X-D3/6-311+G**) = -1894.110173

Gtot(B3LYP-D3/6-31+G*(THF)) = -1893.99402

C	-1.1160409	-1.4518242	9.6499360
C	-0.7682007	-0.4809713	7.7593809
C	-0.6818208	0.5466392	8.7586575
O	-0.9231709	-0.1285969	9.9821188
O	-0.4542735	1.7526957	8.7423753
C	-2.0155916	-4.2879891	12.7099364
C	-1.7765216	-2.9525030	13.0471209
C	-1.4797841	-2.0147580	12.0576315
C	-1.4193801	-2.4053301	10.7076713
C	-1.6648591	-3.7532650	10.3746442
C	-1.9578000	-4.6825103	11.3673298
H	-2.2484148	-5.0155623	13.4843341
H	-1.8232805	-2.6380081	14.0874692
H	-1.2955293	-0.9778651	12.3230009
H	-1.6285423	-4.0502758	9.3305922
H	-2.1477115	-5.7186590	11.0947925
N	-1.0235935	-1.7007090	8.3765547
C	-0.5805523	-0.2439508	6.3829627
H	-0.7143324	0.7954686	6.0993273
C	1.6461962	0.1806692	6.0199728
C	3.9194082	-0.9807867	5.8757681
H	5.7512252	0.0509254	6.2976532
C	-1.3836238	-3.0001699	3.1910291
C	-1.5258998	-1.6224121	3.0057464
C	-1.2545491	-0.7450742	4.0536551
C	-0.8517897	-1.2200588	5.3165895
C	-0.7063425	-2.6117148	5.4877385
C	-0.9684181	-3.4869593	4.4342335
H	-1.5953054	-3.6879917	2.3746678
H	-1.8480830	-1.2309860	2.0430395
H	-1.3690694	0.3258181	3.9040525
H	-0.3901206	-2.9952305	6.4492774
H	-0.8537125	-4.5585224	4.5867277
H	1.6731735	0.8605608	6.8633052
P	4.6626908	-2.1873259	4.7487637
C	3.9503222	-2.0144365	3.0858574
H	4.1802995	-2.9074217	2.4947973
H	2.8714118	-1.8600228	3.1658449
H	4.3778737	-1.1262595	2.6096352
C	4.3250661	-3.8696658	5.3609824
H	4.6395285	-4.5994941	4.6068115
H	4.8818339	-4.0356729	6.2891367
H	3.2572190	-3.9860428	5.5642981
C	6.4660082	-1.9800641	4.6299463
H	6.8543705	-2.7382677	3.9401415
H	6.7077342	-0.9864215	4.2377921
H	6.9307842	-2.1148500	5.6118932
C	2.2599665	-1.6875533	7.5660120
O	1.9834647	-3.0026894	7.5307174
O	2.4254614	-1.0526399	8.5873772
C	1.8350202	-3.6658466	8.8088861
H	1.7075551	-4.7232786	8.5728771
H	2.7247744	-3.5107178	9.4250857
H	0.9541408	-3.2766364	9.3203050
C	2.4002180	-1.1162683	6.1511102
H	1.9680431	-1.8407284	5.4519678
H	4.2315842	0.6755370	7.1399448
C	4.6806155	-0.0446891	6.4592158
C	1.5493473	0.7283844	4.7037663
O	1.0087120	1.9956900	4.7036716
O	1.8125783	0.1485076	3.6409948
C	0.7750050	2.5646340	3.4086782
H	0.3122394	3.5377792	3.5889795
H	1.7129811	2.6904061	2.8565689
H	0.1048421	1.9343146	2.8153230

3.4.7.4. Addition TS *syn-anti*

E(B3LYP-D3/6-31+G*(THF)) = -1894.442972

E(M06-2X-D3/6-311+G**) = -1894.112876

Gtot(B3LYP-D3/6-31+G*(THF)) = -1893.986555

C	-0.8900446	0.1954246	10.5356038
C	-0.7149031	-0.1784328	8.4250852
C	-0.7123625	1.2573884	8.5846609
O	-0.8296729	1.4578580	9.9803871
O	-0.6494770	2.2093114	7.8187364
C	-1.2638258	-0.2035009	14.7659135
C	-1.2334875	1.0692100	14.1886931
C	-1.1105872	1.2148799	12.8063442
C	-1.0151638	0.0798080	11.9830788
C	-1.0455796	-1.2000141	12.5707324

C	-1.1688710	-1.3377072	13.9495666
H	-1.3607073	-0.3131451	15.8438764
H	-1.3078009	1.9534529	14.8177917
H	-1.0902150	2.2042874	12.3592968
H	-0.9702654	-2.0735374	11.9299783
H	-1.1909538	-2.3315160	14.3918718
N	-0.8323118	-0.7757405	9.6780105
C	-0.5663937	-0.8262795	7.2041861
H	-0.6452859	-0.1856469	6.3322823
C	1.8440540	-1.0363855	6.8716431
C	1.8542899	-0.6413222	4.3399724
H	0.2891673	-0.0691922	2.9941855
C	-1.6726602	-4.9347320	6.5923369
C	-2.0424074	-3.9498417	5.6744923
C	-1.6631554	-2.6231024	5.8817109
C	-0.9027398	-2.2495311	7.0047134
C	-0.5392911	-3.2534437	7.9234792
C	-0.9214183	-4.5774001	7.7177536
H	-1.9681656	-5.9701792	6.4363026
H	-2.6334407	-4.2121331	4.7996673
H	-1.9881480	-1.8583108	5.1788794
H	0.0388011	-2.9870372	8.8010747
H	-0.6308194	-5.3371020	8.4405611
H	1.7877118	-1.7911932	7.6434027
P	2.9890189	0.5626559	3.5768850
C	4.7007035	0.4272371	4.1727227
H	5.3066549	1.1657613	3.6354438
H	4.7230633	0.6300674	5.2447572
H	5.0925705	-0.5738286	3.9619547
C	2.3704225	2.2620047	3.7680250
H	3.0498637	2.9592533	3.2652321
H	1.3770187	2.3332155	3.3126102
H	2.3093875	2.4790496	4.8360064
C	3.0501948	0.2179791	1.7824823
H	3.7202121	0.9363440	1.2961923
H	3.4232026	-0.7981104	1.6164465
H	2.0515828	0.3081984	1.3431181
H	3.4309249	-1.5063575	5.5090476
C	2.3280714	-1.5184349	5.5010823
H	-0.0971083	-1.4170700	4.1896633
C	0.6248008	-0.7033444	3.8129336
C	2.3179147	0.2383566	7.2702727
O	2.1182449	0.4904806	8.6041394
O	2.8491459	1.1015865	6.5352036
C	2.4530709	1.8127563	9.0461889
H	2.2298920	1.8284821	10.1149558
H	3.5133234	2.0282296	8.8762821
H	1.8426083	2.5574932	8.5268645
C	2.0019339	-3.0035514	5.2698244
O	2.5402475	-3.7414166	6.2582632
O	1.4039793	-3.4861144	4.3291045
C	2.3185189	-5.1672321	6.1775512
H	2.7414715	-5.5797882	7.0947981
H	1.2486267	-5.3772130	6.1188800
H	2.8274200	-5.5817643	5.3020944

3.4.7.5. Addition TS *anti-syn*
E(B3LYP-D3/6-31+G*(THF)) = -1894.441945
E(M06-2X-D3/6-311+G**) = -1894.113676
Gtot(B3LYP-D3/6-31+G*(THF)) = -1893.984866

C	-0.8288447	0.8349254	9.6322227
C	-0.8960798	-0.1748575	7.7282909
C	-1.1194660	1.2269487	7.4543998
O	-1.0466261	1.8532922	8.7214523
O	-1.3386105	1.8846722	6.4463071
C	-0.5100268	1.8019259	13.7692734
C	-0.7900486	2.8093952	12.8419750
C	-0.8951418	2.5072261	11.4842437
C	-0.7198360	1.1845233	11.0418698
C	-0.4377937	0.1729968	11.9805318
C	-0.3342872	0.4827924	13.3325816
H	-0.4304748	2.0405353	14.8274446
H	-0.9317395	3.8342658	13.1775833
H	-1.1223038	3.2888548	10.7653145
H	-0.3004774	-0.8469596	11.6330557
H	-0.1175544	-0.3052690	14.0502588
N	-0.7393772	-0.3467073	9.1049682
C	-0.8300025	-1.1466859	6.7413288
H	-0.9552968	-0.7639682	5.7327105
C	1.7965417	-1.3899898	6.4827394
H	1.5251457	-2.0681226	7.2782322
C	2.0124217	1.1714615	6.0914873
H	1.0168140	1.9855768	4.3828180
C	-1.3427598	-5.3809314	7.0462155
C	-1.3848827	-4.7366032	5.8069916
C	-1.1957560	-3.3598236	5.7297135
C	-0.9720539	-2.5896424	6.8898811
C	-0.9357578	-3.2537838	8.1348967
C	-1.1154803	-4.6325348	8.2067150
H	-1.4878548	-6.4570944	7.1083057
H	-1.5620368	-5.3099604	4.8998748
H	-1.2195883	-2.8652992	4.7611946
H	-0.7652611	-2.6752445	9.0355152
H	-1.0829100	-5.1266894	9.1756957
C	2.1908111	-1.9918813	5.2633929
O	1.8130655	-3.3268666	5.2212256
O	2.7765892	-1.4724671	4.2917661
C	1.9837054	-3.9813087	3.9662418
H	1.6072529	-4.9982285	4.1057683
H	1.4131353	-3.4841594	3.1722787
H	3.0371478	-4.0112880	3.6648054
P	2.3908622	2.8446080	6.7096848
C	3.9282061	3.5687136	6.0504421
H	3.9916322	4.6106707	6.3846173
H	4.7947839	3.0034991	6.3907945
H	3.8783364	3.5436433	4.9564302
C	2.4291026	2.8576634	8.5292880
H	2.6438433	3.8752889	8.8725646
H	1.4484256	2.5539979	8.9082231
H	3.2049218	2.1820465	8.8947907
C	1.0717429	3.9975217	6.2061135
H	1.2146823	4.9397932	6.7469368
H	1.1392767	4.2022371	5.1326279
H	0.0952584	3.5615253	6.4321613
H	1.8989439	0.1063117	7.9309281
C	2.3541598	-0.0691178	6.9407629
H	1.0494366	0.1486249	4.5248525

C	1.3263295	1.1076574	4.9431405
C	3.8616814	-0.1176077	7.2602909
O	4.2593266	-1.3248813	7.6683218
O	4.6107490	0.8494859	7.2253811
C	5.6463367	-1.4473598	8.0626037
H	5.7606838	-2.4774627	8.4041465
H	6.3014853	-1.2504765	7.2091517
H	5.8782930	-0.7471234	8.8703686

3.4.7.6. Addition TS *anti-anti*
E(B3LYP-D3/6-31+G*(THF)) = -1894.447326
E(M06-2X-D3/6-311+G**) = -1894.118471
Gtot(B3LYP-D3/6-31+G*(THF)) = -1893.989630

C	-1.4304220	1.0366414	9.7043150
C	-0.7104944	0.1986943	7.8549228
C	-1.0231119	1.5694959	7.5756009
O	-1.4769168	2.0901431	8.8114434
O	-1.0167035	2.2829063	6.5671901
C	-2.5441352	1.6705840	13.7677784
C	-2.6448843	2.7197061	12.8495797
C	-2.2865306	2.5266567	11.5152043
C	-1.8208390	1.2726764	11.0852656
C	-1.7195337	0.2190618	12.0147340
C	-2.0794867	0.4194037	13.3432309
H	-2.8252100	1.8242610	14.8069733
H	-3.0064378	3.6926388	13.1736363
H	-2.3687138	3.3406954	10.8014435
H	-1.3508553	-0.7451376	11.6782761
H	-1.9969451	-0.4014503	14.0521571
N	-1.0053790	-0.0763128	9.1861341
C	-0.1255839	-0.6841586	6.9259596
H	0.0726672	-0.2198486	5.9632435
C	2.0148913	-0.6692778	7.5353175
H	1.7921682	-0.1930052	8.4817439
C	2.3152033	1.5745009	6.2852909
H	1.2446937	2.8410168	4.9231559
C	-0.8640543	-4.8847230	6.5312226
C	-0.4505393	-4.1179235	5.4371148
C	-0.1874681	-2.7606101	5.5992195
C	-0.3466377	-2.1315019	6.8513729
C	-0.7495971	-2.9196790	7.9476797
C	-1.0071089	-4.2794708	7.7831505
H	-1.0722940	-5.9451087	6.4072550
H	-0.3319243	-4.5794732	4.4593422
H	0.1352590	-2.1677110	4.7451522
H	-0.8593793	-2.4501494	8.9175615
H	-1.3197866	-4.8722754	8.6405083
C	2.2912044	-2.0755043	7.6862412
O	2.7526202	-2.6602133	6.5307699
O	2.0822121	-2.7433248	8.7016420
C	2.9676933	-4.0734595	6.5999727
H	3.3493723	-4.3664816	5.6187898
H	3.6978115	-4.3248276	7.3757418
H	2.0337655	-4.6007193	6.8120662
P	2.5305583	2.9442016	7.4819653
C	4.1900464	3.6957267	7.3656219
H	4.1915275	4.6188552	7.9564584
H	4.9495980	3.0079920	7.7363165
H	4.3895830	3.9415843	6.3170577
C	2.2100799	2.4446179	9.2005598
H	2.3250145	3.3262884	9.8403672
H	1.1884160	2.0661560	9.2885780
H	2.9267598	1.6769474	9.4960014
C	1.3723473	4.2905926	7.0883333
H	1.4349535	5.0209446	7.9033733
H	1.6696516	4.7849731	6.1572091
H	0.3599623	3.8919580	6.9997874
C	4.2700946	0.1387009	7.0303246
O	5.0416723	-0.6900853	6.3186759
O	4.6714620	0.7528509	8.0117314
C	6.3884248	-0.8956213	6.8074116
H	6.8585424	-1.5696180	6.0901756
H	6.9284895	0.0539685	6.8522507
H	6.3645464	-1.3488500	7.8025996
C	2.8228655	0.1522700	6.5345421
H	2.8098225	-0.3647564	5.5713526
H	1.5256679	1.1091127	4.3788966
C	1.6688470	1.8683844	5.1467028

3.4.7.7. **int1-regio-*syn-syn***
E(B3LYP-D3/6-31+G*(THF)) = -1894.477646
E(M06-2X-D3/6-311+G**) = -1894.134317
Gtot(B3LYP-D3/6-31+G*(THF)) = -1894.015904

C	-1.0896896	1.7451471	9.4304303
C	-0.1301979	0.2948606	8.1655362
C	0.2671845	-0.0360484	9.4769823
O	-0.3687642	0.9459139	10.2875316
O	0.9701762	-0.9173274	10.0150999
C	-3.4977282	4.8880703	11.0372470
C	-2.7404336	4.0768308	11.8876762
C	-1.9452108	3.0539708	11.3722972
C	-1.8910625	2.8177752	9.9831568
C	-2.6585336	3.6433516	9.1324709
C	-3.4485641	4.6619803	9.6551243
H	-4.1188077	5.6834004	11.4429381
H	-2.7707931	4.2398638	12.9633223
H	-1.3637609	2.4253608	12.0404057
H	-2.6258331	3.4645708	8.0612789
H	-4.0341616	5.2848160	8.9812721
N	-0.9685172	1.3894358	8.1736841
C	0.1681067	-0.4659023	6.8986940
H	-0.0064978	0.2164084	6.0570586
C	1.6884469	-0.8431912	6.8467682
C	3.5844751	-1.7854203	5.3919588
H	5.5570364	-2.0392029	6.1896738
C	-2.5700591	-3.7941894	6.3762521
C	-2.3994922	-2.8572721	5.3571524
C	-1.5023030	-1.7977746	5.5252691
C	-0.7669718	-1.6583386	6.7084267
C	-0.9484091	-2.6052275	7.7301294
C	-1.8404622	-3.6634302	7.5640257
H	-3.2728362	-4.6156476	6.2534143
H	-2.9692821	-2.9431648	4.4340046
H	-1.3845524	-1.0604153	4.7322011
H	-0.3866860	-2.4982159	8.6542435
H	-1.9765906	-4.3846737	8.3673266
H	1.9538355	-1.3317147	7.7853567
P	4.1272755	-1.5805764	3.6715782

C	3.5293708	-0.0003485	2.9995850
H	3.8942449	0.0999529	1.9714605
H	2.4358099	0.0232314	3.0027741
H	3.8954730	0.8252263	3.6152502
C	3.4488153	-2.9312982	2.6590258
H	3.6703905	-2.7335215	1.6043480
H	3.9063240	-3.8795280	2.9601394
H	2.3669552	-3.0009145	2.8069936
C	5.9404801	-1.6161151	3.5550193
H	6.2168607	-1.5142422	2.4991213
H	6.3766837	-0.7860024	4.1205971
H	6.3266627	-2.5676725	3.9356144
C	1.6118775	-3.2070968	5.7614407
O	1.8570130	-3.7038815	6.9761780
O	1.1734943	-3.8514561	4.8269467
C	1.4869009	-5.0857786	7.1931247
H	1.7216990	-5.2853908	8.2391805
H	0.4188233	-5.2141982	7.0047226
H	2.0639658	-5.7389977	6.5324121
C	2.0656237	-1.7460508	5.6412998
H	1.5570353	-1.3579030	4.7542288
H	4.1694491	-2.1454406	7.3933974
C	4.4850736	-1.9992511	6.3631030
C	2.4386674	0.4755741	6.7277607
O	3.0614281	0.8236144	7.8580621
O	2.4496019	1.1525733	5.7101889
C	3.6640608	2.1384402	7.8750465
H	4.0610494	2.2666293	8.8830261
H	4.4648636	2.2012916	7.1325956
H	2.9089547	2.8992904	7.6594735

3.4.7.8. **int1-regio-*syn-anti***
E(B3LYP-D3/6-31+G*(THF)) = -1894.471199
E(M06-2X-D3/6-311+G**) = -1894.132969
Gtot(B3LYP-D3/6-31+G*(THF)) = -1894.012315

C	-1.2874967	1.6589124	9.6820342
C	-0.3007933	0.3026344	8.3418847
C	0.0781102	-0.1175770	9.6346351
O	-0.5715193	0.8104014	10.5005603
O	0.7839450	-1.0250076	10.1190543
C	-3.7571017	4.7102922	11.3704421
C	-3.0352953	3.8518178	12.2053892
C	-2.2179810	2.8567668	11.6683269
C	-2.1064317	2.6976704	10.2717124
C	-2.8377477	3.5722977	9.4368405
C	-3.6494995	4.5609699	9.9808347
H	-4.3954108	5.4831358	11.7930029
H	-3.1113861	3.9558428	13.2861876
H	-1.6647707	2.1905725	12.3243183
H	-2.7567180	3.4524059	8.3605476
H	-4.2060169	5.2211975	9.3178103
N	-1.1496818	1.3813579	8.4099495
C	-0.0392804	-0.4087543	7.0358804
H	-0.2905794	0.2981659	6.2377724
C	1.4774695	-0.7431351	6.8993134
C	1.5789947	-0.7332311	4.2722413
H	0.1245627	-0.3075291	2.7607635
C	-2.8050541	-3.7502460	6.7753137
C	-2.9790024	-2.6221030	5.9722832
C	-2.0707345	-1.5628033	6.0467382
C	-0.9698212	-1.6132982	6.9130487
C	-0.8213292	-2.7394814	7.7389172
C	-1.7277713	-3.7976171	7.6666136
H	-3.5102965	-4.5770437	6.7222152
H	-3.8260741	-2.5599260	5.2917307
H	-2.2327140	-0.6732557	5.4404845
H	-0.0188834	-2.7603221	8.4698205
H	-1.6021804	-4.6580273	8.3211634
H	1.7621469	-1.3910528	7.7290806
P	2.8310040	0.2194868	3.3494717
C	4.4489814	0.2593626	4.1770975
H	5.1467543	0.8026729	3.5298756
H	4.3607902	0.7784967	5.1327892
H	4.8313571	-0.7560767	4.3279232
C	2.2634284	1.9196282	3.0427982
H	3.0328886	2.4591511	2.4788736
H	1.3370595	1.8999946	2.4592528
H	2.0832727	2.4077272	4.0031093
C	3.0906632	-0.5897270	1.7341426
H	3.8347106	-0.0264104	1.1592934
H	3.4497533	-1.6128820	1.8901453
H	2.1500598	-0.6221780	1.1744237
H	3.0416992	-1.4839038	5.6607077
C	1.9491102	-1.4450096	5.5689780
H	-0.4189278	-1.4048438	4.1363525
C	0.3725058	-0.8130223	3.6928776
C	2.2261372	0.5716228	7.0652883
O	2.8203468	0.6844384	8.2545518
O	2.2846669	1.4383421	6.2018505
C	3.3934362	1.9762742	8.5652120
H	3.7639837	1.8916614	9.5874977
H	4.2101421	2.2106037	7.8763782
H	2.6239634	2.7500210	8.4959261
C	1.5537862	-2.9376519	5.5386126
O	2.0589175	-3.5530224	6.6199348
O	0.9396931	-3.5077951	4.6610414
C	1.7457364	-4.9588237	6.7579376
H	2.2191282	-5.2725198	7.6893249
H	0.6626902	-5.0891049	6.8109356
H	2.1458436	-5.5225938	5.9106729

3.4.7.9. **int1-regio-*anti-syn***
E(B3LYP-D3/6-31+G*(THF)) = -1894.483977
E(M06-2X-D3/6-311+G**) = -1894.162650
Gtot(B3LYP-D3/6-31+G*(THF)) = -1894.025795

C	-0.8598509	0.8492426	9.6716027
C	-0.5679916	0.0304856	7.7000674
C	-1.5033311	1.0667242	7.5298758
O	-1.6645960	1.6022289	8.8522869
O	-2.1248110	1.5813123	6.5788864
C	-0.7120519	1.6203175	13.8725436
C	-1.5333997	2.3945992	13.0474491
C	-1.5887550	2.1561620	11.6735289
C	-0.8177697	1.1291460	11.0967241
C	0.0090116	0.3526080	11.9362748
C	0.0587742	0.5970920	13.3046388
H	-0.6736819	1.8070727	14.9432540
H	-2.1392534	3.1897548	13.4772165

H	-2.2326030	2.7585273	11.0389577
H	0.6015809	-0.4430740	11.4944447
H	0.6996859	-0.0158345	13.9356767
N	-0.1995527	-0.0797678	9.0264131
C	-0.0872764	-0.9288396	6.6498928
H	-0.2529040	-0.4531350	5.6789199
C	1.4495220	-1.1918258	6.8155922
H	1.5857955	-1.6630276	7.7917641
C	1.8178099	1.3082027	5.9936060
H	1.2119712	2.0448124	4.0821070
C	-2.1699706	-4.7401005	6.5673334
C	-2.0133725	-3.9957810	5.3969947
C	-1.3514645	-2.7647413	5.4336350
C	-0.8412782	-2.2589141	6.6352603
C	-1.0059934	-3.0133892	7.8059151
C	-1.6635027	-4.2430700	7.7728749
H	-2.6858627	-5.6977566	6.5432067
H	-2.4091681	-4.3699184	4.4548156
H	-1.2314631	-2.1889236	4.5176169
H	-0.6252077	-2.6228171	8.7455690
H	-1.7843157	-4.8153721	8.6907305
C	1.9236712	-2.2674788	5.8422903
O	1.8897064	-1.8756024	4.5514567
O	2.2332003	-3.3924381	6.1786707
C	2.1979611	-2.8962445	3.5741071
H	2.0761025	-2.4175068	2.6014264
H	3.2238260	-3.2532386	3.7029656
H	1.5056739	-3.7358788	3.6805439
P	1.7641358	2.9906383	6.6997212
C	3.1786147	3.9633860	6.0909991
H	3.1382823	4.9707819	6.5207030
H	4.1028369	3.4605936	6.3865877
H	3.1315585	4.0313314	4.9988083
C	1.8610952	2.9552128	8.5124515
H	1.8159965	3.9862299	8.8800534
H	1.0293116	2.3838511	8.9328338
H	2.8122464	2.5096799	8.8204352
C	0.2388068	3.8364896	6.1876737
H	0.1268252	4.7511945	6.7808690
H	0.3093391	4.1096629	5.1293262
H	-0.6277192	3.1799379	6.3333730
H	2.0202335	0.4648627	7.9336382
C	2.2424909	0.1529712	6.9067758
H	1.5480727	0.2327222	4.1968314
C	1.5155117	1.1965341	4.6925894
C	3.7695957	0.0546564	6.9164008
O	4.2165003	-1.1965571	7.0067963
O	4.4919566	1.0395266	6.9104495
C	5.6522857	-1.3799154	7.1027839
H	5.7999127	-2.4593164	7.1475396
H	6.1462117	-0.9543383	6.2253821
H	6.0311163	-0.8986981	8.0086412

3.4.7.10. **int1-regio-*anti-anti***

E(B3LYP-D3/6-31+G*(THF)) = -1894.484610

E(M06-2X-D3/6-311+G**) = -1894.167337

Gtot(B3LYP-D3/6-31+G*(THF)) = -1894.024396

C	-0.9398001	0.9290195	9.7433129
C	-0.4243956	0.1950090	7.7828948
C	-1.4511642	1.1351517	7.5639446
O	-1.7513576	1.6277444	8.8811739
O	-2.0627150	1.5941291	6.5815632
C	-1.1178022	1.6248418	13.9547834
C	-1.9701798	2.3238405	13.0946549
C	-1.9196149	2.1119093	11.7161823
C	-1.0090256	1.1876594	11.1708135
C	-0.1510974	0.4868113	12.0452756
C	-0.2073576	0.7043474	13.4179191
H	-1.1616744	1.7909189	15.0287417
H	-2.6832537	3.0387640	13.5001263
H	-2.5881553	2.6546414	11.0540631
H	0.5491827	-0.2293443	11.6245738
H	0.4600845	0.1514029	14.0763221
N	-0.1495975	0.0853130	9.1330449
C	0.0803382	-0.8088278	6.7897040
H	-0.0120899	-0.3732980	5.7903806
C	1.5853890	-1.1435509	7.0545984
H	1.6417863	-1.5235907	8.0736824
C	2.0281594	1.3016356	6.1925301
H	1.3659624	1.9961589	4.2692173
C	-2.1663576	-4.5309457	6.7741554
C	-1.9035441	-3.8610046	5.5781340
C	-1.1926675	-2.6564487	5.5909011
C	-0.7392417	-2.1040191	6.7945770
C	-1.0138338	-2.7824353	7.9911854
C	-1.7190634	-3.9850486	7.9819576
H	-2.7190178	-5.4682759	6.7681483
H	-2.2537721	-4.2723868	4.6334589
H	-0.9859650	-2.1421623	4.6539596
H	-0.6742811	-2.3550354	8.9305781
H	-1.9227659	-4.4983253	8.9199647
C	2.0165730	-2.2593432	6.1122774
O	2.0104314	-3.4540322	6.7158047
O	2.3075906	-2.0986812	4.9364054
C	2.2502156	-4.6025415	5.8710556
H	2.2208521	-5.4665582	6.5363935
H	1.4645614	-4.6740794	5.1139359
H	3.2261519	-4.5247992	5.3840398
P	1.7369815	2.9553116	6.9243263
C	3.2260785	3.9881348	6.6926061
H	2.9973141	5.0122980	7.0091728
H	4.0481833	3.5881839	7.2890222
H	3.5003498	3.9869435	5.6327694
C	1.2111720	3.0482666	8.6681008
H	1.7628690	3.8622668	9.1491044
H	0.1401514	3.2578330	8.6987280
H	1.4046001	2.1207683	9.1998995
C	0.4042559	3.7636112	5.9782568
H	0.1727403	4.7094651	6.4816558
H	0.7129404	3.9815877	4.9520695
H	-0.4847119	3.1216517	5.9809368
C	3.3245661	0.3846394	8.2145046
O	3.6251424	-0.7168882	8.9021676
O	3.6848683	1.5039934	8.5457650
C	4.3822103	-0.5379789	10.1246014
H	4.4932013	-1.5388633	10.5433650
H	5.3593519	-0.1001401	9.9026564
H	3.8348139	0.1111766	10.8132426
C	2.5832308	0.0717050	6.9212481

H	3.3989189	-0.2843693	6.2759721
H	1.9880864	0.2620297	4.3588539
C	1.7849870	1.1986475	4.8743301

3.4.7.11. **TS-cyclo-regio-*syn-syn-syn***
E(B3LYP-D3/6-31+G*(THF)) = -1894.469993
E(M06-2X-D3/6-311+G**) = -1894.157731
Gtot(B3LYP-D3/6-31+G*(THF)) = -1894.009262

C	-0.9825860	2.1236301	9.2223102
C	0.0157685	0.9816358	7.6737681
C	-1.0203455	1.7726641	7.0260721
O	-1.5719627	2.5647147	8.0460408
O	-1.3612559	1.9155132	5.8580042
C	-2.2439918	3.8696564	12.9023136
C	-2.8183314	4.2934377	11.7008096
C	-2.4124917	3.7329822	10.4891905
C	-1.4222174	2.7364150	10.4713540
C	-0.8468707	2.3133414	11.6839405
C	-1.2566130	2.8766689	12.8884898
H	-2.5622548	4.3092161	13.8449518
H	-3.5859240	5.0640027	11.7058748
H	-2.8602558	4.0628764	9.5565117
H	-0.0830649	1.5415859	11.6612128
H	-0.8067755	2.5423076	13.8207796
N	-0.1032467	1.1912335	9.0674021
C	0.3362607	-0.3985692	7.1172057
H	0.2385146	-0.2784819	6.0334683
C	1.8284718	-0.8608915	7.3118930
C	2.2388469	1.3546569	6.0441326
H	1.1871824	3.0324986	6.9197954
C	-2.5891872	-3.4351168	8.1594509
C	-2.2554042	-3.1891649	6.8260664
C	-1.3104063	-2.2090566	6.5134804
C	-0.6833475	-1.4590290	7.5186117
C	-1.0260244	-1.7164717	8.8543860
C	-1.9701283	-2.6944424	9.1701114
H	-3.3273781	-4.1944226	8.4092789
H	-2.7335068	-3.7544905	6.0286330
H	-1.0631805	-2.0185156	5.4702145
H	-0.5554207	-1.1456638	9.6452595
H	-2.2245924	-2.8775397	10.2121912
H	1.8629604	-1.9069420	6.9989871
P	1.9683806	1.8013324	4.3857859
C	0.7860136	0.7690444	3.4272820
H	0.7430912	1.0977387	2.3821609
H	1.1015178	-0.2799346	3.4543238
H	-0.1990260	0.8669664	3.8931714
C	3.5268134	1.7014943	3.4307896
H	3.3483919	1.9656831	2.3825126
H	4.2662325	2.3774823	3.8702670
H	3.9170355	0.6785223	3.4754956
C	1.3421593	3.5065781	4.2517073
H	1.2762546	3.7778797	3.1930108
H	0.3443055	3.5591526	4.6967943
H	2.0169692	4.1984171	4.7643134
C	2.7712645	-0.0374859	6.3474126
H	2.0669985	1.9706008	8.0827353
C	1.6035446	2.0437484	7.1039039
C	2.2298334	-0.9188803	8.7810704
O	2.8678181	0.1833357	9.2095224
O	1.9912603	-1.8692705	9.5031308
H	2.7878021	-0.6386425	5.4241301
C	3.1869957	0.2266571	10.6149790
H	3.6868733	1.1833513	10.7710866
H	2.2680029	0.1684522	11.2042318
H	3.8460983	-0.6034575	10.8845500
C	4.2453242	-0.0453500	6.7804126
O	4.6001232	-1.2612990	7.2502992
O	5.0266666	0.8763376	6.6625315
C	5.9630822	-1.3948223	7.7096153
H	6.0423813	-2.4077698	8.1066866
H	6.6635504	-1.2524419	6.8810147
H	6.1728989	-0.6582012	8.4904186

3.4.7.12. **TS-cyclo-regio-*syn-anti-anti***
E(B3LYP-D3/6-31+G*(THF)) = -1894.473607
E(M06-2X-D3/6-311+G**) = -1894.161501
Gtot(B3LYP-D3/6-31+G*(THF)) = -1894.014202

C	-0.8069383	2.0594873	9.1467606
C	0.0566705	0.8759533	7.5599793
C	-0.9241825	1.7374651	6.9454776
O	-1.3988498	2.5513528	7.9943075
O	-1.2865514	1.9220349	5.7848917
C	-1.7890946	3.8491872	12.8915385
C	-2.3936764	4.3145290	11.7205505
C	-2.0794568	3.7401837	10.4883666
C	-1.1514143	2.6871112	10.4173288
C	-0.5444480	2.2227029	11.5999979
C	-0.8632754	2.8005380	12.8249472
H	-2.0354259	4.2997443	13.8503819
H	-3.1133164	5.1288934	11.7658739
H	-2.5505135	4.1025519	9.5794989
H	0.1744399	1.4107459	11.5386952
H	-0.3886651	2.4343314	13.7326937
N	0.0007816	1.0678628	8.9505060
C	0.3587836	-0.4857046	6.9606059
H	0.2255513	-0.3739929	5.8825362
C	1.8366467	-0.9667153	7.1782933
H	2.0426861	-0.9480476	8.2519442
C	2.3579419	1.2606235	5.9802280
H	1.2807433	2.9660249	6.7744527
C	-2.4861646	-3.5024714	8.2474603
C	-2.3752016	-3.1644070	6.8974258
C	-1.4583420	-2.1897559	6.4931010
C	-0.6450756	-1.5367664	7.4275424
C	-0.7632763	-1.8859118	8.7812923
C	-1.6755467	-2.8589948	9.1876050
H	-3.1995880	-4.2598179	8.5661583
H	-3.0022208	-3.6569563	6.1570203
H	-1.3744572	-1.9343839	5.4385619
H	-0.1462439	-1.3807579	9.5168797
H	-1.7555520	-3.1151647	10.2422230
C	1.9258303	-2.4034578	6.6873315
O	1.9920833	-3.2888361	7.6897571
O	1.8978749	-2.7195808	5.5076854
C	1.9293395	-4.6835417	7.3131867
H	1.9997349	-5.2437186	8.2465992
H	0.9800461	-4.8914430	6.8116538

H	2.7575301	-4.9410624	6.6471278
P	2.0727306	1.5883812	4.2858308
C	0.8821717	0.4820830	3.4351298
H	0.8746368	0.6806917	2.3572099
H	1.1614658	-0.5626376	3.6114076
H	-0.1099774	0.6719908	3.8563717
C	3.6257480	1.4285789	3.3334843
H	3.4388812	1.6382532	2.2742689
H	4.3660841	2.1320113	3.7266527
H	4.0171860	0.4093085	3.4247370
C	1.4478964	3.2819837	4.0532997
H	1.3627366	3.4860895	2.9809438
H	0.4587372	3.3621803	4.5135457
H	2.1339647	4.0020485	4.5094863
C	4.1690786	-0.0088466	7.2358227
O	4.6952162	-1.2406850	7.4012854
O	4.6446168	0.9934570	7.7315576
C	5.8547909	-1.3353792	8.2602542
H	6.1347333	-2.3901572	8.2624942
H	6.6716315	-0.7230872	7.8670047
H	5.6057273	-1.0017018	9.2719187
C	2.8970747	-0.0948155	6.3840803
H	3.1650976	-0.6827511	5.4997736
H	2.1293601	1.9243870	7.9911209
C	1.7202084	1.9942303	6.9862890

3.4.7.13. **TS-cyclo-regio-*anti-anti-anti***
E(B3LYP-D3/6-31+G*(THF)) = -1894.479746
E(M06-2X-D3/6-311+G**) = -1894.164504
Gtot(B3LYP-D3/6-31+G*(THF)) = -1894.020050

C	-1.2685936	2.7156782	7.2421226
C	0.0336810	1.0013988	7.2128069
C	0.3804358	1.7281327	8.3679146
O	-0.4500928	2.8742397	8.3382443
O	1.2374237	1.6040235	9.2664968
C	-4.3272098	5.6028778	6.5102379
C	-3.4565909	5.7645036	7.5920057
C	-2.4489448	4.8313464	7.8351217
C	-2.2958738	3.7127584	6.9939457
C	-3.1763492	3.5578837	5.9040316
C	-4.1792111	4.4932747	5.6682545
H	-5.1143868	6.3304608	6.3254763
H	-3.5643945	6.6216963	8.2535684
H	-1.7788435	4.9589041	8.6802344
H	-3.0648687	2.6916047	5.2580874
H	-4.8540020	4.3556032	4.8256911
N	-1.0213221	1.6276015	6.5642152
C	0.3429644	-0.4454715	6.9017721
H	0.1377340	-0.5977104	5.8350390
C	1.8098756	-0.9550605	7.1517803
H	2.0589259	-0.8265200	8.2071822
C	3.0103311	1.2337405	6.5543019
H	1.8705459	3.0225296	6.1365973
C	-2.4002243	-2.9661833	9.1445589
C	-2.4009747	-2.9995988	7.7489862
C	-1.5127300	-2.1967560	7.0270440
C	-0.6204931	-1.3450919	7.6874653
C	-0.6271021	-1.3182213	9.0911769
C	-1.5078177	-2.1224928	9.8135825
H	-3.0920223	-3.5884991	9.7086573
H	-3.0944275	-3.6494460	7.2189163
H	-1.5115056	-2.2313983	5.9398340
H	0.0486981	-0.6473920	9.6159055
H	-1.5032683	-2.0867644	10.9012641
C	1.7791650	-2.4457770	6.8128760
O	2.1791035	-3.2045721	7.8470113
O	1.4287568	-2.8942390	5.7375499
C	2.1181729	-4.6340833	7.6371984
H	2.4615220	-5.0832565	8.5703719
H	1.0901051	-4.9368623	7.4195618
H	2.7650029	-4.9255840	6.8049939
P	4.0852897	2.0766605	7.7332467
C	5.7381395	2.3842314	7.0094370
H	6.3240126	2.9961115	7.7050773
H	6.2527383	1.4401471	6.8260051
H	5.6154170	2.9280627	6.0666661
C	4.3236657	1.3228540	9.3768849
H	4.9123302	2.0241051	9.9800864
H	3.3385161	1.1791596	9.8228310
H	4.8529895	0.3752861	9.2874459
C	3.4180028	3.7453375	8.0557408
H	4.1060515	4.2482284	8.7441016
H	3.3587141	4.3340057	7.1355422
H	2.4348986	3.6551616	8.5229087
C	4.2672224	-1.0024002	6.4899306
O	4.3355407	-2.1205268	5.7589015
O	5.1613070	-0.6445935	7.2385937
C	5.5000715	-2.9565873	5.9695900
H	5.3667980	-3.8097945	5.3035817
H	6.4119922	-2.4088977	5.7172377
H	5.5444394	-3.2800677	7.0134363
C	2.9459453	-0.2696714	6.2914028
H	2.6629359	-0.4172729	5.2420597
H	1.4500349	1.5495152	5.1459766
C	2.0237683	1.9620296	5.9713365

3.4.7.14. **int3-regio-*syn-syn-syn***
E(B3LYP-D3/6-31+G*(THF)) = -1894.477743
E(M06-2X-D3/6-311+G**) = -1894.174328
Gtot(B3LYP-D3/6-31+G*(THF)) = -1894.01394

C	-0.7560969	2.1586586	9.0706527
C	0.4462688	1.2147104	7.4739443
C	-0.7750649	1.8982500	6.8710413
O	-1.4613514	2.4934990	7.9072001
O	-1.1570664	1.9905195	5.7271319
C	-2.4244124	3.4531493	12.7702670
C	-3.0881238	3.7430387	11.5753671
C	-2.5479295	3.3354397	10.3551907
C	-1.3345841	2.6300336	10.3294502
C	-0.6695588	2.3398740	11.5327504
C	-1.2130782	2.7514219	12.7457466
H	-2.8491934	3.7717119	13.7196476
H	-4.0299337	4.2864071	11.5923078
H	-3.0662814	3.5575421	9.4273107
H	0.2671368	1.7918567	11.4967223
H	-0.6952213	2.5250126	13.6748134
N	0.2953405	1.4541991	8.9034967
C	0.4199922	-0.2851403	7.0398404

H	0.1753593	-0.2181893	5.9744349
C	1.7880428	-1.0226840	7.0252658
C	2.3997037	1.1838371	5.7578188
H	1.5893389	2.9568118	6.7642798
C	-2.7831636	-2.5612679	8.8742612
C	-2.7995137	-2.2841942	7.5057796
C	-1.7602382	-1.5499125	6.9292654
C	-0.6903882	-1.0818155	7.7045140
C	-0.6898276	-1.3610447	9.0802309
C	-1.7249412	-2.0951952	9.6591142
H	-3.5898456	-3.1335504	9.3275331
H	-3.6201742	-2.6385412	6.8854224
H	-1.7817335	-1.3354192	5.8623717
H	0.1111130	-0.9798047	9.7040908
H	-1.7069063	-2.3005804	10.7273504
H	1.5863295	-1.9644067	6.5057520
P	2.0341396	1.6811128	4.1546782
C	0.6904478	0.8944154	3.1521644
H	0.6088478	1.3699994	2.1671708
H	0.9170267	-0.1667340	3.0298019
H	-0.2500441	1.0119208	3.6962486
C	3.4738757	1.6404052	3.0119925
H	3.1860350	2.0557724	2.0391696
H	4.2860371	2.2359273	3.4401154
H	3.8116096	0.6120696	2.8706583
C	1.5376247	3.4452367	4.1742090
H	1.4492330	3.7885994	3.1382554
H	0.5678622	3.5607584	4.6637137
H	2.2874352	4.0520248	4.6901596
H	3.7318184	-0.0400663	6.8913890
C	2.8928871	-0.1820039	6.2000256
H	2.4632089	1.8381758	7.7950955
C	1.7870930	1.8936391	6.9317699
C	2.3342566	-1.4554834	8.3722963
O	2.7148194	-0.4093985	9.1239846
O	2.4442013	-2.6145557	8.7335824
C	3.3765830	-1.1357931	5.1274063
O	4.3965686	-1.8906693	5.5716482
O	2.8780460	-1.2852544	4.0228505
C	3.2463525	-0.7101269	10.4299466
H	3.4557810	0.2577060	10.8873269
H	2.5125252	-1.2659566	11.0214678
H	4.1628767	-1.3019118	10.3436696
C	4.8587996	-2.9293354	4.6770401
H	5.6536517	-3.4471769	5.2155604
H	4.0429363	-3.6174219	4.4382489
H	5.2438718	-2.4894796	3.7525054

3.4.7.15. ***6-cis-cis***
E(B3LYP-D3/6-31+G*(THF)) = -1433.395839
E(M06-2X-D3/6-311+G**) = -1433.147253
Gtot(B3LYP-D3/6-31+G*(THF)) = -1433.042738

C	0.1445952	-0.1300296	5.4887212
C	-0.9630661	0.5667115	5.1956308
C	-1.9906018	0.9619060	6.2374489
C	-1.8041938	0.1420619	7.5559827
C	-0.3010241	0.0317333	7.9869853
C	0.5200407	-0.6147468	6.8536103
H	-2.0691255	-0.8847958	7.2743481
H	0.3754285	-1.7048304	6.8959977
H	0.8186003	-0.3953680	4.6770824
H	1.5889082	-0.4444968	7.0337804
C	-4.3753504	1.1141086	10.9152078
C	-4.4451584	-0.1343306	10.2945013
C	-3.6222430	-0.4192605	9.2029762
C	-2.7188949	0.5328385	8.7083259
C	-2.6601784	1.7850674	9.3422399
C	-3.4794378	2.0719101	10.4341883
H	-5.0107041	1.3382751	11.7693180
H	-5.1352557	-0.8907644	10.6620870
H	-3.6746559	-1.4000868	8.7345812
H	-1.9618630	2.5380905	8.9944657
H	-3.4135671	3.0463243	10.9130673
C	0.4776966	1.2411535	9.6569125
N	0.2423933	1.3188603	8.4037749
C	1.6752572	4.4424543	12.1895098
C	1.5613906	3.1541849	12.7193714
C	1.1802496	2.0904155	11.9012285
C	0.9146710	2.3192818	10.5427969
C	1.0281780	3.6158319	10.0109619
C	1.4079134	4.6713784	10.8342283
H	1.9695075	5.2696064	12.8318391
H	1.7671041	2.9774674	13.7722452
H	1.0851550	1.0899123	12.3121197
H	0.8092745	3.7770959	8.9592401
H	1.4926120	5.6743286	10.4226461
C	-0.2161964	-0.8274327	9.2543666
O	0.2439585	-0.0031711	10.2571693
O	-0.4764905	-1.9885224	9.4321535
C	-1.1992366	0.9414763	3.7699320
O	-2.3704214	1.5933916	3.6212110
O	-0.4520893	0.6923122	2.8362108
C	-2.7222376	1.9884201	2.2775186
H	-3.7065697	2.4523120	2.3587683
H	-1.9915010	2.7014596	1.8843307
H	-2.7638280	1.1122284	1.6238044
H	-2.9858392	0.6892835	5.8722747
C	-2.0232065	2.4897595	6.3852743
O	-3.2830134	2.9113057	6.6038545
O	-1.0697803	3.2371541	6.2973569
C	-3.4513442	4.3287970	6.8129530
H	-4.5232487	4.4809255	6.9483837
H	-2.9053501	4.6470969	7.7061041
H	-3.0878040	4.8911794	5.9482432

3.4.7.16. ***6-cis-trans***
E(B3LYP-D3/6-31+G*(THF)) = -1433.400148
Gtot(B3LYP-D3/6-31+G*(THF)) = -1433.047103

C	-0.0314634	-0.1281561	5.4412418
C	-1.1677356	0.5520721	5.2211096
C	-2.1269753	0.9136871	6.3463722
C	-1.8865113	0.0106800	7.5914292
C	-0.3604746	-0.0088441	7.9452839
C	0.4300674	-0.6234276	6.7761335
H	-1.9807437	1.9622769	6.6189775
H	-2.1265986	-1.0171961	7.2961054
H	0.3270452	-1.7179974	6.8182220
H	0.6162828	-0.3389434	4.5932288

H	1.4997958	-0.4146750	6.9043254
C	-4.2956020	0.9412582	11.0701127
C	-4.1946383	-0.3706095	10.6028833
C	-3.4294506	-0.6505961	9.4693172
C	-2.7531946	0.3708540	8.7872486
C	-2.8653038	1.6861787	9.2631430
C	-3.6299802	1.9678811	10.3955123
H	-4.8902189	1.1634303	11.9536374
H	-4.7094143	-1.1777444	11.1196562
H	-3.3466520	-1.6758862	9.1141206
H	-2.3520500	2.4952798	8.7532063
H	-3.7059818	2.9927408	10.7515929
C	0.4145373	1.3069954	9.5367307
N	0.1042142	1.3314551	8.2968125
C	1.6008517	4.6134265	11.9404684
C	1.5353920	3.3380054	12.5077117
C	1.1570595	2.2435112	11.7299569
C	0.8431595	2.4272558	10.3743231
C	0.9099964	3.7109799	9.8054937
C	1.2882045	4.7975402	10.5881633
H	1.8934530	5.4650312	12.5508021
H	1.7771923	3.1947483	13.5580651
H	1.1015580	1.2529803	12.1708130
H	0.6602920	3.8415648	8.7565465
H	1.3371971	5.7901333	10.1469028
C	-0.1431277	-0.8199455	9.2241329
O	0.3061977	0.0664975	10.1778932
O	-0.3009336	-1.9915569	9.4469335
C	-1.4246689	1.0155026	3.8245109
O	-2.4913341	1.8345639	3.7613975
O	-0.7595245	0.7124699	2.8452393
C	-2.8429705	2.3401106	2.4546674
H	-3.7412735	2.9397396	2.6075949
H	-2.0313478	2.9556002	2.0560171
H	-3.0422132	1.5122296	1.7681845
C	-3.6048620	0.7931395	5.9710415
O	-3.8713283	-0.4040650	5.4168532
O	-4.4470663	1.6365624	6.2004680
C	-5.2529636	-0.6482190	5.0687488
H	-5.2738531	-1.6508503	4.6391763
H	-5.8841950	-0.5971906	5.9605568
H	-5.5968370	0.0903906	4.3392748

3.4.7.17. ***6-trans-cis***
E(B3LYP-D3/6-31+G*(THF)) = -1433.391707
Gtot(B3LYP-D3/6-31+G*(THF)) = -1433.038468

C	0.2052439	-0.0366703	5.3895145
C	-0.9297319	0.6386460	5.1556075
C	-1.9900708	0.8836843	6.2099320
C	-1.6787006	0.1419129	7.5578830
C	-0.1527626	-0.0018090	7.9011112
C	0.5945454	-0.6298462	6.7076746
H	-1.9671761	-0.8983987	7.3669675
H	0.3715476	-1.7071930	6.7021239
H	0.8865810	-0.2019674	4.5577890
H	1.6790486	-0.5520372	6.8546922
C	-3.9867356	1.3685678	11.0216519
C	-4.1783995	0.1131324	10.4422039
C	-3.4365488	-0.2592688	9.3194654
C	-2.4986640	0.6120367	8.7524091
C	-2.3172509	1.8729187	9.3418290
C	-3.0529950	2.2471232	10.4667665
H	-4.5577406	1.6593189	11.9007678
H	-4.8996951	-0.5815579	10.8676637
H	-3.5804360	-1.2453860	8.8821274
H	-1.6048860	2.5797304	8.9250466
H	-2.8922199	3.2268703	10.9112512
C	0.4694176	-0.1489289	10.0209627
N	-0.0461165	-0.8561909	9.0888863
C	1.0785340	-1.2870939	14.0786583
C	1.4729159	-0.0166161	13.6510092
C	1.2848924	0.3654812	12.3227667
C	0.6971573	-0.5303764	11.4156703
C	0.2985739	-1.8065384	11.8487503
C	0.4914789	-2.1810032	13.1750819
H	1.2265849	-1.5816424	15.1152736
H	1.9274143	0.6788867	14.3523345
H	1.5893304	1.3532472	11.9907639
H	-0.1617341	-2.4896159	11.1409035
H	0.1825526	-3.1690066	13.5079620
C	0.5468704	1.2944750	8.3293670
O	0.8488954	1.1470725	9.6735423
O	0.8688310	2.2726191	7.7128717
C	-1.1820718	1.1042358	3.7594319
O	-2.4270539	1.6038304	3.6228083
O	-0.3872541	1.0376926	2.8348012
C	-2.7845503	2.0874945	2.3100034
H	-3.8300636	2.3898747	2.3858651
H	-2.1559949	2.9394580	2.0344776
H	-2.6667101	1.2933790	1.5671889
H	-2.9297073	0.4429378	5.8589717
C	-2.2958763	2.3808988	6.3494494
O	-3.6041693	2.5654027	6.6021750
O	-1.4938427	3.2874955	6.2530779
C	-4.0137176	3.9242225	6.8641358
H	-5.0946792	3.8827700	7.0055878
H	-3.5248487	4.2931653	7.7703486
H	-3.7587113	4.5730565	6.0218857

3.4.7.18. ***6-trans-trans***
E(B3LYP-D3/6-31+G*(THF)) = -1433.399405
Gtot(B3LYP-D3/6-31+G*(THF)) = -1433.049328

C	-0.0353762	-0.0978789	5.3999239
C	-1.1885268	0.5577658	5.1923445
C	-2.1346825	0.9155124	6.3279294
C	-1.8528403	0.0400315	7.5840221
C	-0.3235458	0.0180760	7.9204930
C	0.4564999	-0.5770568	6.7301424
H	-2.0092847	1.9736323	6.5738688
H	-2.0865644	-0.9961904	7.3126077
H	0.3627137	-1.6715503	6.7862195
H	0.6039895	-0.3012600	4.5435095
H	1.5293481	-0.3672604	6.8331974
C	-4.1960210	1.0077573	11.0982814
C	-4.0913572	-0.3112602	10.6517082
C	-3.3472682	-0.6035576	9.5078904
C	-2.6997335	0.4114012	8.7916589
C	-2.8165831	1.7335310	9.2447718

C	-3.5565738	2.0284613	10.3910726
H	-4.7727563	1.2394430	11.9911463
H	-4.5853992	-1.1140055	11.1949860
H	-3.2569428	-1.6342973	9.1710297
H	-2.3328436	2.5452020	8.7075478
H	-3.6344501	3.0591139	10.7300798
C	0.3708099	0.0323704	10.0177168
N	-0.0908615	-0.7660987	9.1331792
C	1.2913514	-0.8653413	14.0754174
C	1.5083530	0.4263996	13.5885736
C	1.2151393	0.7328395	12.2597460
C	0.6994167	-0.2609868	11.4125347
C	0.4811196	-1.5593127	11.9046205
C	0.7777030	-1.8569241	13.2310946
H	1.5216491	-1.1010269	15.1120484
H	1.9064350	1.1972389	14.2439893
H	1.3827212	1.7366127	11.8815106
H	0.0796085	-2.3201791	11.2416672
H	0.6086120	-2.8620168	13.6097937
C	0.1857540	1.4144333	8.2871244
O	0.5749543	1.3557984	9.6076347
O	0.2660486	2.4253948	7.6382605
C	-1.4771985	1.0074638	3.7980687
O	-2.5320669	1.8432075	3.7577786
O	-0.8440139	0.6844153	2.8043050
C	-2.9054066	2.3509953	2.4583714
H	-3.7923878	2.9624500	2.6294923
H	-2.0946749	2.9566031	2.0427329
H	-3.1289081	1.5253034	1.7767072
C	-3.6158983	0.7558848	5.9810811
O	-3.8607774	-0.4452981	5.4245037
O	-4.4758559	1.5749152	6.2301651
C	-5.2422827	-0.7217479	5.1027559
H	-5.2478232	-1.7231466	4.6696994
H	-5.8567221	-0.6888021	6.0071533
H	-5.6185471	0.0109828	4.3830598

3.4.8. PPh_3 ylide

3.4.8.1. NMe_3

E(B3LYP-D3/6-31+G*(THF)) = -1036.337960
E(M06-2X-D3/6-311+G**) = -1036.164374
Gtot(B3LYP-D3/6-31+G*(THF)) = -1036.10842

P	0.0500838	0.8028450	6.4752910
C	-1.5577126	-0.4395459	10.6728383
C	-1.7105480	0.8864678	10.2607663
C	-1.2397616	1.2937998	9.0097486
C	-0.6014147	0.3799821	8.1572752
C	-0.4432387	-0.9497053	8.5861263
C	-0.9246853	-1.3586584	9.8308110
H	-1.9259998	-0.7546730	11.6467560
H	-2.2016055	1.6063976	10.9123234
H	-1.3738780	2.3258601	8.6976914
H	0.0591942	-1.6712756	7.9430277
H	-0.7979710	-2.3917815	10.1474617
C	0.0286140	5.4654941	6.5539701
C	-1.1002217	4.7955034	6.0750761
C	-1.1271555	3.3987364	6.0388988
C	-0.0210837	2.6531134	6.4764430
C	1.1131590	3.3376000	6.9462843
C	1.1353937	4.7327544	6.9918518
H	0.0480190	6.5529118	6.5810177
H	-1.9646083	5.3602314	5.7312767
H	-2.0139798	2.8902032	5.6704542
H	1.9862384	2.7768669	7.2773144
H	2.0194138	5.2480168	7.3613901
C	-3.4413312	-0.2858998	3.5808478
C	-2.1438937	-0.0665472	3.1092253
C	-1.1182956	0.2457429	4.0038573
C	-1.3772341	0.3579005	5.3805570
C	-2.6836545	0.1343448	5.8437387
C	-3.7073178	-0.1880527	4.9492217
H	-4.2400849	-0.5367534	2.8860902
H	-1.9286197	-0.1455596	2.0456422
H	-0.1083825	0.4028454	3.6269377
H	-2.9051452	0.2163887	6.9042988
H	-4.7153454	-0.3592907	5.3216091

3.4.8.2. TS ylide formation

E(B3LYP-D3/6-31+G*(THF)) = -1648.083961
E(M06-2X-D3/6-311+G**) = -1647.807775
Gtot(B3LYP-D3/6-31+G*(THF)) = -1647.697832

C	-0.6887001	0.1174161	5.9765378
C	-1.2248284	1.3494433	5.4508558
O	-2.5288429	1.5490342	5.8346848
O	-0.6367704	2.1675611	4.7347437
C	-1.5035327	-0.7248452	6.9592268
H	-1.6312843	-0.2184947	7.9219564
H	-2.5008063	-0.9078542	6.5426813
C	-0.8394922	-2.0575995	7.2384531
O	-0.9636244	-2.9050915	6.1989759
O	-0.2408622	-2.3447571	8.2613903
C	-0.2808325	-4.1696727	6.3345488
H	-0.4778134	-4.7129195	5.4088990
H	0.7932665	-4.0060056	6.4607330
H	-0.6653743	-4.7251204	7.1950159
C	-3.1331776	2.7629774	5.3654989
H	-4.1444868	2.7702584	5.7789208
H	-2.5759481	3.6382619	5.7156135
H	-3.1737350	2.7885065	4.2710362
H	2.5353784	-0.8809487	5.0885432
C	1.4574607	-0.8385270	4.9549028
H	1.0398109	-1.3804915	4.1058264
C	0.6427593	-0.1650625	5.7532832
P	2.0379014	0.7263396	7.3931148
C	-0.9589567	1.5630885	10.8116955
C	-0.9864262	2.2966725	9.6222913
C	-0.0395873	2.0543367	8.6275196
C	0.9448882	1.0656090	8.8133496
C	0.9668107	0.3328287	10.0090495
C	0.0230682	0.5876246	11.0043501
H	-1.6964078	1.7549835	11.5878900
H	-1.7437929	3.0619662	9.4699448
H	-0.0596500	2.6404613	7.7124258
H	1.7189180	-0.4326408	10.1703328
H	0.0551296	0.0203722	11.9320386
C	3.5776072	4.9435029	6.2638714
C	3.7658329	4.4665066	7.5663400

C	3.3137567	3.1949955	7.9142533
C	2.6805237	2.3823649	6.9554387
C	2.4841164	2.8694826	5.6558007
C	2.9331958	4.1488608	5.3145991
H	3.9250413	5.9388338	5.9955647
H	4.2563505	5.0889763	8.3114851
H	3.4455839	2.8344940	8.9316398
H	1.9437837	2.2708735	4.9293501
H	2.7695191	4.5251840	4.3074356
C	5.5321539	-2.0195887	8.7098908
C	5.8102119	-0.7071614	8.3210672
C	4.7748668	0.1534904	7.9510240
C	3.4422864	-0.2938107	7.9681180
C	3.1713467	-1.6280886	8.3329678
C	4.2106570	-2.4765963	8.7137859
H	6.3416444	-2.6851581	9.0007397
H	6.8369377	-0.3473994	8.3074961
H	5.0098291	1.1702106	7.6516941
H	2.1499347	-2.0014498	8.3168693
H	3.9881311	-3.5001284	9.0071680

3.4.8.3. Ylide *Z*
E(B3LYP-D3/6-31+G*(THF)) = -1648.101348
E(M06-2X-D3/6-311+G**) = -1647.823375
Gtot(B3LYP-D3/6-31+G*(THF)) = -1647.71086

C	-0.7947816	0.3287245	6.1705892
C	-1.6691405	1.4088707	5.9523214
O	-2.9373802	1.2216957	6.4723177
O	-1.3921865	2.4716049	5.3464382
C	-1.2858096	-0.9534958	6.8002908
H	-0.4229419	-1.5514001	7.1310462
H	-1.9014342	-0.7473402	7.6812372
C	-2.1321336	-1.8762949	5.9333619
O	-1.5975054	-2.0662683	4.7094353
O	-3.1630666	-2.4214655	6.2938322
C	-2.3205340	-2.9511903	3.8303109
H	-1.7395191	-2.9862520	2.9071244
H	-2.4004699	-3.9513724	4.2680905
H	-3.3252058	-2.5641849	3.6344675
C	-3.8309135	2.3246595	6.3123907
H	-4.7868598	1.9994692	6.7313089
H	-3.4691874	3.2098273	6.8485134
H	-3.9575390	2.5882151	5.2564921
H	2.4008022	-0.5100815	5.0458261
C	1.3394293	-0.6008538	5.2426436
H	0.8840607	-1.5705733	5.0653096
C	0.5603199	0.4238914	5.6947081
P	1.3849632	2.0671552	5.8280756
C	1.2126200	4.5279227	1.9304413
C	1.6133198	5.1473158	3.1166169
C	1.6567517	4.4189115	4.3063758
C	1.2916097	3.0637020	4.3116491
C	0.8870766	2.4444400	3.1212387
C	0.8515600	3.1770616	1.9350902
H	1.1794744	5.0970185	1.0041437
H	1.8940655	6.1977911	3.1205346
H	1.9734127	4.9102241	5.2214796
H	0.5936987	1.3992836	3.1282757
H	0.5354875	2.6931501	1.0137469
C	5.9106692	1.3856040	6.6850921
C	5.5118344	2.3555989	5.7650007
C	4.1566946	2.5448708	5.4835426
C	3.1803110	1.7637086	6.1205631
C	3.5933547	0.7771270	7.0365720
C	4.9467220	0.5950241	7.3180039
H	6.9663090	1.2384820	6.9018766
H	6.2539249	2.9667588	5.2563830
H	3.8742607	3.2984949	4.7573671
H	2.8645410	0.1242583	7.5070564
H	5.2476131	-0.1738816	8.0257214
C	0.0342627	4.4652967	9.5611797
C	-0.3493241	4.8939056	8.2880640
C	0.0375117	4.1783607	7.1540243
C	0.8338587	3.0310687	7.2859469
C	1.2180947	2.6032826	8.5670055
C	0.8133209	3.3138986	9.6975461
H	-0.2732901	5.0236002	10.4423915
H	-0.9606946	5.7858109	8.1724732
H	-0.3032593	4.4929294	6.1769851
H	1.8340789	1.7205044	8.6997588
H	1.1140403	2.9669448	10.6834813

3.4.8.4. Ylide *E*
E(B3LYP-D3/6-31+G*(THF)) = -1648.097392
E(M06-2X-D3/6-311+G**) = -1647.816241
Gtot(B3LYP-D3/6-31+G*(THF)) = -1647.708455

C	-0.3671731	0.2432395	6.0311860
C	-1.3523659	1.1615216	6.4929292
O	-2.6271799	0.5960454	6.4615309
O	-1.2137651	2.3251309	6.9202363
C	-0.8231565	-1.1356193	5.6176165
H	-1.6797290	-1.0846309	4.9342147
H	-0.0505852	-1.6737709	5.0561459
C	-1.2427287	-2.0640196	6.7536927
O	-1.8679751	-3.1626760	6.2622512
O	-1.0281168	-1.9089641	7.9401878
C	-2.2277753	-4.1680513	7.2300134
H	-2.7231444	-4.9619535	6.6676324
H	-1.3326761	-4.5567092	7.7256477
H	-2.9031853	-3.7567733	7.9857642
C	-3.6731302	1.4096166	6.9881858
H	-4.5908292	0.8246948	6.8796778
H	-3.5052854	1.6434848	8.0458552
H	-3.7659869	2.3526947	6.4367575
H	0.7810328	2.7247351	5.5783651
C	1.4795341	1.9358390	5.8338361
H	2.5088833	2.2259876	6.0054603
C	1.0118882	0.6652040	6.0057779
P	2.2882769	-0.4618889	6.7050726
C	1.7956083	-0.4473784	11.2888681
C	1.0530145	0.4321774	10.4972793
C	1.1934095	0.4255730	9.1088703
C	2.0887362	-0.4740495	8.5096263
C	2.8327856	-1.3627799	9.3061240
C	2.6874947	-1.3441288	10.6929765
H	1.6777244	-0.4373284	12.3701663
H	0.3560296	1.1262294	10.9605279
H	0.6049222	1.1066482	8.5022832

H	3.5171018	-2.0740198	8.8505579
H	3.2663145	-2.0325891	11.3040989
C	6.5337125	0.9831625	5.5549136
C	5.5272812	0.9007176	4.5880417
C	4.2441555	0.4857568	4.9443276
C	3.9595645	0.1385062	6.2791892
C	4.9694154	0.2409020	7.2481380
C	6.2504680	0.6592411	6.8836562
H	7.5332308	1.3075226	5.2747099
H	5.7370249	1.1666691	3.5547861
H	3.4638154	0.4630493	4.1892304
H	4.7647719	0.0062599	8.2869762
H	7.0250576	0.7348642	7.6431283
C	2.1732577	-4.8625086	5.2353464
C	2.7190896	-3.8618461	4.4267303
C	2.7535769	-2.5408434	4.8717372
C	2.2335366	-2.2045894	6.1341135
C	1.6817917	-3.2136684	6.9399553
C	1.6556035	-4.5344676	6.4895214
H	2.1545850	-5.8933731	4.8890125
H	3.1267200	-4.1091705	3.4493159
H	3.1926948	-1.7845641	4.2300430
H	1.2527562	-2.9714467	7.9040195
H	1.2327850	-5.3087398	7.1253308

3.4.8.5. Addition TS *Z*
E(B3LYP-D3/6-31+G*(THF)) = -2469.693030
E(M06-2X-D3/6-311+G**) = -2469.250575
Gtot(B3LYP-D3/6-31+G*(THF)) = -2468.957654

C	-0.9028133	0.9760492	10.0862991
C	-0.4990573	0.1494487	8.1329838
C	-1.0257044	1.4672808	7.9110192
O	-1.3086135	1.9554606	9.2169345
O	-1.2819135	2.1687960	6.9351226
C	-1.0384013	1.7102801	14.2827719
C	-1.5233885	2.6713761	13.3907735
C	-1.4925789	2.4383842	12.0165158
C	-0.9730309	1.2320439	11.5153084
C	-0.4921840	0.2657343	12.4190916
C	-0.5236365	0.5056609	13.7889131
H	-1.0628130	1.8958384	15.3543648
H	-1.9278481	3.6082292	13.7678541
H	-1.8642920	3.1878875	11.3243578
H	-0.0949525	-0.6670042	12.0296404
H	-0.1467324	-0.2481390	14.4770662
N	-0.4435877	-0.0920111	9.4995876
C	-0.1704984	-0.7698545	7.0945911
H	-0.3748941	-0.3372042	6.1184447
C	1.8334706	-1.1072233	6.6780908
H	2.0386739	-1.8302947	7.4572807
H	1.6014106	-1.5803179	5.7282021
C	2.8584595	0.8147036	5.4594491
C	2.6506238	0.0620984	6.6132395
P	3.5551164	0.4620413	8.1745272
C	3.3974800	2.1669993	5.5290481
O	3.5151746	2.7522615	4.3120184
O	3.7018577	2.7607551	6.5653952
C	3.9844796	4.1138811	4.3153015
H	3.9900377	4.4232877	3.2687656
H	3.3127694	4.7504241	4.8992746
H	4.9916188	4.1727213	4.7388305
C	2.5444352	0.2583215	4.0870657
H	3.3326206	0.5402391	3.3829785
H	2.5339429	-0.8390651	4.1049376
C	1.2381979	0.6826078	3.4255304
O	0.3222550	1.0898424	4.3095625
O	1.0448298	0.6175559	2.2236125
C	-0.9676078	1.4783873	3.7820702
H	-1.5578825	1.7190053	4.6635433
H	-0.8586652	2.3489192	3.1284041
H	-1.4088291	0.6535265	3.2147620
C	-1.1535609	-4.9653429	7.2335213
C	-1.1934521	-4.2561518	6.0301033
C	-0.8745862	-2.8981676	6.0085147
C	-0.5204354	-2.2153773	7.1861942
C	-0.4812008	-2.9417286	8.3897647
C	-0.7951782	-4.3004423	8.4098824
H	-1.3989109	-6.0250800	7.2541432
H	-1.4732772	-4.7606210	5.1074473
H	-0.9057913	-2.3545596	5.0653587
H	-0.2098152	-2.4290726	9.3046168
H	-0.7599820	-4.8447604	9.3516259
C	3.5866814	-3.4064299	10.7323552
C	4.6754346	-3.1122001	9.9087737
C	4.6832808	-1.9436959	9.1454821
C	3.5955979	-1.0572263	9.2036847
C	2.5048238	-1.3529127	10.0397569
C	2.5043085	-2.5255274	10.7940738
H	3.5827656	-4.3189882	11.3244951
H	5.5236593	-3.7911606	9.8594602
H	5.5400468	-1.7301614	8.5152189
H	1.6417837	-0.6954783	10.0803019
H	1.6528290	-2.7528928	11.4314569
C	1.7010857	3.6487849	10.9283143
C	1.2917256	3.5678351	9.5960415
C	1.8446755	2.6110863	8.7470075
C	2.8090712	1.7242876	9.2406261
C	3.2097623	1.7924881	10.5865412
C	2.6599284	2.7619544	11.4234621
H	1.2606522	4.3926001	11.5871939
H	0.5256656	4.2373035	9.2159051
H	1.5229412	2.5524069	7.7147065
H	3.9371650	1.0902527	10.9850789
H	2.9716280	2.8141815	12.4635766
C	8.0033394	1.2427088	7.2331015
C	7.3732375	1.9607011	8.2528544
C	6.0218090	1.7520289	8.5280355
C	5.2974350	0.8165177	7.7792320
C	5.9273639	0.0952600	6.7527041
C	7.2793980	0.3124211	6.4833819
H	9.0566782	1.4104141	7.0204719
H	7.9321145	2.6913701	8.8330401
H	5.5359855	2.3292426	9.3087963
H	5.3721673	-0.6281940	6.1613626
H	7.7641657	-0.2471597	5.6867946

3.4.8.6. int1-*Z*
E(B3LYP-D3/6-31+G*(THF)) = -2469.715934
E(M06-2X-D3/6-311+G**) = -2469.276317

Gtot(B3LYP-D3/6-31+G*(THF)) = -2468.975105

C	-1.1496843	0.7690849	9.8040820
C	-0.7188543	-0.0449648	7.8514639
C	-1.8064800	0.8318998	7.6552317
O	-2.0414309	1.3798432	8.9591940
O	-2.5332664	1.1815239	6.7083321
C	-1.0391756	1.9363613	13.9148097
C	-1.9119113	2.5670044	13.0231506
C	-1.9603466	2.1810777	11.6848176
C	-1.1298098	1.1486292	11.2025948
C	-0.2619684	0.5122799	12.1145992
C	-0.2165980	0.9044130	13.4475733
H	-1.0017174	2.2408621	14.9583827
H	-2.5599890	3.3688129	13.3721823
H	-2.6347970	2.6817664	10.9966058
H	0.3839924	-0.2845734	11.7658300
H	0.4664141	0.4018671	14.1299559
N	-0.3655626	-0.0779410	9.1836255
C	-0.1529726	-0.9760845	6.8182865
H	-0.3745902	-0.5248542	5.8469208
C	1.3939746	-1.1962505	6.9291423
H	1.5900197	-1.7115869	7.8675847
H	1.7047588	-1.8984450	6.1447401
C	2.3313309	0.7641609	5.6643873
C	2.2276570	0.0701147	6.8267632
P	3.2411608	0.5313065	8.3140330
C	2.9518135	2.1289472	5.6140605
O	3.2003368	2.5299083	4.3615817
O	3.1733753	2.8194826	6.5944365
C	3.7446010	3.8655682	4.2201194
H	3.8730010	4.0119050	3.1473994
H	3.0488335	4.6004512	4.6349205
H	4.7027177	3.9360258	4.7418798
C	1.8205339	0.2405513	4.3389131
H	2.6325139	0.2713692	3.6047790
H	1.5046132	-0.8037090	4.4046720
C	0.6570367	1.0150131	3.7281693
O	0.0277215	1.7625829	4.6378817
O	0.3321528	0.9167618	2.5603984
C	-1.1587734	2.4725337	4.2026196
H	-1.6447273	2.7946486	5.1200929
H	-0.8694858	3.3150682	3.5673618
H	-1.8145064	1.7976167	3.6485213
C	-1.9288685	-4.9402080	6.9069133
C	-1.8891823	-4.2161503	5.7135432
C	-1.3245118	-2.9368028	5.6873021
C	-0.8010883	-2.3604854	6.8500113
C	-0.8497100	-3.0950472	8.0446310
C	-1.4057595	-4.3735223	8.0743231
H	-2.3678597	-5.9354537	6.9304093
H	-2.2997842	-4.6454982	4.8017158
H	-1.3000572	-2.3775061	4.7530724
H	-0.4643899	-2.6449612	8.9560237
H	-1.4377086	-4.9278879	9.0106059
C	3.8271964	-3.0714964	11.1612106
C	4.9006733	-2.6263860	10.3876541
C	4.7356058	-1.5617518	9.4991230
C	3.4834079	-0.9371034	9.3816675
C	2.4028291	-1.3863579	10.1628844
C	2.5791511	-2.4516445	11.0438542
H	3.9626633	-3.8987129	11.8547785
H	5.8743175	-3.1022677	10.4775690
H	5.5864988	-1.2223237	8.9191553
H	1.4259992	-0.9184696	10.0504991
H	1.7391446	-2.7983631	11.6417585
C	1.4014115	3.7257483	11.0510847
C	0.8711940	3.5284568	9.7738215
C	1.4136551	2.5642094	8.9289578
C	2.4930597	1.7910607	9.3731086
C	3.0155566	1.9698033	10.6651614
C	2.4711370	2.9470156	11.4972074
H	0.9634159	4.4709132	11.7098833
H	0.0163551	4.1111469	9.4413892
H	0.9823297	2.3889368	7.9497373
H	3.8309142	1.3484049	11.0274375
H	2.8723873	3.0855775	12.4980459
C	7.5351951	1.5559220	6.9720370
C	6.9184853	2.3222526	7.9640167
C	5.6105639	2.0403109	8.3596209
C	4.9180616	0.9805153	7.7607708
C	5.5359605	0.2086668	6.7625787
C	6.8428838	0.5012423	6.3709733
H	8.5543034	1.7807542	6.6661847
H	7.4527404	3.1475059	8.4288820
H	5.1323890	2.6537142	9.1171156
H	5.0102138	-0.6191671	6.2925935
H	7.3189709	-0.0987497	5.5991118

3.4.8.7. int1-*E*
E(B3LYP-D3/6-31+G*(THF)) = -2469.715699
E(M06-2X-D3/6-311+G**) = -2469.272376
Gtot(B3LYP-D3/6-31+G*(THF)) = -2468.977331

C	-1.4294129	1.2026768	9.5092968
C	-0.7599363	0.1392168	7.7547120
C	-1.7054966	1.0715468	7.2810382
O	-2.1222719	1.7663796	8.4670653
O	-2.1887430	1.3875343	6.1805979
C	-1.9829916	2.7065008	13.4760196
C	-2.6808217	3.2693761	12.4023601
C	-2.5087594	2.7820143	11.1071654
C	-1.6282534	1.7107942	10.8524438
C	-0.9370427	1.1444540	11.9444809
C	-1.1102872	1.6382041	13.2329838
H	-2.1203420	3.0880296	14.4853310
H	-3.3678140	4.0959233	12.5748320
H	-3.0518522	3.2280419	10.2790787
H	-0.2636108	0.3125454	11.7675540
H	-0.5641437	1.1841266	14.0579522
N	-0.6229676	0.2438553	9.1229317
C	-0.0769333	-0.8992505	6.9150596
H	-0.3234671	-0.6490819	5.8778759
C	1.4806692	-0.9109753	7.0570374
H	1.7173177	-1.2598240	8.0612341
H	1.8957710	-1.6538582	6.3715698
C	2.1099042	1.0792925	5.6034695
C	2.1427012	0.4318487	6.7930586
P	3.0128322	1.1932220	8.2341417
C	1.4520368	0.4863879	4.3718760

O	1.7206244	-0.8107939	4.1904733
O	0.7809750	1.1589348	3.6121602
C	1.1272641	-1.4225683	3.0188627
H	1.4768966	-2.4556651	3.0258854
H	0.0363382	-1.3789096	3.0827069
H	1.4605813	-0.9059853	2.1148184
C	2.6132231	2.4764043	5.3064219
H	1.8138921	3.0216404	4.7857015
H	2.8593684	3.0592974	6.1921655
C	3.8152878	2.4489134	4.3704538
O	4.4708907	3.6145425	4.4091962
O	4.1269985	1.5036872	3.6707307
C	5.6205171	3.7305723	3.5362956
H	6.0140179	4.7333105	3.7077890
H	6.3645199	2.9739359	3.7974533
H	5.3185050	3.6067340	2.4927217
C	-1.2602331	-5.0210717	7.6166344
C	-1.2360468	-4.5057735	6.3186914
C	-0.8775328	-3.1722265	6.1001236
C	-0.5452265	-2.3318074	7.1691668
C	-0.5775443	-2.8586775	8.4696288
C	-0.9308544	-4.1898665	8.6922256
H	-1.5350367	-6.0595106	7.7900737
H	-1.4948915	-5.1422444	5.4746528
H	-0.8587277	-2.7793991	5.0849444
H	-0.3372394	-2.2092229	9.3063471
H	-0.9507532	-4.5807513	9.7080678
C	7.1524733	2.7359908	6.9214531
C	6.3718114	3.5057123	7.7875842
C	5.1042402	3.0644562	8.1690336
C	4.6147631	1.8463965	7.6737988
C	5.3953357	1.0767066	6.7934641
C	6.6638001	1.5238158	6.4245517
H	8.1418486	3.0819461	6.6311656
H	6.7476744	4.4519245	8.1697116
H	4.5050176	3.6699082	8.8427242
H	5.0196302	0.1387201	6.3928896
H	7.2673440	0.9238550	5.7478667
C	3.9010123	-1.8117680	11.6224462
C	4.9435091	-1.3696456	10.8053650
C	4.6888103	-0.4778535	9.7618982
C	3.3779049	-0.0277928	9.5367919
C	2.3262680	-0.4715924	10.3603239
C	2.5952934	-1.3644212	11.3963434
H	4.1058780	-2.5041229	12.4363098
H	5.9602572	-1.7132167	10.9808263
H	5.5123591	-0.1359966	9.1439195
H	1.3062296	-0.1409247	10.1692211
H	1.7835283	-1.7132305	12.0308820
C	0.5349814	4.5218864	10.2137164
C	1.5923338	3.9243264	10.9047876
C	2.3348723	2.9107111	10.3027286
C	2.0191344	2.5001230	8.9943231
C	0.9534093	3.0952532	8.3051148
C	0.2164486	4.1060569	8.9196080
H	-0.0569910	5.2957402	10.6953495
H	1.8293863	4.2344177	11.9194134
H	3.1411504	2.4348512	10.8549700
H	0.6560811	2.7348109	7.3268621
H	-0.6293129	4.5426114	8.3960200

3.4.9. Ammonium ylide

3.4.9.1. NMe_3

E(B3LYP-D3/6-31+G*(THF)) = -174.484509
E(M06-2X-D3/6-311+G**) = -174.428090
Gtot(B3LYP-D3/6-31+G*(THF)) = -174.390197

N	1.9802366	1.0542782	7.0318270
C	0.9517227	1.3466167	8.0279765
H	1.3744555	1.6213712	9.0160636
H	0.3238481	2.1785242	7.6871805
H	0.3076982	0.4705893	8.1685855
C	2.8223106	2.2262527	6.7975563
H	3.3698310	2.5520050	7.7055208
H	3.5621356	2.0073914	6.0183152
H	2.2074591	3.0671047	6.4553582
C	2.7903150	-0.0891502	7.4474652
H	3.3304089	0.0891800	8.3996465
H	2.1540666	-0.9721987	7.5816923
H	3.5343550	-0.3204475	6.6761141

3.4.9.2. TS ylide formation

E(B3LYP-D3/6-31+G*(THF)) = -786.231418
E(M06-2X-D3/6-311+G**) = -786.072554
Gtot(B3LYP-D3/6-31+G*(THF)) = -785.979281

C	-0.8095333	0.0670536	6.0045974
C	-1.6120477	1.2370108	6.1979249
O	-2.8969316	0.9547804	6.6062529
O	-1.2531671	2.4142020	6.0261059
C	-1.3461091	-1.3395120	6.2326135
H	-2.2679278	-1.2816877	6.8236424
H	-1.6082116	-1.8463507	5.2944712
C	-0.4128572	-2.2545592	7.0099540
O	-0.3524803	-3.4819941	6.4543465
O	0.1984548	-1.9639641	8.0252277
C	0.4695959	-4.4536072	7.1382613
H	0.4230201	-5.3581867	6.5295576
H	1.5003698	-4.0965086	7.2145867
H	0.0799457	-4.6468093	8.1420009
C	-3.7409320	2.0902453	6.8363458
H	-4.7051716	1.6827173	7.1501483
H	-3.3322998	2.7346487	7.6225230
H	-3.8628721	2.6843928	5.9244904
H	2.2499092	0.0063584	4.3239039
C	1.1697660	-0.0578386	4.4130321
H	0.6326205	-0.3853772	3.5238434
C	0.4739802	0.2263040	5.5025829
N	1.7377800	1.0103159	6.7848748
C	1.0017647	1.2733329	8.0393109
H	1.7053820	1.5924724	8.8190264
H	0.2673599	2.0576531	7.8627403
H	0.4950429	0.3569030	8.3448252
C	2.2338968	2.2712520	6.1968277
H	2.9005302	2.7881188	6.9013851
H	2.7845560	2.0579650	5.2778665
H	1.3716709	2.8981849	5.9620130
C	2.8309827	0.0466755	7.0408182
H	3.5377618	0.4567339	7.7759326

H	2.3964817	-0.8765593	7.4281320
H	3.3724156	-0.1633750	6.1163378

3.4.9.3. Ylide *Z*
E(B3LYP-D3/6-31+G*(THF)) = -786.239292
E(M06-2X-D3/6-311+G**) = -786.077271
Gtot(B3LYP-D3/6-31+G*(THF)) = -785.984044

C	-0.8436867	0.4866610	6.1750629
C	-1.7757080	1.4914436	5.8361812
O	-3.0636041	1.1603764	6.2412707
O	-1.5880556	2.5873736	5.2541217
C	-1.3644223	-0.7894333	6.8076147
H	-0.5213496	-1.4024481	7.1559375
H	-1.9784091	-0.5805166	7.6916690
C	-2.2276196	-1.7280031	5.9693654
O	-1.9955445	-1.6366332	4.6467691
O	-3.0237076	-2.5228199	6.4453772
C	-2.7655412	-2.5235429	3.8089897
H	-2.4808642	-2.2812492	2.7837433
H	-2.5306461	-3.5687497	4.0338218
H	-3.8366676	-2.3583934	3.9577034
C	-4.0827988	2.1063030	5.9174280
H	-5.0156045	1.6784527	6.2943008
H	-3.8985188	3.0756587	6.3948933
H	-4.1577029	2.2630268	4.8353758
H	2.2511494	-0.6354683	5.0500442
C	1.1796782	-0.5473217	5.1821804
H	0.5822930	-1.3908902	4.8541084
C	0.5357303	0.4948120	5.7475782
N	1.4141752	1.7718133	6.0230928
C	0.8483749	2.5995084	7.1528280
H	1.5449060	3.4189786	7.3466517
H	-0.1165201	2.9936171	6.8470171
H	0.7492771	1.9658722	8.0344277
C	1.4631973	2.6071665	4.7644949
H	2.0633474	3.5017444	4.9563986
H	1.9174497	2.0046277	3.9766750
H	0.4356227	2.8651056	4.5102910
C	2.8261265	1.4046095	6.4139059
H	3.3501346	2.3207276	6.6956091
H	2.7934931	0.7124677	7.2571007
H	3.3422254	0.9515069	5.5690274

3.4.9.4. Addition TS *Z*
E(B3LYP-D3/6-31+G*(THF)) = -1607.817374
E(M06-2X-D3/6-311+G**) = -1607.497359
Gtot(B3LYP-D3/6-31+G*(THF)) = -1607.347391

C	-0.9431789	1.2673388	7.6441130
C	-0.5442780	-0.6156224	6.6779355
C	-1.2720106	0.1760583	5.7331219
O	-1.5344393	1.3887783	6.4065014
O	-1.6388107	0.0046374	4.5701849
C	-1.1191668	4.5575245	10.3504672
C	-1.8037025	4.6192959	9.1334112
C	-1.7564310	3.5482620	8.2413328
C	-1.0198524	2.3940399	8.5610668
C	-0.3332153	2.3369685	9.7895624
C	-0.3833391	3.4107054	10.6734036
H	-1.1578509	5.3949938	11.0434149
H	-2.3790541	5.5061721	8.8770628
H	-2.2878050	3.5991302	7.2956631
H	0.2388818	1.4457815	10.0300963
H	0.1533676	3.3556044	11.6181817
N	-0.3503263	0.1261080	7.8415598
C	-0.0714370	-1.9194418	6.3842535
H	-0.5367296	-2.3155129	5.4823148
C	1.7435554	-1.7542112	5.3492633
H	2.0915486	-2.7776644	5.3355045
H	1.2415955	-1.4934334	4.4271987
C	2.2421989	0.6200979	5.6397455
C	2.5092803	-0.7217967	5.9396974
N	3.5413240	-1.1519518	6.9913988
C	3.8475847	-2.6353863	6.9273468
H	4.6279749	-2.8365714	7.6644372
H	2.9558793	-3.2073693	7.1788237
H	4.2082592	-2.8895891	5.9296414
C	4.8532357	-0.4458637	6.6891358
H	5.6213824	-0.8608705	7.3459280
H	5.0957725	-0.6304419	5.6414566
H	4.7255089	0.6152516	6.8775201
C	3.0853010	-0.8760076	8.4130180
H	2.2285353	-1.5137322	8.6153257
H	3.9125374	-1.1245009	9.0833898
H	2.8255531	0.1716041	8.5064608
C	2.4518381	1.7830325	6.5082408
O	1.6480872	2.8180500	6.1543532
O	3.2029227	1.8921391	7.4825211
C	1.7481278	4.0030240	6.9667257
H	1.0292772	4.7050296	6.5415619
H	1.4889407	3.7760054	8.0039630
H	2.7610287	4.4155023	6.9229255
C	1.6389237	0.9034216	4.2681733
H	0.6285980	0.5133940	4.1101208
H	1.5441385	1.9844220	4.1298579
C	2.5267229	0.4158338	3.1275249
O	1.7973293	0.2689469	2.0027797
O	3.7254560	0.2097822	3.1754737
C	2.5205955	-0.1270682	0.8171947
H	1.7764214	-0.1710626	0.0198660
H	3.2956873	0.6062296	0.5763141
H	2.9832962	-1.1074780	0.9638708
C	0.5839652	-5.0669687	9.2641536
C	0.5975576	-5.3292842	7.8899444
C	0.3840356	-4.2972495	6.9771325
C	0.1603968	-2.9741149	7.4089965
C	0.1286722	-2.7302054	8.7954756
C	0.3419646	-3.7655649	9.7087616
H	0.7502120	-5.8706659	9.9783168
H	0.7680297	-6.3416104	7.5295988
H	0.3851459	-4.5161611	5.9102098
H	-0.0678493	-1.7236681	9.1480948
H	0.3125504	-3.5509749	10.7754206

3.4.9.5. Addition TS *E*
E(B3LYP-D3/6-31+G*(THF)) = -1607.800698
E(M06-2X-D3/6-311+G**) = -1607.475556
Gtot(B3LYP-D3/6-31+G*(THF)) = -1607.329862

C	-0.8085338	0.3759635	10.7770763
C	-0.3667779	0.1688593	8.6793947
C	-0.6175817	1.5662293	8.9017926
O	-0.9083241	1.6606604	10.2821237
O	-0.6211318	2.5710995	8.1911237
C	-1.5785937	-0.3473601	14.9077460
C	-1.7172766	0.9443364	14.3919514
C	-1.4650121	1.1968048	13.0431419
C	-1.0695183	0.1503400	12.1921289
C	-0.9320435	-1.1496463	12.7173854
C	-1.1846946	-1.3931292	14.0634904
H	-1.7771795	-0.5407875	15.9595154
H	-2.0261349	1.7592616	15.0426484
H	-1.5780563	2.2003182	12.6435008
H	-0.6288947	-1.9552868	12.0551060
H	-1.0763565	-2.4012046	14.4579178
N	-0.4939614	-0.5149374	9.8862136
C	-0.1120482	-0.4109198	7.4189094
H	-0.2337502	0.2725447	6.5827750
C	2.0730575	-0.6548946	7.0185666
H	2.2865413	-1.0035974	8.0166767
H	1.8991104	-1.4412833	6.3037679
C	2.7983096	0.8782666	5.1598404
C	2.7014552	0.4974334	6.5082346
N	3.2927123	1.4439238	7.5908452
C	3.0410635	0.9570629	9.0079149
H	3.4118105	1.7295708	9.6837674
H	1.9739592	0.8110028	9.1597639
H	3.5893927	0.0316011	9.1825075
C	4.8044890	1.5162161	7.4699775
H	5.1816921	2.1257616	8.2944599
H	5.1913485	0.4979340	7.5378797
H	5.1048156	1.9486052	6.5245171
C	2.6411144	2.8185795	7.5250378
H	3.1051680	3.4499724	8.2864238
H	2.7737589	3.2713010	6.5481605
H	1.5755344	2.6953242	7.7231013
C	2.2394430	0.0848575	4.0517901
O	1.3612972	-0.8999214	4.3628900
O	2.5315164	0.3109286	2.8736886
C	0.8955628	-1.6844256	3.2472108
H	0.2703130	-2.4606762	3.6867661
H	0.3133438	-1.0655239	2.5575254
H	1.7382439	-2.1271557	2.7079188
C	3.6250001	1.9990223	4.5435134
H	3.0454051	2.5184372	3.7765921
H	3.9525709	2.7677819	5.2424045
C	4.8948842	1.4558272	3.8871171
O	5.1918042	2.1177541	2.7568423
O	5.5871702	0.5667399	4.3489187
C	6.3706878	1.6653720	2.0530654
H	6.4417424	2.2938934	1.1640346
H	7.2606249	1.7810477	2.6784100
H	6.2598594	0.6144411	1.7714173
C	-1.2690424	-4.4564166	6.5338682
C	-1.5926128	-3.4102998	5.6658112
C	-1.1906186	-2.1065921	5.9600080
C	-0.4749899	-1.8185899	7.1345461
C	-0.1597831	-2.8790121	8.0033348
C	-0.5485626	-4.1835124	7.7011219
H	-1.5752203	-5.4747803	6.3039801
H	-2.1582462	-3.6087451	4.7576804
H	-1.4404451	-1.2975674	5.2774190
H	0.3885214	-2.6739134	8.9158563
H	-0.2903925	-4.9920599	8.3821009

3.4.9.6. int1-*Z*

E(B3LYP-D3/6-31+G*(THF)) = -1607.829139
E(M06-2X-D3/6-311+G**) = -1607.505498
Gtot(B3LYP-D3/6-31+G*(THF)) = -1607.355819

C	-0.8962161	0.6684366	9.8778794
C	-0.5979867	-0.1385449	7.8981293
C	-1.8164502	0.5698651	7.8300245
O	-1.9714748	1.1077914	9.1505033
O	-2.6840924	0.7982771	6.9710859
C	-0.4676947	1.8404664	13.9643294
C	-1.5497876	2.3020712	13.2082492
C	-1.6984094	1.9239674	11.8747437
C	-0.7599588	1.0669525	11.2631145
C	0.3261959	0.6024577	12.0370935
C	0.4682459	0.9865336	13.3662751
H	-0.3552043	2.1377876	15.0043940
H	-2.2855981	2.9636961	13.6611458
H	-2.5412969	2.2878481	11.2944603
H	1.0482636	-0.0682678	11.5807389
H	1.3123669	0.6152288	13.9445615
N	-0.0776563	-0.0784682	9.1704099
C	-0.0287338	-0.9826541	6.7969425
H	-0.3102568	-0.4984101	5.8603073
C	1.5315273	-1.1313469	6.8477874
H	1.7605666	-1.7079646	7.7359172
H	1.8426825	-1.7730241	6.0157937
C	2.3496050	0.9202913	5.6602375
C	2.3338859	0.1604987	6.7922229
N	3.1505059	0.5045027	8.0525328
C	3.1391655	-0.5992783	9.1045122
H	3.7610521	-0.2443421	9.9285396
H	2.1143551	-0.7491192	9.4432844
H	3.5784831	-1.5074856	8.6895672
C	4.6190569	0.6706075	7.6830446
H	5.1779163	0.8348662	8.6068065
H	4.9506533	-0.2512704	7.2007673
H	4.7389399	1.5233781	7.0270807
C	2.5950858	1.7370980	8.7620110
H	1.6059000	1.4702974	9.1294471
H	3.2713600	1.9708470	9.5881404
H	2.5595828	2.5679561	8.0691548
C	2.9102349	2.3108444	5.4723745
O	2.6439452	2.7636098	4.2401281
O	3.5013227	2.9997658	6.2913141
C	3.0579982	4.1199094	3.9458880
H	2.7209177	4.3052525	2.9255942
H	2.5879845	4.8167624	4.6453017
H	4.1458812	4.2039740	4.0180833
C	1.7412321	0.3712122	4.3716214
H	2.4754169	0.4719819	3.5668716
H	1.5220694	-0.6955841	4.4365280
C	0.4673325	1.0399936	3.8625941
O	-0.1702159	1.7202046	4.8178423

O	0.0701561	0.9094313	2.7198939
C	-1.4495579	2.3038830	4.4650857
H	-1.9145467	2.5634410	5.4131929
H	-1.2900655	3.1807241	3.8304261
H	-2.0641705	1.5709706	3.9384183
C	-1.6509759	-5.0113089	6.7085639
C	-1.6140963	-4.2432669	5.5428619
C	-1.0940713	-2.9456150	5.5747249
C	-0.6135918	-2.3959180	6.7687379
C	-0.6599132	-3.1734065	7.9354363
C	-1.1705429	-4.4712148	7.9066542
H	-2.0574923	-6.0202400	6.6873397
H	-1.9945342	-4.6511012	4.6083968
H	-1.0746004	-2.3501490	4.6628567
H	-0.3129496	-2.7438216	8.8726620
H	-1.2029670	-5.0601291	8.8213817

3.4.9.7. int1-*E*
E(B3LYP-D3/6-31+G*(THF)) = -1607.827572
E(M06-2X-D3/6-311+G**) = -1607.504996
Gtot(B3LYP-D3/6-31+G*(THF)) = -1607.353911

C	-0.8589493	0.3186165	10.7113960
C	-0.4172310	0.2918003	8.6074142
C	-1.1460780	1.4751553	8.8108551
O	-1.4243828	1.4722326	10.2178782
O	-1.5152713	2.4385223	8.1057704
C	-1.2435773	-0.7344208	14.8335580
C	-1.8297689	0.4419792	14.3574711
C	-1.7079243	0.8045703	13.0150709
C	-0.9930626	-0.0121924	12.1189291
C	-0.4041877	-1.1983313	12.6084395
C	-0.5291513	-1.5518605	13.9473505
H	-1.3425121	-1.0147964	15.8799048
H	-2.3895184	1.0818996	15.0366214
H	-2.1705537	1.7174255	12.6507955
H	0.1417216	-1.8317987	11.9153182
H	-0.0705624	-2.4721955	14.3046336
N	-0.2590610	-0.3936688	9.7952251
C	0.0340664	-0.2876056	7.2986054
H	-0.2632052	0.4124101	6.5122950
C	1.5982652	-0.4850963	7.1925776
H	1.9957293	-0.7649727	8.1683718
H	1.7945402	-1.3416651	6.5491290
C	2.5394627	0.8161940	5.2651095
C	2.3266432	0.7002513	6.5945123
N	2.7484396	1.8135751	7.5697085
C	2.6856353	1.3640086	9.0239656
H	2.9526173	2.2265383	9.6371335
H	1.6759293	1.0391206	9.2613225
H	3.4109540	0.5631594	9.1794382
C	4.1923229	2.2541325	7.3671190
H	4.4900946	2.8210564	8.2502962
H	4.8081404	1.3643779	7.2471727
H	4.2842873	2.8908425	6.4943599
C	1.8001665	2.9959006	7.4191697
H	2.1044529	3.7644054	8.1344192
H	1.8658309	3.3874203	6.4051531
H	0.7788400	2.6707690	7.6251792
C	2.1842848	-0.3218365	4.3265979
O	0.9340728	-0.7689885	4.4374612
O	2.9823008	-0.7224880	3.4956165
C	0.5538848	-1.8538032	3.5495109
H	-0.4479368	-2.1353540	3.8692417
H	0.5564025	-1.5065094	2.5123393
H	1.2474358	-2.6910648	3.6606037
C	3.1838158	1.9124896	4.4312318
H	2.5854129	2.0688866	3.5282449
H	3.2243260	2.8809621	4.9312710
C	4.6085226	1.5362101	4.0139400
O	4.8237412	1.8200364	2.7238558
O	5.4472683	1.0726592	4.7623830
C	6.1375321	1.4883933	2.2105396
H	6.1080787	1.7390422	1.1492364
H	6.9056195	2.0734341	2.7239777
H	6.3346898	0.4219148	2.3484677
C	-2.1111603	-4.0019902	6.5926650
C	-2.5216328	-2.8157515	5.9780798
C	-1.8146502	-1.6306963	6.1986879
C	-0.6901707	-1.6066174	7.0345023
C	-0.2943802	-2.8000695	7.6547479
C	-0.9952859	-3.9873259	7.4335672
H	-2.6583775	-4.9269432	6.4220484
H	-3.3933245	-2.8119711	5.3262870
H	-2.1423126	-0.7101465	5.7183289
H	0.5544277	-2.7990332	8.3328806
H	-0.6719256	-4.9028120	7.9254986

4. Copies of NMR Spectra

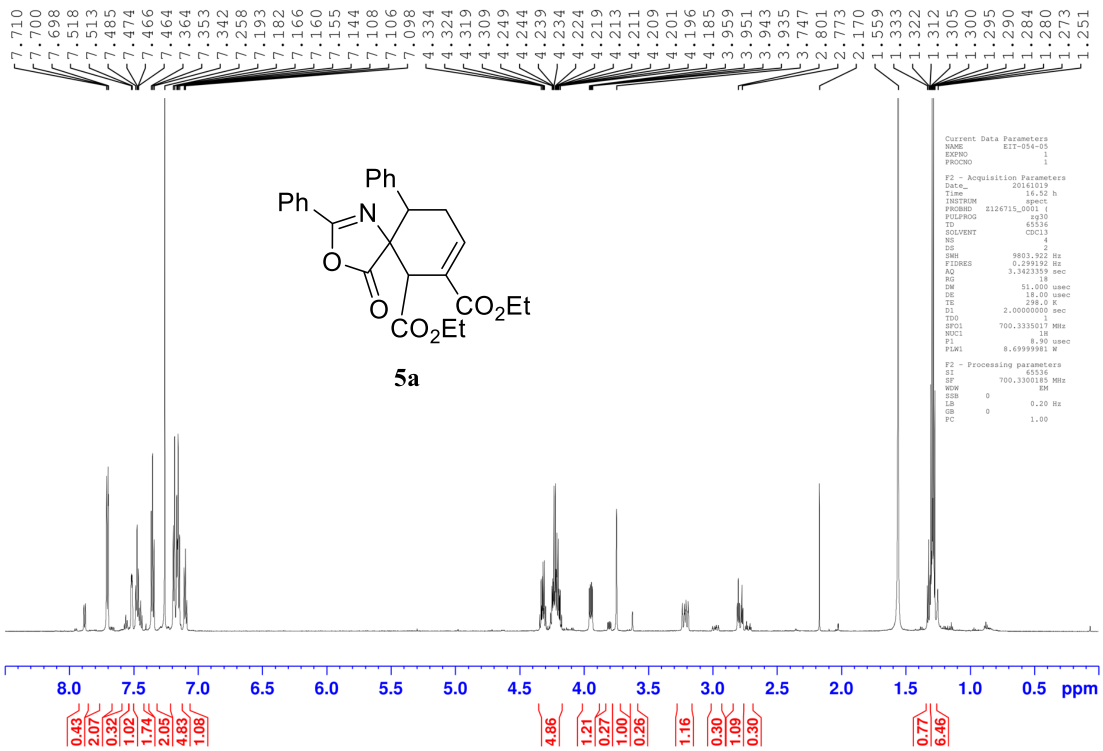

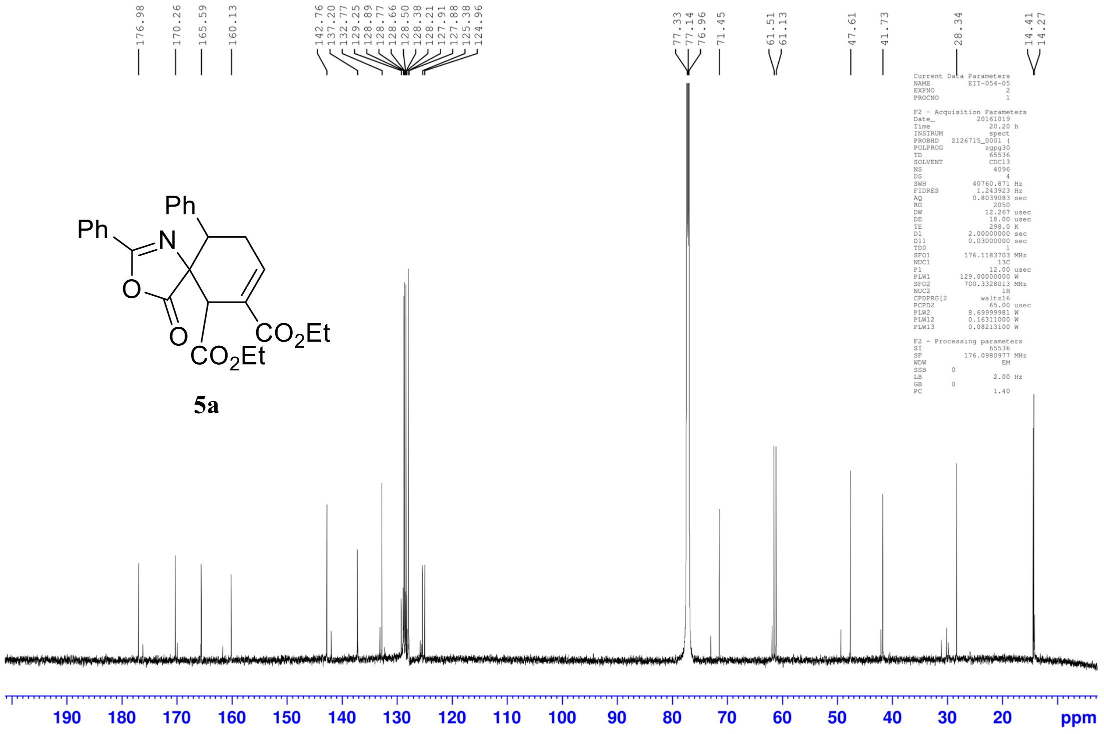

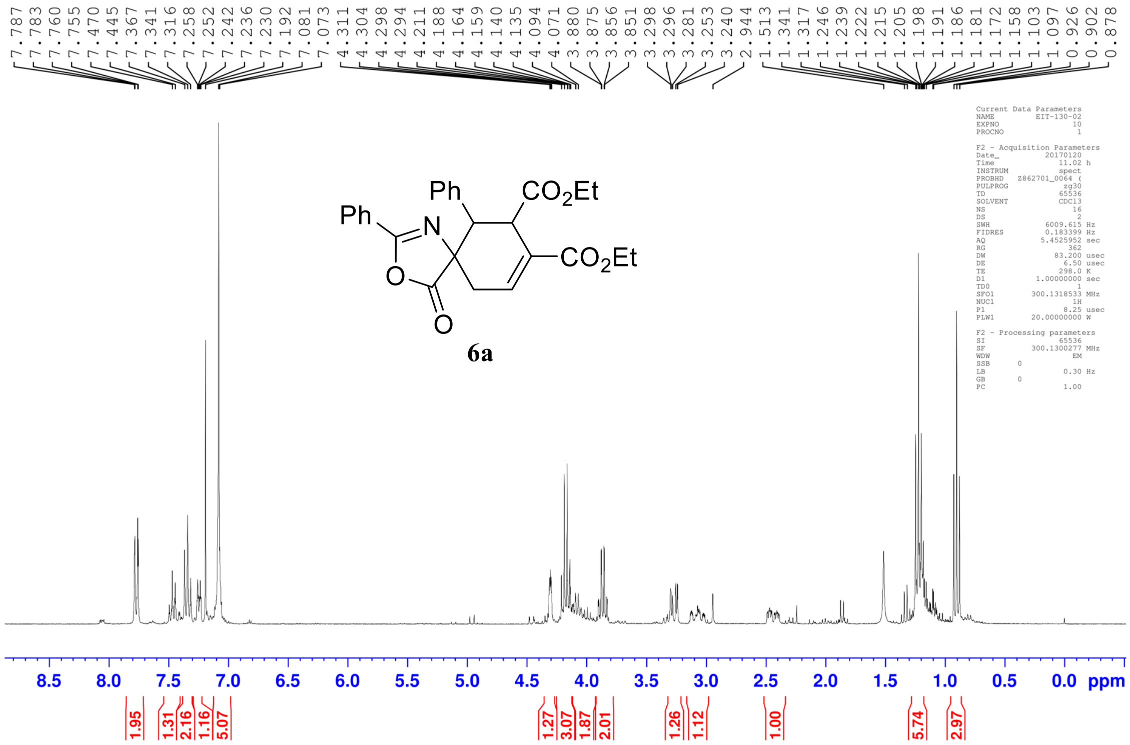

6a

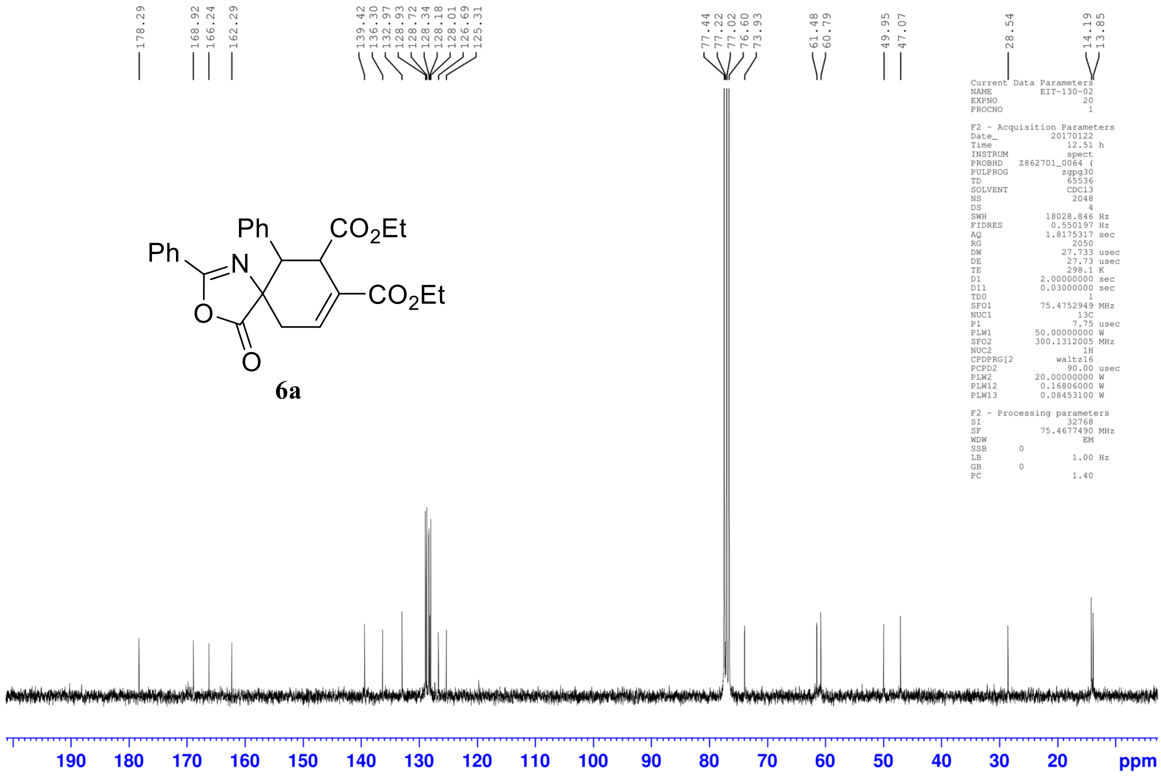

6a

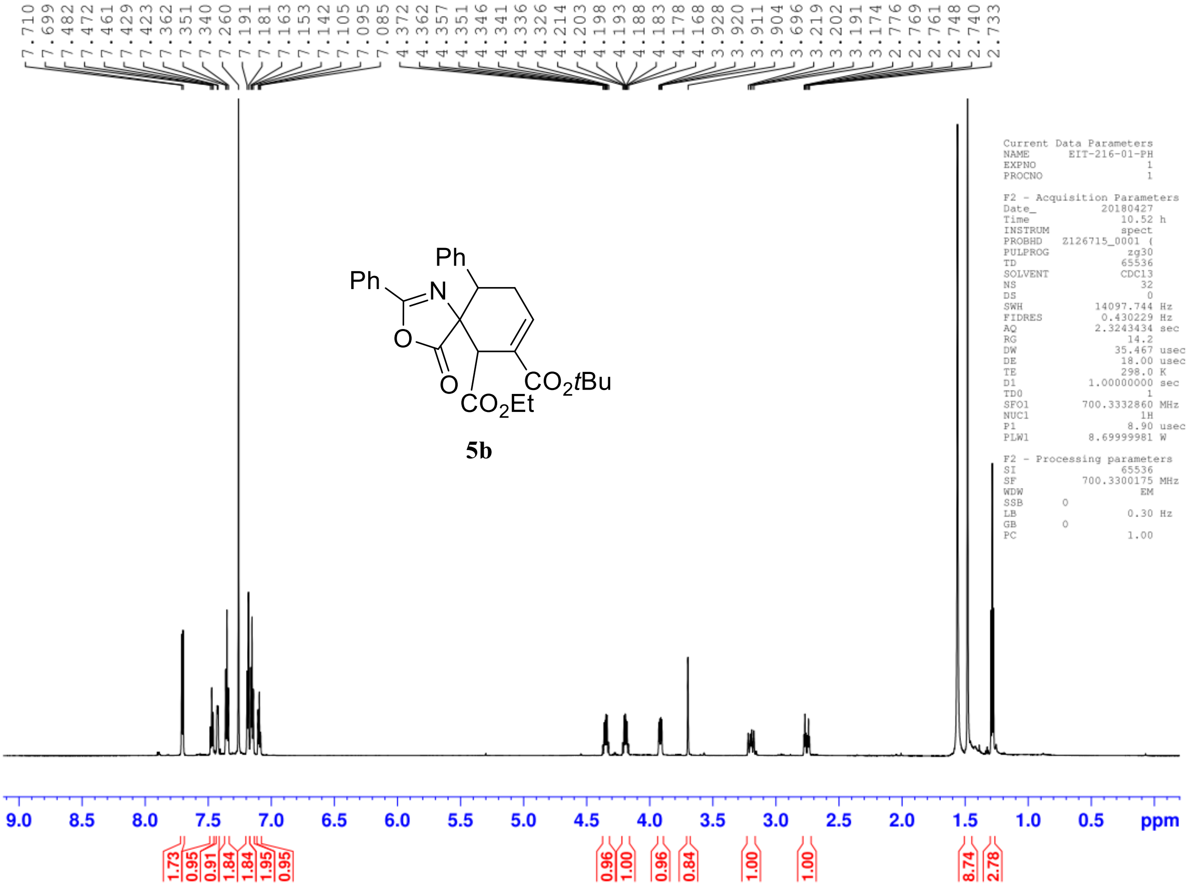
7.710 7.699 7.482 7.472 7.461 7.429 7.423 7.362 7.351 7.340 7.260 7.191 7.181 7.163 7.153 7.142 7.105 7.095 7.085 4.372 4.362 4.357 4.351 4.346 4.341 4.336 4.326 4.214 4.203 4.198 4.193 4.188 4.183 4.178 4.168 3.928 3.920 3.911 3.904 3.696 3.219 3.202 3.191 3.174 2.776 2.769 2.761 2.748 2.740 2.733
Ph
Ph
N
O
O
CO_2tBu
CO_2Et
5b
Current Data Parameters
NAME EIT-216-01-PH
EXPNO 1
PROCNO 1
F2 - Acquisition Parameters
Date_ 20180427
Time 10.52 h
INSTRUM spect
PROBHD Z126715_0001 (
PULPROG zg30
TD 65536
SOLVENT CDCl3
NS 32
DS 0
SWH 14097.744 Hz
FIDRES 0.430229 Hz
AQ 2.3243434 sec
RG 14.2
DW 35.467 usec
DE 18.00 usec
TE 298.0 K
D1 1.00000000 sec
TD0 1
SFO1 700.3332860 MHz
NUC1 1H
P1 8.90 usec
PLW1 8.69999981 W
F2 - Processing parameters
SI 65536
SF 700.3300175 MHz
WDW EM
SSB 0
LB 0.30 Hz
GB 0
PC 1.00
9.0 8.5 8.0 7.5 7.0 6.5 6.0 5.5 5.0 4.5 4.0 3.5 3.0 2.5 2.0 1.5 1.0 0.5 ppm
1.73 0.95 0.91 1.84 1.84 1.95 0.95 0.96 1.00 0.96 0.84 1.00 1.00 8.74 2.78

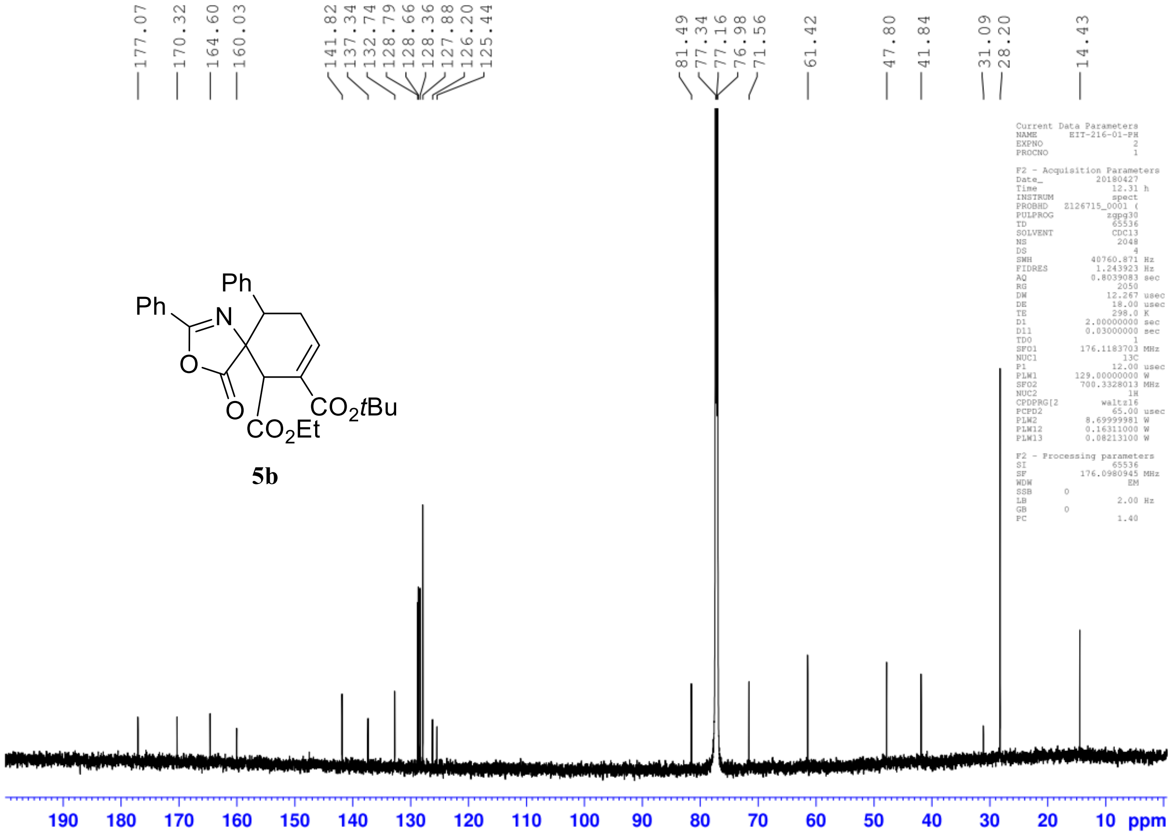
177.07 170.32 164.60 160.03 141.82 137.34 132.74 128.79 128.66 128.36 127.88 126.20 125.44 81.49 77.34 77.16 76.98 71.56 61.42 47.80 41.84 31.09 28.20 14.43
Ph
Ph
N
O
O
CO_2tBu
CO_2Et
5b
190 180 170 160 150 140 130 120 110 100 90 80 70 60 50 40 30 20 10 ppm

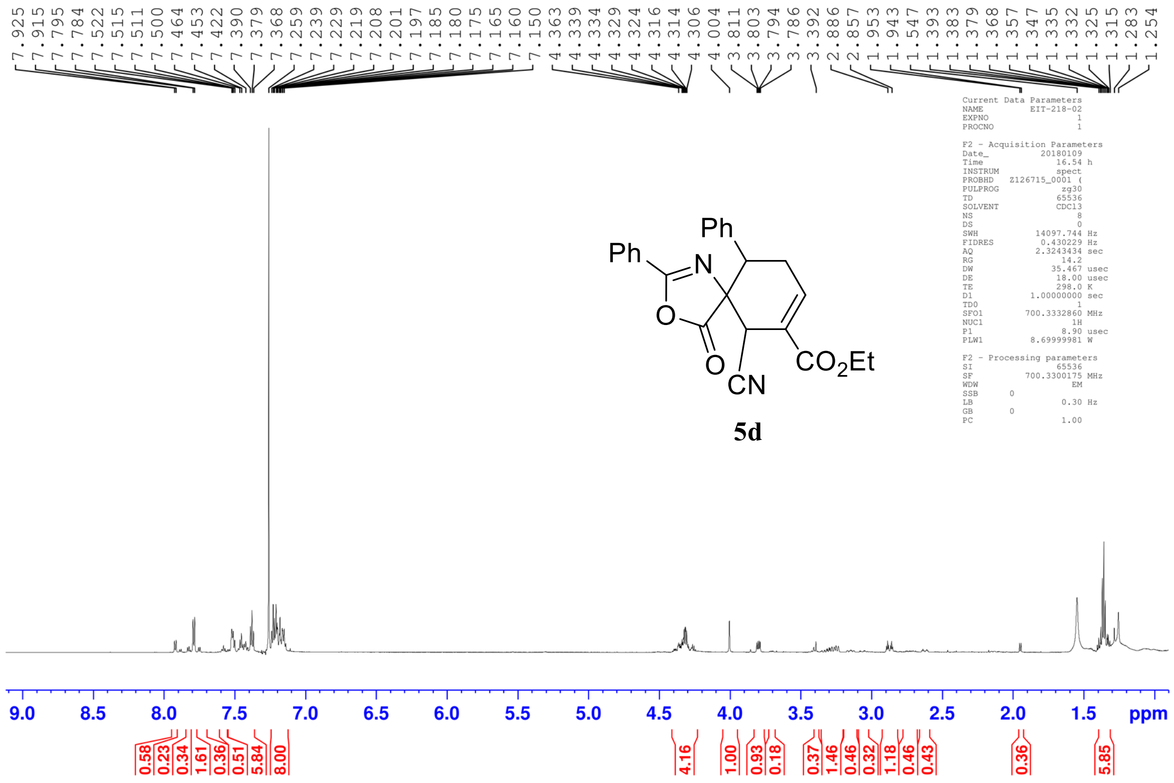

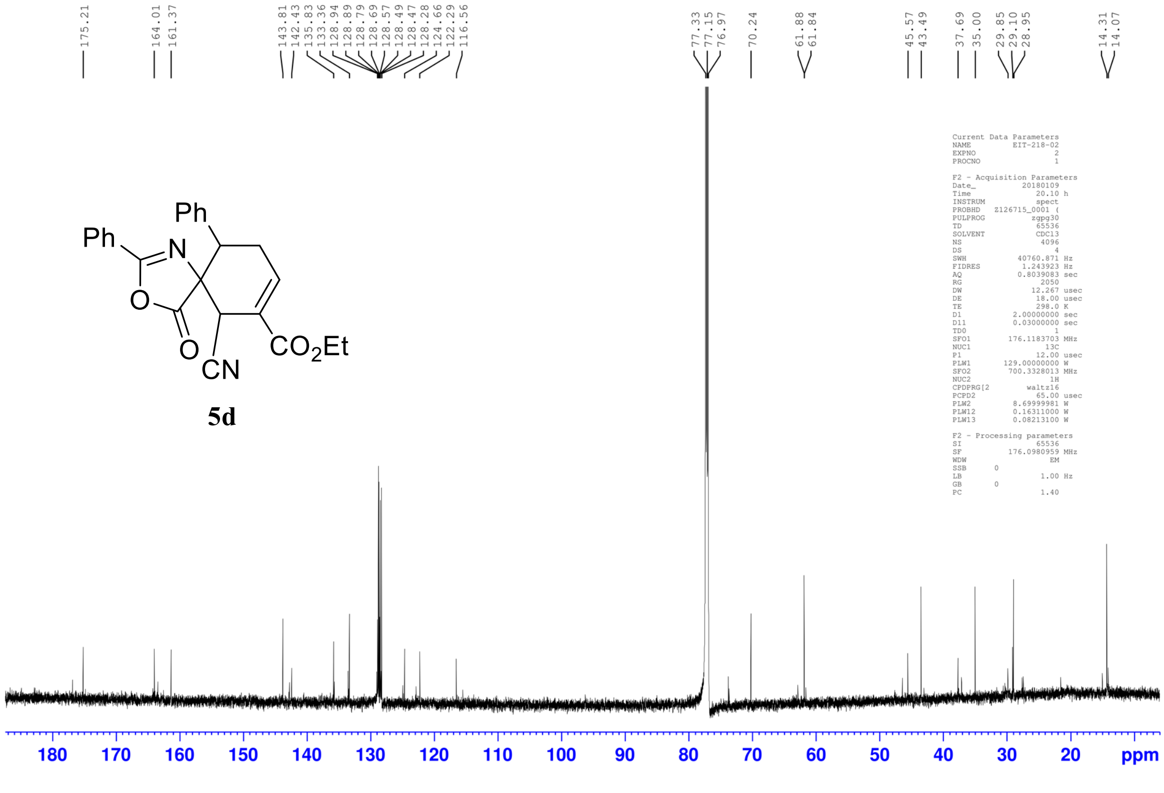

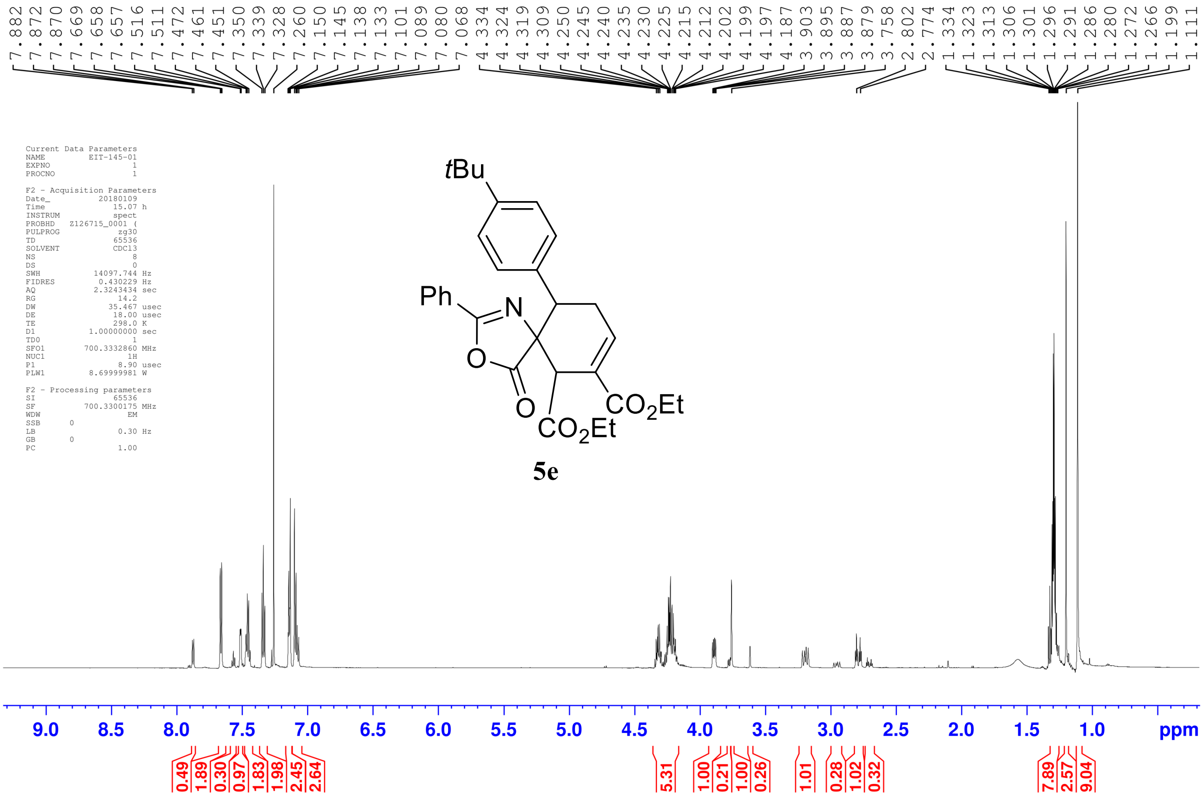

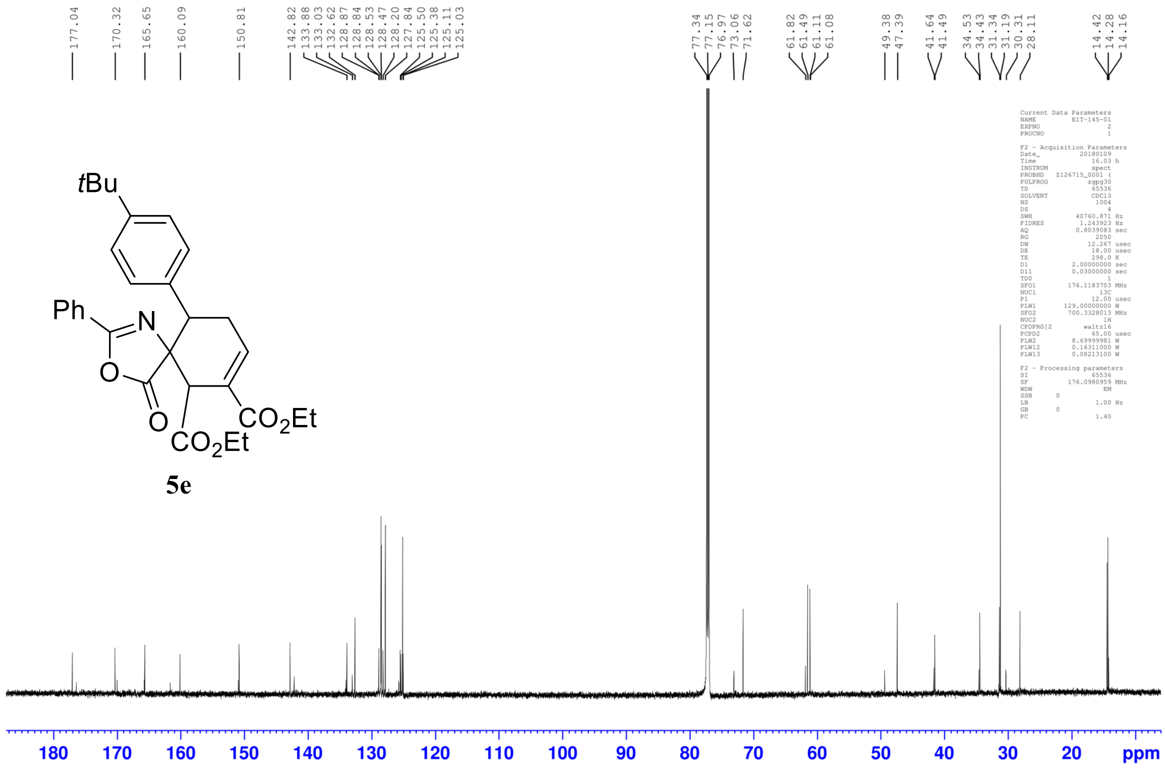

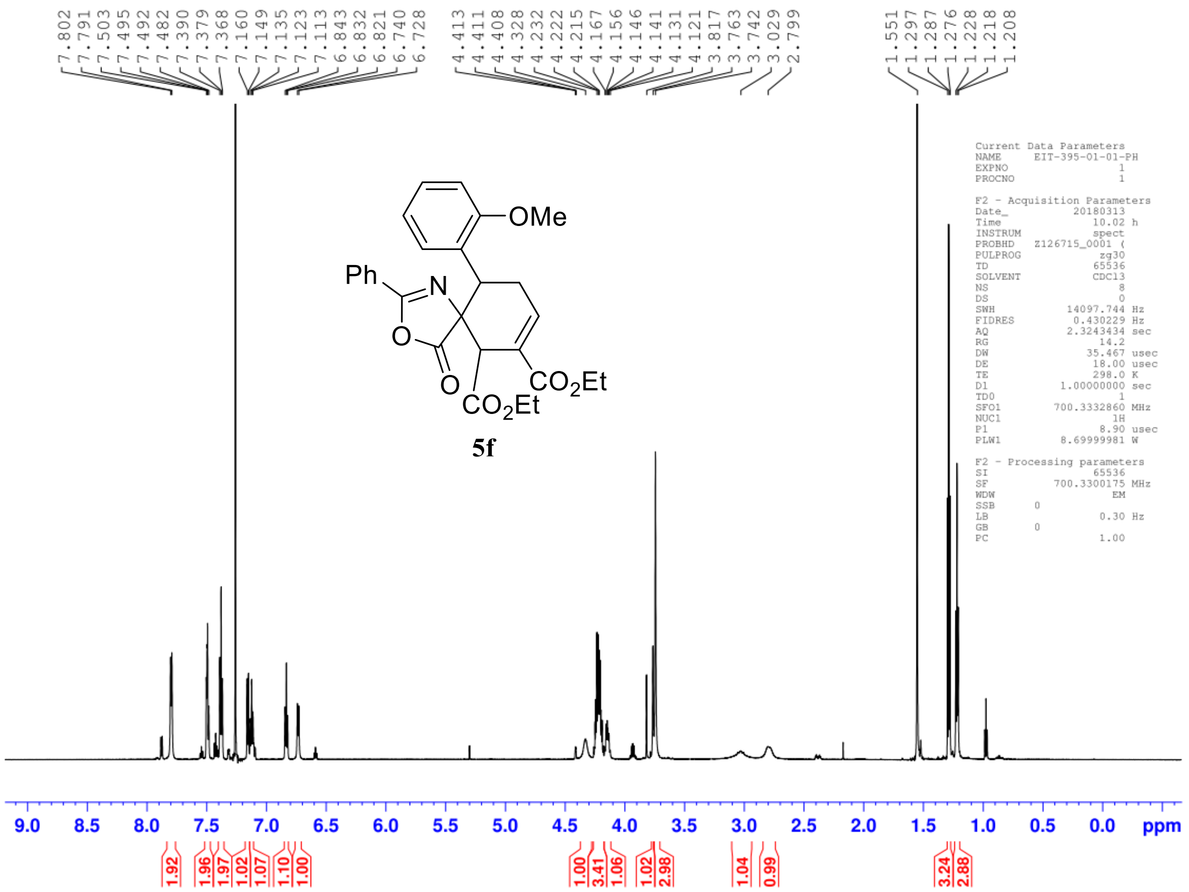
7.802 7.791 7.503 7.495 7.492 7.482 7.390 7.379 7.368 7.160 7.149 7.135 7.123 7.113 6.843 6.832 6.821 6.740 6.728
4.413 4.411 4.408 4.328 4.232 4.222 4.215 4.167 4.156 4.146 4.141 4.131 4.121 3.817 3.763 3.742 3.029 2.799
1.551 1.297 1.287 1.276 1.228 1.218 1.208
OMe
Ph
N
O
O
CO2Et
CO2Et
5f
9.0 8.5 8.0 7.5 7.0 6.5 6.0 5.5 5.0 4.5 4.0 3.5 3.0 2.5 2.0 1.5 1.0 0.5 0.0 ppm
1.92 1.96 1.97 1.02 1.07 1.10 1.00 1.00 3.41 1.06 1.02 2.98 1.04 0.99 3.24 2.88

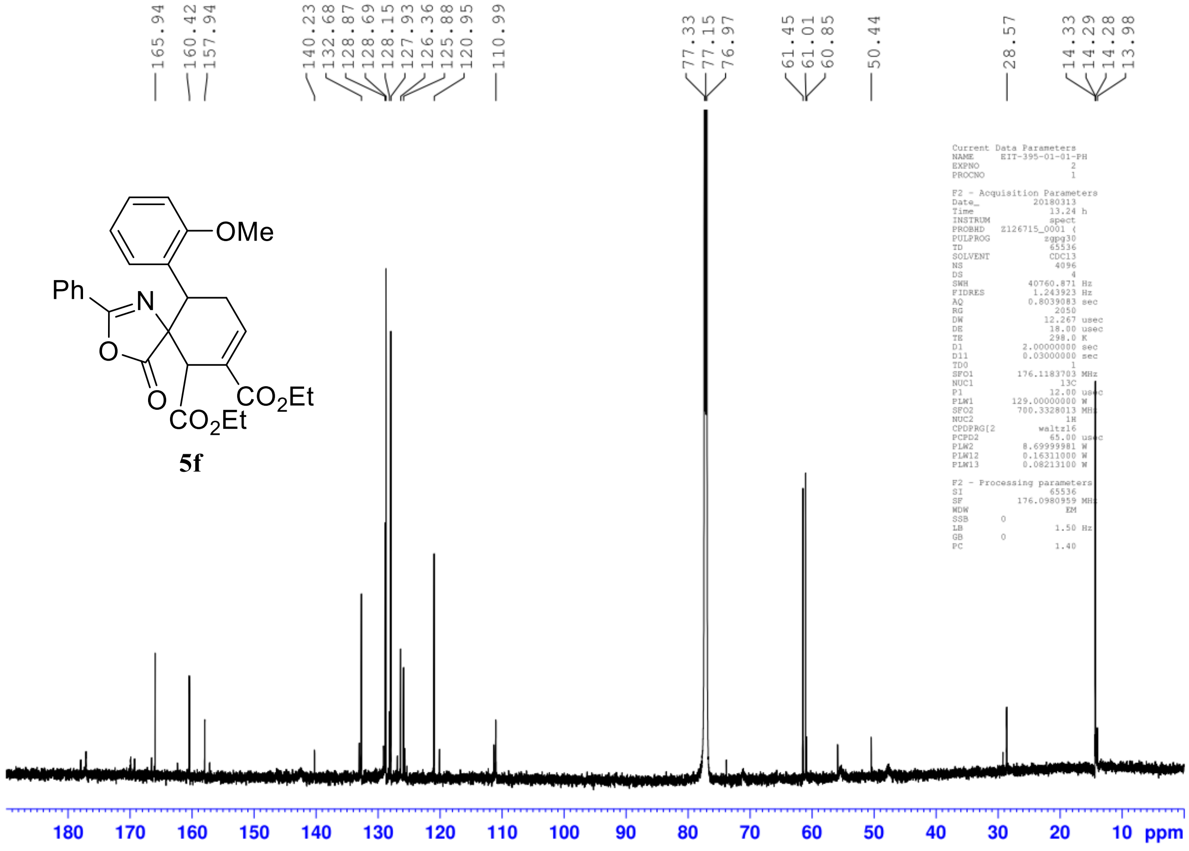
165.94 160.42 157.94
140.23 132.68 128.87 128.69 128.15 127.93 126.36 125.88 120.95 110.99
77.33 77.15 76.97
61.45 61.01 60.85 50.44 28.57 14.33 14.29 14.28 13.98
OMe
Ph
N
O
O
CO2Et
CO2Et
5f
180 170 160 150 140 130 120 110 100 90 80 70 60 50 40 30 20 10 ppm

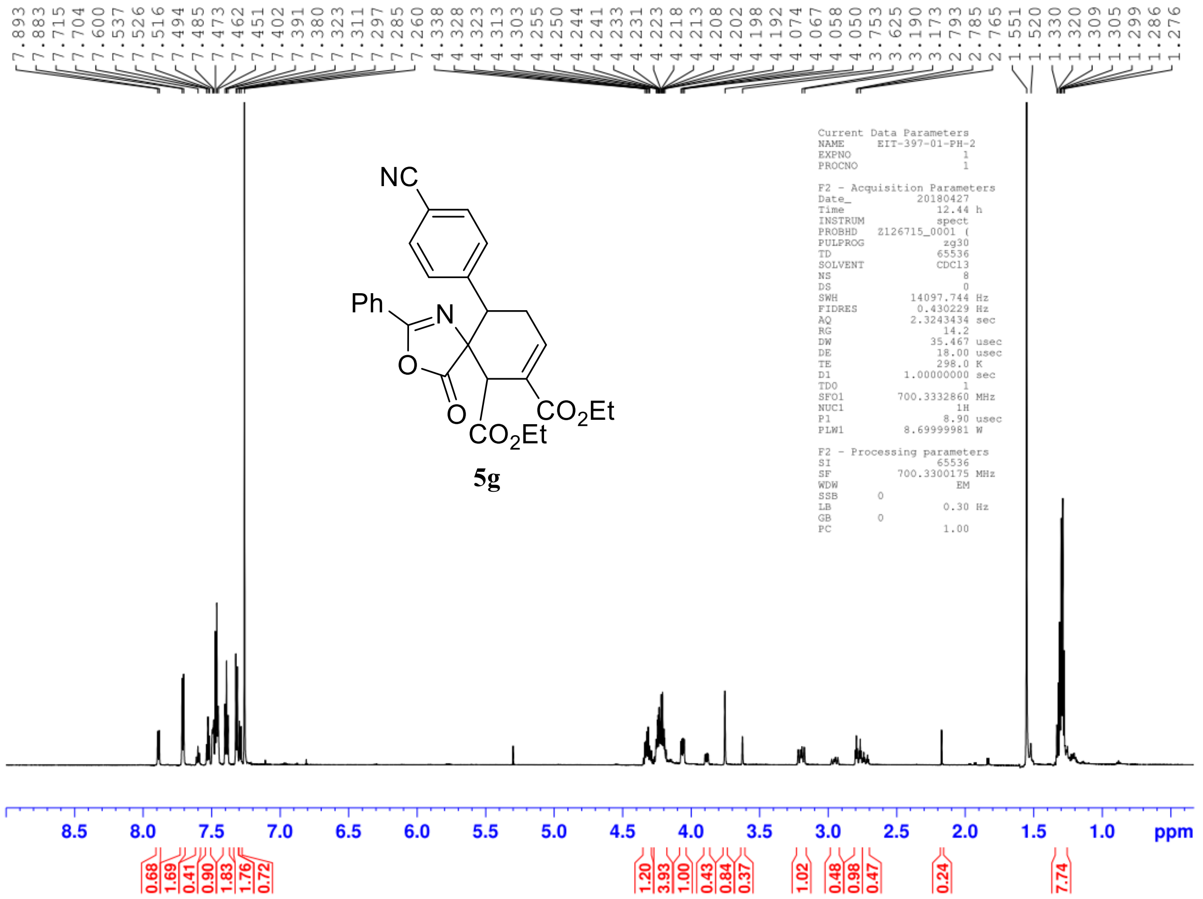

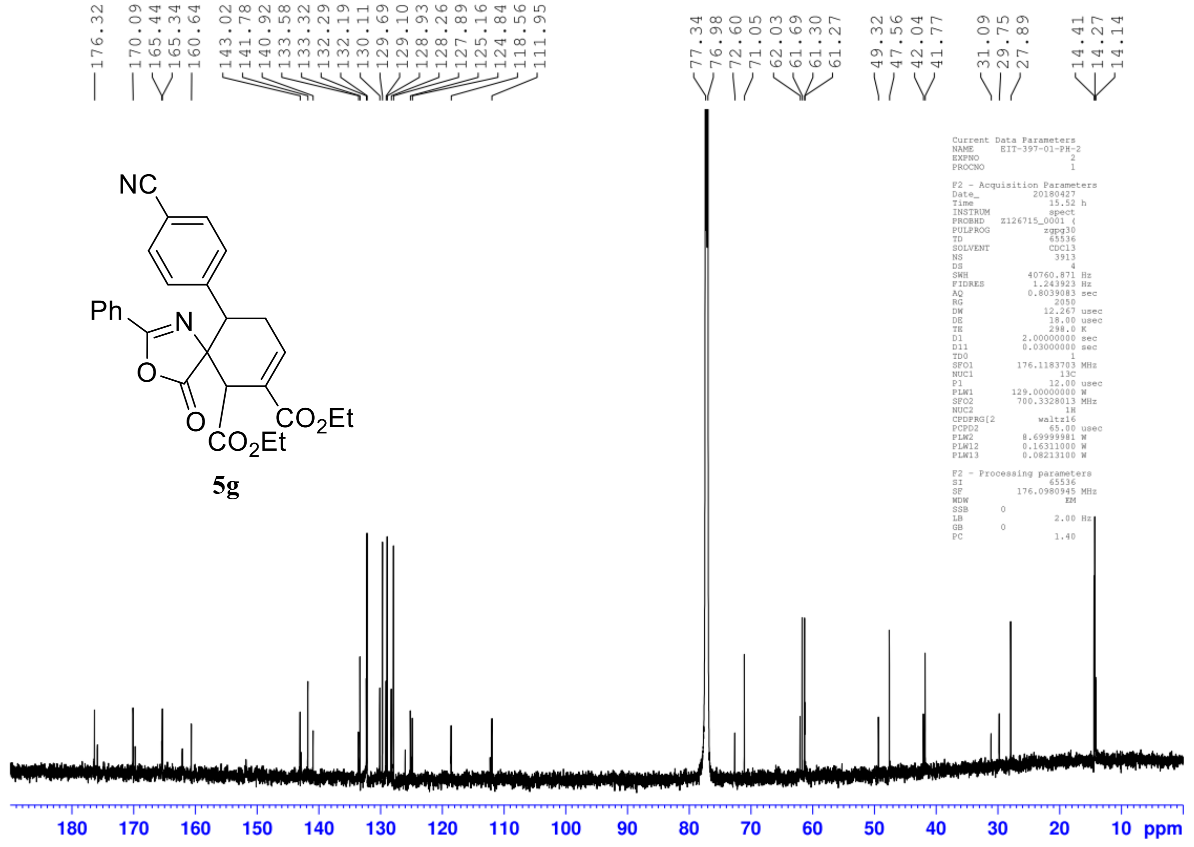

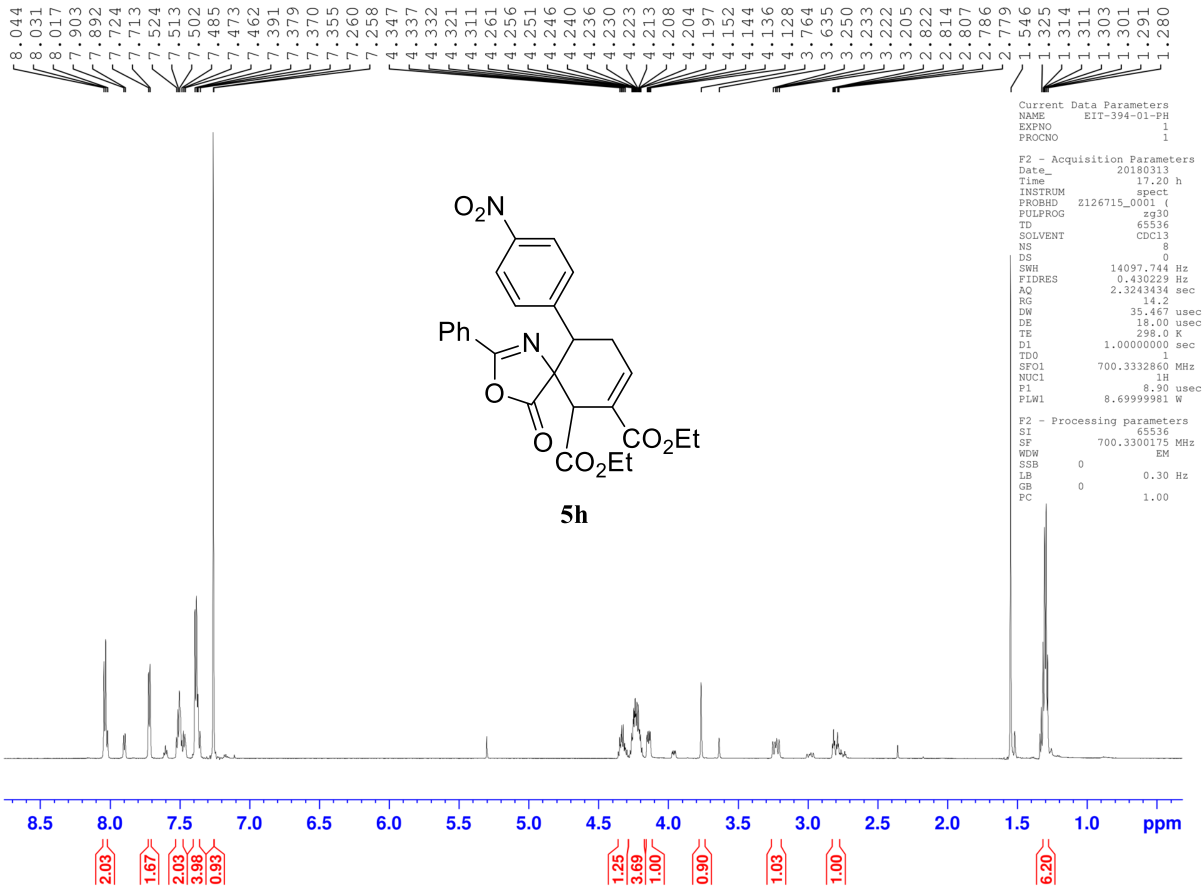

8.044
8.031
8.017
7.903
7.892
7.724
7.713
7.524
7.513
7.502
7.485
7.473
7.462
7.391
7.379
7.370
7.355
7.260
7.258
4.347
4.337
4.332
4.321
4.311
4.261
4.256
4.251
4.246
4.240
4.236
4.230
4.223
4.213
4.208
4.204
4.197
4.152
4.144
4.136
4.128
3.764
3.635
3.250
3.233
3.222
3.205
2.822
2.814
2.807
2.786
2.779
1.546
1.325
1.314
1.311
1.303
1.301
1.291
1.280
Current Data Parameters
NAME EIT-394-01-PH
EXPNO 1
PROCNO 1
F2 - Acquisition Parameters
Date_ 20180313
Time 17.20 h
INSTRUM spect
PROBHD Z126715_0001 (
PULPROG zg30
TD 65536
SOLVENT CDCl3
NS 8
DS 0
SWH 14097.744 Hz
FIDRES 0.430229 Hz
AQ 2.3243434 sec
RG 14.2
DW 35.467 usec
DE 18.00 usec
TE 298.0 K
D1 1.00000000 sec
TD0 1
SFO1 700.3332860 MHz
NUC1 1H
P1 8.90 usec
PLW1 8.69999981 W
F2 - Processing parameters
SI 65536
SF 700.3300175 MHz
WDW EM
SSB 0
LB 0.30 Hz
GB 0
PC 1.00
O2N
Ph
N
O
O
CO2Et
CO2Et
5h
8.5
8.0
7.5
7.0
6.5
6.0
5.5
5.0
4.5
4.0
3.5
3.0
2.5
2.0
1.5
1.0
ppm
2.03
1.67
2.03
3.98
0.93
1.25
3.69
1.00
0.90
1.03
1.00
6.20

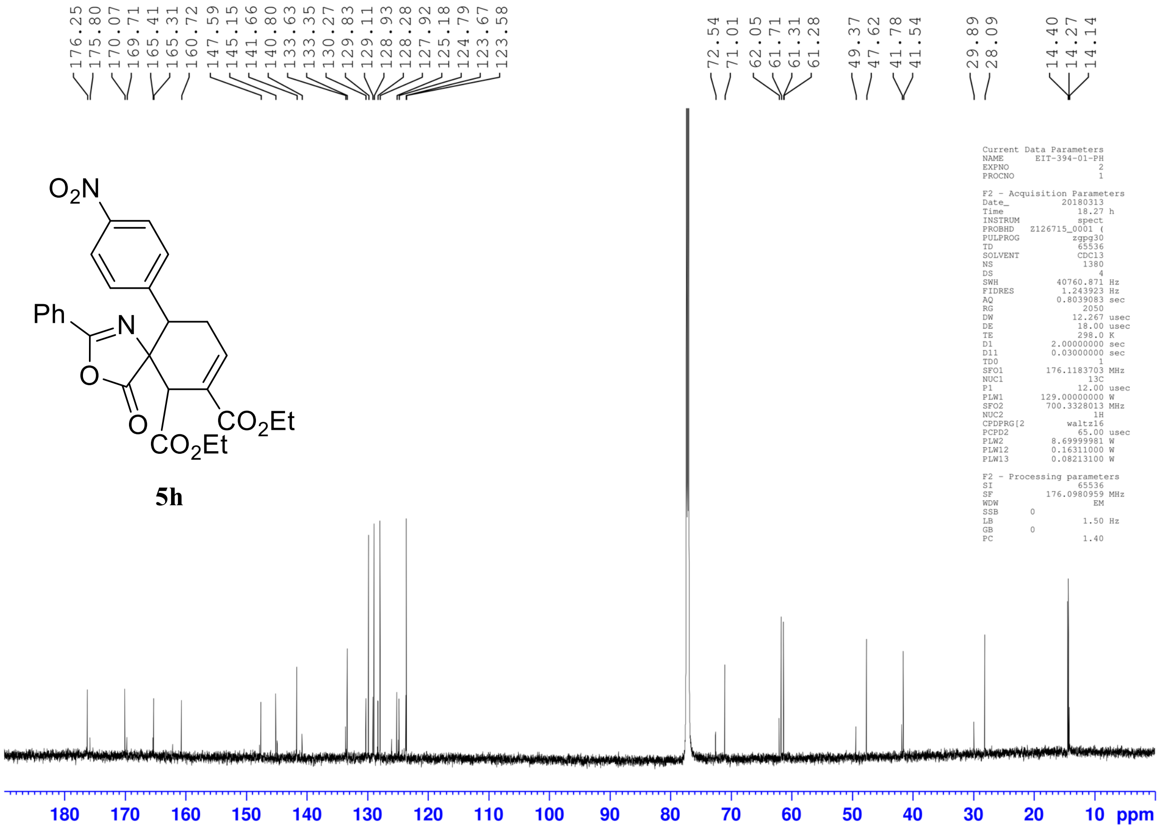

176.25
175.80
170.07
169.71
165.41
165.31
160.72
147.59
145.15
141.66
140.80
133.63
133.35
130.27
129.83
129.11
128.93
128.28
127.92
125.18
124.79
123.67
123.58
72.54
71.01
62.05
61.71
61.31
61.28
49.37
47.62
41.78
41.54
29.89
28.09
14.40
14.27
14.14
Current Data Parameters
NAME EIT-394-01-PH
EXPNO 2
PROCNO 1
F2 - Acquisition Parameters
Date_ 20180313
Time 18.27 h
INSTRUM spect
PROBHD Z126715_0001 (
PULPROG zgpg30
TD 65536
SOLVENT CDCl3
NS 1380
DS 4
SWH 40760.871 Hz
FIDRES 1.243923 Hz
AQ 0.8039083 sec
RG 2050
DW 12.267 usec
DE 18.00 usec
TE 298.0 K
D1 2.00000000 sec
D11 0.03000000 sec
TD0 1
SFO1 176.1183703 MHz
NUC1 13C
P1 12.00 usec
PLW1 129.00000000 W
SFO2 700.3328013 MHz
NUC2 1H
CPDPRG[2 waltz16
PCPD2 65.00 usec
PLW2 8.69999981 W
PLW12 0.16311000 W
PLW13 0.08213100 W
F2 - Processing parameters
SI 65536
SF 176.0980959 MHz
WDW EM
SSB 0
LB 1.50 Hz
GB 0
PC 1.40
O2N
Ph
N
O
O
CO2Et
CO2Et
5h
180
170
160
150
140
130
120
110
100
90
80
70
60
50
40
30
20
10
ppm

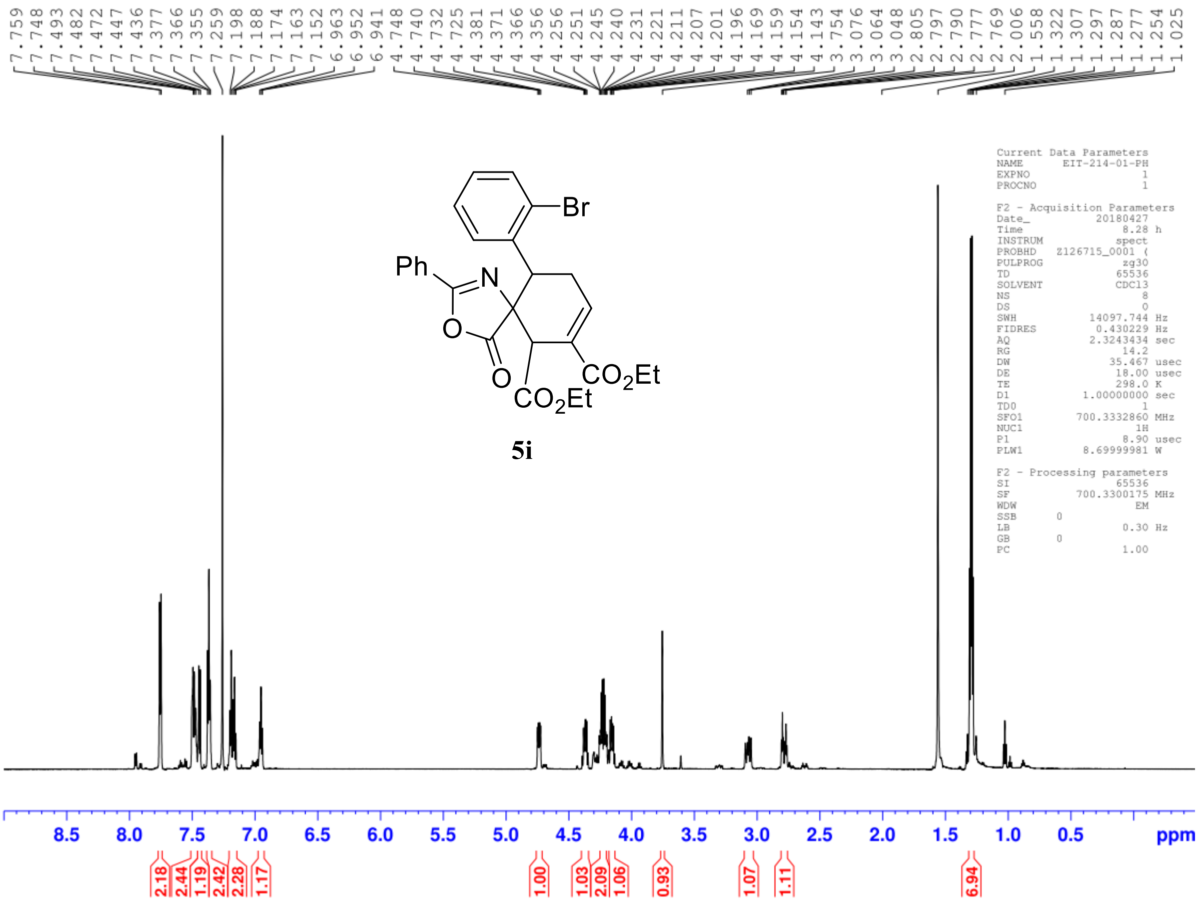

7.759 7.748 7.493 7.482 7.472 7.447 7.436 7.377 7.366 7.355 7.259 7.198 7.188 7.174 7.163 7.152 6.963 6.952 6.941 4.748 4.740 4.732 4.725 4.381 4.371 4.366 4.356 4.256 4.251 4.245 4.240 4.231 4.221 4.211 4.207 4.201 4.196 4.169 4.159 4.154 4.143 3.754 3.076 3.064 3.048 2.805 2.797 2.790 2.777 2.769 2.006 1.558 1.322 1.307 1.297 1.287 1.277 1.254 1.025
Br
Ph
N
O
O
CO2Et
CO2Et
5i
Current Data Parameters
NAME EIT-214-01-PH
EXPNO 1
PROCNO 1
F2 - Acquisition Parameters
Date_ 20180427
Time 8.28 h
INSTRUM spect
PROBHD Z126715_0001 (
PULPROG zg30
TD 65536
SOLVENT CDCl3
NS 8
DS 0
SWH 14097.744 Hz
FIDRES 0.430229 Hz
AQ 2.3243434 sec
RG 14.2
DW 35.467 usec
DE 18.00 usec
TE 298.0 K
D1 1.00000000 sec
TD0 1
SFO1 700.3332860 MHz
NUC1 1H
P1 8.90 usec
PLW1 8.69999981 W
F2 - Processing parameters
SI 65536
SF 700.3300175 MHz
WDW EM
SSB 0
LB 0.30 Hz
GB 0
PC 1.00
8.5 8.0 7.5 7.0 6.5 6.0 5.5 5.0 4.5 4.0 3.5 3.0 2.5 2.0 1.5 1.0 0.5 ppm
2.18 2.44 1.19 2.42 2.28 1.17 1.00 1.03 2.09 1.06 0.93 1.07 1.11 6.94

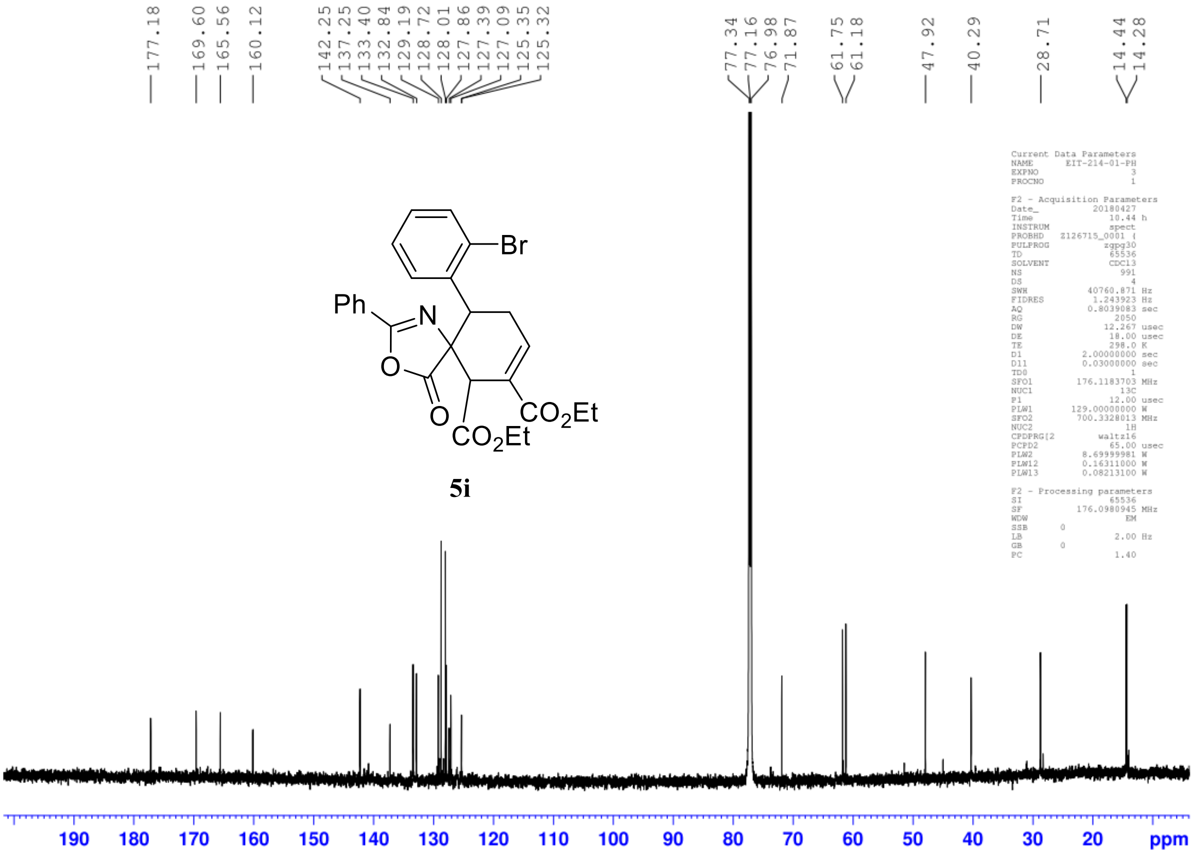

177.18 169.60 165.56 160.12 142.25 137.25 133.40 132.84 129.19 128.72 128.01 127.86 127.39 127.09 125.35 125.32 77.34 77.16 76.98 71.87 61.75 61.18 47.92 40.29 28.71 14.44 14.28
Br
Ph
N
O
O
CO2Et
CO2Et
5i
Current Data Parameters
NAME EIT-214-01-PH
EXPNO 3
PROCNO 1
F2 - Acquisition Parameters
Date_ 20180427
Time 10.44 h
INSTRUM spect
PROBHD Z126715_0001 (
PULPROG zgpg30
TD 65536
SOLVENT CDCl3
NS 991
DS 4
SWH 40760.871 Hz
FIDRES 1.243923 Hz
AQ 0.8039083 sec
RG 2050
DW 12.267 usec
DE 18.00 usec
TE 298.0 K
D1 2.00000000 sec
D11 0.03000000 sec
TD0 1
SFO1 176.1183703 MHz
NUC1 13C
P1 12.00 usec
PLW1 129.00000000 W
SFO2 700.3328013 MHz
NUC2 1H
CPDPRG[2 waltz16
PCPD2 65.00 usec
PLW2 8.69999981 W
PLW12 0.16311000 W
PLW13 0.08213100 W
F2 - Processing parameters
SI 65536
SF 176.0980945 MHz
WDW EM
SSB 0
LB 2.00 Hz
GB 0
PC 1.40
190 180 170 160 150 140 130 120 110 100 90 80 70 60 50 40 30 20 ppm

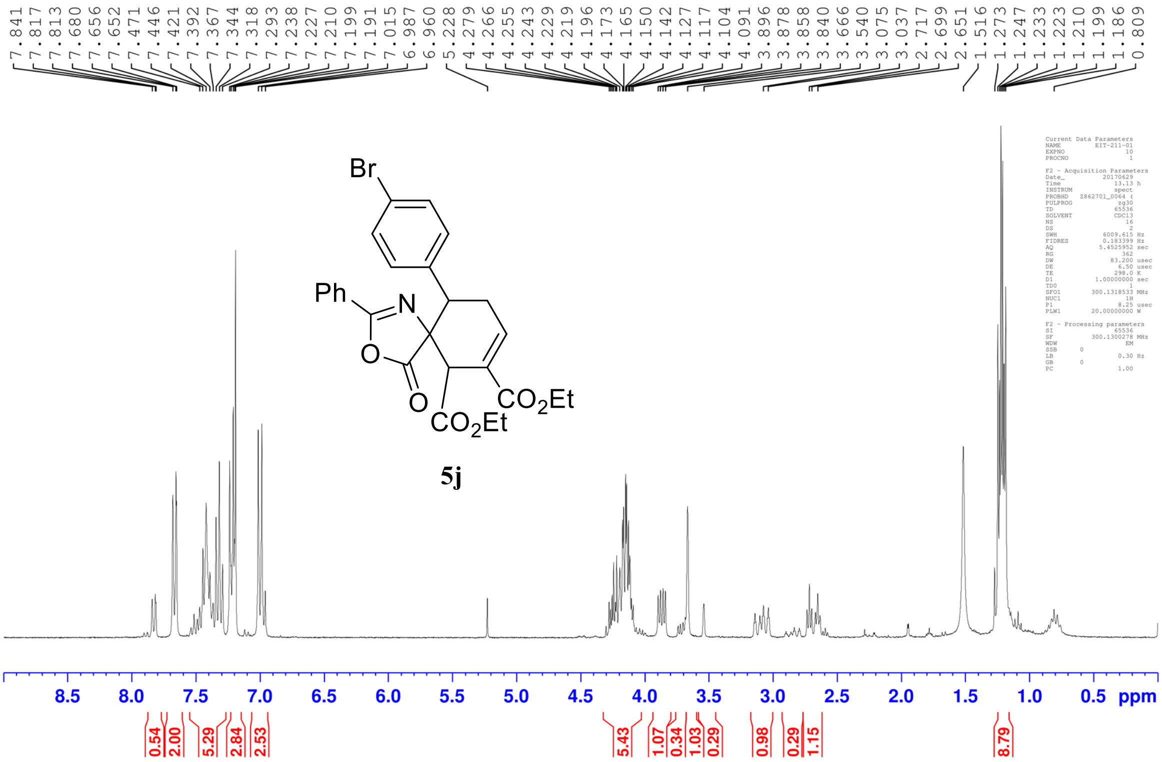
7.841 7.817 7.813 7.680 7.656 7.652 7.471 7.446 7.421 7.392 7.367 7.344 7.318 7.293 7.238 7.227 7.210 7.199 7.191 7.015 6.987 6.960 5.228 4.279 4.266 4.255 4.243 4.229 4.219 4.196 4.173 4.165 4.150 4.142 4.127 4.117 4.104 4.091 3.896 3.878 3.858 3.840 3.666 3.540 3.075 3.037 2.717 2.699 2.651 1.516 1.273 1.247 1.233 1.223 1.210 1.199 1.186 0.809
Br
Ph
N
O
O
CO2Et
CO2Et
5j
8.5 8.0 7.5 7.0 6.5 6.0 5.5 5.0 4.5 4.0 3.5 3.0 2.5 2.0 1.5 1.0 0.5 ppm
0.54 2.00 5.29 2.84 2.53 5.43 1.07 0.34 1.03 0.29 0.98 0.29 1.15 8.79

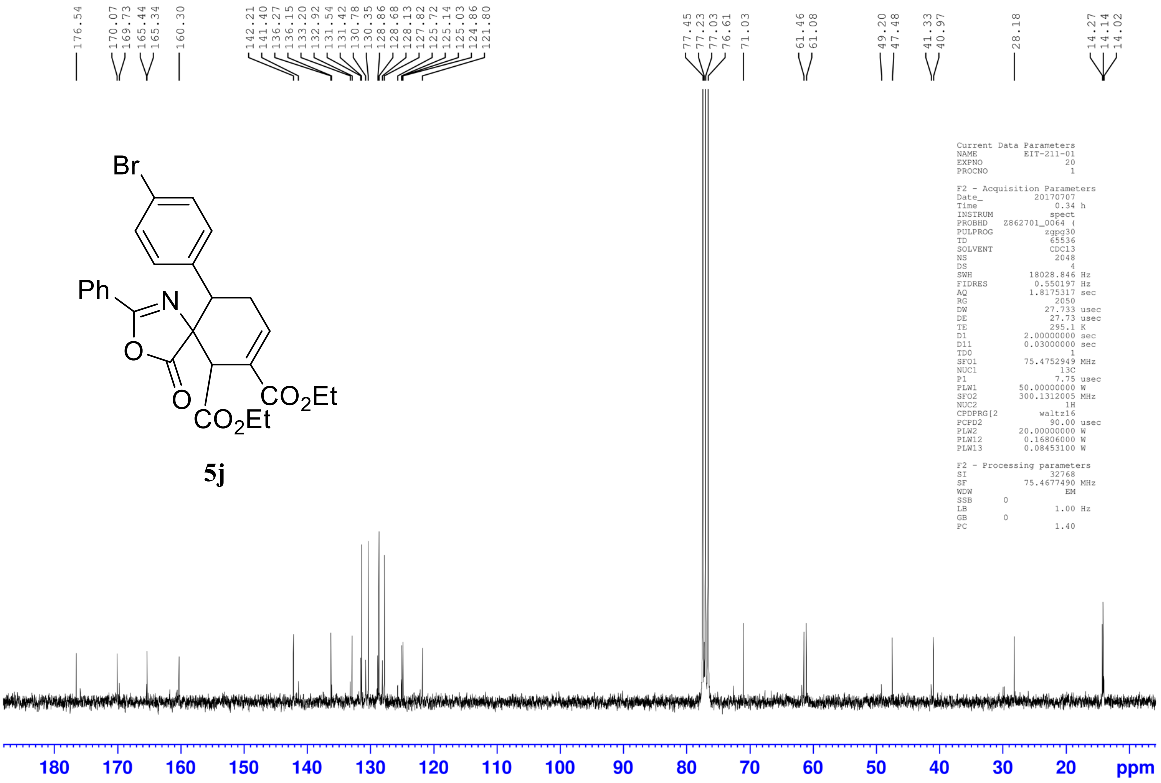
176.54 170.07 169.73 165.44 165.34 160.30 142.21 141.40 136.27 136.15 133.20 132.92 131.54 131.42 130.78 130.35 128.86 128.68 128.13 127.82 125.72 125.14 125.03 124.86 121.80 77.45 77.23 77.03 76.61 71.03 61.46 61.08 49.20 47.48 41.33 40.97 28.18 14.27 14.14 14.02
Br
Ph
N
O
O
CO2Et
CO2Et
5j
180 170 160 150 140 130 120 110 100 90 80 70 60 50 40 30 20 ppm

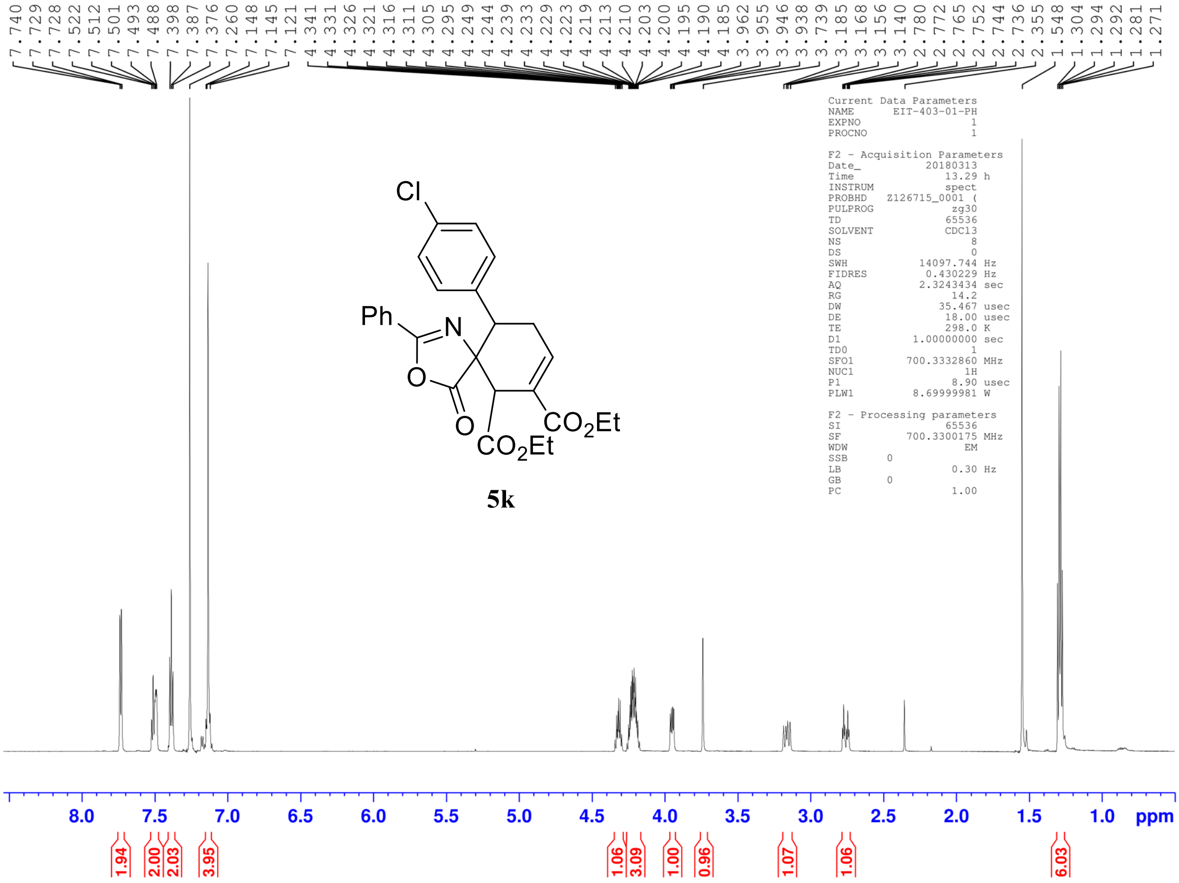
5k
ppm

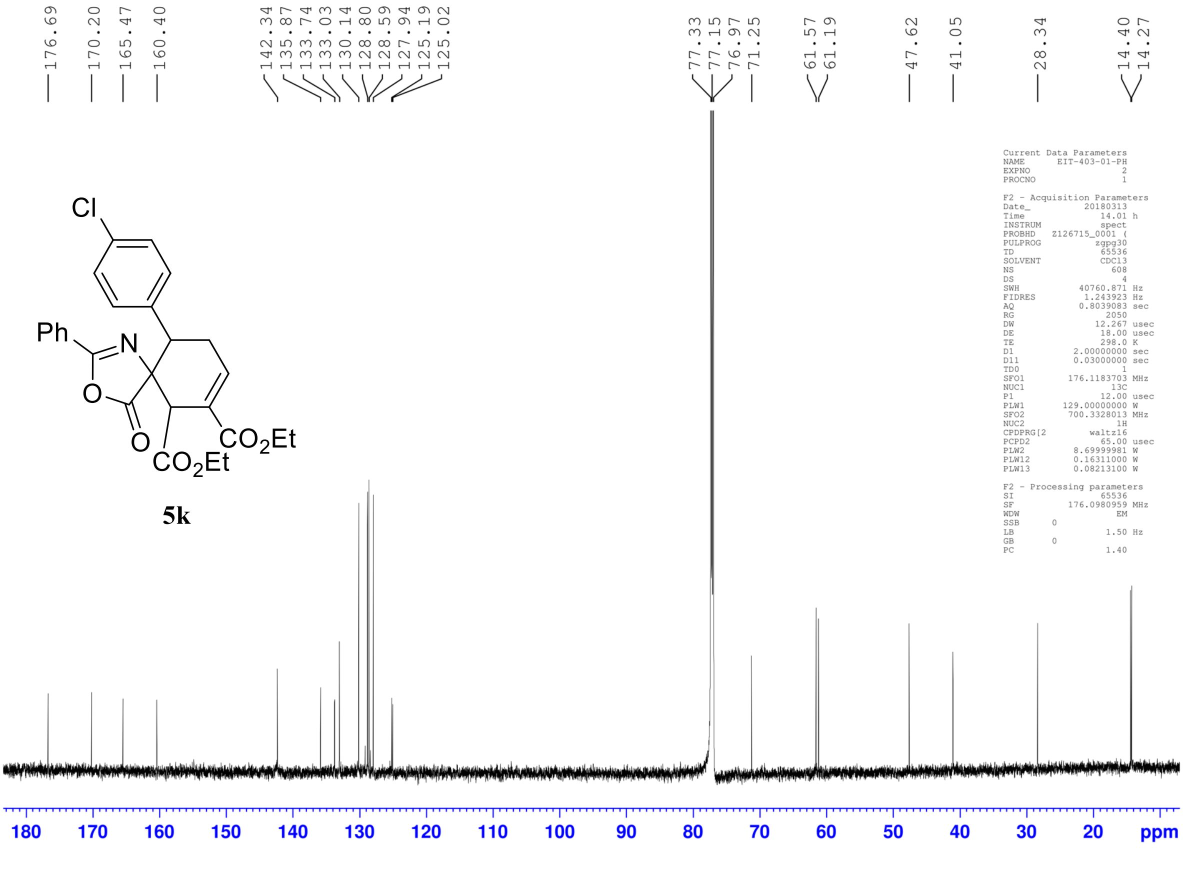
5k
ppm

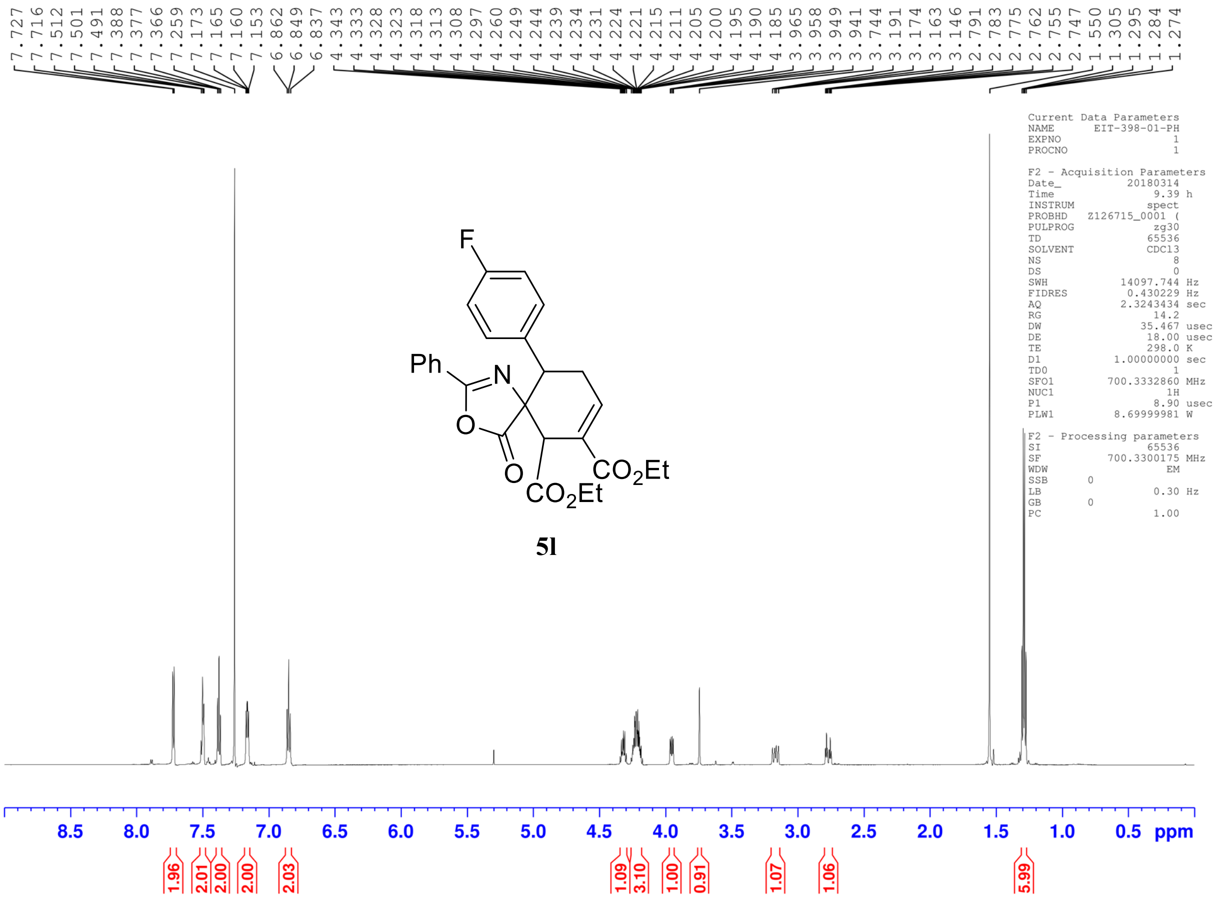
7.727
7.716
7.512
7.501
7.491
7.388
7.377
7.366
7.259
7.173
7.165
7.160
7.153
6.862
6.849
6.837
4.343
4.333
4.328
4.323
4.318
4.313
4.308
4.297
4.260
4.249
4.244
4.239
4.234
4.231
4.224
4.221
4.215
4.211
4.205
4.200
4.195
4.190
4.185
3.965
3.958
3.949
3.941
3.744
3.191
3.174
3.163
3.146
2.791
2.783
2.775
2.762
2.755
2.747
1.550
1.305
1.295
1.284
1.274
Current Data Parameters
NAME EIT-398-01-PH
EXPNO 1
PROCNO 1
F2 - Acquisition Parameters
Date_ 20180314
Time 9.39 h
INSTRUM spect
PROBHD Z126715_0001 (
PULPROG zg30
TD 65536
SOLVENT CDCl3
NS 8
DS 0
SWH 14097.744 Hz
FIDRES 0.430229 Hz
AQ 2.3243434 sec
RG 14.2
DW 35.467 usec
DE 18.00 usec
TE 298.0 K
D1 1.00000000 sec
TD0 1
SFO1 700.3332860 MHz
NUC1 1H
P1 8.90 usec
PLW1 8.69999981 W
F2 - Processing parameters
SI 65536
SF 700.3300175 MHz
WDW EM
SSB 0
LB 0.30 Hz
GB 0
PC 1.00
F
Ph
N
O
O
CO2Et
CO2Et
5l
8.5
8.0
7.5
7.0
6.5
6.0
5.5
5.0
4.5
4.0
3.5
3.0
2.5
2.0
1.5
1.0
0.5
ppm
1.96
2.01
2.00
2.00
2.03
1.09
3.10
1.00
0.91
1.07
1.06
5.99

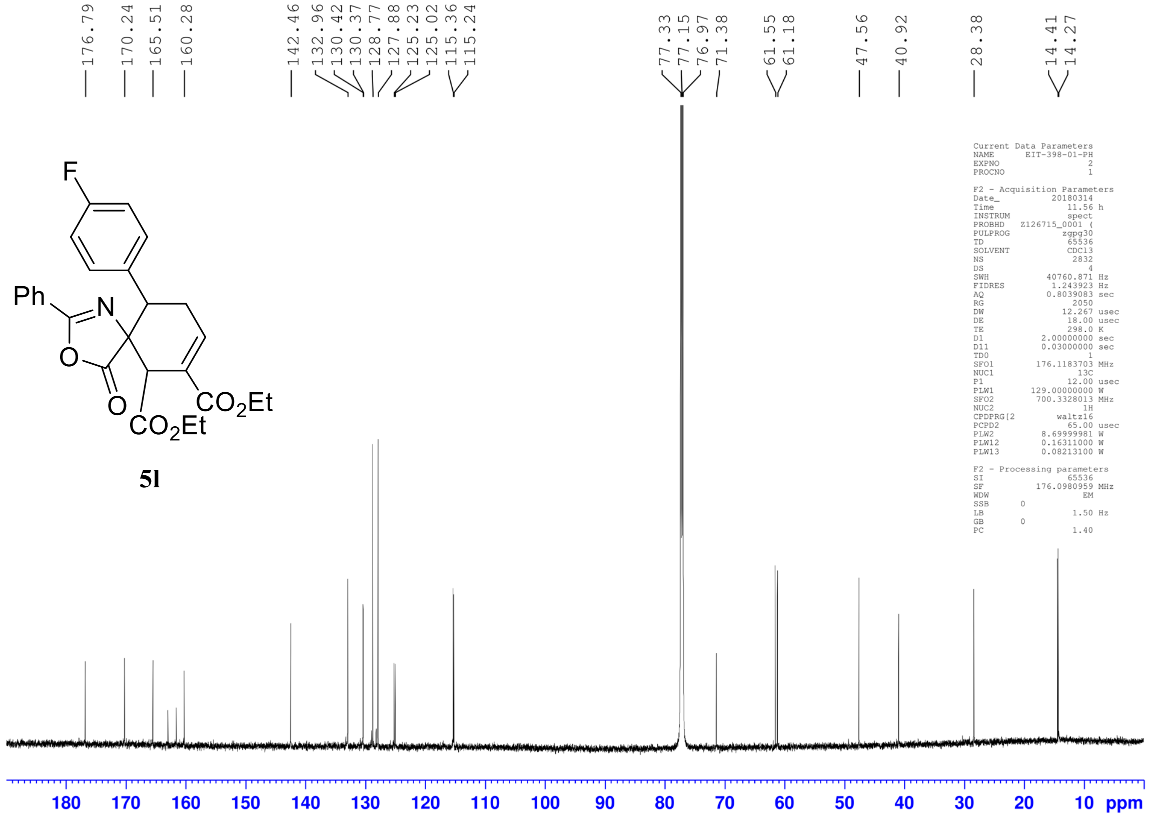
176.79
170.24
165.51
160.28
142.46
132.96
130.42
130.37
128.77
127.88
125.23
125.02
115.36
115.24
77.33
77.15
76.97
71.38
61.55
61.18
47.56
40.92
28.38
14.41
14.27
Current Data Parameters
NAME EIT-398-01-PH
EXPNO 2
PROCNO 1
F2 - Acquisition Parameters
Date_ 20180314
Time 11.56 h
INSTRUM spect
PROBHD Z126715_0001 (
PULPROG zgpg30
TD 65536
SOLVENT CDCl3
NS 2832
DS 4
SWH 40760.871 Hz
FIDRES 1.243923 Hz
AQ 0.8039083 sec
RG 2050
DW 12.267 usec
DE 18.00 usec
TE 298.0 K
D1 2.00000000 sec
D11 0.03000000 sec
TD0 1
SFO1 176.1183703 MHz
NUC1 13C
P1 12.00 usec
PLW1 129.00000000 W
SFO2 700.3328013 MHz
NUC2 1H
CPDPRG[2 waltz16
PCPD2 65.00 usec
PLW2 8.69999981 W
PLW12 0.16311000 W
PLW13 0.08213100 W
F2 - Processing parameters
SI 65536
SF 176.0980959 MHz
WDW EM
SSB 0
LB 1.50 Hz
GB 0
PC 1.40
F
Ph
N
O
O
CO2Et
CO2Et
5l
180
170
160
150
140
130
120
110
100
90
80
70
60
50
40
30
20
10
ppm

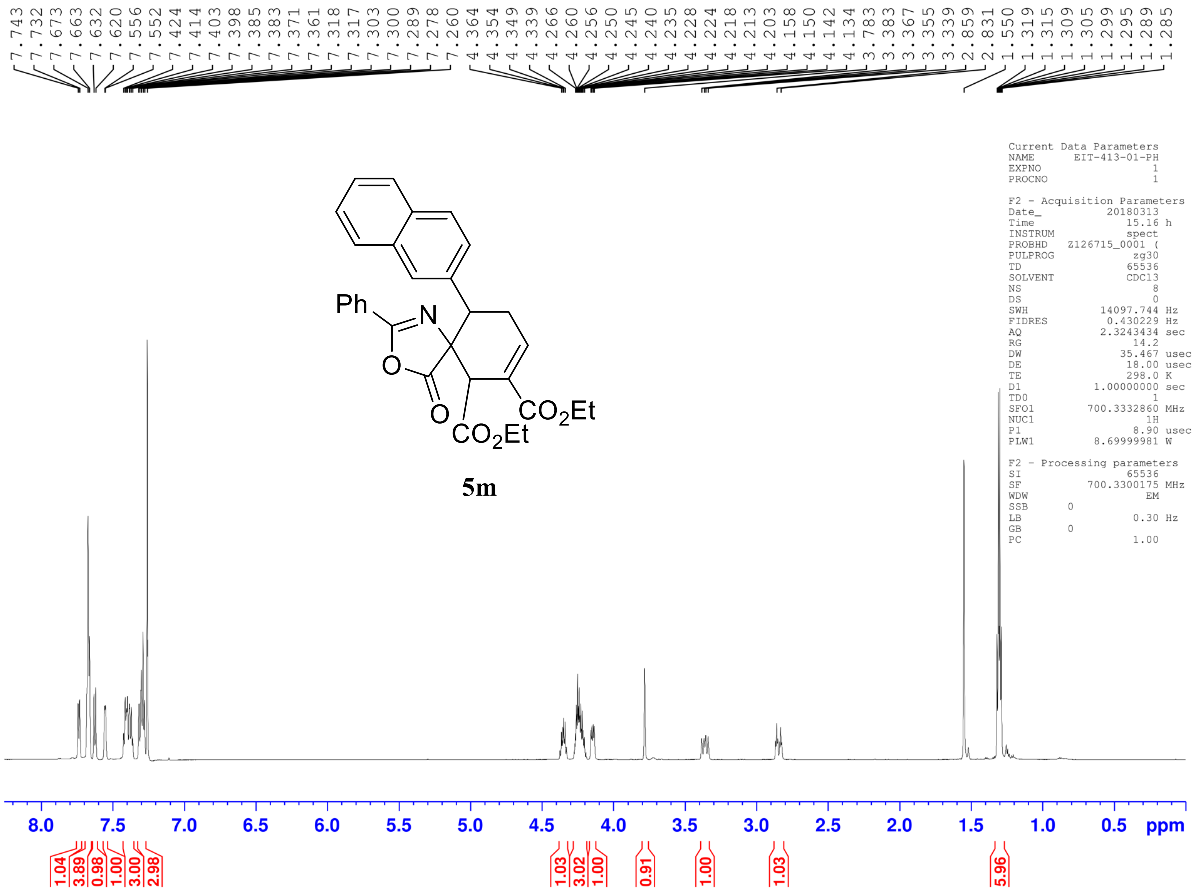
7.743 7.732 7.673 7.663 7.632 7.620 7.556 7.552 7.424 7.414 7.403 7.398 7.385 7.383 7.371 7.361 7.318 7.317 7.303 7.300 7.289 7.278 7.260 4.364 4.354 4.349 4.339 4.266 4.260 4.256 4.250 4.245 4.240 4.235 4.228 4.224 4.218 4.213 4.203 4.158 4.150 4.142 4.134 3.783 3.383 3.367 3.355 3.339 2.859 2.831 1.550 1.319 1.315 1.309 1.305 1.299 1.295 1.289 1.285
Ph
N
O
O
CO2Et
CO2Et
5m
Current Data Parameters
NAME EIT-413-01-PH
EXPNO 1
PROCNO 1
F2 - Acquisition Parameters
Date_ 20180313
Time 15.16 h
INSTRUM spect
PROBHD Z126715_0001 (
PULPROG zg30
TD 65536
SOLVENT CDCl3
NS 8
DS 0
SWH 14097.744 Hz
FIDRES 0.430229 Hz
AQ 2.3243434 sec
RG 14.2
DW 35.467 usec
DE 18.00 usec
TE 298.0 K
D1 1.00000000 sec
TD0 1
SFO1 700.3332860 MHz
NUC1 1H
P1 8.90 usec
PLW1 8.69999981 W
F2 - Processing parameters
SI 65536
SF 700.3300175 MHz
WDW EM
SSB 0
LB 0.30 Hz
GB 0
PC 1.00
8.0 7.5 7.0 6.5 6.0 5.5 5.0 4.5 4.0 3.5 3.0 2.5 2.0 1.5 1.0 0.5 ppm
1.04 3.89 0.98 1.00 3.00 2.98 1.03 3.02 1.00 0.91 1.00 1.03 5.96

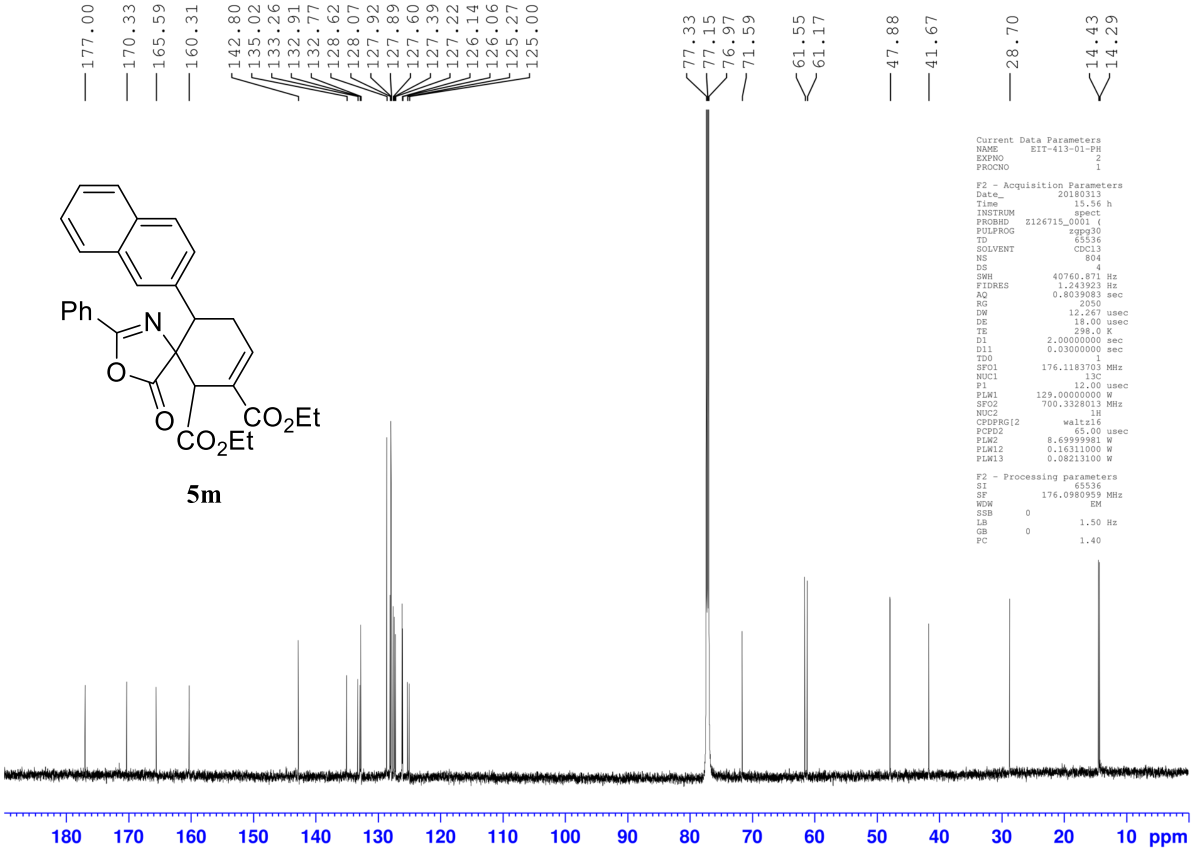
177.00 170.33 165.59 160.31 142.80 135.02 133.26 132.91 132.77 128.62 128.07 127.92 127.89 127.60 127.39 127.22 126.14 126.06 125.27 125.00 77.33 77.15 76.97 71.59 61.55 61.17 47.88 41.67 28.70 14.43 14.29
Ph
N
O
O
CO2Et
CO2Et
5m
Current Data Parameters
NAME EIT-413-01-PH
EXPNO 2
PROCNO 1
F2 - Acquisition Parameters
Date_ 20180313
Time 15.56 h
INSTRUM spect
PROBHD Z126715_0001 (
PULPROG zgpg30
TD 65536
SOLVENT CDCl3
NS 804
DS 4
SWH 40760.871 Hz
FIDRES 1.243923 Hz
AQ 0.8039083 sec
RG 2050
DW 12.267 usec
DE 18.00 usec
TE 298.0 K
D1 2.00000000 sec
D11 0.03000000 sec
TD0 1
SFO1 176.1183703 MHz
NUC1 13C
P1 12.00 usec
PLW1 129.00000000 W
SFO2 700.3328013 MHz
NUC2 1H
CPDPRG[2 waltz16
PCPD2 65.00 usec
PLW2 8.69999981 W
PLW12 0.16311000 W
PLW13 0.08213100 W
F2 - Processing parameters
SI 65536
SF 176.0980959 MHz
WDW EM
SSB 0
LB 1.50 Hz
GB 0
PC 1.40
180 170 160 150 140 130 120 110 100 90 80 70 60 50 40 30 20 10 ppm

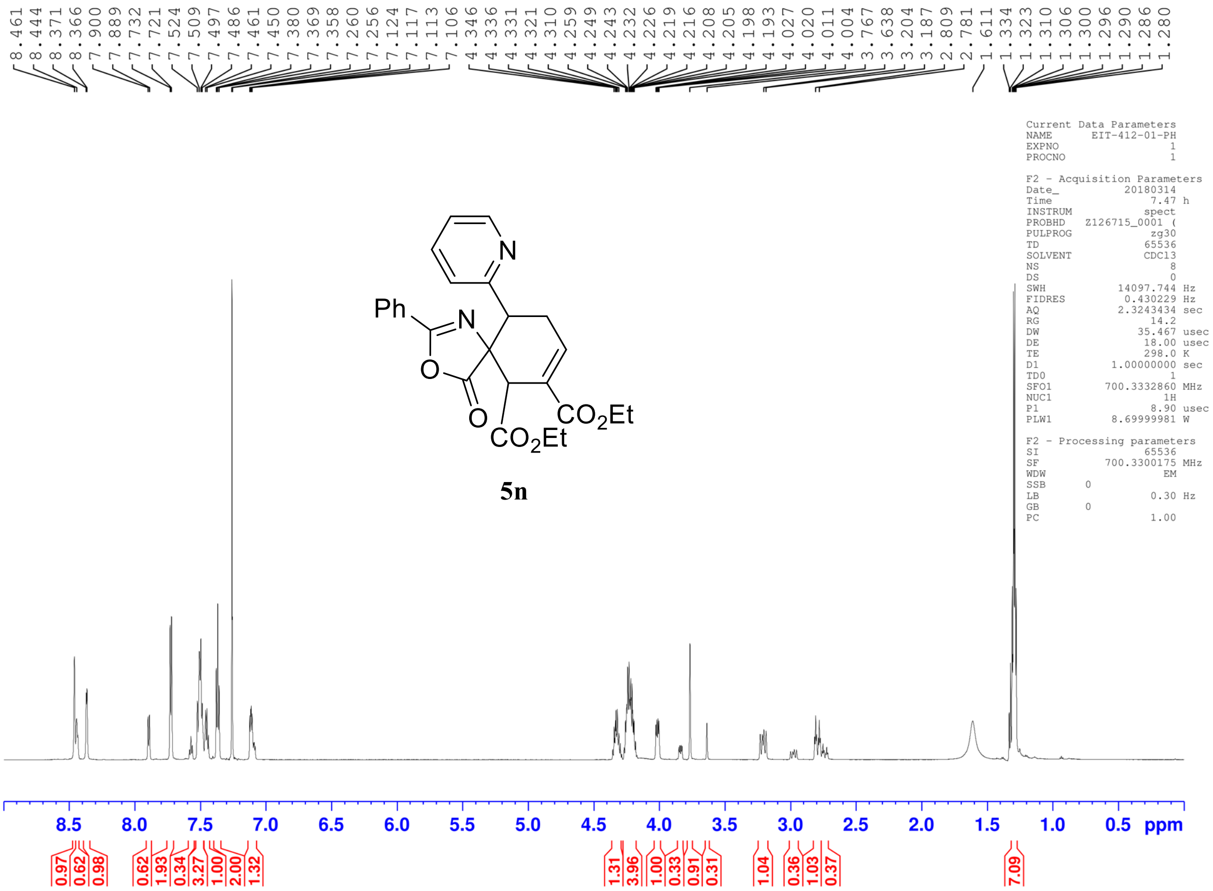
8.461 8.444 8.371 8.366 7.900 7.889 7.732 7.721 7.524 7.509 7.497 7.486 7.461 7.450 7.380 7.369 7.358 7.260 7.256 7.124 7.117 7.113 7.106 4.346 4.336 4.331 4.321 4.310 4.259 4.249 4.243 4.232 4.226 4.219 4.216 4.208 4.205 4.198 4.193 4.027 4.020 4.011 4.004 3.767 3.638 3.204 3.187 2.809 2.781 1.611 1.334 1.323 1.310 1.306 1.300 1.296 1.290 1.286 1.280
Ph
N
O
CO2Et
5n
ppm

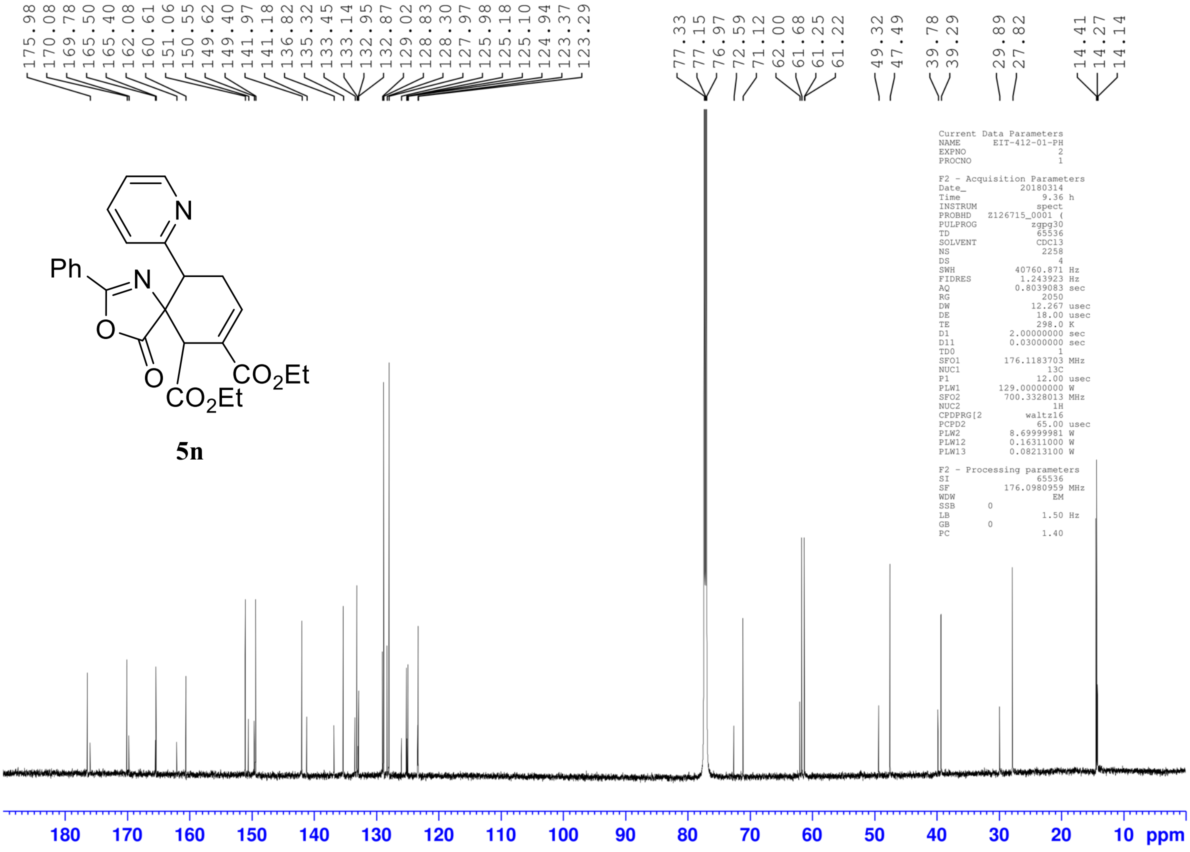
175.98 170.08 169.78 165.50 165.40 162.08 160.61 151.06 150.55 149.62 149.40 141.97 141.18 136.82 135.32 133.45 133.14 132.95 132.87 129.02 128.83 128.30 127.97 125.98 125.18 125.10 124.94 123.37 123.29 77.33 77.15 76.97 72.59 71.12 62.00 61.68 61.25 61.22 49.32 47.49 39.78 39.29 29.89 27.82 14.41 14.27 14.14
Ph
N
O
CO2Et
5n
ppm

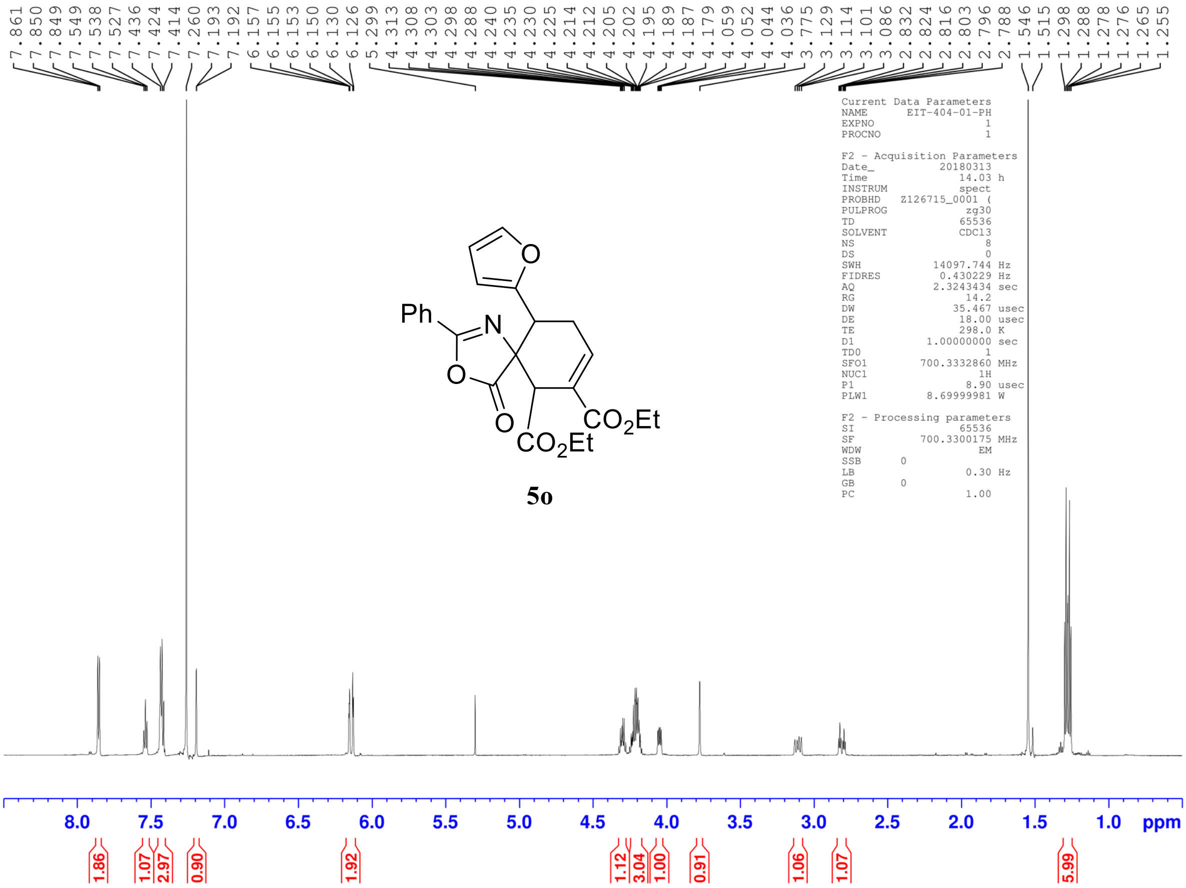
7.861 7.850 7.849 7.549 7.538 7.527 7.436 7.424 7.414 7.260 7.193 7.192 6.157 6.155 6.153 6.150 6.130 6.126 5.299 4.313 4.308 4.303 4.298 4.288 4.240 4.235 4.230 4.225 4.214 4.212 4.205 4.202 4.195 4.189 4.187 4.179 4.059 4.052 4.044 4.036 3.775 3.129 3.114 3.101 3.086 2.832 2.824 2.816 2.803 2.796 2.788 1.546 1.515 1.298 1.288 1.278 1.276 1.265 1.255
Current Data Parameters
NAME EIT-404-01-PH
EXPNO 1
PROCNO 1
F2 - Acquisition Parameters
Date_ 20180313
Time 14.03 h
INSTRUM spect
PROBHD Z126715_0001 (
PULPROG zg30
TD 65536
SOLVENT CDCl3
NS 8
DS 0
SWH 14097.744 Hz
FIDRES 0.430229 Hz
AQ 2.3243434 sec
RG 14.2
DW 35.467 usec
DE 18.00 usec
TE 298.0 K
D1 1.00000000 sec
TD0 1
SFO1 700.3332860 MHz
NUC1 1H
P1 8.90 usec
PLW1 8.69999981 W
F2 - Processing parameters
SI 65536
SF 700.3300175 MHz
WDW EM
SSB 0
LB 0.30 Hz
GB 0
PC 1.00
Ph
N
O
O
CO2Et
CO2Et
5o
8.0 7.5 7.0 6.5 6.0 5.5 5.0 4.5 4.0 3.5 3.0 2.5 2.0 1.5 1.0 ppm
1.86 1.07 2.97 0.90 1.92 1.12 3.04 1.00 0.91 1.06 1.07 5.99

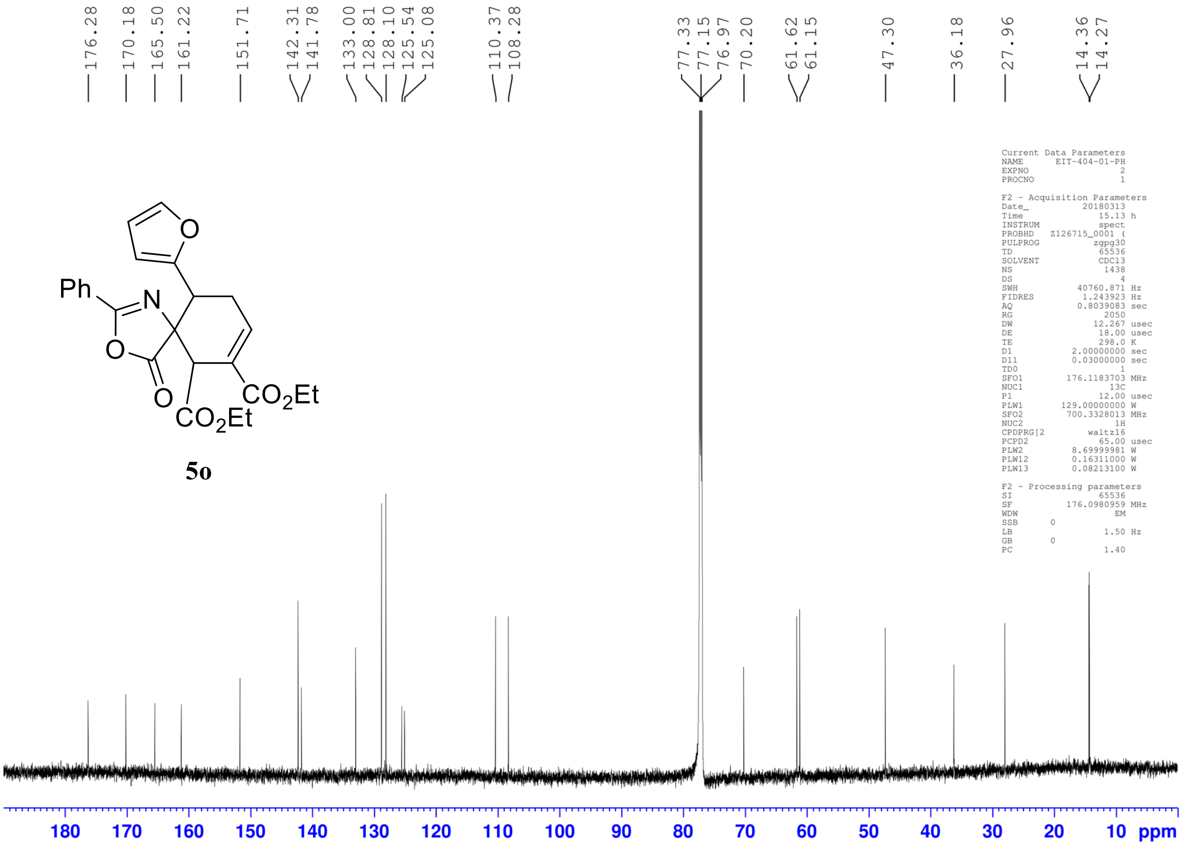
176.28 170.18 165.50 161.22 151.71 142.31 141.78 133.00 128.81 128.10 125.54 125.08 110.37 108.28 77.33 77.15 76.97 70.20 61.62 61.15 47.30 36.18 27.96 14.36 14.27
Current Data Parameters
NAME EIT-404-01-PH
EXPNO 2
PROCNO 1
F2 - Acquisition Parameters
Date_ 20180313
Time 15.13 h
INSTRUM spect
PROBHD Z126715_0001 (
PULPROG zgpg30
TD 65536
SOLVENT CDCl3
NS 1438
DS 4
SWH 40760.871 Hz
FIDRES 1.243923 Hz
AQ 0.8039083 sec
RG 2050
DW 12.267 usec
DE 18.00 usec
TE 298.0 K
D1 2.00000000 sec
D11 0.03000000 sec
TD0 1
SFO1 176.1183703 MHz
NUC1 13C
P1 12.00 usec
PLW1 129.00000000 W
SFO2 700.3328013 MHz
NUC2 1H
CPDPRG[2 waltz16
PCPD2 65.00 usec
PLW2 8.69999981 W
PLW12 0.16311000 W
PLW13 0.08213100 W
F2 - Processing parameters
SI 65536
SF 176.0980959 MHz
WDW EM
SSB 0
LB 1.50 Hz
GB 0
PC 1.40
Ph
N
O
O
CO2Et
CO2Et
5o
180 170 160 150 140 130 120 110 100 90 80 70 60 50 40 30 20 10 ppm

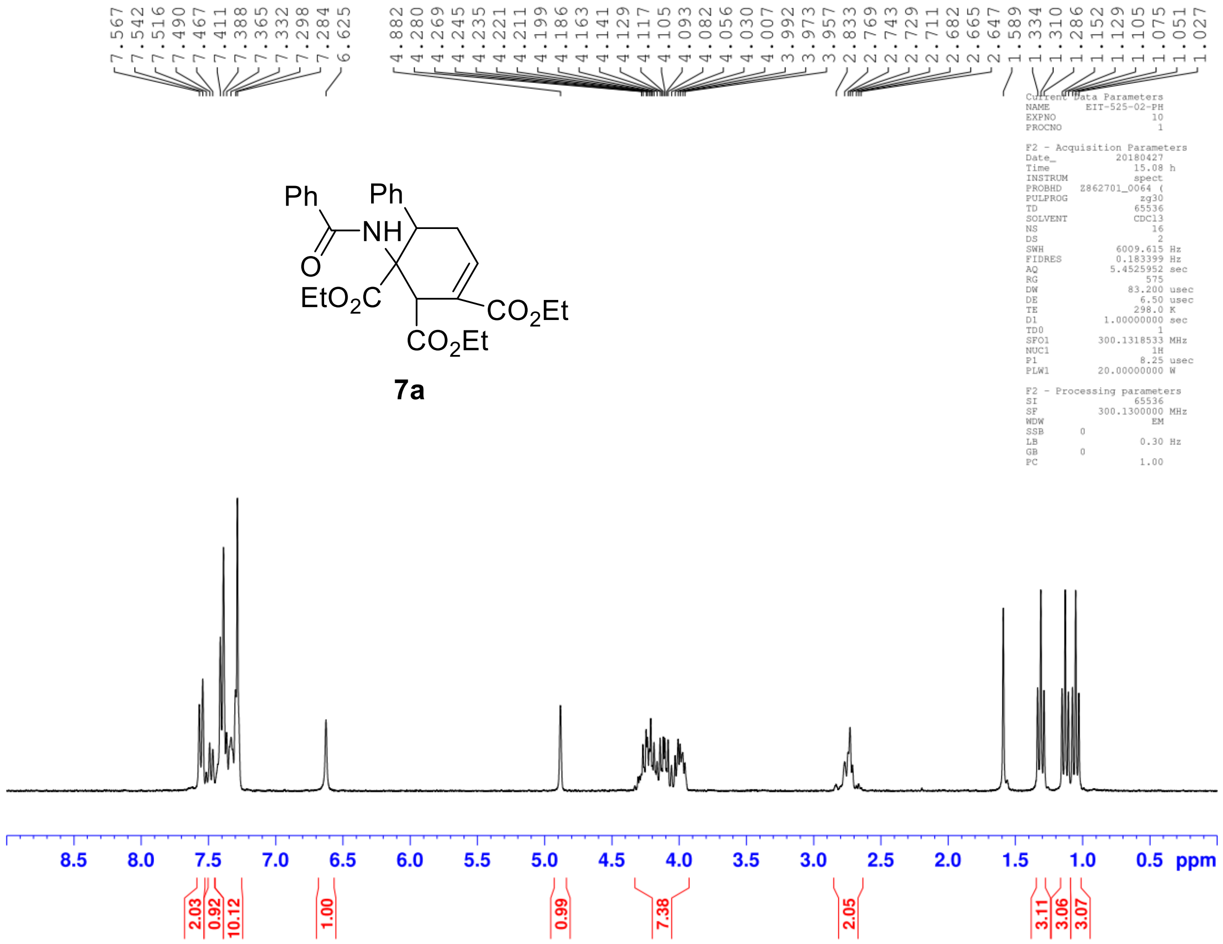
7.567 7.542 7.516 7.490 7.467 7.411 7.388 7.365 7.332 7.298 7.284 6.625
4.882 4.280 4.269 4.245 4.235 4.221 4.211 4.199 4.186 4.163 4.141 4.129 4.117 4.105 4.093 4.082 4.056 4.030 4.007 3.992 3.973 3.957
2.833 2.769 2.743 2.729 2.711 2.682 2.665 2.647
1.589 1.334 1.310 1.286 1.152 1.129 1.105 1.075 1.051 1.027
Ph
Ph
NH
O
EtO_2C
CO_2Et
CO_2Et
7a
Current Data Parameters
NAME EIT-525-02-PH
EXPNO 10
PROCNO 1
F2 - Acquisition Parameters
Date_ 20180427
Time 15.08 h
INSTRUM spect
PROBHD Z862701_0064 (
PULPROG zg30
TD 65536
SOLVENT CDCl3
NS 16
DS 2
SWH 6009.615 Hz
FIDRES 0.183399 Hz
AQ 5.4525952 sec
RG 575
DW 83.200 usec
DE 6.50 usec
TE 298.0 K
D1 1.00000000 sec
TD0 1
SFO1 300.1318533 MHz
NUC1 1H
P1 8.25 usec
PLW1 20.00000000 W
F2 - Processing parameters
SI 65536
SF 300.1300000 MHz
WDW EM
SSB 0
LB 0.30 Hz
GB 0
PC 1.00
8.5 8.0 7.5 7.0 6.5 6.0 5.5 5.0 4.5 4.0 3.5 3.0 2.5 2.0 1.5 1.0 0.5 ppm
2.03 0.92 10.12 1.00 0.99 7.38 2.05 3.11 3.06 3.07

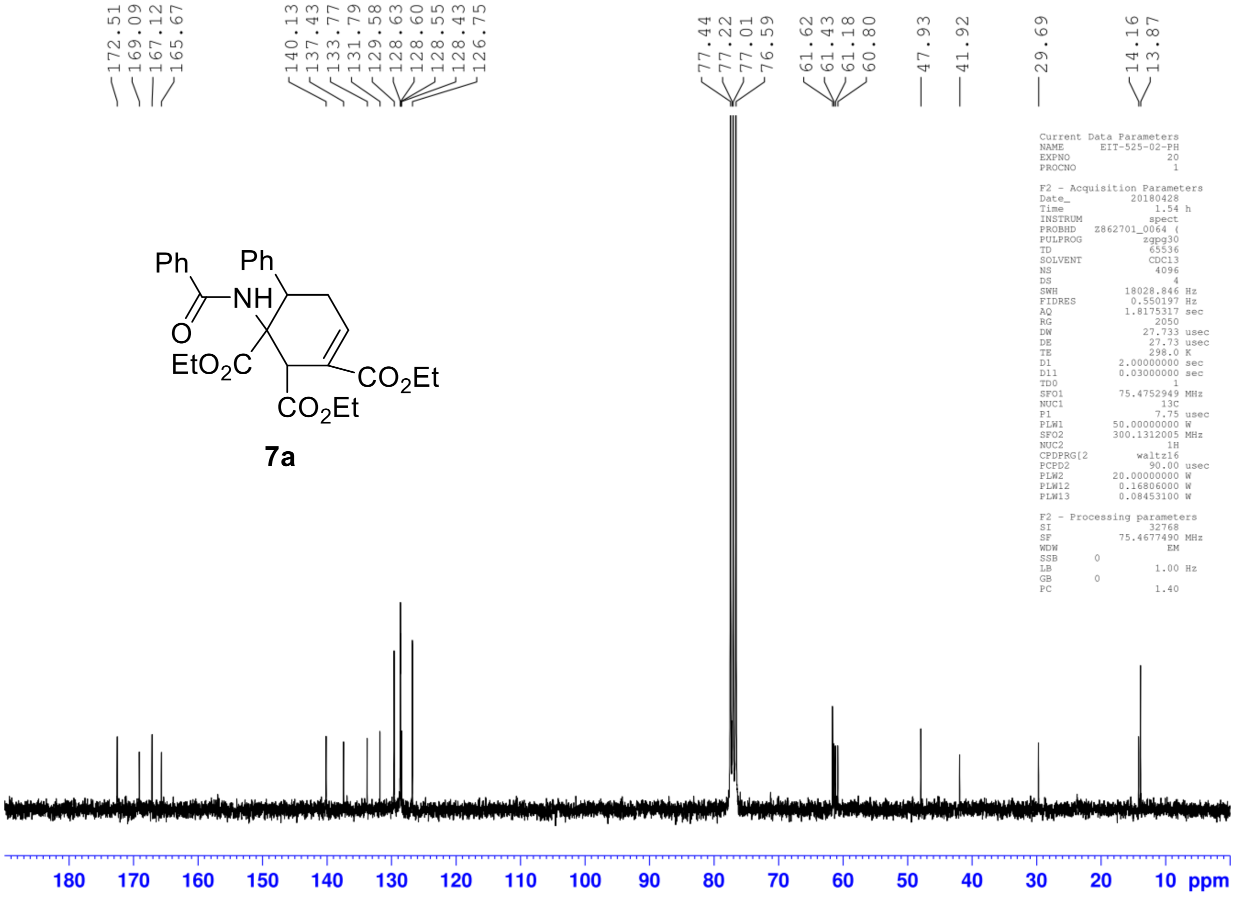
172.51 169.09 167.12 165.67
140.13 137.43 133.77 131.79 129.58 128.63 128.60 128.55 128.43 126.75
77.44 77.22 77.01 76.59
61.62 61.43 61.18 60.80
47.93
41.92
29.69
14.16 13.87
Ph
Ph
NH
O
EtO_2C
CO_2Et
CO_2Et
7a
Current Data Parameters
NAME EIT-525-02-PH
EXPNO 20
PROCNO 1
F2 - Acquisition Parameters
Date_ 20180428
Time 1.54 h
INSTRUM spect
PROBHD Z862701_0064 (
PULPROG zgpg30
TD 65536
SOLVENT CDCl3
NS 4096
DS 4
SWH 18028.846 Hz
FIDRES 0.550197 Hz
AQ 1.8175317 sec
RG 2050
DW 27.733 usec
DE 27.73 usec
TE 298.0 K
D1 2.00000000 sec
D11 0.03000000 sec
TD0 1
SFO1 75.4752949 MHz
NUC1 13C
P1 7.75 usec
PLW1 50.00000000 W
SFO2 300.1312005 MHz
NUC2 1H
CPDPRG[2 waltz16
PCPD2 90.00 usec
PLW2 20.00000000 W
PLW12 0.16806000 W
PLW13 0.08453100 W
F2 - Processing parameters
SI 32768
SF 75.4677490 MHz
WDW EM
SSB 0
LB 1.00 Hz
GB 0
PC 1.40
180 170 160 150 140 130 120 110 100 90 80 70 60 50 40 30 20 10 ppm
